# Genome of the Asian longhorned beetle (*Anoplophora glabripennis*), a globally significant invasive species, reveals key functional and evolutionary innovations at the beetle–plant interface

**DOI:** 10.1186/s13059-016-1088-8

**Published:** 2016-11-11

**Authors:** Duane D. McKenna, Erin D. Scully, Yannick Pauchet, Kelli Hoover, Roy Kirsch, Scott M. Geib, Robert F. Mitchell, Robert M. Waterhouse, Seung-Joon Ahn, Deanna Arsala, Joshua B. Benoit, Heath Blackmon, Tiffany Bledsoe, Julia H. Bowsher, André Busch, Bernarda Calla, Hsu Chao, Anna K. Childers, Christopher Childers, Dave J. Clarke, Lorna Cohen, Jeffery P. Demuth, Huyen Dinh, HarshaVardhan Doddapaneni, Amanda Dolan, Jian J. Duan, Shannon Dugan, Markus Friedrich, Karl M. Glastad, Michael A. D. Goodisman, Stephanie Haddad, Yi Han, Daniel S. T. Hughes, Panagiotis Ioannidis, J. Spencer Johnston, Jeffery W. Jones, Leslie A. Kuhn, David R. Lance, Chien-Yueh Lee, Sandra L. Lee, Han Lin, Jeremy A. Lynch, Armin P. Moczek, Shwetha C. Murali, Donna M. Muzny, David R. Nelson, Subba R. Palli, Kristen A. Panfilio, Dan Pers, Monica F. Poelchau, Honghu Quan, Jiaxin Qu, Ann M. Ray, Joseph P. Rinehart, Hugh M. Robertson, Richard Roehrdanz, Andrew J. Rosendale, Seunggwan Shin, Christian Silva, Alex S. Torson, Iris M. Vargas Jentzsch, John H. Werren, Kim C. Worley, George Yocum, Evgeny M. Zdobnov, Richard A. Gibbs, Stephen Richards

**Affiliations:** 1 Department of Biological Sciences, University of Memphis, 3700 Walker Ave., Memphis, TN 38152 USA; 2 Feinstone Center for Genomic Research, University of Memphis, Memphis, TN 38152 USA; 3 USDA, Agricultural Research Service, Center for Grain and Animal Health, Stored Product Insect and Engineering Research Unit, Manhattan, KS 66502 USA; 4 Department of Entomology, Max Planck Institute for Chemical Ecology, Jena, 07745, Germany; 5 Department of Entomology and Center for Chemical Ecology, The Pennsylvania State University, University Park, PA 16802 USA; 6 USDA, Agricultural Research Service, Daniel K Inouye US Pacific Basin Agricultural Research Center, Tropical Crop and Commodity Protection Research Unit, Hilo, HI 96720 USA; 7 Center for Insect Science and Department of Neuroscience, University of Arizona, Tucson, AZ 85721 USA; 8 Department of Biology, University of Wisconsin Oshkosh, Oshkosh, WI 54901 USA; 9 Department of Genetic Medicine and Development and Swiss Institute of Bioinformatics, University of Geneva, Geneva, 1211 Switzerland; 10 The Massachusetts Institute of Technology and The Broad Institute of MIT and Harvard, Cambridge, MA 02142 USA; 11 Department of Biological Sciences, University of Illinois at Chicago, Chicago, IL 60607 USA; 12 Department of Biological Sciences, University of Cincinnati, Cincinnati, OH 45221 USA; 13 Department of Biology, University of Texas at Arlington, Arlington, TX 76019 USA; 14 Department of Biological Sciences, North Dakota State University, Fargo, ND 58108 USA; 15 Human Genome Sequencing Center, Department of Human and Molecular Genetics, Baylor College of Medicine, One Baylor Plaza, Houston, TX 77030 USA; 16 USDA, Agricultural Research Service, Red River Valley Agricultural Research Center, Biosciences Research Laboratory, Fargo, ND 58102, USA; 17 USDA, Agricultural Research Service, National Agricultural Library, Beltsville, MD 20705 USA; 18 Department of Biology, University of Rochester, Rochester, NY 14627 USA; 19 USDA, Agricultural Research Service, Beneficial Insects Introduction Research, Newark, DE 19713 USA; 20 Department of Biological Sciences, Wayne State University, Detroit, MI 48202 USA; 21 School of Biology, Georgia Institute of Technology, Atlanta, GA 30332 USA; 22 Department of Entomology, Texas A&M University, College Station, TX 77843 USA; 23 Department of Biochemistry and Molecular Biology, Department of Computers Science and Engineering, and Department of Fisheries and Wildlife, Michigan State University, East Lansing, MI 48824 USA; 24 USDA, Animal and Plant Health Inspection Service, Plant Pest and Quarantine, Center for Plant Health Science and Technology, Otis Laboratory, Buzzards Bay, MA 02542 USA; 25 Graduate Institute of Biomedical Electronics and Bioinformatics, National Taiwan University, Taipei, 10617 Taiwan; 26 Department of Biology, Indiana University, Blomington, IN 47405 USA; 27 Department of Microbiology, Immunology, and Biochemistry, University of Tennessee Health Science Center, Memphis, TN 38163 USA; 28 Department of Entomology, University of Kentucky, Lexington, KY 40546 USA; 29 Institute for Developmental Biology, University of Cologne, Cologne, 50674 Germany; 30 Department of Biology, Xavier University, Cincinnati, OH 45207 USA; 31 Department of Entomology, University of Illinois at Urbana-Champaign, Urbana, IL 61801 USA

**Keywords:** Chemoperception, Detoxification, Glycoside hydrolase, Horizontal gene transfer, Phytophagy, Xylophagy

## Abstract

**Background:**

Relatively little is known about the genomic basis and evolution of wood-feeding in beetles. We undertook genome sequencing and annotation, gene expression assays, studies of plant cell wall degrading enzymes, and other functional and comparative studies of the Asian longhorned beetle, *Anoplophora glabripennis*, a globally significant invasive species capable of inflicting severe feeding damage on many important tree species. Complementary studies of genes encoding enzymes involved in digestion of woody plant tissues or detoxification of plant allelochemicals were undertaken with the genomes of 14 additional insects, including the newly sequenced emerald ash borer and bull-headed dung beetle.

**Results:**

The Asian longhorned beetle genome encodes a uniquely diverse arsenal of enzymes that can degrade the main polysaccharide networks in plant cell walls, detoxify plant allelochemicals, and otherwise facilitate feeding on woody plants. It has the metabolic plasticity needed to feed on diverse plant species, contributing to its highly invasive nature. Large expansions of chemosensory genes involved in the reception of pheromones and plant kairomones are consistent with the complexity of chemical cues it uses to find host plants and mates.

**Conclusions:**

Amplification and functional divergence of genes associated with specialized feeding on plants, including genes originally obtained via horizontal gene transfer from fungi and bacteria, contributed to the addition, expansion, and enhancement of the metabolic repertoire of the Asian longhorned beetle, certain other phytophagous beetles, and to a lesser degree, other phytophagous insects. Our results thus begin to establish a genomic basis for the evolutionary success of beetles on plants.

**Electronic supplementary material:**

The online version of this article (doi:10.1186/s13059-016-1088-8) contains supplementary material, which is available to authorized users.

## Background

**Fig. 1:**
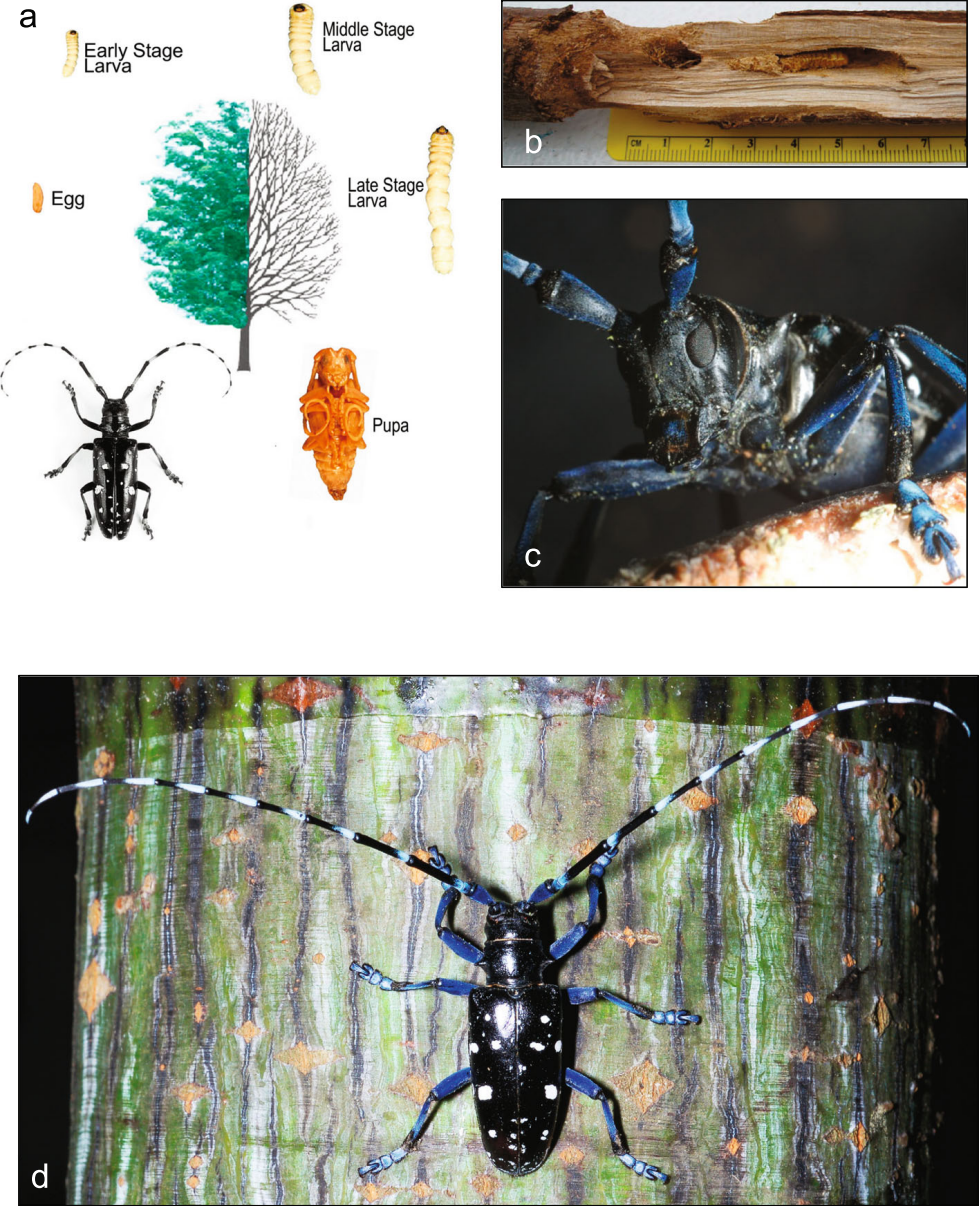
*A. glabripennis*, the Asian longhorned beetle, is a high profile invasive pest species capable of inflicting severe damage on its hosts, which include many important orchard, ornamental, and forest tree species. **a** Life cycle (adapted from Michael Bohne, used with permission; image of adult female courtesy of Barbara Strnadova, used with permission). **b** Wood dissected to expose feeding *A. glabripennis* larva (image courtesy of Kelli Hoover, used with permission). **c**, **d** Adult *A. glabripennis* (images courtesy of David Lance, used with permission). Early stage larvae are specialized wood-borers, feeding in galleries under the bark of host trees (in the subcortical tissue and phloem). Larger, later stage larvae tunnel deep into the heartwood (mature xylem) of their hosts, where they continue feeding and complete development [4]. Adults are comparatively short-lived external feeders, consuming small amounts of tissue from host leaves and twigs. *A. glabripennis* is broadly polyphagous on woody angiosperms. It is native to eastern Asia but has recently become established in several countries in North America, Europe, and beyond via solid wood packing material. *A. glabripennis* is a globally significant pest whose economic impact in the US alone, if uncontrolled, has been conservatively estimated at $889 billion (adjusted for inflation, May 2016) [5]. It is capable of attacking both healthy and susceptible trees [77] and is broadly polyphagous, feeding on at least 100 species of woody angiosperms worldwide [4, 78, 79]

Beetles (order Coleoptera; >400,000 described extant species) account for more than 20 % of metazoans. The causes of this apparent “inordinate fondness” [1] are widely debated, but the evolution of specialized trophic interactions with plants—such as wood-feeding (xylophagy)—is assumed to have played an important role [2, 3]. The beetle family Cerambycidae Latreille (>35,000 species; longhorned beetles) is the most diverse radiation of wood-feeding animals on Earth. Most species complete their entire development while feeding exclusively on the tissues of woody plants. Recent work has established the Asian longhorned beetle (*Anoplophora glabripennis*) as a model for studies of the digestive physiology of wood-feeding beetles (see references cited herein). *A. glabripennis* is a globally significant invasive species, capable of inflicting severe damage on many economically important orchard, ornamental, and forest trees (>100 species) [4]. Its potential economic impact in the United States alone, if uncontrolled, has been conservatively estimated at $889 billion (adjusted for inflation, May 2016) [5]. Early stage *A. glabripennis* larvae are specialized wood-borers, feeding in galleries under bark in the subcortical tissue and phloem of both healthy and susceptible living trees (Fig. 1). Larger, later stage larvae tunnel deep into the heartwood, where they continue feeding and complete development. Adults are comparatively short-lived external feeders, consuming small amounts of tissue from host tree leaves and twigs [4].

Nitrogen, free amino acids, and protein are typically scarce in wood and access to sugars, minerals, and other key nutrients is severely impeded by lignified plant cell walls. Furthermore, woody plant tissues contain a diversity of allelochemicals that must be detoxified or sequestered when eaten [6]. Successful feeding on woody plants therefore requires specialized metabolic adaptations. The genomes of *A. glabripennis* and certain other phytophagous beetles are known to contain genes encoding plant cell wall degrading enzymes (PCWDEs) [7–9]. PCWDEs degrade cellulose, hemicellulose, or pectin (the main polysaccharide networks in plant cell walls), liberating sugars, minerals, and other nutrients from woody plant tissues. Some cerambycid PCWDEs were originally obtained via horizontal gene transfer (HGT) from fungi or bacteria, and have subsequently diversified to form multi-gene families [10]. This is in contrast to other wood feeding insects, e.g., termites and some ants and cockroaches, which have broadly similar metabolic capabilities conveyed by symbionts whose genomes contain many of the same families of genes [11]. Additionally, lignin is degraded during passage through the *A. glabripennis* gut [12], suggesting a role for enzymes secreted into the gut by the beetle, its gut microbiota, or both parties. In vitro, PCWDEs and lignin-degrading enzymes encoded by the genomes of insects and their symbionts may be important in a wide range of biotechnological processes, including the production of biofuels and food [7, 8].

We investigated the genomic basis of specialized phytophagy on woody plants by *A. glabripennis* through genome and transcriptome sequencing and annotation, comparative genomic analyses, gene expression assays, and functional genomic studies. Complementary comparative analyses involving the *A. glabripennis* genome and 14 additional insect genomes, including two additional beetles whose genomes are studied here for the first time—the emerald ash borer (*Agrilus planipennis*, family Buprestidae) and the bull-headed dung beetle (*Onthophagus taurus*, family Scarabaeidae)—were undertaken to reconstruct broader patterns in the evolution of insect (especially beetle) genes encoding enzymes involved in the digestion of woody plant tissues or detoxification of plant allelochemicals.

## Results and discussion

### General genome features

**Fig. 2:**
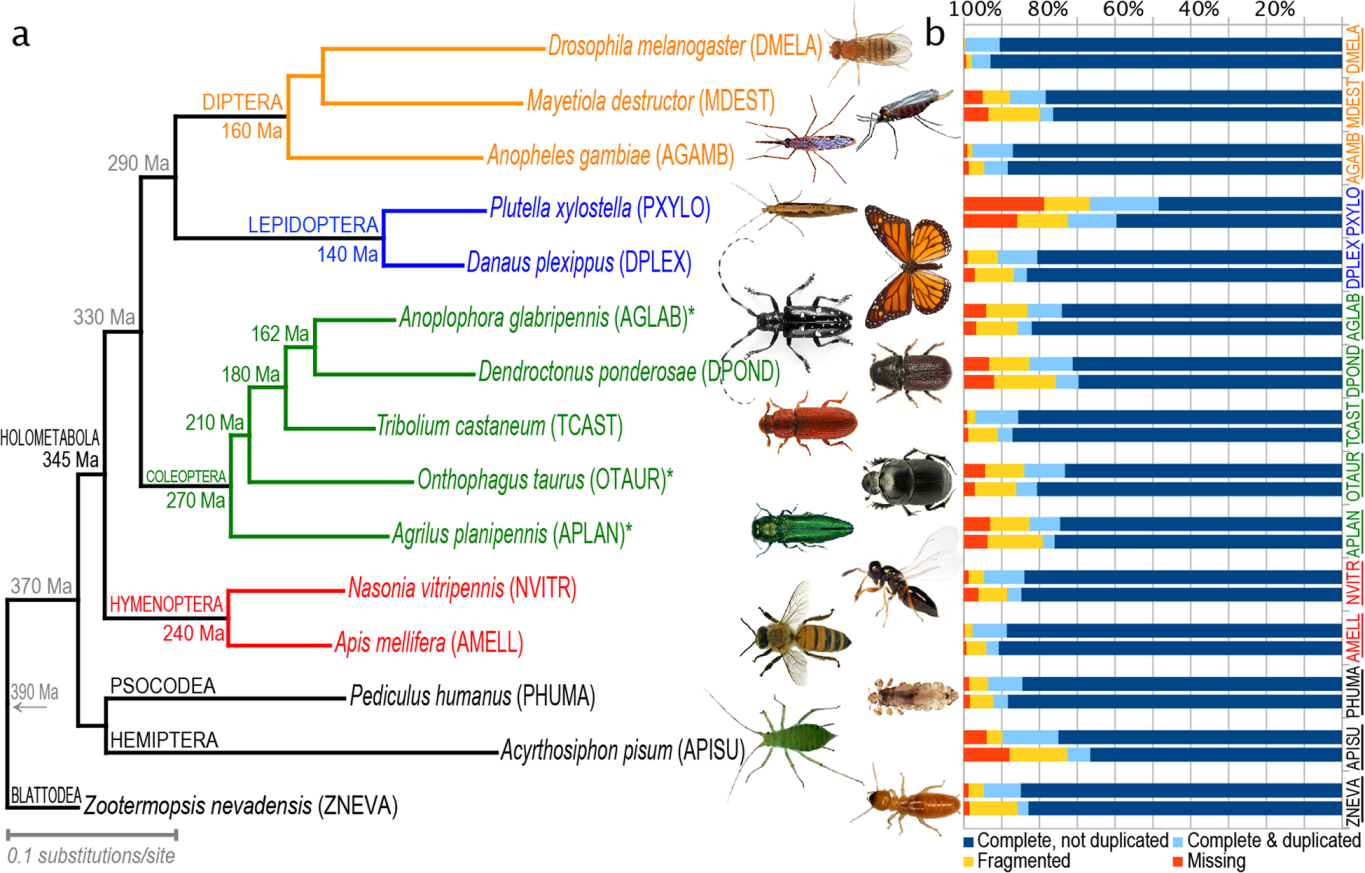
Phylogenetic relationships and estimates of completeness among the 15 insect genomes studied. **a** Maximum likelihood (ML) phylogenetic tree based on amino acid sequences from 523 orthologs. All nodes have 100 % ML bootstrap support. The tree was rooted with *Zootermopsis nevadensis. Asterisks* indicate genomes that were sequenced via i5k and are analyzed herein for the first time. Estimated divergence times are shown along branches subtending the crown group nodes they refer to and were obtained from [3] for Coleoptera and [80] for all others. **b** The completeness of both genome assemblies and official gene sets (OGSs) of each of the insects was assessed using 2675 arthropod benchmarking universal single-copy orthologs (BUSCOs). For each species, the *bottom bar* in the histogram shows the OGS-based results, whereas the *top bar* shows the genome-based results. Images courtesy of: Nicolas Gompel (*DMELA*), Scott Bauer/USDA-ARS (*MDEST*), Chris Lewis (*PXYLO*), Didier Decouens (*DPLEX*), Barbara Strnadova (*AGLAB*), Klaus Bolte (*DPOND*), Kohichiro Yoshida (*TCAST*), Rafal Celadyn (*OTAUR*), PA Dept. of CNR (*APLAN*), Elizabeth Cash (*NVITR*), Gary McClellan (*AMELL*), John and Kendra Abbott/Abbott Nature Photography (*PHUMA*), Sandy Rae (*APISU*), Don Loarie (*ZNEVA*)

We generated and assembled 134× sequence coverage of the *A. glabripennis* genome from a single female *A. glabripennis* larva, creating a draft genome reference assembly of 710 Mb with contig and scaffold N50s of 16.5 kb and 659 kb, respectively (Additional file 1: Table S3). While the *A. glabripennis* genome (female 981.42 ± 3.52 Mb, male 970.64 ± 3.69 Mb) is much larger than the four existing published beetle genomes (ranging from 163–208 Mb) [13–16], it is average-sized for the order Coleoptera (mean = 974 Mb) [17]. As in other draft genome assemblies, repetitive heterochromatin sequences could not be assembled, accounting for the differences between assembled sequence and genome sizes. The proportion of un-assembled genome in *A. glabripennis* is similar to that seen in other insect genome assemblies. Using a customized MAKER pipeline [18], 22,035 gene models were annotated. Manual curation involved 1144 gene models (Additional file 1: Table S4; Additional file 2: Table S6). The automated annotations and manual curations were merged into a non-redundant official gene set (OGS v1.2) with 22,253 protein-coding gene models and 66 pseudogenes (Additional file 2: Table S6), in contrast to the 13,526–19,222 gene models reported for existing published beetle genomes. The completeness of the *A. glabripennis* genome assembly and OGS were assessed using benchmarking sets of universal single-copy orthologs (BUSCOs) [19] and compared with 14 other insect genomes (Fig. 2). The *A. glabripennis* gene set had slightly fewer missing BUSCOs (~3.3 %) than most of the other genomes studied. Comparing BUSCO results from the *A. glabripennis* OGS to those obtained from searching the entire genome sequence, the number of missing genes was reduced, indicating that some genes were missed during the automated annotation process. Nonetheless, except for unassembled heterochromatin and other repetitive regions, the *A. glabripennis* genome is well represented and of high quality.

**Fig. 3:**
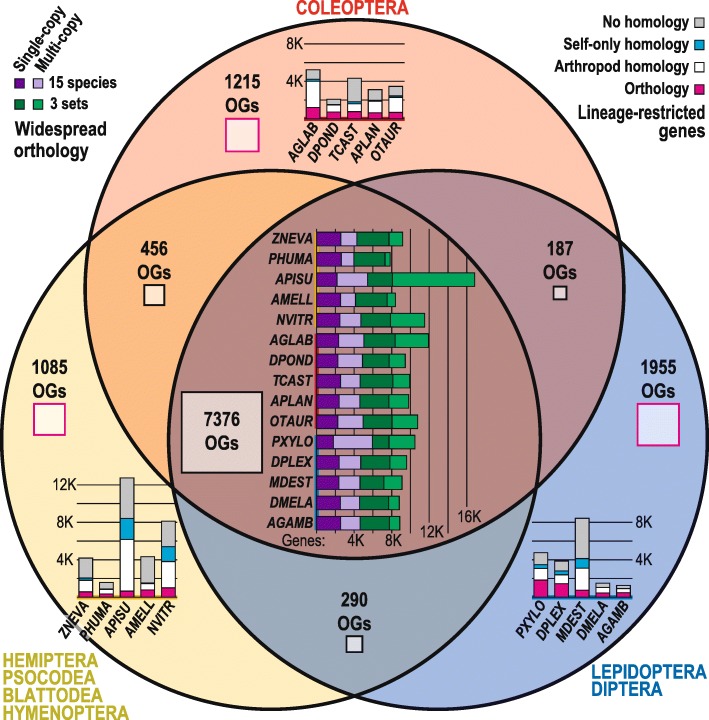
Orthology and homology assignments of *A. glabripennis* genes with those of 14 other insect species. A conserved core of about 5000 orthologs per species (5029 *A. glabripennis* genes) is maintained in orthologous groups with gene members from all 15 species, about half with a single gene (*dark purple*) and half with multiple copies (*light purple*). A variable fraction of genes is less well maintained but still widespread (*green*) with orthologs in at least two species from each of the three sets of insect species. Lineage-restricted genes include those with orthologs only within each set (*pink*), with recognizable homology to other arthropod genes (*white*) or their own genes (*cyan*), or without any significant homology (*gray*). The numbers of orthologous groups (*OGs*) are shown with *area-proportional boxes* for the set intersections and the lineage-restricted orthologs. See “Methods” for orthology classification details

OrthoDB orthology delineation [20] revealed that *A. glabripennis* has a conserved core of 5029 genes classified in orthologous groups (OGs) with orthologs from the 14 other insect genomes studied (Fig. 3). *A. glabripennis* has a high number of widespread orthologs (6880 total) in OGs that are not universal but nevertheless have representatives from each of the three sets of species studied (see “Methods”; Additional file 1: Section I.6). About half (3346) of these genes are maintained as single-copy orthologs, while the remainder (3534) appear to have duplicated. Such duplications are more frequent in *A. glabripennis* than in most of the other species but are not as extreme as in *Acyrthosiphon pisum* (pea aphid, family Aphididae; 8779). Examining OGs with orthologs from only two of the three species sets showed that the Coleoptera have maintained more ancient orthologs than the Diptera and Lepidoptera. Of the five Coleoptera genomes studied, *A. glabripennis* has the most Coleoptera-specific genes (5229), suggestive of a high degree of adaptive novelty. Of these, 1210 have identifiable orthologs in the other beetles and 2789 show no clear orthology but do have homologs in other arthropods, i.e., they are likely divergent gene copies, consistent with the large numbers of paralogs in the *A. glabripennis* genome. This leaves a small set of 1003 unique *A. glabripennis* genes with no homology to the other arthropod genes. A phylogenomic analysis of orthologs (Fig. 2) places *A. glabripennis* sister to *Dendroctonus ponderosae* (mountain pine beetle, family Curculionidae), as expected [21, 22].

Following insertion into eukaryotic genomes, bacterial HGTs will either degrade through mutational degradation or, occasionally, evolve into functional genes [23]. In addition to glycoside hydrolase (GH) family genes (discussed in section titled Plant cell wall degradation), eight HGT candidates were found from bacteria to *A. glabripennis* using a DNA-based HGT pipeline [23] and junctions between the insertion and flanking sequences were confirmed in multiple libraries (Additional file 1: Table S7). The DNA based pipeline is effective at finding HGTs with DNA sequence similarity to their bacterial source even if they are not transcriptionally active [23–25]. Four candidates were from bacteria most closely related to *Wolbachia*, and two show high (95 %) sequence similarity to *Wolbachia*, suggesting relatively recent insertion. The other two show lower similarity (70–71 %) and contain indels and are, therefore, more likely to represent older insertions undergoing degradation. Other represented potential sources include, *Calothrix*, *Clostridium*, and *Rickettsia*. None of these HGT candidates showed significant expression in RNA-seq reads for adult males, females, or larvae, although this does not rule out expression in other stages or tissue-specific expression of these candidates below detection in whole organism RNA-seq. Recent insertions have similarly been detected in other arthropod genomes using the DNA-based pipeline [24, 25]. In contrast, the GH HGTs are more ancient insertions that have evolved into functional genes [26–30] (see results from in vitro functional characterization, discussed in section titled Plant cell wall degradation). No microbial scaffolds were found in the *A. glabripennis* assembly, likely because the tissues used for sequencing (Additional file 1) are not known to be associated with microbes.

*A. glabripennis* harbors similar numbers and kinds of genes involved in growth, development, and reproduction as *Tribolium castaneum* (and other insects; Additional file 1: Section VI). Some of these gene clusters (e.g., homeodomain transcription factors) correlate in scale with its genome size (~5× larger than *T. castaneum*) but also show *A. glabripennis*-specific paralogous expansion and gene dispersal. Key components of the genetic mechanisms underlying diapause in other insects were also found in the *A. glabripennis* genome. In contrast, *A. glabripennis* appears to possess an incomplete methylation machinery, including the maintenance methyltransferase DNMT1, but lacking the de novo methyltransferase DNMT3, which was lacking from both the genome assembly and the unassembled raw reads (Additional file 1: Section VI.10). While a similar situation is found in both *T. castaneum* and *Drosophila melanogaster* (common fruit fly, family Drosophilidae), many other insects, including other beetles such as *Onthophagus taurus* [31] and *Nicrophorus vespilloides* [13] (burying beetle, family Silphidae), have retained the complete machinery. A full description of the genes studied in the *A. glabripennis* genome can be found in Additional file 1.

### Plant cell wall degradation

**Fig. 4:**
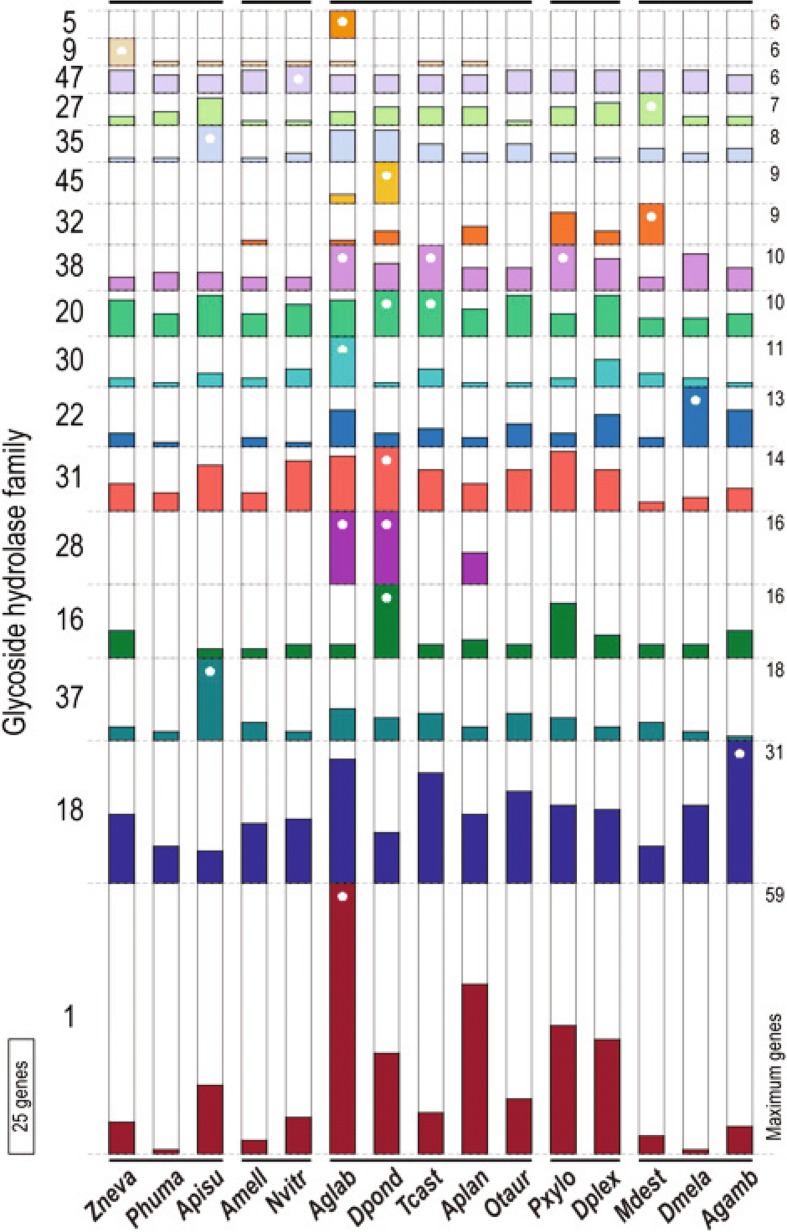
Sub-family sizes for gycoside hydrolases found in the genome sequences of 15 insect species, including *A. glabripennis*. Species with the maximum gene count for each are indicated with a *white asterisk*. Among the examined species, *A. glabripennis* showed the most genes with matches to GH domains, the majority of which were found as multi-copy orthologs. This elevated gene count was mainly due to GH family 1 (IPR001360), members of which exhibit beta-glucosidase, beta-galactosidase, 6-phospho-beta-galactosidase, 6-phospho-beta-glucosidase, lactase-phlorizin hydrolase, beta-mannosidase, and myrosinase activities. Uniquely among the examined species, six *A. glabripennis* genes matched GH family 5 (IPR001547), also known as cellulase family A, whose members exhibit endoglucanase, beta-mannanase, exo-1,3-glucanase, endo-1, 6-glucanase, xylanase, and endoglycoceramidase activities. *A. glabripennis* also had two matches to the GH family 45 (IPR000334, endoglucanase activity), also known as cellulase family K, which was also found in *D. ponderosae* (nine copies). Members of GH family 28 (IPR000743) are pectinases that exhibit polygalacturonase and rhamnogalacturonase activities and had matches to 16 genes in *A. glabripennis* (18 were identified by manual annotation; 19 were reported in [8]), 16 in *D. ponderosae* and 7 in *A. planipennis* (50 were manually annotated)

We manually annotated 86 GH family genes (Fig. 4 and Table 1; Additional file 1: Figure S18 and Tables S9 and S17) in the *A. glabripennis* genome, more than are known from any other insect. These include a large expansion of 57 GH1 genes, which putatively exhibit (amongst others) β-glucosidase and β-galactosidase activities. Only 15 GH1 genes are known from *T. castaneum* [15], and only 19 from *D. ponderosae* [14]. We manually annotated 11 putative endo- and exoglucanases (cellulases), members of GH9, subfamily 2 of GH5, GH45, and GH48, and 18 GH28 genes encoding putative pectin-degrading polygalacturonases. Previous work has shown that a number of GH family genes have been acquired from microbes by HGT (e.g., [24–30]; Table 1), and Fig. 4 shows the distribution of these and endogenous GHs in the 15 arthropod genomes studied herein. The genome of *A. glabripennis* was unique among the 15 species studied in containing matches to GH5 (IPR001547; Fig. 4), whose members exhibit predominantly endo- and/or exo-glucanase, mannanase, and xylanase activities.

**Table 1:** Plant cell wall degrading enzymes identified in the *A. glabripennis* genome assembly by manual annotation

Gene family	Putative function	Genes total	Pseudogenes
*Cellulose/hemicellulose degradation*			
GH9	Endo-β-1,4-glucanase	1	0
GH45	Endo-β-1,4-glucanase	2	0
GH5 subfamily 2	Endo/exo-β-1,4-glucanase	6	0
GH48	Reducing end-acting cellobiohydrolase	2	0
GH1	β-Glucosidase (myrosinase, cyanogenic β-glucosidase)	57	3
*Pectin degradation*			
GH28	Polygalacturonase	18	0

**Fig. 5:**
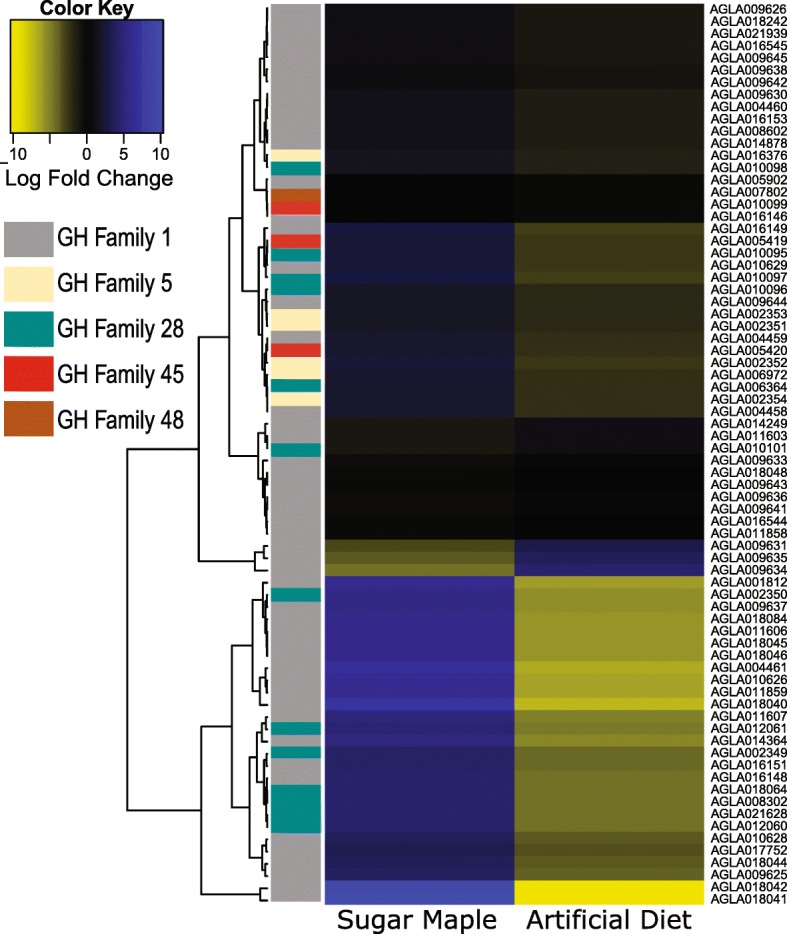
Heatmap showing expression levels from *A. glabripennis* gycoside hydrolase genes with putative involvement in plant cell wall degradation. Logfold changes in expression levels in genes collected from *A. glabripennis* larvae feeding in the wood of living sugar maple trees are shown versus those from larvae feeding on a nutrient-rich artificial diet. While the expression levels of GH genes were variable, several were significantly upregulated in larvae feeding in the wood of living sugar maple

Genes encoding GH9 cellulases have an ancient origin in animals [26]. The other beetle-derived GH families involved in plant cell wall digestion have a more recent origin and were putatively obtained via HGT from bacteria or fungi. GH5 subfamily 2 genes were likely acquired via HGT from Bacteroidetes [27]. GH45 genes were likely acquired by the last common ancestor (LCA) of the Phytophaga (the sister beetle superfamilies Chrysomeloidea and Curculionoidea) via HGT from a fungus [28, 29]. Amino acid sequences of beetle GH48 cellulases are similar to bacterial cellobiosidases, but their function(s) remain unclear; they may have evolved to scavenge nitrogen by degrading chitin in the gut or diet [81], e.g., from host plant tissues containing fungi, or from fungi resident in the gut (e.g., yeasts, *Fusarium solani*) which are thought to concentrate nitrogen and synthesize essential amino acids [9, 30, 35]. GH48s are constitutively highly expressed in *A. glabripennis* larvae (Fig. 5), and their induction in larvae feeding in a nutrient-poor environment (reported herein) is consistent with a putative role in nutrient scavenging. They were most likely acquired by the LCA of the Phytophaga via HGT from a bacterial donor [28, 30]. GH28 genes were likely acquired by the LCA of the Phytophaga via HGT from an ascomycete fungus and subsequently expanded and diversified, but lost in the longhorned beetle subfamily Lamiinae (which includes *A. glabripennis*). After this loss, a GH28 gene was apparently re-acquired by Lamiinae via HGT from a fungal donor [10]

We investigated diet-dependent regulation of GH family genes via an RNA-seq-based differential expression analysis of *A. glabripennis* larvae feeding on an artificial diet versus the wood of living sugar maple trees, a preferred host. All GH5 and GH45 cellulases were expressed at least twofold higher in larvae feeding in sugar maple (Fig. 5) and have likely roles in converting cellulose into more easily digestible cello-oligosaccharides. Over 30 GH1 genes were most highly expressed in larvae feeding in sugar maple. Many of these genes are putative β-glucosidases and likely convert cellobiose and other oligosaccharides released from the plant cell wall into monosaccharides. GH1 enzymes can have broad catalytic and substrate specificities, so GH1 genes induced in larvae feeding in sugar maple could also function as β-xylosidases, β-glucuronidases, β-galactosidases, β-mannosidases, or exo-β-1,4-glucanases, serving to hydrolyze substrates released from the hemicellulose matrix. Additionally, many β-glucosidases also have known roles in detoxification [32, 33] (discussed in section titled Detoxification of plant allelochemicals). Twelve GH28 genes showed elevated expression in larvae feeding in sugar maple, and their homologs are known to function as polygalacturonases in relatives of *A. glabripennis* [7, 10]. Thus, pectinous components of plant primary cell walls may serve as a significant source of sugars for early instar *A. glabripennis* larvae. GH35 genes were also induced in *A. glabripennis* larvae feeding in sugar maple. These had highest scoring BLAST alignments to β-galactosidase and could play roles in processing β-1,4-linked galactose oligomers released from the plant cell wall matrix. GH30 genes were also highly induced in larvae feeding in sugar maple. While some of these were expressed in both larvae and adults, two were expressed exclusively in larvae (AGLA015835 and AGLA015837) and may be important for digesting components of plant secondary cell walls. Consistent with this hypothesis, these two GH30 genes were strongly upregulated in insects feeding in sugar maple compared to on an artificial diet with log fold change expression values of 6.7 (false discovery rate (FDR) = 1.14e-05) and 6.0 (FDR = 1.83e-07). Additionally, three other GH30 genes were more highly expressed in larvae feeding in sugar maple, including AGLA015834 (logFC = 5.0; FDR = 2.96e-11), AGLA015831 (logFC = 1.96; FDR = 0.029), and AGLA001694 (logFC = 1.80; FDR = 0.05). Although the expression patterns of these genes seem consistent with a role in breaking down secondary cell wall polysaccharides in the larval stage, the precise reactions catalyzed by these gene products could not be predicted based on electronic annotations.

To determine substrate specificity and the contribution of enzymes encoded by GH family genes to the metabolism of plant cell wall polysaccharides, 15 of the 18 known *A. glabripennis* GH28 genes (putative polygalacturonases) were functionally characterized in vitro. Heterologous expression succeeded for all but GH28-4 (AGLA010098; Additional file 1: Figure S5). Most GH28 proteins were active against at least one homogalacturonan polymer in plate assays. A group of phylogenetically related proteins, GH28-1 (AGLA010095), -2 (AGLA010096), -3 (AGLA010097), and -5 (AGLA010099), all located in tandem on one genomic scaffold, showed no activity against homogalacturonan polymers (Additional file 1: Figures S5, S6b, S7). However, they did exhibit exopolygalacturonase activity, similar to a previously characterized GH28 from a near relative of *A. glabripennis* [7] (Additional file 1: Figure S6c). GH28-11 (AGLA002350), the only polygalacturonase expressed in both *A. glabripennis* larvae and adults [7], and GH28-17 (AGLA025090) both functioned as endopolygalacturonases; however, accumulation of galacturonic acid monomers was also observed for GH28-11, indicating that it could also function as an exopolygalacturonase (Additional file 1: Figure S6c). Overall, the repertoire of GH28 enzymes encoded by the *A. glabripennis* genome contains both endo- and exo-polygalacturonases and is able to act on substrates with varying degrees of methylation. These enzymes are highly complementary, allowing *A. glabripennis* to efficiently decompose pectinous homogalacturonan polymers present in the primary cell walls of living woody plant tissues.

Six GH5 genes, two GH45 genes, and one GH9 gene were also functionally characterized in vitro. GH5-1 (AGLA002353) functioned as an endo-β-1,4-xylanase (EC 3.2.1.8), GH5-2 (AGLA002352), GH5-5 (AGLA006972), GH45-1 (AGLA005419), and GH45-2 (AGLA005420) functioned as endo-β-1,4-glucanases (EC 3.2.1.4), and GH5-2 showed endo-β-1,4-xyloglucanase activity (EC 3.2.1.151) (Additional file 1: Figures S8b and S9). GH5-2 also hydrolyzed carboxymethylcellulose (CMC), indicating that enzymes encoded by this gene possess the ability to endohydrolyse the 1,4-β-D-glucosidic linkages in both CMC and xyloglucan and may function to degrade both cellulose and components of hemicellulose in vivo. GH5-3 (AGLA002354), GH5-4 (AGLA002351), GH5-6 (AGLA016376), and GH9 (AGLA010313) did not harbor any enzymatic activity against the substrates tested, indicating that they are not endo-acting enzymes. To investigate how GH5 enzymes degrade their substrates, the products were subsequently analyzed by thin layer chromatography (TLC) (see “Methods”; Additional file 1: Figure S8c), validating the roles of GH5-1 as a xylanase, GH5-2 as a dual-acting xyloglucanase/endoglucanase, and GH5-5 as an endoglucanase. Furthermore, although no zone of clearing was observed for GH5-6 in an agarose diffusion assay, accumulations of glucose and cellobiose were observed via TLC after incubation with CMC, suggesting that it functions as an exo-β-1,4-glucanase (Additional file 1: Figure S8c). None of these enzymes had the ability to degrade crystalline cellulose substrates. However, Geib et al. [34] observed activity against Avicel in enzyme extracts prepared from larval *A. glabripennis* guts. This suggests that (a) GH5 and GH45 cellulases act synergistically in vivo to degrade these substrates, (b) other *A. glabripennis*-encoded enzymes besides those characterized in this study possess the ability to degrade Avicel, or (c) that enzymes produced by the gut microbial community are responsible for the aforementioned previously observed activity. Notably, the cellulases encoded by numerous members of the *A. glabripennis* gut microbial community possess carbohydrate-binding domains, which could enhance the efficiency of these enzymes against crystalline substrates by allowing them to bind and degrade their substrates in a processive manner [30, 35]. Thus, the *A. glabripennis* genome encodes at least three families of cellulases and hemicellulases (subfamily 2 of GH5, GH9, and GH45) and one family of polygalacturonases (GH28) that provide it with an arsenal of enzymes capable of degrading the main polysaccharides of the cellulose and hemicellulose networks in both primary and secondary plant cell walls.

GH28, GH45, and subfamily 2 of GH5 were collectively detected only in the three phytophagous beetle genomes studied (*A. glabripennis*, *A. planipennis* and *D. ponderosae*) (Fig. 4; Additional file 1: Figure S18) and were lacking from the 12 other insect genomes. Specifically, GH28 was detected in *A. glabripennis*, *A. planipennis*, and *D. ponderosae*, GH45 was detected only in *A. glabripennis* and *D. ponderosae* (sister taxa in our phylogeny, spanning the basal split in the clade Phytophaga [36] (Fig. 2), and subfamily 2 of GH5 was detected exclusively in *A. glabripennis*. Subfamily 2 of GH5 genes have been found in at least one other cerambycid [7] and may be unique to superfamily Chrysomeloidea (leaf beetles, cerambycids, and their relatives). *A. glabripennis*, *A. planipennis*, and *D. ponderosae* are all specialized phytophages belonging to species-rich taxonomic groups of beetles that feed on the subcortical tissues of woody plants and interact with specialized suites of gut microbes. Interestingly, the genomes of the wood-feeding termites *Macrotermes* and *Zootermopsis* lack all three of the aforementioned gene families. However, these genes are present in the genomes of their gut symbionts. This is in contrast to the phytophagous beetles we studied, whose ancestors obtained these genes (in their genomes) via HGT from bacteria and fungi [8, 14] (Additional file 1: Figures S5 and S9). These genes subsequently diversified in beetle genomes to form multi-gene families [10]. Notably, the GH28 family genes we annotated in *A. planipennis* were apparently acquired independently (via HGT from an ascomycete fungus donor) from those in *A. glabripennis* and *D. ponderosae*. Independently acquired GH28 genes are also known from phytophagous Hemiptera in the species-rich family Miridae [37].

GH1 family genes can encode enzymes having both digestive and non-digestive functions. Twenty-three *A. glabripennis* GH1 sequences had ~44 % identity to sequences annotated as myrosinases (MYR) [31] in the *T. castaneum* genome [38]. One sequence closely matches known myrosinase active site motifs. For some insects, including flea beetles, myrosinases are known to synergize alarm or aggregation pheromones [39, 40]. Non-Brassicaceae, woody plant sources of glucosinolytes, which are the substrates detoxified by myrosinase, are present in the *A. glabripennis* native range [41]. An additional possibility is that one or more of these *A. glabripennis* sequences is a cyanogenic β-glycosidase [33]. Toxic cyanogenic glycosides are used by some plants (including known hosts of *A. glabripennis*) as a defense against insect-feeding, analogous to the myrosinase system. Interestingly, five *A. glabripennis* GH1 sequences are intermediate in similarity to known myrosinases and a known cyanogenic β-glycosidase (Additional file 1: Figure S16).

Microbes in the gut of *A. glabripennis* are known to have definitive roles in nutrient biosynthesis and nutrient recycling, helping the beetle to thrive under nutrient-poor conditions [35, 42, 43]. *A. glabripennis* microbes encode an arsenal of laccases, peroxidases, aldo-keto reductases, dyp-type peroxidases [30], and at least one lignin peroxidase, which is encoded by a fungal symbiont belonging to the *F. solani* species complex [44]. Several of the aforementioned genes are actively expressed in the *A. glabripennis* larval midgut [35]. While these enzymes have not been functionally characterized in vitro, they may facilitate lignin degradation in the *A. glabripennis* gut. The *A. glabripennis* genome itself may also encode genes that facilitate lignin degradation. *A. glabripennis* encodes eight genes with hemocyanin domains, three of which are significantly more highly expressed in larvae feeding in sugar maple, including the gene models AGLA002479 (2.1 log-fold upregulation), AGLA002478 (2.5 log-fold upregulation), and AGLA001233 (3.4 log-fold upregulation). All three genes were originally thought to function as storage hexamer proteins. However, the ability of at least one termite-derived hemocyanin highly expressed in salivary glands to oxidize model lignin compounds and other aromatic compounds in vitro [45], and the high expression levels of these three genes in multiple organisms that feed in wood [46], could signal that they work synergystically with gut microbes in *A. glabripennis* to facilitate oxidative degradation of prominent linkages in the lignin polymer and/or other biopolymers in vivo.

### Detoxification of plant allelochemicals

To gain further insights into the genomic basis of the broad host range of *A. glabripennis* (>100 known host tree species) and its concomitant invasiveness, we studied gene families hypothesized to encode key enzymes involved in the detoxification of plant allelochemicals (Additional file 1: Tables S17–S26 and Figures S18–S22). Cytochrome P450s (CYP450; Additional file 1: Figure S21 and Tables S20 and S25) encode the most prevalent detoxification enzymes in insects and participate in many other important physiological processes. A total of 106 genes and 19 pseudogenes predicted to encode CYP450s were manually annotated in the *A. glabripennis* genome; 137 genes and 6 pseudogenes were detected by matches to InterPro domains, the third highest number in our comparative genomic study after the beetles *T. castaneum* and *O. taurus*. Examining the CYP450 sub-families showed that *A. glabripennis* had five times as many group II matches (18 genes; including CYP4 and CYP6) than the average across the other insect species studied. CYP6 enzymes metabolize a wide range of toxic compounds and are known to clear odorants in insect antennae [47]. CYP4 enzymes are involved in cuticular hydrocarbon biosynthesis and have been implicated in insecticide resistance [48]. Supporting their roles in detoxification, 25 CYP450 genes were induced in the guts of *A. glabripennis* larvae feeding in sugar maple, including many genes in *A. glabripennis*-specific clades (Additional file 1: Figure S10). Only two of the genes that were induced (CYP18A1, CYP314A1) occurred in orthologous pairs with *T. castaneum* genes. Therefore, while the many CYP450 ortholog pairs between *T. castaneum* and *A. glabripennis* presumably carry out functions conserved over millions of years of evolution, expansion of several CYP families and the evolution of *A. glabripennis*-specific CYP clades relative to *T. castaneum* suggests that these genes have evolved and diversified in *A. glabripennis* as a mechanism to overcome host plant defenses.

**Fig. 6:**
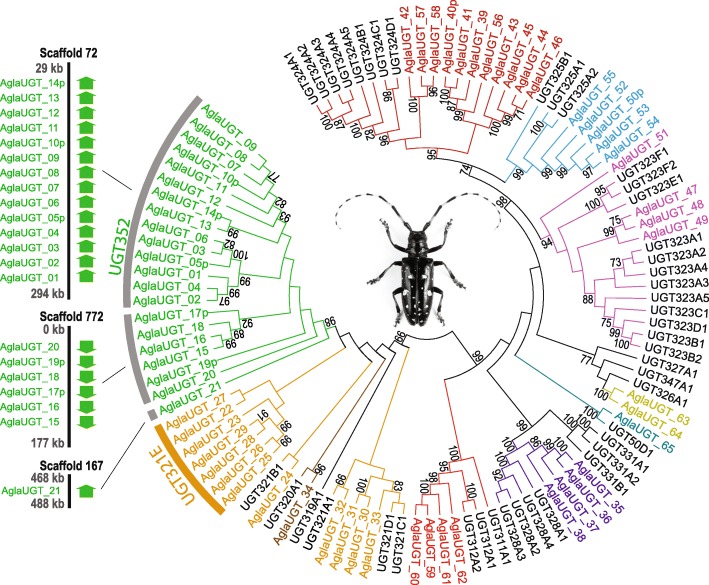
Phylogenetic tree showing *A. glabripennis* (*color*) and *T. castaneum* (*black*) UDP-glycosyltransferases (*UGTs*), reconstructed from amino acid sequences using ML inference (MLBS values <70 not shown). Each gene belonging to UGT352, UGT321, and UGT328 consists of four exons, with the long first exon (ca. 810 amino acids) followed by three short exons. Each member of UGT323, UGT324, and UGT325 is composed of four exons with the short first exon (ca. 200 amino acids) and the long second exon (ca. 800 amino acids) followed by two short exons. UGT312 and UGT353 (AglaUGT_63 and _64) consistently contain genes with five exons. Scaffold 72 is shown to illustrate the tandem arrangement typical of *A. glabripennis* UGTs. Photo of *A. glabripennis* courtesy of Barbara Strnadova, used with permission

UDP-glycosyltransferases (UGTs) assist with the detoxification and elimination of xenobiotics (foreign substances such as those produced by parasites) and in the regulation of endobiotics (substances produced, e.g., in response to the presence of parasites). We manually annotated 65 putative UGTs, including seven pseudogenes, in the *A. glabripennis* genome (Fig. 6; Additional file 1: Figures S11, S12, and S22 and Tables S21 and S26; Additional file 2: Table S16). Only two taxa have so far been reported to harbor a greater number of UGT genes, *Locusta migratoria* (the migratory locust, family Acrididae; 68 UGTs) [49] and the aphid *A. pisum* (72 UGTs; reported herein via matches to InterPro domains; 58 UGT genes were reported for *A. pisum* by Ahn et al. [50]). The expansion of UGTs in *A. glabripennis* may be related to its ability to feed on a broad range of healthy host plants, a feature shared with *L. migratoria*. Approximately 92 % of *A. glabripennis* UGTs are arranged in a tandem manner and 50 of them were concentrated in just seven clusters. Most UGTs thus appear to have diversified by tandem gene duplication, resulting in increased substrate range of host secondary metabolites by altering the N-terminal substrate binding domain of the enzyme. The largest UGT family observed in *A. glabripennis*, UGT352, is unique to this species and consists of 21 genes. Fourteen UGT352 genes were positioned in the same orientation in a cluster on one scaffold (Fig. 6). An *A. glabripennis*-specific expansion of seven genes was found in the UGT321 gene family. These expansions may enable *A. glabripennis* to adapt to a wide range of host plant defenses. Consistent with this hypothesis, four UGTs were strongly upregulated in *A. glabripennis* larvae feeding in sugar maple, including two UGT321 genes, and one UGT352. Although only a portion of the potential detoxification genes harbored in the *A. glabripennis* genome were induced while feeding in sugar maple—just one of the many host plants of *A. glabripennis*—the existence of a diverse metabolic repertoire likely helps *A. glabripennis* feed on different host species that produce different defensive compounds.

In addition, the *A. glabripennis* genome was found to contain more putative esterases than any of the other insect genomes studied (Additional file 1: Figure S20 and Tables S19 and S24). This is due mainly to a large expansion of type-B carboxylesterases (COesterases; IPR002018), most of which are paralogs. COesterases are important for the metabolism of xenobiotics and for degrading ester bonds linking lignin to hemicellulose in plant secondary cell walls. We identified 107 COesterases in the *A. glabripennis* genome (Additional file 1: Figure S14), more than double the average in the other species studied. Most COesterases occur in large clusters; only 28 (25 %) occur as singletons. Two large clades of COesterases, one containing 17 genes and the other 13 genes, were unique to *A. glabripennis. A. glabripennis* also had the most genes (eight total) matching the thioesterase domain (IPR001031). COesterases were among the most highly induced genes in *A. glabripennis* larvae feeding in sugar maple and most of the highly induced COesterases belonged to *A. glabripennis*-specific clades and formed tandem repeats in the genome, potentially signifying novel functions related to digestion of woody plant tissues or detoxification of plant allelochemicals.

Digestive proteinases may play key roles in scavenging nitrogen from plant cell wall proteins or midgut endosymbionts and may help phytophagous insects cope with proteinase inhibitors produced by plants [51]. *A. glabripennis*-specific expansions of several proteinase OGs were observed in comparison to *T. castaneum* and *D. ponderosae*. The largest were OGs EOG8V724X and EOG8V19NQ, comprising tandem arrays of eight and seven trypsin genes, respectively. Both OGs contain genes predicted to encode secreted serine proteinases. Most proteinase genes were unique to each of the five beetle species studied, suggesting that their evolution occurred largely after speciation and may be correlated with exposure to different digestive enzyme inhibitors and with feeding on different diets. These gene families appear to be highly dynamic and may largely shape the digestive physiology of phytophagous insects.

### Sensory biology

*A. glabripennis* adults use a complex set of chemical and visual cues for host plant and mate finding. We compared the members of four gene families involved in chemoperception (olfaction and gustation) and vision in *A. glabripennis* with those from *T. castaneum* and *D. melanogaster*. We manually annotated 52 odorant binding protein (OBP) genes in the *A. glabripennis* genome (Additional file 1: Figure S23). Most OBPs comprise a large expansion of the minus-C subfamily, and the remaining genes were placed singly or in small radiations that exhibit the classic 6-cysteine motif. One OBP (AglaOBP51) was identified as a member of the plus-C group, the same as in *T. castaneum* and *D. ponderosae* [52], suggesting that the tendency toward minus-C OBPs originated at least with the beetle infraorder Cucujiformia (~190 Ma) [3]. *A. glabripennis* has 131 odorant receptor (OR) genes in addition to the highly conserved OR co-receptor Orco (Additional file 1: Figure S24). These include representatives of all seven subfamilies of beetle ORs except group 6 and follow the pattern of frequent paralogous radiations typical of insect chemoreceptors. Two new lineages of ORs were identified in *A. glabripennis* and placed as outgroups to OR groups 4, 5, and 6 in *T. castaneum* (Or106-115/126-132 and Or101-103). The function of beetle ORs remains mostly unknown, and receptors have only been characterized from *Megacyllene caryae* (hickory borer, family Cerambycidae; McarOr3). AglaOr29 is notably sister to McarOr3, which is sensitive to 2-methylbutan-1-ol, a pheromone component of *Megacyllene* [53].

*A. glabripennis* has an extensive suite of 234 gustatory receptors (GRs), including three conserved candidate CO_2_ receptors (Gr1–3), ten candidate sugar receptors (Gr4–13), and three candidate fructose receptors related to DmGr43a (Gr14–16). The remaining 127 GRs encode 218 receptors through alternative splicing and presumably belong to the general category of candidate bitter taste receptors, although some likely are also involved in contact pheromone perception [54], a component of *A. glabripennis* mate-finding behavior [55]. *A. glabripennis* has 72 ionotropic receptors (IRs), including orthologs of the conserved co-receptors IR8a and 25a and of IR21a, 40a, 41a, 68a, 76b, 93a, and 100a. The IR75 lineage consists of eight genes compared with six in *T. castaneum* and seven in *D. melanogaster*. These are all candidate ORs, while the candidate GRs, represented by the DmIr20a clade of 40 genes [56], consist of 55 genes, compared to 53 in *T. castaneum*, although these two beetles exhibit differential species-specific expansion of gene lineages within this large grouping. Like *T. castaneum* [15, 57], *A. glabripennis* has large OR and GR repertoires compared with *D. melanogaster*, and indeed most other insects except ants, but their OBP and IR repertoires are more comparable with that of *D. melanogaster* and similar to many other insects (Additional file 1: Table S27). The optical sensitivity of *A. glabripennis* appears to be similar to that of *T. castaneum* [58]. *A. glabripennis* has a single long-wavelength-sensitive opsin and a single UV-sensitive opsin. *A. glabripennis* differs from *T. castaneum*, however, in having the Rh7 opsin, whose function is unknown, and in lacking the c-opsin found in most other insects and other arthropods, which is presumed to have a non-visual function [59].

## Conclusions

*A. glabripennis* possesses a remarkably robust enzymatic repertoire capable of digesting most of the polysaccharides it encounters while feeding on woody host plants (cellulose, xyloglucan, xylan, and pectin). Furthermore, diverse suites of detoxification genes and several classes of digestive proteinases provide *A. glabripennis* with the metabolic plasticity needed to overcome the challenges of feeding on different host trees, each with a distinct profile of defensive compounds. Many of the paralogs in gene families encoding enzymes typically involved in plant cell wall degradation (PCWDEs) and detoxification occur in large clusters in the *A. glabripennis* genome and appear to have diversified by tandem gene duplication. Large expansions of genes encoding CYP450s, UGTs, COesterases (these three together are sometimes called the defensome; e.g., [60]) and GH1s in the *A. glabripennis* genome are particularly notable, as they are among the largest such repertoires of detoxification genes known in insects. Genes encoding PCWDEs are also uniquely expanded in number in the *A. glabripennis* genome. The *A. glabripennis* genome encodes genes from a remarkable three families of putative cellulases (GH5 subfamily 2, GH9, and GH45), and one of these, GH5 subfamily 2, evolved in such a way that it provides the beetle with an arsenal of enzymes possessing the ability to degrade the main polysaccharides of the cellulose and hemicellulose (xylan and xyloglucan) networks in both primary and secondary plant cell walls. *A. glabripennis* also has the ability to degrade lignin, either through the activities of its gut microbial fauna and/or by way of enzymes encoded in its genome. Our results are notable in including not only an enumeration of genes potentially involved in plant cell wall degradation and detoxification (thus facilitating specialized phytophagy on woody plants and a wide host range), but also results from experimental assessments of gene expression and enzyme activities.

Acquisition of new genes (here, GH5, GH28, and GH45 family genes) via HGT from bacteria and fungi followed by gene copy number amplification and functional divergence contributed to the addition, expansion, and enhancement of the metabolic repertoire of *A. glabripennis*, certain other beetles, and, to a lesser degree, other phytophagous insects. Our results thus further establish a genomic basis for the invasiveness and broad host plant range of *A. glabripennis* and reveal genomic innovations potentially underlying the evolutionary success of insects—especially beetles—on plants.

## Methods

### Genome size and DNA and RNA for sequencing

The genome size of five male and five female adult *A. glabripennis* collected from the former Chicago, IL, USA infestation were estimated via flow cytometry. The *A. glabripennis* specimens sequenced for this project were obtained from a USDA-APHIS colony stocked with the descendants of beetles collected from current and former infestations in IL, NY, and MA, except when noted otherwise in the supplement (Additional file 1: Table S1). The *A. glabripennis* genome was sequenced from DNA that was extracted from a single late instar female larva (G Biosciences, Omniprep kit), whose sex was determined after sequencing (Additional file 1: Figure S3).

### Genome sequencing and assembly

An enhanced Illumina-ALLPATHS-LG [61] sequencing and assembly strategy was employed. We sequenced four libraries of nominal insert sizes 180 bp, 500 bp, 3 kb, and 8 kb at genome coverages of 59.7×, 45.8×, 58.7×, and 20.5×, respectively. Sequencing was performed on Illumina HiSeq2000s generating 100-bp paired-end reads. Reads were assembled using ALLPATHS-LG (v35218) and further scaffolded and gap-filled using in-house tools Atlas-Link (v.1.0) and Atlas gap-fill (v.2.2) (https://www.hgsc.bcm.edu/software/). Data for the *A. glabripennis* genome have been deposited in the GenBank/EMBL/DDBJ Bioproject database under the accession code PRJNA163973 (Additional file 1: Table S3). Raw genomic sequence data have been deposited in the GenBank/EMBL/DDBJ Sequence Read Archive under the accession codes SRX326764, SRX326768, SRX326767, SRX326766, and SRX326765. The genome assembly has been deposited to GenBank under the accession GCA_000390285.1. RNA-seq datasets used in gene prediction have been deposited to the GenBank/EMBL/DDBJ sequence read archive under the accession codes SRX873913 and SRX873912.

### Automated annotation

The *A. glabripennis* genome assembly was subjected to automatic gene annotation using a MAKER 2.0 [18, 24, 62] annotation pipeline tuned for arthropods. Both protein and RNA-seq evidence from extant arthropod gene sets were used to guide gene models. The genome assembly was first subjected to de novo repeat prediction and Core Eukaryotic Genes Mapping Approach (CEGMA) analysis [63] to generate gene models for initial training of the ab initio gene predictors. Three rounds of training of the Augustus [64] and SNAP [65] gene predictors within MAKER were used to bootstrap to a high quality training set. RNA-seq data from *A. glabripennis* adult males and females was used to identify exon–intron boundaries. Finally, the pipeline used a nine-way homology prediction with human, *D. melanogaster*, and *Caenorhabditis elegans*, and InterPro Scan5 to allocate gene names. The automated gene set is available from the BCM-HGSC website (https://www.hgsc.bcm.edu/asian-long-horned-beetle-genome-project) and at the National Agricultural Library (https://i5k.nal.usda.gov).

### Community curation

The *A. glabripennis* genome was curated to improve the structural and functional annotations of genes and gene families of interest using the Web Apollo manual curation tool [66] (Additional file 1: Table S4; Additional file 2: Tables S5 and S6). Web Apollo is an interactive, web-based manual curation tool that visualizes user-generated annotation changes in real time, allowing remote collaboration on annotations. The *A. glabripennis* genome coordinator (D. McKenna, University of Memphis) organized a group of experts to manually curate genes or gene families of interest in Web Apollo. Web Apollo (https://apollo.nal.usda.gov/anogla/jbrowse/) tracked all evidence used for the MAKER gene predictions, as well as an additional RNA-seq dataset that was not used in the generation of the MAKER gene predictions. The manually curated models were inspected for quality, including overlapping models, internal stop codons within the coding sequence, gff3 formatting errors, and mixed transcript types within gene models. The quality-corrected models were then merged with the MAKER-predicted gene set to generate an official gene set (OGS), followed by post-processing to ensure curation information was transferred adequately. A full list of conditions for mRNA, gene, exon, and coding sequence is provided in Additional file 1: Table S5. All functional information was included in the OGS. Information on the *A. glabripennis* genome project is collated at the i5k Workspace [67] (https://i5k.nal.usda.gov/Anoplophora_glabripennis), and the genome, transcript, and protein sets can be searched via BLAST and browsed via the JBrowse genome browser [68] (https://apollo.nal.usda.gov/anogla/jbrowse). All manually curated genes and transcripts and their curation actions are provided in Additional file 2: Table S6. Additional details on annotation methods are provided in the Additional file 1.

### Assessing orthology and the quality of genome assembly and annotation

Orthology data from OrthoDB v8 [20] with a total of 87 arthropod species were analyzed to identify orthology and homology assignments of *A. glabripennis* genes with those of other beetles and representative species from six other insect orders. The gene sets of *A. planipennis* and *O. taurus* (unpublished data, manuscript in preparation; Fig. 2) were mapped to OrthoDB v8 orthologous groups (OGs) to include them in the analysis. The selected species include several that feed on plants and were partitioned into three species sets: five Coleoptera, five Lepidoptera/Diptera, and five outgroup insects. Arthropod OGs were queried with custom Perl scripts to identify OGs with genes from all three species sets (across 15 species), just two sets (across ten species), or restricted to a single set (across five species). To be considered shared, OGs were required to contain genes from at least two species in each set. For those shared among all three sets (a total of 7376 OGs), the numbers of single-copy and multi-copy orthologs were summed across all OGs for each species. Lineage-restricted genes without orthologs were assessed for significant homology (e-value <1e − 05) to other arthropod genes from OrthoDB or for significant homology (e-value <1e − 05) to genes from their own genomes (self-only homology). The completeness of the *A. glabripennis* genome assembly and annotated offical gene set (OGS) were assessed using BUSCOs [19]. We compared the results from *A. glabripennis* to those from 14 other insect genomes (Fig. 2b; Additional file 1: Figure S1). We used the Arthropoda gene set, which consists of 2675 single-copy genes that are present in at least 90 % of Arthropoda.

### Identification of bacterial to eukaryote HGTs

HGTs were identified as described in Wheeler et al. [23]. Briefly, we used BLASTN to compare genomic scaffolds against a bacterial database containing 1097 complete bacterial genome sequences downloaded from the National Center for Biotechnology Information (NCBI). Regions with significant bacterial identity (E value <1e − 5) were then compared to a second database containing representative animal genomes (see Wheeler et al. [23] for a list of animal species) obtaining a corresponding “animal” BLASTN E value score. If the animal E value score was less than the bacterial E value score the sequence was excluded as a slowly evolving highly conserved gene. Candidates were then further annotated manually for flanking eukaryotic genes and junctions between eukaryotic and bacterial sequences in the libraries. For glycoside hydrolases, the same methods were used, but we additionally simply BLASTed the genome using sequences of known, characterized PCWDEs found in phytophagous beetles [8–10], including *Apriona japonica* [7], a close relative of *A. glabripennis*.

### Differential expression analysis of *A. glabripennis* larvae feeding on sugar maple versus artificial diet

Five pairs of adult male and female *A. glabripennis* were allowed to maturation feed on fresh twigs collected from Norway maples (*Acer platanoides*, family Aceraceae) for two weeks. After this period, the beetles were allowed to mate and oviposit into potted sugar maple trees (*Acer saccharum*) maintained in a USDA-approved quarantine greenhouse for two weeks. The trees were harvested approximately 60 days after the eggs hatched and four third-instar larvae were collected. Four third-instar larvae feeding on an artificial diet [69] were also harvested. Larvae were surface sterilized, dissected, and their midguts were removed and frozen in liquid nitrogen. RNA was isolated, and ribosomal RNA was depleted from the sample using a Ribominus Eukaryotic Kit for RNA-seq (Life Technologies). The enriched mRNA was further poly(A) purified and multiplexed Illumina libraries were constructed using the TruSeq RNA Sample Prep kit (Illumina, San Diego, CA, USA). Samples were pooled and sequenced on a single Illumina HiSeq lane at the University of Delaware Biotechnology Institute (Newark, DE, USA) to generate approximately 13 million 101-nucleotide paired-end reads per sample. Forward reads were trimmed and quality filtered using ea-utils (https://expressionanalysis.github.io/ea-utils/) and high quality reads of at least 75 nucleotides in length were mapped to the *A. glabripennis* reference genome assembly using TopHat [70]. Read counts that mapped to each locus (version v0.5.3 annotations) were summed using HTSeq [71]; reads that spanned multiple features were summed using the union mode and reads that did not map uniquely to a single region in the genome were discarded. Differential expression analysis was performed using edgeR [72]. Features with less than ten mapped reads were removed from the analysis, read counts were normalized by quantile normalization, and variances were estimated using tagwise dispersions. Statistical analysis was performed using Fisher’s exact tests; features were flagged as differentially expressed if they had a log fold change greater than 1.0 and an adjusted *p* value of <0.05. Experiment-wise false discovery rate (FDR) was estimated at 0.05. The raw Illumina reads used for the differential expression analysis have been deposited into NCBI’s Sequence Read Archive (SRA) and are associated with Bioproject PRJNA279780. The read counts used to compute differential expression have been deposited in Gene Expression Omnibus (GEO) under the accession GSE68149.

### In vitro functional characterization of plant cell wall degrading enzymes

*A. glabripennis* larval samples were obtained from D. Lance (USDA-APHIS-PPQ). Larvae were chilled on ice and cut open; midguts from 1.5-month-old, 4-month-old, and 8-month-old larvae were collected and stored in an excess of RNA Later solution (Ambion) prior to shipping. RNA was subsequently isolated using the innuPREP RNA Mini Kit (Analytik Jena) according to the manufacturer’s protocol. Genomic DNA contamination was removed by DNAse treatment (TURBO DNAse, Ambion) for 30 min at 37 °C. Midgut RNA was further purified using the RNeasy MinElute Clean up Kit (Qiagen) following the manufacturer’s protocol and eluted in 20 μl of RNA storage solution (Ambion). Integrity and quality of the RNA samples were determined using the RNA 6000 Nano LabChip kit (Agilent Technologies) on an Agilent 2100 Bioanalyzer (Agilent Technologies) according to the manufacturer’s instructions.

Open reading frames encoding putative PCWDEs were amplified by PCR using gene-specific primers. The forward primer was designed to introduce a 5′ Kozak sequence, and the reverse primer was designed to omit the stop codon. Equal amounts of total RNA prepared from midguts either of 1.5-month-old or 4-month-old or 8-month-old larvae were pooled, and 1 μg total RNA from this pool was used to generate first-strand cDNAs using the SMARTer RACE cDNA Amplification Kit (BD Clontech) following the manufacturer’s instructions. These cDNAs were subsequently used as templates for PCR amplifications. PCR products were cloned into the pIB/V5-His TOPO/TA (Invitrogen) vector, in frame with a V5-(His)_6_ epitope at the carboxyl terminus. Constructs were transfected into insect *Sf*9 cells, which were grown to confluence, and expression of the recombinant proteins was validated as described previously [7]. Diffusion assays were performed using 1 % agarose Petri dishes in McIlvaine buffer (pH 5.0) containing one of the following substrates: 0.1 % carboxymethylcellulose (CMC, Sigma-Aldrich); 0.1 % beechwood xylan (Sigma-Aldrich); 0.1 % xyloglucan from tamarind seeds (Megazyme); 0.1 % pectin from citrus peels (Sigma-Aldrich); 0.1 % demethylated polygalacturonic acid (Megazyme). Enzyme activity was detected using a 0.1 % Congo Red solution as described previously [7].

TLC analysis of hydrolysis reaction products was also performed. The culture medium of transiently transfected cells was first dialyzed against distilled water at 4 °C for 48 h, using Slide-A-Lyzer Dialysis Cassettes with a 10-kDa cutoff, before being desalted with Zeba Desalt Spin Columns with a 7-kDa cutoff (both Thermo Scientific), according to the manufacturer’s instructions. Enzyme assays (20 μl) were set up using 14 μl of dialyzed and desalted crude enzyme extracts mixed with 4 μl of a 1 % substrate in solution in a 20 mM McIlvaine buffer (pH 5.0). For GH5-1 to -6, the following substrates were tested: carboxymethyl cellulose (CMC), avicel (suspension), beechwood xylan, and xyloglucan. For GH28s, the following substrates were tested: demethylated polygalacturonic acid and pectin from citrus peels. The activity of GH28s on 10 μg/μl aqueous solution of tri- and di-galacturonic acid was also tested. Enzyme assays were incubated and plates developed as described previously [7].

Amino acid alignments were carried out using MUSCLE version 3.7 on the Phylogeny.fr web platform (http://www.phylogeny.fr) [73] and were inspected and corrected manually when needed. Bayesian analyses were carried out in MrBayes 3.1.2 [74]. Two runs were conducted for the dataset showing agreement in topology and likelihood scores. To obtain support from a second independent method, maximum likelihood analyses were also performed using MEGA5 [75]. The robustness of each analysis was tested using 1000 bootstrap replicates.

### Comparative genomics of phytophagy and detoxification across Insecta

Gene families and subfamilies associated with phytophagy (particularly xylophagy) and polyphagy or detoxification were identified by searching for matches to relevant InterPro domains in the complete gene sets from the genomes of 15 exemplar insect species. These included five beetles (*A. glabripennis*, *D. ponderosae*, *T. castaneum*, *A. planipennis* (unpublished), and *O. taurus* (unpublished)); five basal insects (*Zootermopsis nevadensis* (dampwood termite, family Termopsidae), *Pediculus humanus* (human louse, family Pediculidae), *A. pisum*, *Apis mellifera* (honey bee, family Apidae), and *Nasonia vitripennis* (jewel wasp, family Pteromalidae)); two lepidopterans (*Plutella xylostella* (diamondback moth, family Plutellidae) and *Danaus plexippus* (monarch butterfly, family Nymphalidae)); and three dipterans (*Mayetiola destructor* (Hessian fly, family Cecidomyiidae), *D. melanogaster*, and *Anopheles gambiae* (African malaria mosquito, family Culicidae)). Protein domains were annotated with InterProScan5 [76] using the following domain libraries: PfamA-27.0, PrositeProfiles-20.97, SMART-6.2, SuperFamily-1.75, and PRINTS-42.0. The gene families examined included glycoside hydrolases, peptidases, esterases, cytochrome P450s, and UDP-glucosyltransferases.

The classifications based on InterPro domain counts were used only for those cases where the maximum gene count in a given species was greater than 5 (i.e., at least one species had a potential expansion of more than five genes). The orthology status of each of these identified genes was assessed using OrthoDB v8 [20] to determine if the gene was found as a single-copy ortholog, or with co-orthologs, or whether it showed homology to the domain but was not classified in any orthologous group. The results of the counts of each relevant domain type and the orthology status for the identified genes are given in Additional file 1: Tables S17–S26. Domains were selected for plotting from the complete list to avoid redundant domains (e.g., subfamilies rather than families and just one of N/C-terminal domains). For each gene family, the bar charts were plotted with largest subfamily at the bottom and smallest at the top, showing the counts for each subfamily per species (Additional file 1: Figures S18–S22). The orthology status of genes in the subfamily bar charts (i.e., those plotted and where at least one species has more than five genes) show the totals in each species partitioned into homologs and single-copy and multi-copy orthologs (Additional file 1: Tables S19–S23).

More information on methods is available in Additional file 1, and supporting scripts are available at https://github.com/NAL-i5K/AGLA_GB_supp-scripts.

## Additional files

Additional file 1: Supplementary figures, tables, methods, and other text. (DOCX 37347 kb)
Additional file 2: Large supporting tables. (XLSX 344 kb)
