# Supplementary material for: Genome of the Asian longhorned beetle (Anoplophora glabripennis), a globally significant invasive species, reveals key functional and evolutionary innovations at the beetle–plant interface
Source: Genome Biol. 2016 Nov 11;17:227. doi: 10.1186/s13059-016-1088-8 (PMC5105290; doi:10.1186/s13059-016-1088-8)
Supplement: Additional file 1: — Supplementary figures, tables, methods, and other text. (DOCX 37347 kb) [file 13059_2016_1088_MOESM1_ESM.docx]

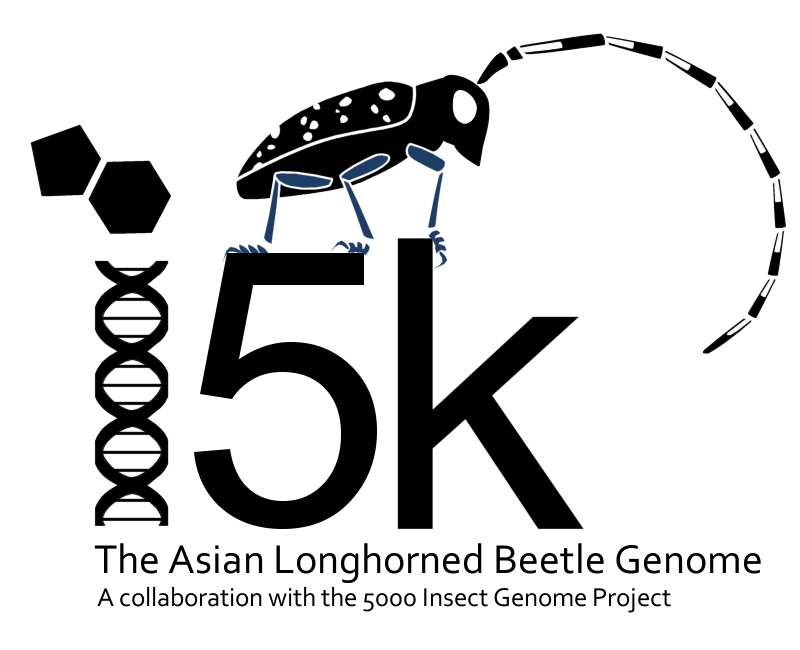


**SUPPLEMENTARY INFORMATION**

**(Additional File 1)**

(logo: R. Mitchell)

**The International Asian Longhorned Beetle Genome Consortium**

**SUPPLEMENTARY INFORMATION**

**I. General Features of the *Anoplophora glabripennis* Genome (Page 5)**

I.1 Genome size (S. Johnston)

I.2 DNA and RNA for sequencing (D.D. McKenna, D.R. Lance)

I.3 Genome sequencing and assembly (S. Richards, D.S.T. Hughes, S.C. Murali, J. Qu, S. Dugan, S.L. Lee H. Chao, H. Dinh, Y. Han, H. Doddapaneli, K.C. Worley, D.M. Muzny, R.A. Gibbs)

I.4 Automated gene annotation using Maker 2.0 (D.S.T. Hughes, S. Richards)

I.5 Community curation (M. Poelchau, C. Childers, C. Lee, H. Lin)

I.6 OrthoDB orthology, BUSCO assessment, and phylogenomic analysis (R. M. Waterhouse, P. Ioannidis, E. M. Zdobnov)

I.7 Genome organization and chromosome synteny (H. Blackmon, J. Demuth)

I.8 Candidate bacterial horizontal gene transfers and potential contaminating bacterial scaffolds (A. Dolan, C. Silva, J. Werren)

**II. Digestive Physiology and Specialized Phytophagy (Page 38)**

II.1 Plant cell wall and lignin degradation (R. Kirsch, Y. Pauchet)

II.2 Gene expression analysis of plant cell wall degrading enzymes (PCWDEs) in *A. glabripennis* larvae (E. Scully, K. Hoover, S. Geib)

II.3 *In vitro* functional characterization of *A. glabripennis* PCWDEs (R. Kirsch, A. Busch, Y. Pauchet)

II.4 Horizontal gene transfer and PCWDEs (Y. Pauchet)

**III. Detoxification and Polyphagy (Page 74)**

III.1 Cytochrome P450s (D.R. Nelson, S. Haddad)

III.2 UDP-glycosyltransferases (S. Ahn)

III.3 Carboxylesterases (E. Scully)

III.4 Myrosinase and cyanogenic β-glycosidase-like sequences (L. Kuhn, A. Ray, E. Scully)

III.5 Digestive proteinases (E. Scully)

**IV. Comparative Genomics of Phytophagy and Detoxification (Page 108)**

(R. M. Waterhouse, J. Duan, S. Geib, D. McKenna, A. Moczek, E. Scully)

**V. Sensory Biology: Olfaction, Gustation and Vision (Page 135)**

V.1 Chemoperception (H. Robertson, R. Mitchell, T. Bledsoe)

V.2 Vision (M. Friedrich, J. Jones)

**VI. Growth and Development (Page 144)**

VI.1 Bone morphogenic protein signaling pathway (J.A. Lynch, H. Quan, L. Cohen, D. Arsala, D. Pers)

VI.2 Germline genes (J.A. Lynch, H. Quan, L. Cohen, D. Arsala, D. Pers)

VI.3 Homeodomain transcription factors (Hox, Iro-C) (K.A. Panfilio, I.M. Vargas Jentzsch)

VI.4 Embryo and imaginal disc patterning genes (J.A. Lynch, H. Quan, L. Cohen, D. Arsala, D. Pers)

VI.5 Dorso-ventral fate specifying factors (J J.A. Lynch, H. Quan, L. Cohen, D. Arsala, D. Pers)

VI.6 Nuclear receptors (S.R. Palli)

VI.7 Toll pathway (J J.A. Lynch, H. Quan, L. Cohen, D. Arsala, D. Pers)

VI.8 RNAi pathway (S. Geib, B. Calla)

VI.9 Cuticular proteins (A.J. Rosendale, J.B. Benoit; K.A. Panfilio)

VI.10 DNA methylation (K.M. Glastad, M.A.D. Goodisman)

**VII. Diapause and Stress Tolerance (Page 179)**

(G. Yocum, A.K. Bennett, J.H. Bowsher, J.M. Johnson, J.P. Rinehart, R.L. Roehrdanz, A.C. Torson)

**VIII. Life Cycle (Page 181)**

**IX. Supplemental References (Page 183)**

Mention of trade names or commercial products in this publication is solely for the purpose of providing specific information and does not imply recommendation or endorsement by the U.S. Department of Agriculture. USDA is an equal opportunity provider and employer.**I. General Features of the *Anoplophora glabripennis* Genome**

**I.1 Genome size**

**Contributor: J. Spencer Johnston (**Texas A & M University, Department of Entomology)

**Summary**

The genome size of *Anoplophora glabripennis* was estimated from 10 individuals (five males, five females) and is reported as the theoretical average genome size of a gamete (represented as the quantity 1C), and expressed as 1C +/- 1 standard error. In general, 1C is the actual gametic size for the homogametic sex (female). For the heterogametic sex (male), it is calculated as the average between the X-bearing and the Y-bearing (or lack of X in an X/O system) gamete. To determine the genome size difference between the X-bearing and the Y bearing gamete, the difference is then taken between the 2C female (2A XX) and the 2C male (2A XY).

Graduate students in the L. Hanks Laboratory (University of Illinois at Urbana-Champaign) collected the *A. glabripennis* specimens from the site of the Chicago infestation. Methods for estimating genome size followed those in [1]. Briefly, we combined neural tissue from the head of an *A. glabripennis* sample with the intact head of a *Drosophila virilis* standard (1C = 328 Mbp) in a 2ml Dounce tissue grinder with 1ml of Galbraith buffer. The combined tissues were ground with 15 strokes of the "B" pestle and filtered through a 40u nylon filter, then stained in the cold and dark for at least 20 minutes with 25 mg of propidium iodide. The relative fluorescence of the stained DNA in the diploid nuclei of both the sample and the standard was scored using a Partec Cyflow flow cytometer, with green laser excitation set at 532nm and with a long pass filter (> 590 nm). The quantity of DNA was scored as the ratio of the mean fluorescence of the sample (plus standard) multiplied by the amount of DNA in the standard, and is expressed as 1C = the amount of DNA in a gamete.

For our samples, the difference in genome size between the X-bearing and Y-bearing gametes was 21.5 Mbp. The average male genome size was 1C = 970.6 +/- 2.5 Mbp and, as excepted, was smaller than the average female genome size of 1C = 981.4 +/- 3.5 Mbp. The female genome size of *A. glabripennis* is well within the female genome size range of other known long-horned beetles (Cerambycidae). These include values for another 7 species and average 864 Mbp and range from 1C = 497 Mbp for a *Phymatodes testaceus* female to 1C = 1282 Mbp for an *Anelaphus moestus* female. The larger standard error for the female genome size reflects a single, unusually smaller value of 1C = 969.9 Mbp for one of our samples. Excluding this value, the average female genome size increases and has a smaller standard error (1C = 984.3 +/- 2.6 Mbp; *n* = 4). However, we prefer the smaller estimate of 981.4 Mbp because it accounts for an apparently widespread, though poorly understood feature of coleopteran genomes (but not those of other insect orders), in which female genome size ranges frequently overlap those of males (but not vice versa). In our study, and those of other coleopteran genomes, sex determination is known to have been accurate (this being morphologically trivial in those cases) and so we are confident that the exceptional female genome size in our study is nevertheless an accurate sample point. This anomalous feature of coleopteran genomes may indicate the presence of widespread female sex chromosome heterogeneity within the order, and future work into this genomic characteristic is warranted.

**I.2** **DNA and RNA for sequencing**

**Contributors**

**Duane D. McKenna** (University of Memphis, Department of Biological Sciences)

**David R. Lance** (United States Department of Agriculture, Animal and Plant Health Inspection Service, Plant Pest and Quarantine, Center for Plant Health Science and Technology, Otis Laboratory)

**Summary**

The specimens sequenced for this project were live-frozen at -80 °C or in liquid nitrogen, shipped on dry ice from the laboratory of D. Lance to D. McKenna, and stored at -80 °C until used for DNA and RNA extraction. The beetles used (a larva for DNA, a larva and male and female adults for RNA) were from a “mixed colony,” containing beetles collected from the Chicago, IL, Brooklyn, NY, and Worcester, MA infestations. DNA was extracted from a single female larva (see section II.2) using the Omniprep column-free DNA extraction kit from G-Biosciences Inc. (<http://www.gbiosciences.com/OmniPrep-desc.aspx>; St. Louis, MO). The larva was cut into several pieces while frozen at -80C and dissected to remove the head, terminus and gut. DNA was extracted from the remains of the larva in 4 separate extraction tubes because of the large volume of tissue used, and pooled after extraction. We extracted DNA from a larva because in our experience larvae produced larger DNA fragments, on average, than extractions from adults. The manufacturer’s extraction protocol was followed, except that we incubated with proteinase K for 6 hours. The resulting genomic DNA was run on a 1% agarose gel, treated with RNAse, and quantified via Qubit (Life Technologies Inc.; Grand Island, NY) (Table S1). RNA was extracted using the Qiagen RNeasy Mini Kit (Qiagen Inc.; Valencia, CA) following the manufacturer’s instructions.

Additional RNA-Seq data (used to assist with manual annotation) were obtained from larvae in the Hoover lab (The Pennsylvania State University; PSU). Specifically, tissue from a pool of four whole *A. glabripennis* larvae (reared in sugar maple in the PSU quarantine greenhouse) was macerated and RNA was extracted using the PowerMicrobiome RNA Isolation Kit (MoBio, Carlsbad, CA).

**Table S1. DNA and RNA used for RNAseq and DNA sequencing.**

| **Sample ID** | **Life Stage extracted** | **Sex** | **Sample Type** | **Conc. (ng/ul)** | **Vol. (ul)** | **Total (ug)** | **Notes 1** | **Notes 2** |
| --- | --- | --- | --- | --- | --- | --- | --- | --- |
| DDM3004 | Adult | female | Total RNA | Not estimated | 100+ | Not estimated | In nuclease-free water | Extracted from lateral ½ of body |
| DDM3005 | Adult | male | Total RNA | Not estimated | 100+ | Not estimated | In nuclease-free water | Extracted from lateral ½ of body |
| DDM3006 | Larva | Unknown | Total RNA | Not estimated | 100+ | Not estimated | In nuclease-free water | Extracted from entire larva except head, terminus, and gut contents |
| DDM3010 | Larva | Unknown, later determined to be female | DNA | 200 | 425 | 85 | In TE buffer | Extracted from entire larva except head, terminus, and gut contents |

**I.3 Genome sequencing and assembly**

**Contributors**

**Stephen Richards**, **Daniel S.T. Hughes, Shwetha C. Murali, Jiaxin Qu, Shannon Dugan, Sandra L. Lee, Hsu Chao, Huyen Dinh, Yi Han,** **HarshaVardhan Doddapaneli, Kim C. Worley,** **Donna M. Muzny and** **Richard A. Gibbs** (Baylor College of Medicine, Department of Human and Molecular Genetics, Human Genome Sequencing Center)

**Summary**

We used an enhanced Illumina-ALLPATHS-LG sequencing and assembly strategy (see Table S2 for a summary of software used). We sequenced four libraries of nominal insert sizes 180 bp, 500 bp, 3 kb and 8 kb at genome coverages of 59.7X, 45.8X, 58.7X and 20.5X respectively (assuming a 710 Mb genome size). These raw sequences have been deposited in the NCBI SRA; the accessions are shown in Table S3, BioSample ID: SAMN02222868.

To prepare the 180 bp and 500 bp libraries, we used a gel-cut paired end library protocol. Briefly, 1 µg of DNA was sheared using a Covaris S-2 system (Covaris, Inc. Woburn, MA) using the 180-bp or 500-bp program. Sheared DNA fragments were purified with Agencourt AMPure XP beads, end-repaired, dA-tailed, and ligated to Illumina universal adapters. After adapter ligation, DNA fragments were further size-selected by agarose gel and PCR-amplified for 6 to 8 cycles using Illumina P1 and Index primer pair and Phusion® High-Fidelity PCR Master Mix (New England Biolabs). The final library was purified using Agencourt AMPure XP beads and quality was assessed by Agilent Bioanalyzer 2100 (DNA 7500 kit), determining library quantity and fragment size distribution before sequencing.

Long mate pair libraries with 3 kb and 8 kb insert sizes were constructed according to the manufacturer’s protocol (Mate Pair Library v2 Sample Preparation Guide art # 15001464 Rev. A PILOT RELEASE). Briefly, 5 µg (for 3 kb gap size library) or 10 µg (8 kb gap size library) of genomic DNA was sheared to desired size fragments by Hydroshear (Digilab, Marlborough, MA), then end-repaired and biotinylated. Fragment sizes between 3 - 3.7 kb (3 kb) or 8-10 kb (8 kb) were purified from 1% low melting agarose gel and then circularized by blunt-end ligation. These size-selected circular DNA fragments were then sheared to 400-bp (Covaris S-2), purified using Dynabeads M-280 Streptavidin Magnetic Beads, end-repaired, dA-tailed, and ligated to Illumina PE sequencing adapters. DNA fragments with adapter molecules on both ends were amplified for 12 to 15 cycles with Illumina P1 and Index primers. Amplified DNA fragments were purified with Agencourt AMPure XP beads. Quantification and size distribution of the final library was determined before sequencing as described above.

Sequencing was performed on Illumina HiSeq 2000s generating 100 bp paired end reads. Reads were assembled using ALLPATHS-LG (v35218) [2] and further scaffolded and gap-filled using in-house tools Atlas link (v.1.0) and Atlas gap-fill (v.2.2) (https://www.hgsc.bcm.edu/software/). This yielded an assembly of size 710 Mb with contig N50 of 16.5 kb and scaffold n50 of 659 Mb. The assembly has been deposited in the NCBI: BioProject PRJNA167479.

| **Table S2: Software used and URLs** |
| --- |

| Category | Software | URL | | |  |
| --- | --- | --- | --- | --- | --- |
| Genome Assembly: | SeqPrep | https://github.com/jstjohn/SeqPrep | |  |  |
|  | ALLPATHS-LG v35218 [2] | http://www.broadinstitute.org/software/allpaths-lg/blog/ |  |  |  |
|  | Atlas-Link v1.0 | https://www.hgsc.bcm.edu/software/atlas-link | |  |  |
|  | Atlas Gap-Fill V2.2 | https://www.hgsc.bcm.edu/software/atlas-gapfill | |  |  |
|  | Assemblathon_stats.pl [247] | <http://korflab.ucdavis.edu/datasets/Assemblathon/Assemblathon2> | |  |  |
| Automated Genome Annotation | CEGMA [248] | <http://korflab.ucdavis.edu/Datasets/cegma/> | |  |  |
| *= included within Maker run. | Maker 2.22 [3] | http://www.yandell-lab.org/software/maker.html | |  |  |
|  | Repeat masker* | http://www.repeatmasker.org |  |  |  |
|  | SNAP* | http://korflab.ucdavis.edu/software.html | |  |  |
|  | Exonerate* [7] | https://www.ebi.ac.uk/about/vertebrate-genomics/software/exonerate |  |  |  |
|  | Augustus* [249] | http://bioinf.uni-greifswald.de/augustus/ | |  |  |
|  | Interpro Scan5 [141] | https://www.ebi.ac.uk/interpro/interproscan.html | |  |  |
|  | BUSCO [250] | http://busco.ezlab.org | |  |  |
| Gene Modeling Curation | WebApollo [6] | [http://genomearchitect.org](https://apollo.nal.usda.gov/anogla/jbrowse/) | |  |  |
|  | i5K Workspace [8] | https://i5k.nal.usda.gov/Anoplophora_glabripennis and https://apollo.nal.usda.gov/anogla/jbrowse/ | |  |  |
|  | OGS post processing commands | https://github.com/NAL-i5K/AGLA_GB_supp-scripts | | | |
| HGT detection | BLASTN [37] | http://blast.ncbi.nlm.nih.gov/Blast.cgi | |  |  |
| Gene Organization and Chromosome Synteny | TBLASTN [37] | http://blast.ncbi.nlm.nih.gov/Blast.cgi | |  |  |
|  | smallPhylogeny [251] | http://137.122.149.195/Softwares/smallPhylogeny/smallPhylogeny.html | |  |  |
| AminoAcid Alignments and Trees | MUSCLE v3.7 [31] | http://www.phylogeny.fr | | |  |
|  | MrBayes 3.1.2 [73] | http://mrbayes.sourceforge.net | | |  |
|  | MEGA5 [74]. | http://www.megasoftware.net | | |  |

**Table S3. Sequencing, assembly, annotation statistics, and accession numbers.**

| Bio Projects | i5K Pilot NCBI Bio-project | PRJNA163973  http://www.ncbi.nlm.nih.gov/bioproject/163973 |
| --- | --- | --- |
|  | *Anoplophora glabripennis* NCBI Bio-project | PRJNA167479 http://www.ncbi.nlm.nih.gov/bioproject/167479 |
|  | NCBI Bio-sample | SAMN02222868 http://www.ncbi.nlm.nih.gov/biosample/SAMN02222868 |
| Genome Sequence | 180 bp insert (insert size 164 bp) | 209.7 M spots, 42.4 Gbp |
|  | 500 bp insert (insert sizes 385 and 324 bp) | 2 runs, 160.5 M spots, 32.5 Gbp |
|  | 3 kb insert (insert size 3,177 bp) | 206.4 M spots, 41.7 Gbp |
|  | 8 kb insert (insert size 6,967 bp) | 72.4 M spots, 14.6 Gbp |
|  | 180 bp insert NCBI SRA Accession | SRX326764  <http://www.ncbi.nlm.nih.gov/sra/SRX326764> |
|  | 500 bp insert NCBI SRA Accession | SRX326768 and SRX326767  <http://www.ncbi.nlm.nih.gov/sra/SRX326768> and <http://www.ncbi.nlm.nih.gov/sra/SRX3267687> |
|  | 3 kb insert NCBI SRA Accession | SRX326766  <http://www.ncbi.nlm.nih.gov/sra/SRX326766> |
|  | 8 kb insert NCBI SRA Accession | SRX326765  <http://www.ncbi.nlm.nih.gov/sra/SRX326765> |
| Genome Assembly | Number of contigs | 91,083 |
|  | Contig N50 | 16,551 bp |
|  | Number of scaffolds | 10,473 |
|  | Scaffold N50 | 658,851 bp |
|  | Size of final assembly | 707,712,193 bp |
|  | Size of final assembly - without gaps | 602,426,910 bp |
|  | NCBI Genome Assembly Accession | GCA_000390285.1  http://www.ncbi.nlm.nih.gov/assembly/GCA_000390285.1 |
| RNAseq Data | Adult Male RNAseq reads | 33.3 M spots, 6.7 Gbp |
|  | Adult Female RNAseq reads | 30 M spots, 6.1 Gbp |
|  | Adult Male RNAseq SRA Accession | SRX873913  http://www.ncbi.nlm.nih.gov/sra/SRX873913 |
|  | Adult Female RNAseq SRA Accession | SRX873912  http://www.ncbi.nlm.nih.gov/sra/SRX873912 |
| Automated Genome Annotation (Agla_0.5.3) | Genes (AGLA_0.5.3) | 22,035 |
|  | Average Transcript length | 1,476 bp |
|  | Average CDS length | 1,126 bp (375 aa) |
|  | Exons per gene | 4.66 |
|  | Genome Annotation Link | National Agricultural Library  https://i5k.nal.usda.gov/Anoplophora_glabripennis |

**I.4 Automated gene annotation using Maker 2.0**

**Contributors**

**Daniel S.T. Hughes and** **Stephen Richards** (Baylor College of Medicine, Department of Human and Molecular Genetics, Human Genome Sequencing Center)

**Summary**

The *Anoplophora glabripennis* genome was subjected to automatic gene annotation using a Maker 2.0 annotation pipeline tuned specifically for arthropods. The pipeline was designed to be systematic, providing a single consistent procedure for annotation of the genome assemblies for species in the pilot study, and scalable to handle 100’s of genome assemblies. Both protein and RNA-seq evidence from extant arthropod gene sets were used to guide gene models. The core of the pipeline was a Maker 2 instance, modified slightly to enable efficient running on our computational resources [3]. The genome assembly was first subjected to de-novo repeat prediction and CEGMA analysis to generate gene models for initial training of the ab-initio gene predictors. Three rounds of training of the Augustus [4] and SNAP [5] gene predictors within Maker were used to bootstrap to a high quality training set. Input protein data included 1 million peptides from a non-redundant reduction (90% identity) of Uniprot Ecdysozoa (1.25 million peptides) supplemented with proteomes from eighteen additional species (*Strigamia maritima, Tetranychus urticae, Caenorhabditis elegans, Loa loa, Trichoplax adhaerens, Amphimedon queenslandica, Strongylocentrotus purpuratus, Nematostella vectensis, Branchiostoma ﬂoridae, Ciona intestinalis, Ciona savignyi, Homo sapiens, Mus musculus, Capitella teleta, Helobdella robusta, Crassostrea gigas, Lottia gigantea,* and *Schistosoma mansoni*) leading to a final nr peptide evidence set of 1.03 million peptides. RNAseq from *A. glabripennis* adult males and females was used judiciously to identify exon-intron boundaries but with a heuristic script to identify and split erroneously joined gene models. We compared to a standard set of models for QC purposes: for *A. glabripennis*, of 1977 single copy ortholog gene models available in all arthropods prior to the BCM-HGSC i5K pilot project, 1947 were found in the assembly, and 1920 in the final predicted gene set. Finally, the pipeline used a nine-way homology prediction with human, *Drosophila melanogaster* and *C. elegans*, and InterPro Scan5 to allocate gene names. The automated gene set is available from the BCM-HGSC website (https://www.hgsc.bcm.edu/asian-long-horned-beetle-genome-project) and at the National Agricultural Library (<https://i5k.nal.usda.gov>).

**I.5 Community curation**

**Contributors**

**Monica F. Poelchau and** **Christopher Childers** (USDA-ARS National Agricultural Library)

**Chien-Yueh Lee and** **Han Lin** (USDA-ARS National Agricultural Library, National Taiwan University, Graduate Institute of Biomedical Electronics and Bioinformatics)

**Author contributions:**

M.F.P. performed post-processing of the OGS, contributed to the design of gff-cmp-cat, and wrote this section; C.C. implemented and maintained Web Apollo, and contributed to the design of gff-cmp-cat; C.-Y. L. and H.L. designed and ran software.

**Summary**

The *Anoplophora glabripennis* genome was curated to improve the structural and functional annotations of genes and gene families of interest using the Web Apollo manual curation tool [6]. Through this process we curated a total of 1,144 gene models encompassing 1,168 mRNAs and 66 pseudogenic transcripts. Two hundred thirty eight [238] mRNAs and 66 pseudogenic transcripts were newly added (Table S4), and 1 mRNA was removed without replacement. We created an official gene set (OGS) by merging the computationally predicted genes with the manual curations. We then developed a program that categorizes the structural changes between the predicted and curated genes. There was no evident bias towards any type of structural annotation change in the manually curated genes, suggesting that the computational gene prediction method did not carry any strong biases. Nevertheless, a substantial number of new genes were added during the manual curation process, most of which were chemoreceptors.

**Manual curation methods**

The *A. glabripennis* genome coordinator (D. McKenna) organized a group of experts to manually curate genes or gene families of interest in Web Apollo [6]. Web Apollo is an interactive, web-based manual curation tool that visualizes user-generated annotation changes in real time, allowing annotators to collaborate on annotations remotely following specific conditions for a curated gene. Annotators were provided an annotation guide (<http://genomearchitect.org/web_apollo_user_guide>), a set of standard operating procedures to adhere to (<https://i5k.nal.usda.gov/content/rules-web-apollo-annotation-i5k-pilot-project>), and offered optional training via webinar from the Web Apollo development team. The featured Web Apollo installation (<https://apollo.nal.usda.gov/anogla/jbrowse/>) keeps track of all evidence used for the MAKER gene predictions, as well as an additional RNA-Seq dataset that was not used in the generation of the MAKER gene predictions (contributed by E. Scully). Custom scripts were developed to convert RNA-Seq alignments to bigwig format for better display purposes (<https://github.com/hotdogee/bam_to_bigwig>). One annotator (D. Nelson) generated protein sequences that were aligned to the *A. glabripennis* genome via a custom modification of the exonerate protein2genome algorithm [7] that renders output in gff3 format (available on GitHub, <https://github.com/hotdogee/exonerate-gff3>. command line: exonerate -q queryfile -t targetfile --model protein2genome --querytype protein --targettype dna --showvulgar no --showalignment no --showtargetgff yes --showcigar no --minintron 20 --maxintron 30000 --geneseed 150 --score 250 --verbose 0 --gff3 yes). Each alignment was uploaded to Web Apollo and subsequently visually inspected and corrected.

After the curation period had ended, the manually curated models were inspected for quality, including overlapping models, internal stop codons within the CDS, gff3 formatting errors, and mixed transcript types within gene models. The quality-corrected models were then merged with the MAKER-predicted gene set to generate an Official Gene Set v1.2 using a custom script (D. Hughes) followed by post-processing to ensure curation information was transferred adequately.

**Evaluation of the manual curation process**

To generate a comprehensive list of manual curation actions, we developed a program (gff-cmp-cat, https://github.com/chienyuehlee/gff-cmp-cat) that 1) finds overlapping features at the gene, mRNA, exon and CDS level and 2) independently identifies the number of added, deleted, merged, split, extended, reduced, and otherwise changed genes and mRNAs in the manually curated gene set. A key assumption is that the predicted gene models do not have multiple isoforms. The program calculates the differences between overlapping genes and mRNAs between two gff3 files of gene annotations from the same genome assembly. For this analysis, the first file was the Maker set of computationally predicted genes (predicted file), and the second file contained the manual curations (curated file). Once loaded, the differences between overlapping genes and mRNAs from the predicted file to the curated file were calculated. The differences are categorized into nine ‘action’ types, described in Additional file 2: Table S5 in the Large Supplementary Tables excel file.

**Manual curation results**

Twenty four active annotators curated a total of 1,144 gene models encompassing 1,168 mRNAs and 66 pseudogenic transcripts. Two hundred and thirty eight [238] mRNAs and 66 pseudogenic transcripts were newly added (Table S4), and 1 mRNA was removed without replacement. The majority of added gene models were from the Odorant receptor, Gustatory receptor, and Ionotropic receptor families (see *Chemoperception*). After manual curation, there were 214 extended and 260 reduced mRNAs, and 68 mRNAs with internal CDS or exon coordinate changes that did not affect the boundaries of the model. Additionally, 109 of the curated mRNAs resulted from split computational models, and 61 curated mRNAs resulted from merges, indicating similar numbers of split and merge actions. Three hundred and twelve [312] mRNAs were left unchanged at the sequence level, but functional information was added, including names, symbols, descriptions, PubMed references, Gene Ontology categories, cross-references to other genes, and other comments explaining annotation actions as necessary. All functional information was included in OGS v1.2. Information on the *A. glabripennis* genome project is also collated at the i5k Workspace [8] (<https://i5k.nal.usda.gov/Anoplophora_glabripennis>), and the genome, transcript and protein sets can be searched via BLAST and browsed via the JBrowse genome browser [9] (<https://apollo.nal.usda.gov/anogla/jbrowse>). We provide a summary of all manually curated genes and transcripts and their curation actions as Additional file 2: Table S6.

**Discussion**

The purpose of the manual curation effort was not to comprehensively evaluate the MAKER gene predictions, but for curators to individually improve their genes of interest. Our detailed classification of the changes between the computationally predicted and manually curated genes allows us to make some general observations about the curation process. Assuming that the manually curated genes improved the computational predictions, there was no bias towards any type of sequence modification. This suggests that the computational gene prediction method used did not have strong biases itself. A substantial change to the dataset was the addition of gene models: 216 genes and 238 mRNAs were added to the dataset, the majority of which [225/239] were odorant, gustatory, and ionotropic receptors (see *Chemoperception*). Chemoreceptors tend to have highly specific expression patterns [10, 11], often leading to low RNA-Seq support. In addition, these gene families often diverge rapidly [12, 13], so evidence from protein alignments for computational gene prediction programs is often lacking. These two factors likely contributed to the poor prediction of these gene families by the MAKER program [14, 15].

**Table S4**. **Number of modifications performed during the manual curation process for different feature types of the *A. glabripennis* genome assembly v1.2.** Calculations for the ‘Action type’ are explained in the supplementary text.

| **Action type** | **gene** | **mRNA** | **pseudogene** | **pseudogenic transcript** |
| --- | --- | --- | --- | --- |
| Added Models | 216 | 238 | 66 | 66 |
| Deleted Models | 1 | 1 |  |  |
| Extended Models | 206 | 214 |  |  |
| Reduced Models | 237 | 260 |  |  |
| Models modified within boundary coordinates | 64 | 68 |  |  |
| Models resulting from merged CDS | 54 | 61 |  |  |
| Models resulting from split CDS | 102 | 109 |  |  |
| Models resulting from merged UTRs | 16 | 16 |  |  |
| Models resulting from split UTRs | 12 | 12 |  |  |
| All structurally modified models | 794 | 856 |  |  |
| Models with only functional annotations | 284 | 312 |  |  |
| **Total number of manually curated gene models** | 1078 | 1168 | 66 | 66 |

**Table S5.** **Descriptions of the conditions that need to be met for a curated gene, mRNA, exon, or CDS to be marked with an 'action type'.**

Due to its size, this table has been moved to the supplementary excel file entitled “AdditionalFile 2_04Aug2016”.

**Table S6. A summary of all curated genes, pseudogenes, mRNAs, and pseudogenic transcripts, and their curation actions**.

Due to its size, this table has been moved to the supplementary excel file entitled “AdditionalFile 2_04Aug2016”.

**SI.6 OrthoDB orthology, BUSCO assessment, and phylogenomic analysis**

**Contributors**

**Robert M. Waterhouse** (Department of Genetic Medicine and Development, University of Geneva Medical School, Geneva, Switzerland; Swiss Institute of Bioinformatics, Geneva, Switzerland; Computer Science and Artificial Intelligence Laboratory, Massachusetts Institute of Technology, Cambridge, MA, USA; The Broad Institute of MIT and Harvard, Cambridge, MA, USA)

**Panagiotis Ioannidis & Evgeny M. Zdobnov** (Department of Genetic Medicine and Development, University of Geneva Medical School, Geneva, Switzerland; Swiss Institute of Bioinformatics, Geneva, Switzerland)

**Summary**

Species selection:

**5 beetles:** AGLAB *Anoplophora glabripennis* (Asian longhorned beetle, this study)*,* DPOND *Dendroctonus ponderosae* (mountain pine beetle) [16]*,* TCAST *Tribolium castaneum* (red flour beetle) [17]*,* APLAN *Agrilus planipennis* (emerald ash borer)*,* OTAUR *Onthophagus taurus* (taurus scarab dung beetle).

**5 basal insects:** ZNEVA *Zootermopsis nevadensis* (dampwood termite) [18]*,* PHUMA *Pediculus humanus* (human body louse) [19]*,* APISU *Acyrthosiphon pisum* (pea aphid) [20]*,* AMELL *Apis mellifera* (honey bee) [21, 22]*,* NVITR *Nasonia vitripennis* (parasitoid jewel wasp) [23].

**5 lepidopterans-dipterans:** PXYLO *Plutella xylostella* (diamondback moth) [24]*,* DPLEX *Danaus plexippus* (monarch butterfly) [25]*,* MDEST *Mayetiola destructor* (hessian fly) [26]*,* DMELA *Drosophila melanogaster* (fruit fly) [27]*,* AGAMB *Anopheles gambiae* (malaria mosquito) [28].

**Orthology methods:** Orthology data from OrthoDB v8 [29] with a total of 87 arthropod species were analyzed to identify orthology and homology assignments of *Anoplophora glabripennis* genes with those of other beetles and representative species from six other insect orders. The gene sets of *Agrilus planipennis* and *Onthophagus taurus* were mapped to OrthoDB v8 orthologous groups to include them in the analysis. The selected species include several that feed on plants and were partitioned into three species sets as described above. Arthropod orthologous groups (OGs) were queried with custom Perl scripts to identify OGs with genes from all three species sets (across 15 species), just two sets (across 10 species), or restricted to a single set (across 5 species). To be considered shared, orthologous groups were required to contain genes from at least two species in each set. For those shared among all three sets (a total of 7’376 OGs), the numbers of single-copy and multi-copy orthologs were summed across all OGs for each species. Lineage-restricted genes without orthologs were assessed for significant homology (e-value <1e-05) to other arthropod genes from OrthoDB or for significant homology (e-value <1e-05) to genes from their own genomes (self-only homology).

**Orthology Results (Fig. 3, main paper):**

- *A. glabripennis* has a conserved core of 5,029 orthologs shared across all 15 insects (purple)
- *A. glabripennis* has a high number (6,880) of widespread orthologs (green) – maintained single-copy ancient genes (3,346, dark green), and maintained duplicated ancient genes (3,534, light green), although *A. pisum* has many more maintained duplicated ancient genes (8,779)
- Beetles maintain more ancient orthologs than the more derived Diptera and Lepidoptera: beetles-outgroup exclusive = 456 OGs compared with lep+dip-outgroup exclusive = 290 OGs
- Of the 5 beetles, *A. glabripennis* has the highest number of lineage-specific genes (5,229), although the fraction with no homology is small (1,003), e.g. *T. castaneum* has more unrecognizable genes (2,516).

Of the 4,019 *A. glabripennis* that lack orthology support, 2,789 showed significant homology (e-value <1e-05) to other arthropod genes from OrthoDB and a further 227 showed significant homology to genes from their own genomes (self-only homology). These are likely to be gene copies that have diverged beyond recognition for confident orthology assignments. Genes lacking orthology are also supported with matches to InterPro domains (1,022 genes) and with expression evidence from mapped RNA-seq transcripts (548). In addition, although the proteins without orthologs are expectedly generally shorter than those with orthology (mean amino acids 181 versus 419), the vast majority (3,643) are multi-exonic genes (mean exons for no-orthology genes = 3.24 versus 4.82 for genes with orthologs). Thus while some of the *A. glabripennis* genes without orthology support might be gene fragments or spurious *ab initio* predictions, the majority of the genes do show evidence of being true genes.

**Phylogenomic analysis (Fig. 2, main paper)**

For the phylogenomic analysis, we obtained 523 single-copy orthologs from OrthoDB [29], in order to build a concatenated phylogenetic tree with RAxML [30]. Briefly, a multiple sequence alignment was performed using muscle [31] for each orthologous group, separately. Then, the resulting alignments were trimmed using trimAl [32] with parameters “-w 3 -gt 0.95 -st 0.01”. The trimmed alignments were concatenated using the “seqret” program from the EMBOSS suite [33]. This concatenated alignment was used to build the phylogeny using RAxML 7.6.6 with the PROTGAMMA model of amino acid substitutions and 100 bootstrap replicates. The final tree was edited with TreeGraph 2.4 [34], Dendroscope 3.2.8 [35] and InkScape 0.9.1.

**BUSCO-based quality assessment (Fig. S1)**

For evaluating the quality of the *A. glabripennis* assembly we used the Benchmarking Universal Single-Copy Orthologs (BUSCO) [36] and assessed both the genome as well as the set of predicted genes. We used the Arthropoda gene set, which consists of 2,675 single-copy genes that are present in at least 90% of Arthropoda. The results show that has a completeness around average for Coleoptera, with the exception of the red flour beetle *T. castaneum*, which ranks higher. It is also worth noting that the number of missing genes from the official gene set (OGS) is relatively high in *A. glabripennis* as well as in the other beetles, except *T. castaneum*. This is in contrast with all other arthropods in which the missing genes are considerably less, except in the case of the diamondback moth, *P. xylostella*. However, when the results from the *A. glabripennis* OGS are compared to those obtained from the genome sequence, it is evident that the number of missing genes is reduced. This means that certain genes were missed during gene annotation.

**Figure S1. BUSCO analysis based on official gene sets (OGSs) and genome sequences.** The results show that the *A. glabripennis* OGS has about the same level of completeness as the other Coleoptera. Examining the genome sequence, however, shows that there are certain genes that were probably missed during gene annotation. The 2,675 BUSCOs at the Arthropoda level were used for the analysis. For each species, the bottom bar in the histogram shows the OGS-based results, whereas the top bar shows the genome-based results. The different orders of Holometabola have been colored as in Fig. 2 in the main paper. Species names abbreviations are the same as those used in Fig. 2 in the main paper (note that Dpule in the figure here is excluded from Fig. 2b in the main paper).

**
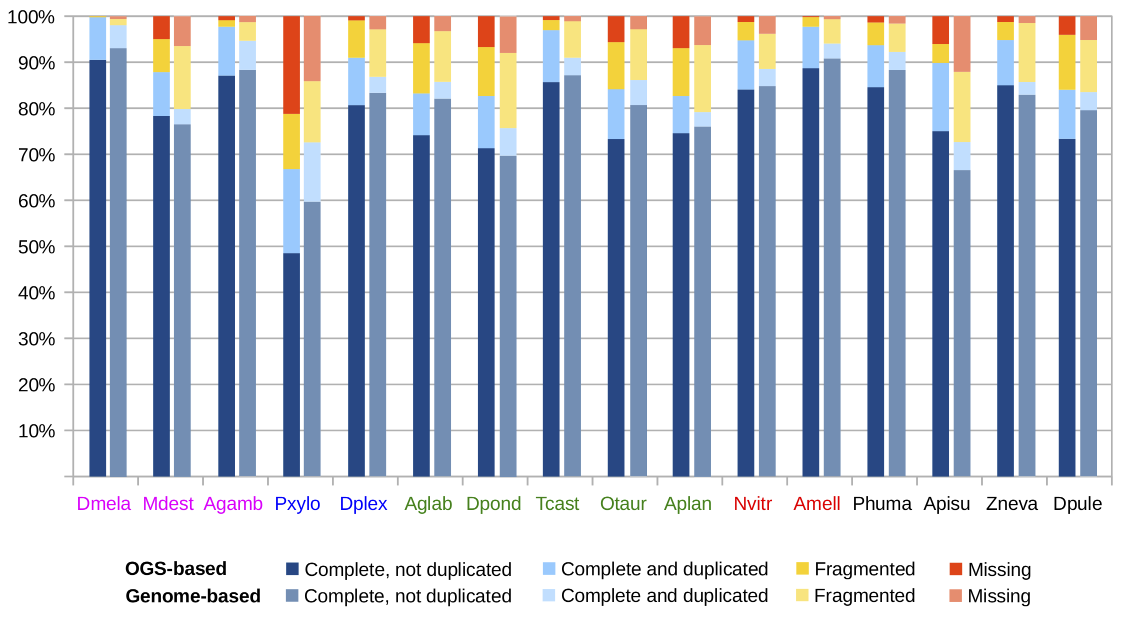
**

**I.7 Genome organization and chromosome synteny**

**Contributors**

**Heath Blackmon and Jeff Demuth (**University of Texas at Arlington, Department of Biology)

**Summary**

Mapping the assemblies of *Anoplophora glabripennis* and *Dendroctonus ponderosae* to the chromosomes of *Tribolium castaneum* reveals that amid overall widespread conservation of chromosome synteny, many smaller scale genome rearrangements have occurred (Fig. S2). In *A. glabripennis* at least 364 genes have experienced interchromosomal translocations. Further, based on ancestral genome reconstruction, we infer that a minimum of 408 total chromosomal rearrangements have occurred in *A. glabripennis* since its most recent common ancestor with the other two species. The most striking rearrangement is a 1.84 Mbp translocation from “chromosome 3” to “chromosome 4” containing 31 genes.

Although a lack of genetic linkage analyses in *A. glabripennis* and *D. ponderosae* limit the scope of our inferences based on mapping to *T. castaneum* (e.g., we cannot identify chromosomal fissions necessary to explain the n=9 to n=11 transition), the homology assignment between *T. castaneum* and the other species begins to lay the foundation for a standardization of the nomenclature for beetle chromosomes. This is valuable for situations like those we see in *D. ponderosae* where the homolog of chromosome 4 appears to be highly degenerated, suggesting that it is now part of the neo sex chromosomes.

**Methods.**

We used the *T. castaneum* version 3.0 assembly and gene annotations to identify genes that could be used as potential markers for synteny analysis of the *A. glabripennis* and *D. ponderosae* genomes. For inclusion in the analysis, we required confident orthology assignment and sufficient neighboring marker information to unambiguously assign synteny of chromosomal blocks. Orthology was inferred by reciprocal best blast hit (tblasx) [37] and filtered to remove matches with bit scores less than 100. We then evaluated whether there was sufficient consistency among markers on each *A. glabripennis* and *D. ponderosae* scaffold to unambiguously assign to a single chromosome in the *T. castaneum* assembly. To do this, we first numbered each marker according to its orthologous origin in *T. castaneum* (chromosome number and relative position). Next, for each scaffold in *A. glabripennis* and *D. ponderosae* we counted the number of markers from each orthologous *T. castaneum* chromosome. If the most common chromosome number on the scaffold was encountered more frequently than expected by chance given the total number of markers on the scaffold (binomial probability less than 0.01), we assigned the scaffold to the most common chromosome number. Finally, we ordered the assigned scaffolds based on their marker positions in the *T. castaneum* genome. Cross-species synteny was visualized using R [38]

To quantify the amount of genome rearrangement necessary to explain the patterns of synteny across the three genomes, we used the program smallPhylogeny [39] to perform ancestral genome reconstruction. Using the set of genes present in the assigned scaffolds, smallPhylogeny reconstructs the most parsimonious intermediate genome shared by *T. castaneum*, *A. glabripennis* and *D. ponderosae*. The program computes the minimum number of events (translocations, inversions, fusions, and fissions) required to convert each of the three genomes into an ancestral genome shared by the three extant species. Since smallPhylogeny accounts for changes in the strand on which a gene is found we also polarized the strand information in *A. glabripennis* and *D. ponderosae* to minimize differences in relation to *T. castaneum*.

We identified 2,447 and 2,984 orthologous marker genes for assignment of chromosome synteny in the *A. glabripennis* and *D. ponderosae* assemblies, respectively. Based on these markers we assigned 132 *A. glabripennis* scaffolds and 98 *D. ponderosae* scaffolds to their orthologous *T. castaneum* chromosomes. Since our mapping approach requires multiple genes to be assembled on a scaffold, larger scaffolds are more readily assigned homology to a *T. castaneum* chromosome. This is reflected in the mean size of assigned scaffolds. In the *A. glabripennis* assembly the mean scaffold size is 67 kb while the mean size of assigned scaffolds is 1.3 Mb; In the *D. ponderosae* assembly the mean scaffold size is 30 kb while the mean assigned scaffold is 1.2 Mb. Fragmented assemblies resulted in 2821 markers on 1003 scaffolds in *A. glabripennis* and 659 markers on 582 scaffolds in *D. ponderosae* that could not be assigned unambiguously to a *T. castaneum* chromosome. In total, 160MB (23%) and 117Mb (46%) of the *A. glabripennis* and *D. ponderosae* assemblies are assigned to homologous *T. castaneum* chromosomes.

Results & DIscussion.

The presence of markers within a scaffold that are inconsistent with its assigned chromosome is a clear signature of interchromosomal translocation. In *A. glabripennis* 14.8% (364 of 2,447) of markers show this signature (*D. ponderosae* = 17.4%; 520 of 2,984 markers). Ancestral genome reconstruction using smallPhylogeny [39] identifies 837 genes that can be positionally assigned in the most recent common ancestor of the three species. To explain the current organization of these genes across the three species requires 364 (*T. castaneum*), 408 (*A. glabripennis*), and 483 (*D. ponderosae*) chromosomal rearrangements on the respective branches (Fig. S2). Because these estimates are influenced by branch length and assembly quality, we repeated the analysis with an additional four unpublished but publicly available genome assemblies (*T. confusum, T. madens, T. freemani,* and *Onthophagus taurus*). Including the additional species reduces to 237 the number of markers that can be unambiguously assigned in the ancestor. However, the result of this additional analysis showed the same pattern; the branch leading to *A. glabripennis* has experienced fewer rearrangements (57) than has the branch leading to *D. ponderosae* (77).

Because we cannot at this time separate genome assembly contig scaffolding error from chromosomal re-arrangement this analysis should be thought of as initial with the scaffolds requiring orthoganol validation. However, the error mode of the scaffolding algorthyms used is to break scaffolds when un-certain, so we expect that the majority of identified chromosomal re-arrangements are correct.

Since we sequenced this genome from a larva, and since holometabolous insect larvae typically cannot be reliably sexed using morphological characters, we undertook an analysis of read depth to look for a peak showing reads mapping to the X chromosome (indicative of sequencing a male). Based on this analysis (Fig. S3), it is clear that we sequenced a female. The *A. glabripennis* figure shows no secondary peak indicating an X chromosome – hence it looks to have an equal X: autosome ratio, as expected for a female.

**Figure S2. Synteny among three beetle genomes.** Each marker gene that could be unambiguously assigned from the *A. glabripennis* or *D. ponderosae* genome scaffolds to an orthologous *T. castaneum* chromosome position is represented by an individual vertical line. Lines are color-coded based on their *T. castaneum* orthologous chromosome of origin. For clarity, gaps between markers are not shown, but scaling to base pairs (rather than marker number) does not affect the interpretation since gaps are similarly distributed among chromosomes (data not shown). Numbers below each beetle indicate the haploid autosome number and sex chromosome system. Y-chromosomes are not available since the specimen of *A. glabripennis* sequenced was a female. Branches on the phylogeny indicate the number of rearrangements necessary to explain genome organization since the three species’ most recent common ancestor.


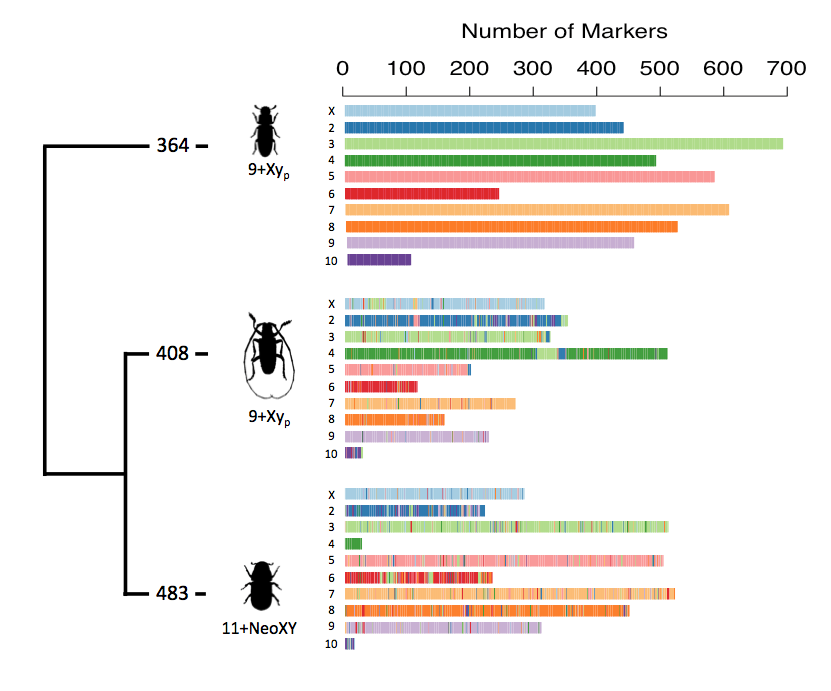


*D. ponderosae*

*T. castaneum*

*A. glabripennis*

**Figure S3. Analysis of read depth for *Anoplophora glabripennis.*** The lack of a secondary peak (which indicates the presence of an X chromosome) is consistent with having sequenced a female.

**I.8 Candidate bacterial horizontal gene transfers and potential contaminating bacterial scaffolds (**GH family genes are discussed separately**)**

**Contributors**

**Amanda Dolan, Christian Silva and John H. Werren** (University of Rochester, Department of Biology)

**Genome File Name**: Agla_Bt103082013.genome.fa downloaded from the I5K Baylor FTP website

**Summary**

*Non-glycosyl hydrolase HGT Candidates*

For *Anoplophora glabripennis*, 16 Horizontal Gene Transfer (HGT) candidates from bacteria were found using computational methods and confirmed by subsequent manual annotation. Of these, 5 have strong support based on the presence of eukaryotic or *A. glabripennis* flanking gene(s) and a large difference between prokaryotic and eukaryotic NBCI blast e-values and bitscores. Of the 5 strong HGT candidates, 4 were from bacteria most closely related to *Wolbachia* with 3 of these annotated as hypothetical proteins and 1 as putative major capsid protein. The fifth strong candidate is annotated as a hypothetical protein from a bacterium most closely related to Calothrix. None of these show significant expression based on RNA-seq reads for males, females, or larvae generated for this project. In addition, 4 are moderately strong candidates. These are moderately strong because they have a weaker (1e-5 to 1e-8) blastn hit and only to a single bacterial genus (*Nostoc, Buchnera* and *Rickettsia*) and one is near the end of a scaffold and could represent an assembly error (*Photorhabdus)*. The remaining 7 HGT candidates we consider to be weak. They may be real, but the evidence is relatively weak because they either fall within a repeat (identified by repeatmasker on Web Apollo) or have relatively strong blast matches to eukaryotes (See Additional file 2: Table S8 for more specific information). Of these 7 candidates, 3 fall within repeat regions (*Mycoplasma* and *Brachyspira)*, 4 have some eukaryotic similarity (and these are from *Clostridium, Buchnera, Marinomonas* and *Methylobacterium)*. Table S7 (below) shows summary information for the 8 strongest HGT candidates after additional manual curation of the HGT candidate list.

*Potential Bacterial Scaffolds*

In addition to HGT candidates, there were no candidate “contaminating” bacterial scaffolds identified. Typically we find scaffolds that are probably from bacteria and these most likely are from bacterial associates of the insect. However in this case it was somewhat expected because the larvae were dissected to remove head, terminus and gut from the sequenced individual prior to DNA isolation.

**Methods**

Two different computational pipeline scripts were used to identify HGT candidates and contaminating bacterial scaffolds in the genome assembly.

Information on “Old” Computational Pipeline

This method is most useful for identification of contaminating bacterial scaffolds in the assembly. The scaffold assemblies are run through this pipeline [40], which compares sequences for matches to ~1000 different bacterial genomes and compares the blast matches to a set of up to 9 reference eukaryotic genomes. For *A. glabripennis*, the animal database contained transcripts from the following animal genera: *Anopheles, Drosophila, Xenopus, Nasonia, Apis, Daphnia, Mus,* and *Homo.* Scaffold outputs are then sorted into potential horizontal gene transfers (HGTs), contaminating bacterial scaffolds or likely conserved scaffolds. Sorting is based on length of the scaffold, length of bacterial matches across the scaffold, and difference score in bacterial blast match relative to eukaryotic match [40]. This pipeline focuses on the best blastn bacterial hit (highest e-value) on each scaffold, and therefore additional regions with bacterial similarity were found manually using NCBI blastn.

Information on “New” Computation Pipeline

Since the old pipeline sorted scaffolds based on only the top prokaryotic hit information, there was concern that HGT candidates were being missed if they were not the best hit. The “new” computational pipeline breaks long scaffolds into 1,000bp intervals and searches each of them against the bacterial database. Any positive hits of the 1,000bp regions were then searched against the animal database. The bacterial database contained about 1,000 bacterial species and was masked for low complexity regions using the NCBI Dustmasker function. The animal database for *A. glabripennis* contained transcripts from a representative from each of the following animal genera: *Anopheles, Drosophila, Xenopus, Daphnia, Strongylocentrotus, Mus, Homo, Aplysia, Caenorhabditis, Hydra, Monosiga and Acanthamoeba*. The significance e-value cut-off used was 1e-5 for both the animal and bacterial hits. Regions of bacterial similarity that fell from the end of one 1,000bp interval to the adjacent interval were joined. Only the putative HGT regions that were greater than or equal to 100bp and did not have any animal hits to the animal database were used in the final analysis.

Manual Annotation Steps

The candidate HGT outputs are then manually curated by the following basic steps: The potential HGT region on the scaffold is analyzed via blastn (NCBI) to the nr/nt database. If this indicates that the region is simply a conserved gene in insects and other metazoan organisms, it is noted as conserved and disregarded. If not, the region is analyzed via blastx to the nr/nt database to determine if it is a conserved gene or remains an HGT candidate. If it remains an HGT candidate, flanking genes are analyzed by both blastn/blastx of the flanking sequence and/or by observations of gene models and transcription models on the species-specific WebApollo/JBrowse. Outputs meeting the criteria of a potential HGT are labeled as an HGT candidate in the comments tab, and supplementary information files are provided with these.

**Table S7. Summary of strongest HGT candidates: Non-GH HGT candidates found in the new pipeline in comparison to the old pipeline, with boundary coverage evidence, bacterial length and identity percent, the Bact/Euk Exp Ratio, which is the e-value score for the best bacterial and best eukaryotic match, which shows the much stronger matches to bacteria.**

| **Sorting Column** | **Location of LGT Candidate** | **Likely Bacterial Source** | **Bacterial Gene Annotation** | **bacterial length & % identity** | **Bact/Euk Exp ratio** | **Are there est models or RNA-seq reads in LGT region?** | **Flanking Eukaryotic *A. glab* gene present on both or one side of LGT? Or is LGT in intron or exon of a *A.glab* gene?** | **Flanking Eukrayotic gene on same contig as LGT Candidate?** | **Does Eukaryotic Flanking gene have RNA-seq support?** | **Sequence Reads Spanning Left Junction (180 + 500 bp insert libraries) counts** | **Sequence Reads Spanning Left Junction (3kb + 8kb insert libraries) counts** | **Sequence Reads Spanning Right Junction (180 + 500 bp insert libraries) counts** | **Sequence Reads Spanning Left Junction (3kb + 8kb insert libraries) counts2** | **3kb and 8kb mate pair inserts spanning left junction (count)** | **3kb and 8kb mate pair inserts spanning right junction (count)** | **Length (bp)** | **GC % - LGT** | **GC % Original Bacteria** | **GC % *A.glab*** |
| --- | --- | --- | --- | --- | --- | --- | --- | --- | --- | --- | --- | --- | --- | --- | --- | --- | --- | --- | --- |
| 12 | Scaffold12:267864-267983 | Wolbachia wRi | Primosomal protein N | 113/119 (95%) | 1E-25 | No | Both | No | Yes | 56 | 17 | 66 | 52 | 6 | 24 | 119 | 35.29 | 35.16 | 32.74 |
| 33 | Scaffold33:1361452-1361740 | Wolbachia wSol | Putative major capsid protein | 125/178 (70%) | 5.47945E-10 | No | In intron | In intron | Yes | 62 | 48 | 67 | 76 | 79 | 89 | 288 | 37.15 | 34.92 | 32.74 |
| 132 | Scaffold132:650680-650845 | Wolbachia wRi | hypothetical protein | 84/88 (95%) | 2E-19 | No | Both | Adjacent | Yes | 35 | 33 | 38 | 27 | 25 | 10 | 165 | 44.24 | 35.16 | 32.74 |
| 156 | Scaffold156:768041-768496 | Wolbachia wPip | Hypothetical protein | 344/482 (71%) | 3.0303E-57 | No | Both | Adjacent | Yes | 66 | 55 | 18 | 60 | 86 | 115 | 455 | 30.11 | 34.19 | 32.74 |
| 269 | Scaffold269:122669-122782 | Rickettsia peacockii str. Rustic | glycosyltransferase | 86/118 (73%) | 0.000322581 | No | Both | No | Yes | 29 | 16 | 35 | 12 | 13 | 9 | 113 | 21.24 | 32.56 | 32.74 |
| 442 | Scaffold442:158778-158912 | Nostoc sp. PCC7107 | unknown | 106/144 (74%) | 8.57143E-08 | No | In intron | In intron | Yes | 59 | 48 | 50 | 41 | 17 | 17 | 134 | 26.87 | 40.36 | 32.74 |
| 570 | Scaffold570:139605-139786 | Clostridium noyvi | Methionine gamma-lyase | 134/185 (72%) | 1.75E-14 | Weak | In exon | In exon | Weak | 85 | 71 | 75 | 41 | 45 | 42 | 181 | 37.02 | 28.86 | 32.74 |
| 624 | Scaffold624:980111-980383 | Calothrix | hypothetical protein | 176/247 (71%) | 0.000000002 | No | Both | Yes | Yes | 65 | 21 | 66 | 11 | 22 | 25 | 272 | 30.51 | 42.25 | 32.74 |

| **LGT Sequence** |
| --- |
| CTCTTGTCACTATATTTCCAAACTATTCCGATCAAGCGTTTTTTGTCAAATGGCACCACTACGTAATCTCCAAGTAAAATCTCAGTATTTTCCTCAATTGCATACGAAAATAACTGATC |
| ACAAGGAAAAAGAGTGTTTGGATCAGAAAGGTCATTCAAAGCGCTTGCGGATGTGACAACTGATTATTGCCATAAGAAATAAACATGCGATAGCATTGAACACTTTGCAATAGAAGGGAAAAAGAGAAAATGTCGGTAAGTATTGCGTCATATTGAGGACAACTTTAGTAGAAAGTTTATGACTGGGATTCATACTTTGATAAATCCGTGTTATTCGATGCATTTATCAATACGCTATGGTTAACATAGCATACAAGAGGTGGGCAGCGCTAAGAAATGACATGAGAT |
| ATCCCAGTGTCAAGCACTGGGATGACAGCATCATAAAGGAACTAGTGTCAGCTACTTGAATGACATCATTCTTTTTCCTGGATCCCAGTGTCAGCTACTCGGATGACACCATTTGTTGTAAACTCACTTTTACTCTATGGTCTTGCCGCTATCCTGAACGGATAC |
| GCAGTTTTATCTACTCTAAATGGTTACAAAACAGTAGATACTATAGAGTTTTGCTGCTGCAGAATGGATAATATGCATAGAGGAAAAGCTACTAGAAAGAGATTCTAAAAAAAACAGTATTAGTTATGCTAAGTGATTAATATTATTTTGATTCTTTTAGAATTAGGTGCGTAGGTCAAGAAATTACGTGCGTTATATAAATAGCTTGACCGTAAAGATTATGCAAACTTTTTACCTTAATTATCAGATCAAATACCAGATCTGTTTGAGGAAACAAAAAAATGTATGATGAATGTTTGGGAGAGTATAATAAAATTTAAAACCTAAGACCAGAGGCAACAAACGATTTCCTCTATTTTTGAAGATTATTCGTTATATGTAGAATAGCACATGTCATTGTGAATATAAATAAACGGGAGGTAACTAAAGGAATATCAGAGAACATATTAAACCAC |
| TTATTTCTTATAACACGAAATAATATTTTATGAATCTACACAGATTAGTAAAAAATAAACCACAGAAAACATCATTTGTTATTACTCTCAAGTAAATTAAACTTTTGGTATTT |
| GACTCATTAATAAACCTCAGACTCATTAATAGAAACTCATAGACTCATTAATAAATCATAGACTCGTTAATAAACTGTAGACTCATTAATGAATACTCGTAGACTCATTAATTAATCATAGACTCATTAATAAA |
| TGTCCGTTAATATATTTCGTTGCCGACTGCATAACTACATCAGCACCAAGTTCTAAAGGTCTTTGTAGGTATGGTGTAAGAAATGTGTTATCCACACCTAACAAAACCTGCTTTTCTTTAGCTACTTGAGCCACTTCGCAAATATCTGTTATTTTCAATGTGGTATTAGAAGGATTCTCAA |
| AAATTTCGAGTTCTAGCACGATATATCGAGTTCTAGCATGAAATTCCGAGTTCTATCATAAAATTTCGAATTCTAGCACGAAATTTCGAGTTTTAACACGAAATTTCGAGTTCTAGCACAAAATTTCGAATTCTAGCATTAAATTTTGAGCTCTAGCACGAAATTTCGAGTTCTAATAATGAAATTTCGAGTTCTAGCTCCAAATTTCAAATTCAAGTATAAAATTTCGAGTTCTAGTATGAAATTTCGAGTTTTAAAACGAAATTTCAAGT |

**Table S8. *Anoplophora glabripennis* Bacterial HGT candidates (not including glycoside hydrolases, which are covered separately; see “Plant cell wall and lignin degradation”).**

Due to its size, this table has been moved to the supplementary excel file entitled “AdditionalFile 2_04Aug2016”.

**II. Digestive Physiology and Specialized Phytophagy**

**II.1 Plant cell wall and lignin degradation**

**Contributors**

**Roy Kirsch, André Busch and Yannick Pauchet** (Max Planck Institute for Chemical Ecology, Department of Entomology)

**Summary**

Wood-boring beetles from the family Cerambycidae have evolved to thrive in a highly challenging environment. They feed exclusively on woody tissues, and to efficiently access the nutrients present in this sub-optimal environment, they have to cope with a recalcitrant barrier called the plant cell wall. The primary cell wall surrounds living plant cells and is composed of the most abundant biopolymers on the planet, namely, cellulose, hemicellulose and pectins. The latter are replaced by lignin in dead plant tissue, forming the secondary wall. All of these polymers, apart from lignin, are polysaccharides. A growing body of evidence indicates that genomes of the Phytophaga [41] contain genes encoding plant cell wall degrading enzymes (PCWDEs) having the ability to hydrolyze and utilize plant cell wall polysaccharides. In particular, in species of cerambycid beetles from the subfamily Lamiinae, several cellulases have been previously identified and functionally characterized [42-46]. Recent surveys of larval gut transcriptomes of *A. glabripennis*, as well as those of another species of Lamiinae (*Apriona japonica*) revealed that these PCWDEs are not encoded by single copy genes, but are part of small to medium-size gene families [47, 48]. Typically, cellulose digestion requires three classes of hydrolytic enzymes: endoglucanases (EC 3.2.1.4), exoglucanases (cellobiohydrolases: EC 3.2.1.74 and 3.2.1.91), and b-glucosidases (cellobiases: EC 3.2.1.21). In addition, pectinases and hemicellulases are necessary for the digestion of the other plant cell wall polysaccharides. All these enzymes are grouped into glycoside hydrolase (GH) families according to their amino-acid sequence similarities and their folding patterns [49].

A total of 86 non-redundant gene models that encoded PCWDEs (Table S9) were annotated. Among these genes, 11 of them encode putative endo- and exoglucanases members of three GH families, namely GH9, GH5 subfamily 2, GH45 and GH48. We observed a large expansion of genes encoding putative β-glucosidases (GH1) with a total of 57 genes (including three pseudogenes) in *A. glabripennis* compared to only 15 found in the model beetle *T. castaneum*, 19 in the genome of *Dendroctonus ponderosae*, and 23 and 28 genes found in the transcriptomes of the two chrysomelid beetles western corn rootworm (*Diabrotica virgifera virgifera* [Chrysomelidae: Galerucinae]) and the flea beetle *Phyllotreta striolata* [50] (Chrysomelidae: Galerucinae), respectively. Regarding non-cellulolytic PCWDEs, a total of 18 genes encoding putative pectin-degrading polygalacturonases were annotated (GH28s). Though hemicellulose is the second most abundant polysaccharide found in woody tissue, genes encoding hemicellulolytic enzymes, such as mannanases and xylanases, were not detected. However, the apparent lack of genes encoding hemicellulolytic enzymes is likely moderated by the presence of a GH5 subfamily 2 enzyme that has evolved the ability to degrade xylan instead of its typical substrate cellulose, which was identified in the lamiine relative of *A. glabripennis,* *Apriona japonica* [51]. Additionally, an enzyme with the ability to degrade birch xylan was previously identified in *A. glabripennis* through zymogram analysis [52]; MALDI-TOF analysis of peptides derived from this protein suggested that it was derived from a gene belonging to GH1, which is a family that is not typically associated with xylanase activity. This finding, along with the discovery of a GH5 enzyme capable of degrading xylan in a close relative of *A. glabripennis*, suggests that PCWDEs may have evolved different functions in the Cerambycidae relative to orthologs in other lineages of beetles. Thus, a full understanding of the contribution of these enzymes to the metabolism of plant cell wall polysaccharides is not likely possible without *in vitro* functional characterization of these enzymes.

**Table S9. Manually curated *Anoplophora glabripennis*** **gene families encoding** **plant cell wall degrading enzymes.** Genes encoding GH9 cellulases are of ancient origin in animals [53]. The other beetle-derived GH families involved in plant cell wall digestion have a more recent origin and were putatively obtained via HGT. GH5 subfamily 2 genes were likely acquired by the LCA of the Phytophaga via HGT from Bacteroidetes [45]. GH45 genes were likely acquired by the LCA of the Phytophaga via HGT from a fungus [54, 55]. Amino acid sequences of beetle GH48 cellulases are similar to bacterial cellobiosidases, but their function(s) remain unclear; they may have evolved to scavenge nitrogen by degrading chitin in the gut or diet, e.g., from host plant tissues containing fungi, or from fungi resident in the gut (e.g., yeasts, *Fusarium solani*) which are thought to concentrate nitrogen and synthesize essential amino acids [56-58]. GH48s are constitutively highly expressed in *A. glabripennis* larvae, and their induction in larvae feeding in a nutrient poor environment (reported herein) is consistent with a putative role in nutrient scavenging. They were most likely acquired by the LCA of the Phytophaga via HGT from a bacterial donor [54, 57]. GH28 genes were likely acquired by the LCA of the Phytophaga via HGT from an ascomycete fungus and subsequently expanded and diversified, but lost in the longhorned beetle subfamily Lamiinae (which includes *A. glabripennis*). After this loss, a GH28 gene was apparently re-acquired by Lamiinae via HGT from a fungal donor [59]. (Also see Table S10).

| **Gene family** | **Putative function** | **Genes total** | **Pseudogenes** |
| --- | --- | --- | --- |
| *Cellulose/hemicellulose degradation* | | | |
| GH9 | Endo-β-1,4-glucanase | 1 | 0 |
| GH45 | Endo-β-1,4-glucanase | 2 | 0 |
| GH5 subfamily 2 | Endo/exo-β-1,4-glucanase | 6 | 0 |
| GH48 | Putative reducing end-acting cellobiohydrolase | 2 | 0 |
| GH1 | β-glucosidase | 57 | 3 |
| *Pectin degradation* | | | |
| GH28 | polygalacturonase | 18 | 0 |

**Table S10. Other manually curated *Anoplophora glabripennis*** **gene families relevant to digestive physiology.**

| **Gene family** | **Putative function** | **Genes total** | **Pseudogenes** |
| --- | --- | --- | --- |
| ***Starch digestion*** | | | |
| GH13 | Alpha-amylase | 3 | 0 |
| GH31 | Alpha-glucosidase | 9 | 1 |
| ***beta-1,3-glucan binding and/or degradation*** | | | |
| GH16 (1) | Beta-1,3-glucanase | 2 | 0 |
| GH16 (2) | Beta-glucan recognition protein (no catalytic activity) | 3 | 0 |
| ***Related to Bt toxins mode of action*** | | | |
| Cadherin-like protein | - | 1 | 0 |
| APN | Alanine aminopeptidase | 16 | 0 |
| ALP | Alkaline phosphatase | 6 | 0 |
| ***Miscellaneous*** | | | |
| FLOT2^1^ | Flotillin 2 | 1 | 0 |
| GMC oxidoreductase^1^ | - | 12 | 0 |

^1^There is a highly conserved cluster of GMC oxidoreductase genes in insects located in the first intron of the FLOT2 gene.

**II.2 Gene expression analysis of plant cell wall degrading enzymes (PCWDEs) in *A. glabripennis* larvae**

**Contributors**

**Erin Scully** (USDA-ARS, Stored Product Insect and Engineering Research Unit, Manhattan, KS)

**Kelli Hoover** (The Pennsylvania State University, Department of Entomology and Center for Chemical Ecology)

**Scott Geib** (USDA-ARS, Pacific Basin Agricultural Research Center)

**Methods**

In brief, five pairs of adult males and females were allowed to maturation feed on fresh twigs collected from Norway maples (*Acer platanoides*) for two weeks. After this period, adult beetles were allowed to mate and oviposit into potted sugar maple trees (*Acer saccharum*) maintained in a USDA-approved quarantine greenhouse at The Pennsylvania State University (University Park, PA) for two weeks. Trees were harvested approximately 60 days after the eggs hatched (indicated by the first appearance of frass) and four third instar larvae were collected. In addition, four third instar larvae feeding on a nutrient rich artificial diet, consisting of 22% cellulose, 9.7% wheat germ, 5.1% torula yeast, 4.0% agar, 2.9% sucrose, 1.7% casein, 0.8% vitamin mix; 0.5% Wesson salt mix; and 0.2% cholesterolKeena MA [60] were also harvested. Larvae were surface sterilized in Coverage Plus followed by two rinses in sterile nuclease free water. Larvae were dissected, midguts were removed and immediately frozen in liquid nitrogen. Tissue was stored at -80 °C. RNA was isolated using the RNA Power Soil Microbiome Isolation Kit (MoBio, Carlsbad, CA). RNA integrity and concentration were verified using the Agilent RNA Nano Assay (Life Technologiest, Carlsbad, CA) and a Nano Drop spectrophotometer (Thermo-Fisher, Rockford, MD). Ribosomal RNA was depleted from the sample using Ribominus Eukaryotic Kit for RNA-Seq (Life Technologies). The enriched mRNA was further polyA purified and multiplexed Illumina libraries were constructed using the TruSeq RNA Sample Prep kit (Illumina, San Diego, CA). Samples were pooled and sequenced on a single Illumina HiSeq lane at the University of Delaware Biotechnology Institute (Newark, DE) to generate approximately 13 million 101 nt paired end reads per sample. Forward reads were trimmed and quality filtered using ea-utils and high quality reads of at least 75 nt in length were mapped to the *A. glabripennis* reference genome assembly using Tophat. Read counts that mapped to each locus (version v0.5.3 annotations) were summed using HTSeq; reads that spanned multiple features were summed using the union mode and reads that did not map uniquely to a single region in the genome were discarded. Differential expression analysis was performed using edgeR. Features with less than 10 mapped reads were removed from the analysis, read counts were normalized by quantile normalization, and variances were estimated using tagwise dispersions. Statistical analysis was performed using a Fisher’s exact test. Features were flagged as differentially expressed if they had a log fold change greater than 1.0 and an adjusted p-value of less than 0.05. Experiment wise false discovery rate (FDR) was estimated at 0.05.

**Results**

Over 3200 differentially expressed genes were identified, including over 1500 genes that were induced by feeding in sugar maples. Supporting their role in plant cell wall metabolism, a diverse profile of genes containing glycoside hydrolase (GH) family domains were induced in insects feeding on sugar maple. Not surprisingly, all of the GH 5 and GH 45 PCWDEs were more highly expressed in larvae feeding in sugar maple (Fig. S4), supporting their roles in processing major constituents of the plant secondary cell wall, namely cellulose, xylan, and xyloglucans. Conversely, the GH 48 cellobiohydrolases and GH 9 endoglucanase were not induced in insects feeding in sugar maple (Fig. S4). While the GH 48 genes were constitutively highly expressed in insects feeding in both sugar maple and artificial diet, the GH 9 endoglucanase was downregulated in the guts of sugar-maple fed *A. glabripennis* larvae. Interestingly, the recombinant GH 9 endoglucanase displayed no activity towards any of the cellulose and hemicellulose substrates assayed and may have evolved to serve a different function in *A. glabripennis*, such as starch digestion. Additionally, over 30 genes containing GH family1 domains were more highly expressed in larvae feeding in wood. Many of these genes had highest scoring BLAST alignments to β-glucosidases and likely convert cellobiose and other oligosaccharides released from the cell wall by hydrolytic enzymes into glucose. However, the amino acid sequences of the GH 1 genes are highly divergent at the amino acid level compared to similar genes harbored in the *Tribolium castaneum* genome indicating that they may have evolved new functions in *A. glabripennis*, including, but not limited to detoxification of plant secondary compounds (e.g. phenolic glycosides) or digestion of other oligosaccharides released from the plant cell wall (e.g., xylose-, mannose-, galactose-, or arabinose-containing oligosaccharides liberated from the hemicellulose matrix). Consistent with the hypothesis of neofunctionalization of subsets of these GH 1 genes in *A. glabripennis*, one of the GH 1 genes located on scaffold 725 that was induced displayed activity towards birch xylan, suggesting that it functions as a xylanase or β-xylosidase [52] and not as a β-glucosidase. The expansion and diversification of this family of genes in *A. glabripennis* relative to other phytophagous insects suggests that it may be correlated with its polyphagous lifestyle.

Twelve different GH 28 polygalacrutonase genes were also highly expressed in wood-feeding larvae, suggesting that pectinous components of primary cell walls may serve as a significant source of sugars for early instar larvae as they feed in immature wood. Several genes containing GH 35 domains were also induced in wood-feeding larvae. These genes had highest scoring BLAST alignments to β-galactosidase and could play roles in processing galactose oligomers released from hemicellulose. Seven genes containing GH 30 domains and two genes containing GH 20 domains were also highly induced in larvae feeding in wood; however, the precise substrate preferences of these enzymes could not be directly inferred from electronic annotation. In insects, GH family 20 enzymes typically serve roles as β-hexosamidases and participate in chitin metabolism, although their substrate preferences have evolved in different taxa [61]. Meanwhile, the GH family 30 enzymes encoded by insects are not as well studied and enzymes assigned to this family can function as β-xylosidases, β-glucuronidases, β-glucosidases, or glucosylceramidases. Five of these GH 30 genes are adjacent to each other on scaffold 504 and may have evolved through tandem duplication. While a subset of these GH 30 genes are expressed in both the larval and adult life stages, two are expressed exclusively in the larval stage (AGLA015835 and AGLA015837) and may be important for digesting components of plant secondary cell walls. Finally, seven GH 18 chitinases were more highly expressed in larvae feeding in live trees. Previously, it was hypothesized that *A. glabripennis* recycles nitrogen by breaking down and re-using chitin deposited in the peritrophic matrix that comprises the midgut or by obtaining nitrogen from the fungal residents of the gut (i.e. yeasts, *Fusarium solani*), which have been hypothesized to concentrate nitrogen and synthesize essential amino acids [56, 58, 61, 62]. The induction of these genes in larvae feeding in a nutrient poor environment is consistent with a putative role in nutrient scavenging.

Several classes of defensive genes were also significantly upregulated in larvae feeding in living sugar maple wood, including 21 cytochrome P450s, 4 UDP-glycuronsyl transferases, and 15 glutathione S-transferases. Clusters of cytochrome P450 genes on scaffolds 29, 81, 83, and 563 were also upregulated. Almost all of the induced cytochrome P450s were derived from clan 3 (17), while two genes belonging to clan 4 and two genes belonging to the mitochondrial clan were also induced. Larval-specific glutathione S-transferases on scaffolds 57 and 1402 and four UDP-glucuronsyltransferases located on several different scaffolds were also induced and may also aid in the detoxification of plant defensive compounds. Three of these genes were from UDP-glucuronsyltransferase families that have undergone lineage specific expansions in *A. glabripennis* compared to *Tribolium*, namely two genes from family 321 and a single gene from family 352. The final upregulated UDP-glucuronosyltransferase gene belonged to family 325. Although only a portion of the potential detoxification enzymes harbored in the *A. glabripennis* genome were induced while feeding in sugar maple, this diverse repertoire likely allows the beetle to colonize other host trees, which likely produce a wide variety of defensive compounds.

**Figure S4 (Fig. 7, main paper). Heatmap of expression levels from GH genes with putative involvement in plant cell wall degradation.** Logfold changes in expression levels in GH genes collected from *A. glabripennis* larvae feeding in the wood of living sugar maple (*Acer saccharum*) trees and a nutrient rich artificial diet. While the expression levels of GH genes were variable, several were significantly upregulated in individuals feeding in the wood of living sugar maple.

**II.3 *In vitro* functional characterization of *A. glabripennis* PCWDEs**

**Contributors**

**Roy Kirsch, André Busch and Yannick Pauchet** (Max Planck Institute for Chemical Ecology, Department of Entomology)

**Materials & Methods**

*Anoplophora glabripennis* samples were kindly provided by David Lance (USDA-APHIS-PPQ, Buzzards Bay, U.S.A). Larvae were chilled on ice and cut open; midguts from 1.5 month old, 4 month old and 8 month old larvae were collected and stored in an excess of RNA Later solution (Ambion) prior to being sent to Germany. RNA was subsequently isolated using the innuPREP RNA Mini Kit (Analytik Jena) according to the manufacturer’s protocol. Genomic DNA contamination was removed by DNAse treatment (TURBO DNAse, Ambion) for 30 min at 37 °C. Midgut RNA was further purified by using the RNeasy MinElute Clean up Kit (Qiagen) following the manufacturer’s protocol and eluted in 20 μl of RNA storage solution (Ambion). Integrity and quality of the RNA samples were determined using the RNA 6000 Nano LabChip kit (Agilent Technologies) on an Agilent 2100 Bioanalyzer (Agilent Technologies) according to the manufacturer’s instructions.

Open reading frames encoding putative plant cell wall degrading enzymes (PCWDEs) were amplified by PCR using gene-specific primers. The forward primer was designed to introduce a 5’ Kozak sequence, and the reverse primer was designed to omit the stop codon. Equal amounts of total RNA prepared from midguts either of 1.5 month old or 4 month old or 8 month old larvae were pooled, and 1 μg total RNA from this pool was used to generate first strand cDNAs using the SMARTer RACE cDNA Amplification Kit (BD Clontech), following the manufacturer’s instructions. These cDNAs were subsequently used as templates for PCR amplifications. PCR products were cloned into the pIB/V5-His TOPO/TA (Invitrogen), in frame with a V5-(His)_6_ epitope at the carboxyl-terminus. The correct sequence and direction of cloning were verified by Sanger sequencing of individual clones. *Sf*9 cells were cultivated in GIBCO Sf-900 II SFM (Invitrogen) on 24-well plates for tissue culture (Falcon; BD) at 27 °C until 70% confluence was achieved. Positive constructs were then transfected in *Sf*9 cells (Invitrogen) using FuGENE HD (Promega) as a transfection reagent. After 72 h, the culture medium was harvested, and successful expression was verified by Western blot using the anti-V5-HRP antibody (Invitrogen). Alternatively, and in order to collect enough material for downstream enzymatic activity assays, a single clone per construct was chosen to be transiently transfected in a 6-well plate format. After 72 h, culture medium was harvested and centrifuged (16,000 x*g*, 5 min, 4 °C) to remove cell debris and stored at 4 °C until used. Again, successful expression was verified by Western blot using the anti-V5-HRP antibody.

Agarose diffusion assays

To test the substrate specificity of each of the PCWDEs expressed in insect cells, diffusion assays were performed using 1 % agarose Petri dishes in McIlvaine buffer (pH 5.0) containing one of the following substrates: 0.1 % carboxymethylcellulose (CMC, Sigma-Aldrich); 0.1 % beechwood xylan (Sigma-Aldrich); 0.1 % xyloglucan from Tamarind seeds (Megazyme); 0.1 % pectin from citrus peels (Sigma-Aldrich); 0.1 % demethylated polygalacturonic acid (Megazyme). Two millimeter holes were made in the agarose, and 10 μl of crude enzyme extracts were deposited into each hole. Activity was revealed, after 24 h of incubation at 40 °C, with 0.1 % Congo Red solution (for CMC, beechwood xylan and xyloglucan) or with 0.1 % Ruthenium red solution (for citruspectin and demethylatyed polygalacturonic acid); each plate was then destained with 1 M NaCl or distilled water (for ruthenium red) until pale activity zones appeared against a dark red background.

TLC analysis of hydrolysis reaction products

The culture medium of transiently transfected cells was first dialyzed against distilled water at 4 °C for 48 h, using Slide-A-Lyzer Dialysis Cassettes with a 10 kDa cut off, before being desalted with Zeba Desalt Spin Columns 7 kDa cut off (both Thermo Scientific), according to the manufacturer´s instructions. Samples were stored at 4 °C until used. Enzyme assays (20 ul) were set up using 14 µl of dialyzed and desalted crude enzyme extracts mixed with 4 µl of a 1 % substrate in solution in a 20 mM McIlvaine buffer (pH 5.0). For GH5-1 to -6, the following substrates were tested: CMC, avicel (suspension), beechwood xylan and xyloglucan. For GH28s, the following substrates were tested: demethylated polygalacturonic acid and pectin from citrus peels. The activity of GH28s on 10 µg/µl aqueous solution of tri- and di-galacturonic acid was also tested. Enzyme assays were incubated for 16 h at 40 °C and applied to TLC plates (silica gel 60, 20x20 cm, Merck) afterwards. Plates were developed with 1-butanol/glacial acetic acid/water (2:1:1) for 150 min and were dried before developing them a second time. Assays containing pectic substrates were developed with ethyl acetate/acetic acid/formic acid/water (9:3:1:4) as mobile phase. Carbohydrates were then stained by quickly soaking the plates in 0.2 % (w/v) orcinol in methanol/sulfuric acid (9:1), followed by a short heating until spots appeared on the plates. The reference standard contained 2 µg each of glucose, cellobiose, cellotriose, cellotetraose, cellopentaose (all from Sigma-Aldrich) and cellohexaose (Megazyme) or 2 µg each of xylose (Sigma), xylobiose and xylotriose (both Megazyme) or 2 µg of mono-, di- and tri-galacturonic acid (all from Santa Cruz Biotechnology).

Phylogenetic analyses

Amino acid alignments were carried out using MUSCLE version 3.7 on the Phylogeny.fr web platform (<http://www.phylogeny.fr>) [63] and were inspected and corrected manually when needed. Bayesian-inferred analyses were carried out in MrBayes 3.1.2 [64], which was set for the amino acid models to mix, thereby allowing model jumping between fixed-rate amino acid models to select the best one, and an estimated gamma distribution of rates of evolution. Markov chain Monte Carlo runs were carried out for 400,000 generations after which the average standard deviation of split frequencies was found inferior to 0.01, sampling every 100th generation and using a burn-in rate of 25 %. Two runs were conducted for the dataset showing agreement in topology and likelihood scores. To obtain support from a second independent method, maximum likelihood analyses were also performed and were conducted in MEGA5 [65]. The best model of protein evolution was determined in MEGA5 using the ‘find best DNA/protein models’ tool. In all cases, the best model was the Whelan and Goldman (WAG) model, incorporating a discrete gamma distribution (shape parameter = 5) to model evolutionary rate differences among sites (+G) and a proportion of invariable sites (+I). The robustness of each analysis was tested using 1,000 bootstrap replicates.

**Results and Discussion**

Hydrolysis of polygalacturonic acid polymers and oligomers by *A. glabripennis* putative polygalacturonases

Out of the 18 GH28 genes present in the *A. glabripennis* genome, only three (GH28-6, -7 and -12) could not be amplified by PCR using cDNAs prepared from larval midgut RNA as template (Table S11), and thus could not be subsequently cloned in the plasmid for expression in insect cells (Fig. S5). The remaining 15 GH28s were transiently expressed in *Sf*9 cells with a carboxyl-terminal tag allowing their detection by Western blot. Heterologous expression succeeded for all GH28 proteins apart from GH28-4, and they were found to be secreted in the culture medium after Western blot, as predicted by the presence of an amino-terminal signal peptide (Fig. S6A). The variation observed in the apparent molecular weight of these GH28 proteins is most likely due to differences in their degree of N-glycosylation, ranging between zero for GH28-8 and -13 and up to seven for GH28-5 as predicted by the NetNGlyc 1.0 Server (http://www.cbs.dtu.dk/services/NetNGlyc/). According to an amino acid alignment of the cerambycid-derived GH28s identified here (Fig. S7); all of the *A. glabripennis* GH28s proteins present no amino acid substitutions at crucial positions including the catalytic and substrate binding residues, as well as the typical three disulfide bridges. Therefore, our prediction is that the 14 GH28 proteins we managed to heterologously express in *Sf*9 cells should all be active polygalacturonases (EC 3.2.1.15) acting on homogalacturonan substrates.

To gain insight into the potential activity of these 14 predicted polygalacturonases, we first performed agarose diffusion assays using two types of homogalacturonan polymers, either a demethylated form of polygalacturonic acid or citrus pectin for which the degree of methylation has been estimated to be between 50 and 70 % [66, 67] (Fig. S6B) Typically, enzymatic activity detected by this kind of assay (presence of a halozone in a dark purple background) is due to the action of endopolygalacturonases [68]. Our first observations revealed that a majority of the GH28 proteins tested were active against at least one of the two substrates tested. A major exception comes from a group of phylogenetically related GH28s (GH28-1, -2, -3 and -5), for which the corresponding genes are located in tandem on scaffold 341), that show no activity on both substrates by agarose diffusion assay (Fig. S6B). Interestingly, this latter group of GH28s clusters together with two previously characterized GH28 proteins from the lamiine *Ap. japonica*. One of these two proteins (*Ap. japonica* GH28-1) was characterized as being an exopolygalacturonase [47]. To test whether *A. glabripennis* GH28-1, -2, -3 and -5 could also be exopolygalacturonases, we incubated these heterologously expressed proteins with the same two substrates and assessed their activity using thin layer chromatography (Fig. S6C). Using both substrates, we observed an accumulation of monomer of galacturonic acid for the latter four enzymes, indicating their exopolygalacturonase activity. Subsequently, we also tested the activity of GH28-1, -2, -3 and -5 against oligomers of galacturonic acid, namely trigalacturonic and digalacturonic acid. GH28-1 and -2 were both able to completely degrade both the trimer and the dimer of galacturonic acid, whereas GH28-3 and -5 could only act on the trimer and not the dimer (Fig. S6C). In parallel, we also tested an extra two GH28 proteins by thin layer chromatography, GH28-11 and GH28-17. GH28-11 is the only polygalacturonase expressed both in larvae and adults [47]. This enzyme showed similar activity against both demethylated polygalacturonic acid and citrus pectin using agarose diffusion assays. Our thin layer chromatography analyses revealed that GH28-11 not only released dimers, trimers and longer oligomers from polygalacturonic acid, a typical pattern for endopolygalacturonases such as GH28-17, but we also observed an accumulation of monomers of galacturonic acid for GH28-11 (Fig. S6C).

Overall, the repertoire of GH28 proteins encoded by the *A. glabripennis* genome, composed of both endo- and exo-polygalacturonases able to act on substrates with various degrees of methylation, seems highly complementary and would allow this beetle to efficiently decompose homogalacturonan polymers surrounding living plant cells present in wood tissues.

Hydrolysis of cellulose and hemicellulose polymers by enzymes from glycoside hydrolase families 5 and 45 in *A. glabripennis*

A number of endo- and exo-acting cellulases have been previously characterized in cerambycids and are members of two different glycoside hydrolase families, GH5 subfamily 2 and GH45 [42, 44-47, 69]. As mentioned above, a screen of the *A. glabripennis* genome revealed the presence of six genes encoding GH5 subfamily 2 putative cellulases as well as two genes encoding putative GH45 cellulases. An additional gene encoding a putative cellulase part of a third family, GH9, was also retrieved during this genome screen (Table S11). This gene is a homolog of one in *T. castaneum* (EFA05721) encoding for an endo-β-1,4-glucanase of the GH9 family [70].

To obtain further insights into the substrate specificity of these nine putative cellulase genes, we transiently expressed them in *Sf*9 cells in frame with a tag facilitating their detection by Western blot. Their expression was monitored 72 hours post-transfection by Western blot; the six GH5 subfamily 2 proteins as well as both GH45s and the GH9 were successfully expressed and secreted in the culture medium of transfected cells (Fig. S8A). To assess the enzymatic activity of these nine proteins, we first chose to use typical substrates of cellulases and hemicellulases, namely several forms of cellulose (a major constituent of both primary and secondary plant cell walls) such as carboxymethyl cellulose, Avicel (a crystalline form of cellulose) and filter paper, xyloglucan (a major constituent of the hemicellulose network of the primary cell wall) and beechwood xylan (a major constituent of the hemicellulose network of the secondary cell wall) [71, 72]. We first performed agarose diffusion assays with the water soluble substrates (beechwood xylan , carboxymethylcellulose (CMC) and xyloglucan). Such assays, after Congo Red staining, are well known to favor the recognition of endo-acting enzymes, because they quickly reduce the viscosity of the polysaccharide used as substrate thus allowing the degradation products from the gel matrix to diffuse rapidly [73]. Under these conditions, a single halozone corresponding to GH5-1 could be observed on the xylan plate, indicating that this enzyme is an endo-β-1,4-xylanase (EC 3.2.1.8) (Fig. S8B). When CMC was used as substrate, four halozones appeared after staining with Congo Red which corresponded to GH5-2, GH5-5 GH45-1 and GH45-2, identifying them as being endo-β-1,4-glucanases (EC 3.2.1.4) (Fig. S8B). Finally, a single halozone was observed on the xyloglucan plate corresponding to GH5-2 reflecting its xyloglucan endo-β-1,4-glucanase activity (EC 3.2.1.151). Taking into account that GH5-2 could also hydrolyze CMC, this enzyme possesses the interesting ability to endohydrolyse the 1,4-β-D-glucosidic linkages in both CMC and xyloglucan (Fig. S8B). Four out of the nine putative cellulases, namely GH5-3, GH5-4, GH5-6 and GH9, did not harbor any enzymatic activity against the three substrates tested using agarose diffusion assays, indicating that these four proteins are at least not endo-acting enzymes. Altogether, our data indicate that the GH5 subfamily 2 in *A. glabripennis* seems to have evolved in such a way that its members diversified to be able to degrade all the major constituents of the cellulose and hemicellulose networks of both primary and secondary plant cell walls.

Taking these facts into account and to further our understanding about the substrate specificity of the GH5 subfamily 2 proteins, we investigated how these enzymes degrade their substrate. To do so, we performed activity assays in tubes that were subsequently analyzed by thin-layer chromatography (Fig. S8C). As predicted by our agarose diffusion assay analyses, when beechwood xylan was used as substrate, a strong activity was revealed only for GH5-1 with an accumulation of xylobiose, xylotriose, xylotetraose and xylopentaose as well as a strong smear of larger oligomers (Fig. S8C). Degradation products of CMC could be detected for GH5-2 and for GH5-5, thus confirming the activity observed after agarose diffusion assays (Fig. S8C). The pattern of degradation products produced by GH5-2 and GH5-5 is slightly different. An accumulation of cellotetraose and cellotriose and a weak signal for cellobiose could be observed for GH5-2. For GH5-5, the pattern of degradation products of CMC showed an accumulation of cellotriose and cellobiose and a strong smear of larger degradation products typical of endo-acting enzymes (Fig. S8C). Unexpectedly, we also observed an accumulation of glucose and cellobiose for GH5-6 after incubation with CMC; no halozone could be previously observed after agarose diffusion assays for this enzyme, classifying it as an exo- β-1,4-glucanase. To determine whether this GH5-6 also possessed the ability to degrade crystalline cellulose, we performed the same assay but using Avicel and filter paper instead of CMC as substrate, but no degradation products could be observed (data not shown). Finally and as expected after our agarose diffusion assays, using xyloglucan as substrate, degradation products could only be observed for GH5-2 with an accumulation of relatively long oligomers of glucose (hexamers, heptamers and octamers).

Although the *A. glabripennis* genome encodes for 3 gene families of putative cellulases (GH5 subfamily 2, GH9 and GH45), a single of these, the GH5 subfamily 2, evolved in such a way that it provides the beetle with an arsenal of enzymes possessing the ability to degrade the main polysaccharides of the cellulose and hemicellulose networks in both primary and secondary plant cell walls (Fig. S9).

**Author contributions**

R.K. and A.B. designed and performed experiments; Y.P. designed experiments and wrote the paper.

**Acknowledgement:** We are grateful to Bianca Wurlitzer and Domenica Schnabelrauch for technical support This work was supported by the Max-Planck-Gesellschaft.

**Figure S5**. **Phylogram showing Lamiinae GH28 proteins with information on substrate specificity**.

A Bayesian-inferred phylogeny is shown which compares the predicted amino acid sequences of the 18 GH28s found in the *Anoplophora glabripennis* (AGL) genome with those described for *Apriona japonica* (AJA). Posterior probability support values are indicated at corresponding nodes followed by bootstrap values (1000 replicates) derived from a maximum-likelihood analysis. A GH28 sequence derived from the yeast *Saccharomyces kudriavzevii* (SKU1, EJT42295.1) was used as an outgroup, reflecting the origin of these genes via HGT from fungi [59]. The first column (PCR) refers to whether the corresponding open reading frame could have been PCR-amplified from larval gut cDNAs and cloned into the pIB/V5-His TOPO-TA vector for heterologous expression in *Sf*9 cells. The second column (Expression) refers to whether the corresponding proteins could have been successfully expressed after transfection in Sf9 cells (Fig. S6A). The third column (Plate assays) summarized the enzymatic activity observed for each heterologously expressed GH28 protein after agarose diffusion assays (Fig. S6B). The fourth column summarized the enzymatic activity observed for the GH28 proteins which did not show activity after agarose diffusion assays, and two (GH28-11 and -17) which did show activity after agarose diffusion assays. Substrate details are as follows: 1: demethylated polygalacturonic acid derived from citrus pectin; 2: citrus pectin; 3: trigalacturonic acid; 4: digalacturonic acid. n.t.: not tested.


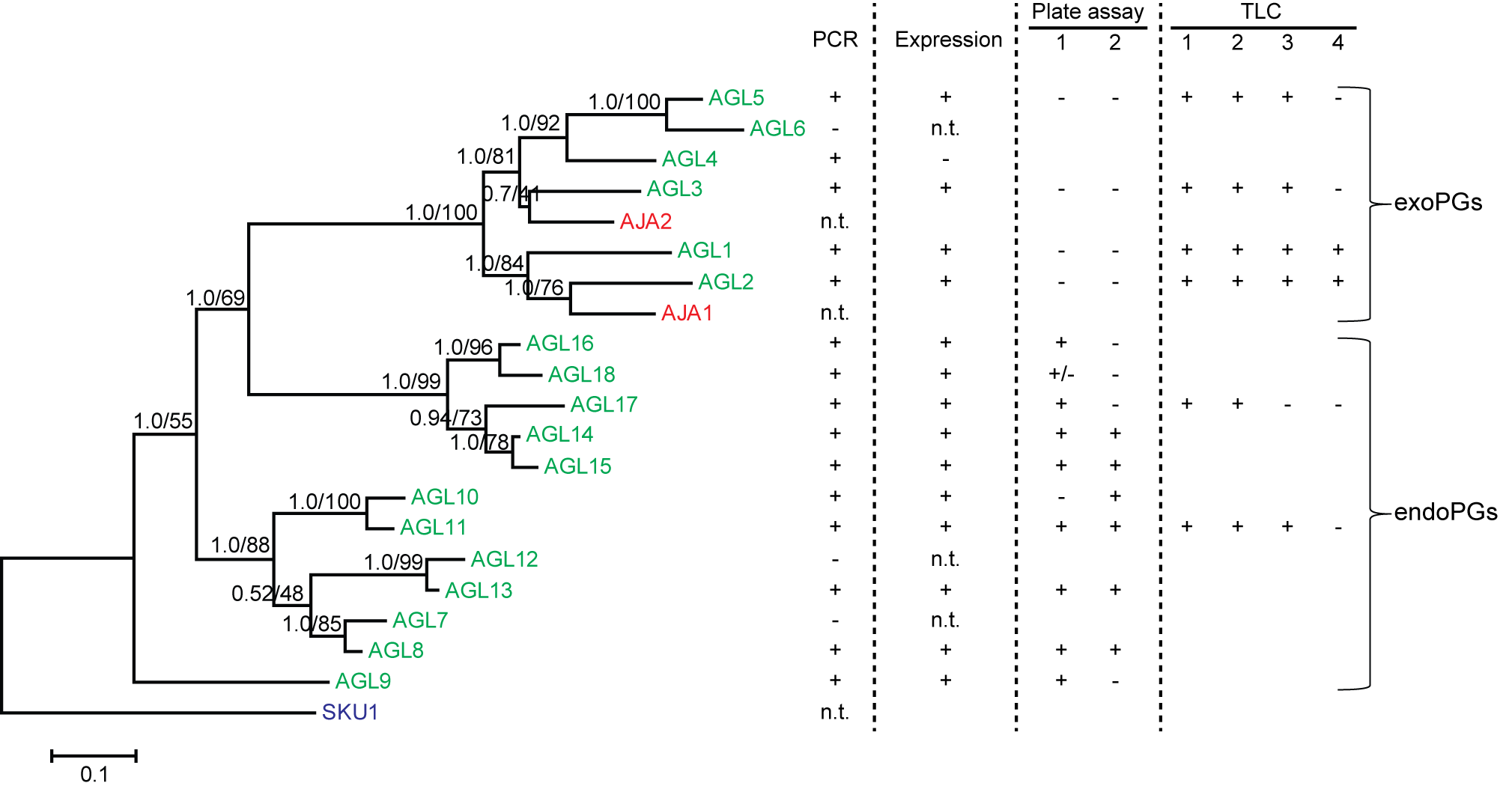


**Figure S6.** **Heterologous expression and substrate specificity of *Anoplophora glabripennis* GH28s.**

**(A)** *Sf*9 insect cells were transfected with two clones (except for GH28-10 for which only a single clone could have been tested) of each of the 15 successfully PCR amplified GH28 ORFs found in the *A. glabripennis* genome, cloned into the pIB/V5-His TOPO-TA vector. The presence of the corresponding heterologously expressed proteins was verified, 72 h post-transfection, by Western blot using an antibody directed against the V5 epitope. Molecular weight markers in kilodaltons are indicated next to each Western blot.

**(B)** In parallel, culture media from each transfection were deposited on an agarose plate containing either 0.1% demethylated polygalacturonic acid prepared from citrus pectin or crude citrus pectin (DM 50-70% based on literature) in McIlvaine buffer pH 5.0 and incubated for 24 h at 40 °C. Halo zones reflecting endopolygalacturonase activity were revealed after staining with ruthenium red.

**(C)** Thin-layer chromatography (TLC) analyses of the hydrolysis end products of demethylated polygalacturonic acid prepared from citrus pectin, crude citrus pectin, as well as trimers and dimers of galacturonic acid by the *A. glabripennis* GH28s found to be negative after agarose diffusion assays. Two endopolygalacturonases, GH28-11 and -17, were also included. Crude enzyme extracts derived from the culture media of transfected *Sf*9 cells were incubated with the substrates tested in McIlvaine buffer pH 5.0 at 40 °C for 16 h before being spotted on TLC plates. Three negative controls were included as followed: -C1: demethylated polygalacturonic acid incubated with GH28-1 previously boiled for 10 min; -C2: demethylated polygalacturonic acid incubated with GH28-17 previously boiled for 10 min; -C3: demethylated polygalacturonic acid was incubated without enzyme. The standards (STD) used were: MGA: monogalacturonic acid; DGA: digalacturonic acid and TGA: trigalacturonic acid.


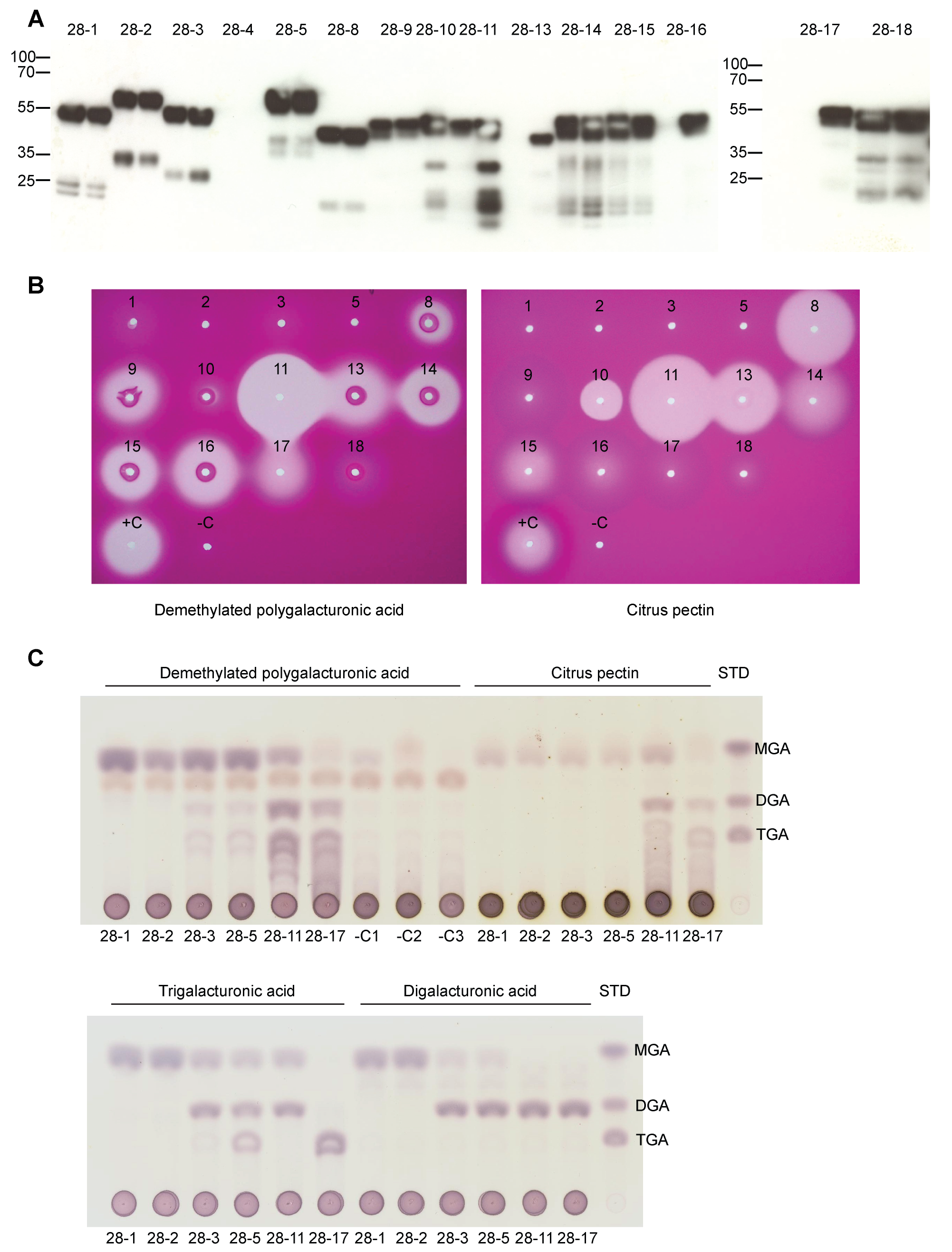


**Figure S7.** **Amino acid alignment of *Anoplophora glabripennis* GH28 proteins together with two previously characterized ones from *Apriona japonica*.**

The predicted signal peptide was removed from each protein sequence prior to alignment. The degree of amino acid conservation is indicated by purple shadings. The three catalytic aspartic acid residues are indicated by red arrows. The three disulfide bridges conserved between cerambycids and yeast GH28s are shown with a bracket. Asterisks signal the positions of amino acids that are highly conserved and previously shown to be functionally important residues between all characterized eukaryotic and prokaryotic actives PGs. A GH28 sequence derived from the yeast *Saccharomyces kudriavzevii* (SKU1, EJT42295.1) used as an outgroup for the phylogenetic analysis presented in Fig. S5 was also included in the alignment.


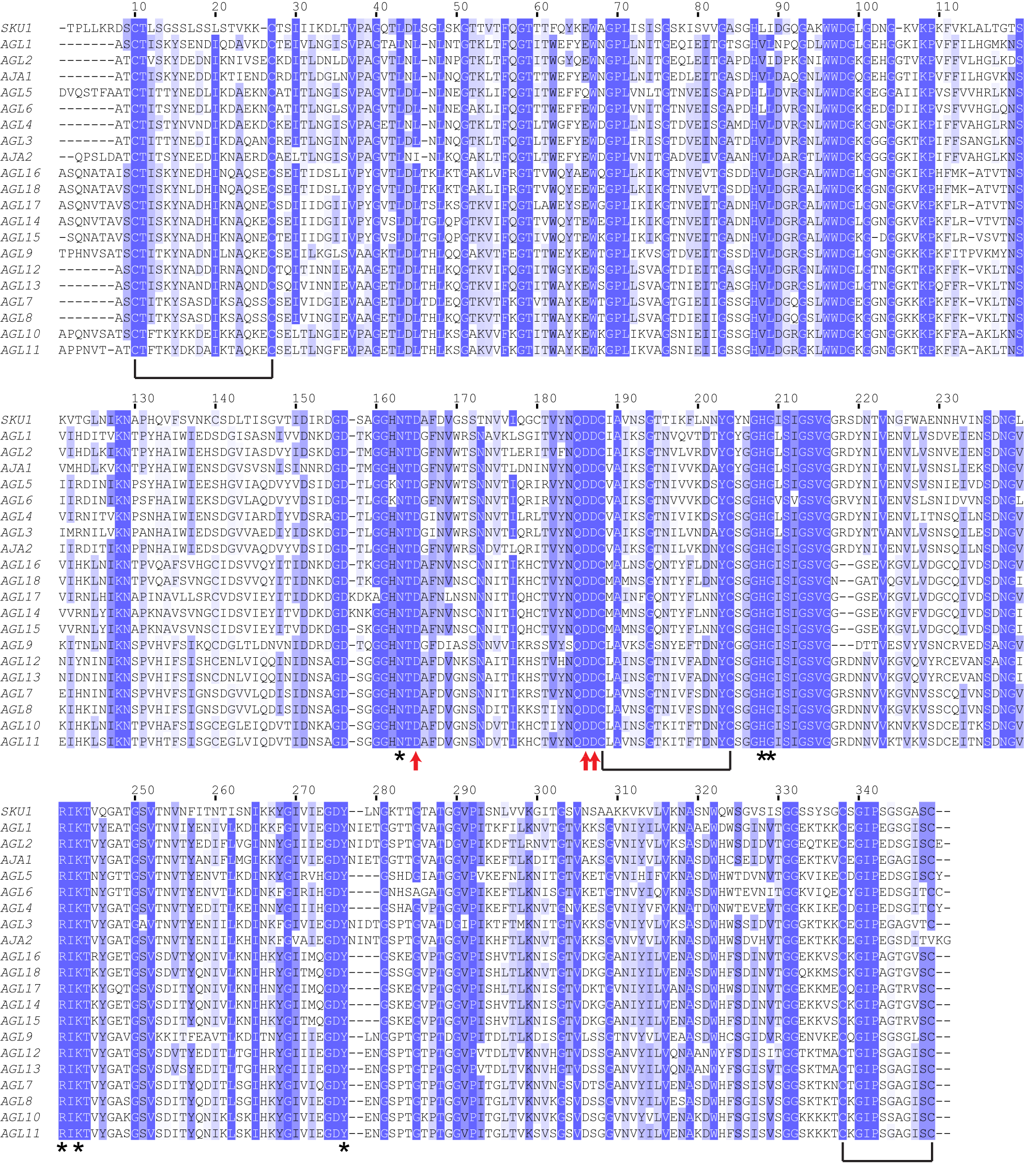


**Figure S8.** **Heterologous expression and substrate specificity of *A. glabripennis* putative cellulases.**
**(A)** *Sf*9 insect cells were transfected with two clones (except for GH5-6 for which only a single clone could have been tested) of each of the nine successfully PCR amplified putative cellulase ORFs found in the *A. glabripennis* genome (six GH5 subfamily 2; two GH45; a single GH9), cloned into the pIB/V5-His TOPO-TA vector. The presence of the corresponding heterologously expressed proteins was verified, 72 h post-transfection, by Western blot using an antibody directed against the V5 epitope. Molecular weight markers in kilodaltons are indicated next to each Western blot.

**(B)** In parallel, culture media from each transfection, corresponding to a positively expressed putative cellulase, were deposited on an agarose plate containing either 0.1% beechwood xylan or carboxymethylcellulose or tamarind seed xyloglucan in McIlvaine buffer pH 5.0 and incubated for 24 h at 40 °C. Halozones reflecting enzymatic activity were revealed after staining with Congo Red.

**(C)** Thin-layer chromatography (TLC) analyses of the hydrolysis end products of beechwood xylan, carboxymethylcellulose and tamarind seed xyloglucan produced by the six GH5 subfamily 2 and the GH9. Crude enzyme extracts derived from the culture media of transfected *Sf*9 cells were incubated with the substrates tested in McIlvaine buffer pH 5.0 at 40 °C for 16 h before being spotted on TLC plates. A negative control was included where the corresponding substrates were incubated without enzyme. The standards used for the carboxymethylcellulose and xyloglucan TLC were: G1: glucose; G2: cellobiose; G3: cellotriose; G4: cellotetraose; G5: cellopentaose and G6:cellohexaose. The standards used for the xylan TLC were: X1: xylose; X2: xylobiose and X3: xylotriose.


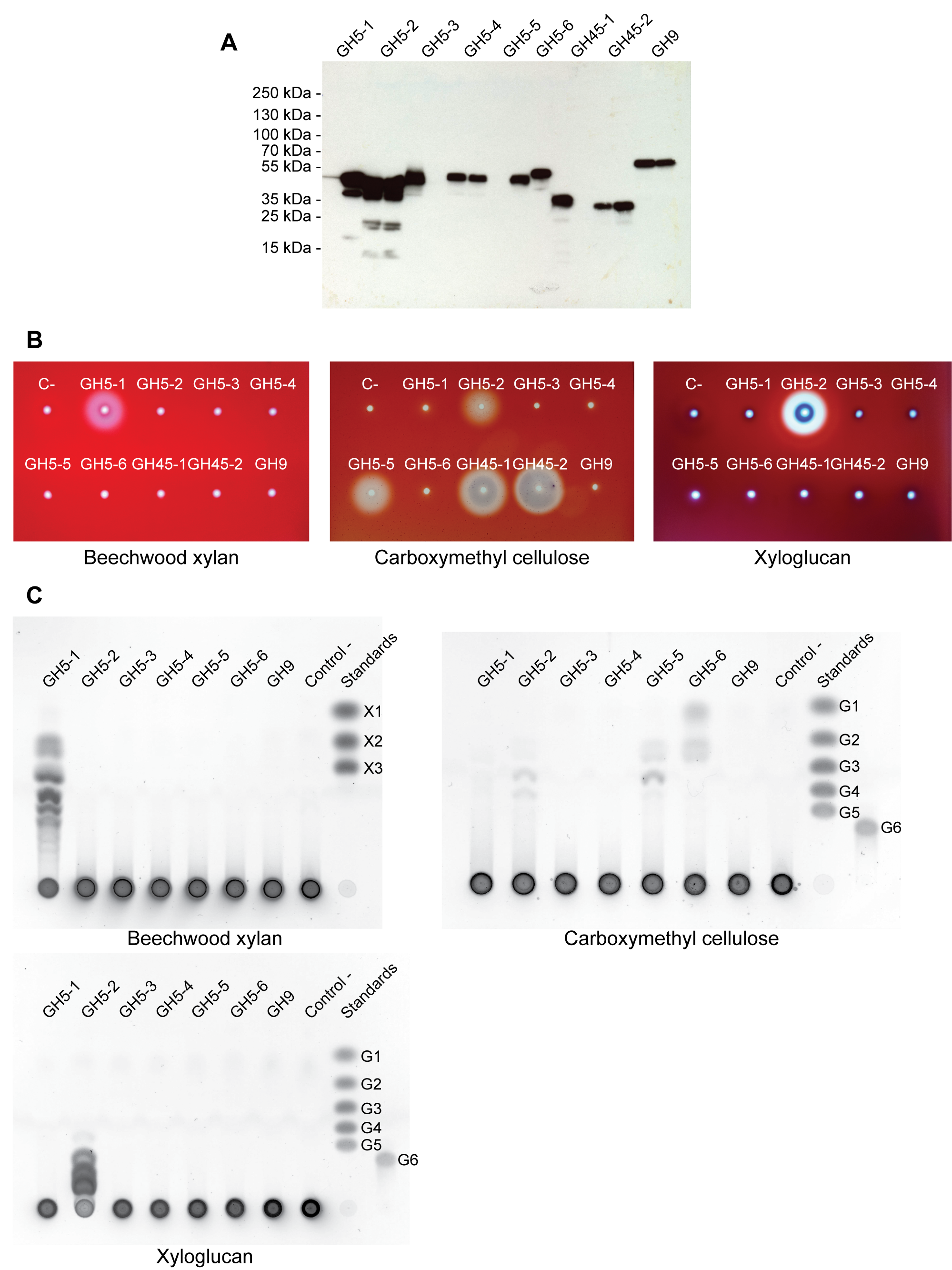


**Figure S9.** **Phylogenetic relationships among cerambycid-derived GH5s with information on their enzymatic properties (substrate specificity).**

A Bayesian-inferred phylogeny is shown that compares the predicted amino acid sequences of the six GH5 subfamily 2 proteins found in the *Anoplophora glabripennis* (AGL) draft genome with those described for *Apriona japonica* (AJA) (both Cerambycidae: Lamiinae). Posterior probability support values are indicated at corresponding nodes followed by Bootstrap values (1000 replicates) derived from a maximum-likelihood analysis. A GH5 sequence derived from the bacterium *Zobellia galactanivorans* (ZGA, G0L1Q5) was used as an outgroup, reflecting the assumed origin of these genes via HGT from bacteria (no comprehensive phylogenetic analysis has been performed so far). The AJA and the AGL sequences are indicated in red and in green, respectively. Other cerambycid-derived sequences (all from subfamily Lamiinae) are indicated in black. AGE: *Apriona germari*; PHI: *Psacothea hilaris*; OAL: *Oncideres albomarginata chamela*; ACH: *Anoplophora chinensis*. Next to the phylogenetic tree is indicated the experimentally determined enzymatic classification for each protein as well as the enzymatic classification code. * AGL2 (GH5-2) is a bifunctional endo-β-1,4 glucanase (EC 3.2.1.4) / endo-β-1,4-xyloglucanase (EC 3.2.1.151).


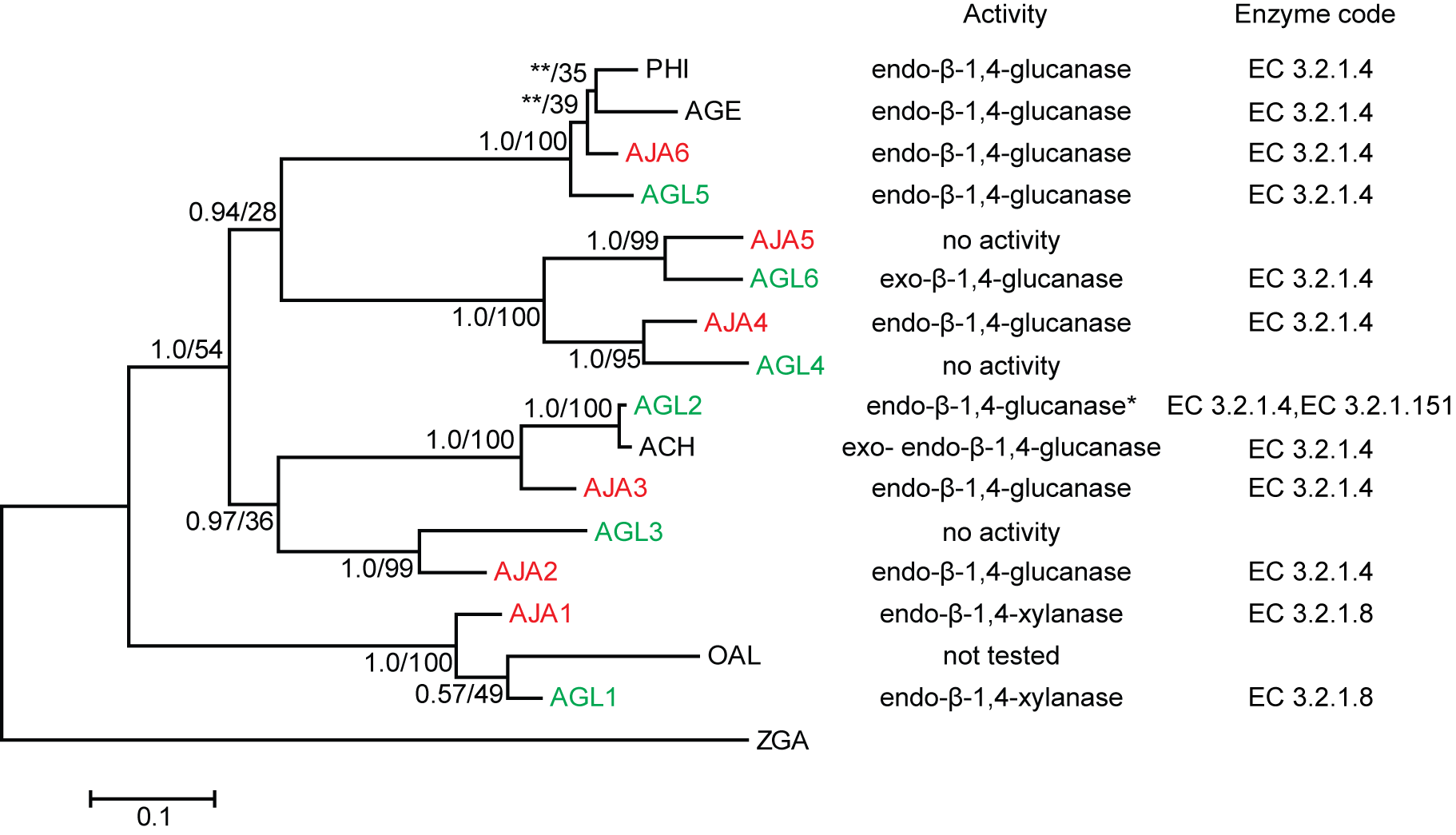


**Table S11. Summary of the gene encoding for PCWDEs for which heterologous expression in insect *Sf*9 cells has been attempted.**

| **Gene symbol** | **Gene ID** | **Expression status** |
| --- | --- | --- |
| *Cellulolytic/Hemicellulolytic* | | |
| GH9 | AGLA010313-RA | successful |
| GH5-1 | AGLA002353-RA | successful |
| GH5-2 | AGLA002352-RA | successful |
| GH5-3 | AGLA002354-RA | successful |
| GH5-4 | AGLA002351-RA | successful |
| GH5-5 | AGLA006972-RA | successful |
| GH5-6 | AGLA016376-RA | successful |
| GH45-1 | AGLA005419-RA | successful |
| GH45-2 | AGLA005420-RA | successful |
| *Pectinolytic* | | |
| GH28-1 | AGLA010095-RA | successful |
| GH28-2 | AGLA010096-RA | successful |
| GH28-3 | AGLA010097-RA | successful |
| GH28-4 | AGLA010098-RA | unsuccessful |
| GH28-5 | AGLA010099-RA | successful |
| GH28-6 | AGLA010101-RA | unsuccessful |
| GH28-7 | AGLA021349-RA | unsuccessful |
| GH28-8 | AGLA018064-RA | successful |
| GH28-9 | AGLA006364-RA | successful |
| GH28-10 | AGLA002349-RA | successful |
| GH28-11 | AGLA002350-RA | successful |
| GH28-12 | AGLA012060-RA | unsuccessful |
| GH28-13 | AGLA012061-RA | successful |
| GH28-14 | AGLA025221-RA | successful |
| GH28-15 | AGLA025195-RA | successful |
| GH28-16 | AGLA025082-RA | successful |
| GH28-17 | AGLA025090-RA | successful |
| GH26-18 | AGLA025220-RA | successful |

**II.4 Horizontal gene transfer and PCWDEs**

**Contributor**

**Yannick Pauchet** (Max Planck Institute for Chemical Ecology, Department of Entomology)

**Summary:**

It used to be believed that most animals (Metazoa) do not possess endogenous enzymes enabling them to degrade plant cell wall polysaccharides and that they rely instead on their symbiotic microorganisms for their digestion. It is since recently clear that genomes of the Phytophaga group of beetles, which encompasses the two superfamilies Chrysomeloidea (Chrysomelidae and Cerambycidae) and Curculionoidea [41], contain genes encoding plant cell wall degrading enzymes (PCWDEs) enabling the degradation of plant cell wall polysaccharides such as pectin, hemicellulose and cellulose. In *Anoplophora glabripennis*, these enzymes are distributed in several glycoside hydrolase (GH) families according to the Carbohydrate-active enzyme nomenclature [49]. Our analyses revealed that in *A. glabripennis*, cellulolytic and hemicellulolytic enzymes are distributed in the families GH9, GH5 subfamily 2, GH45 and GH48 whereas pectolytic enzymes of a single gene family (GH28) can be found in this insect species.

The movement of genetic information, other than by sexual means from parents to their offspring is defined as horizontal gene transfer (HGT). Whereas the genes encoding the GH9 family of cellulases are of ancient origin in animals [53], the origins of the other beetle-derived PCWDE-encoding genes, including those of *A. glabripennis*, have recently attracted a lot of attention. The distribution of cellulases from GH5 subfamily 2 is extremely patchy in eukaryotes. To date the presence of these genes has only been described in cerambycid beetles, plant parasitic nematodes, and symbiotic protists of termites and wood-feeding cockroaches [47, 74-76]. Recent analyses suggest that genes encoding members of GH5 subfamily 2 have been acquired by plant parasitic nematodes and cerambycid beetles through horizontal gene transfer (HGT) from bacteria of the Bacteroidetes [45, 74, 77]. Similarly, the distribution of genes encoding GH45 cellulases is also patchy in animals. To date, the presence of these genes was only described in beetles of the Phytophaga, and in protists of termites and molluscs [78-80]. Together with the high degree of similarity between beetle-derived and fungal GH45s, this strongly suggests that these genes were also originally acquired by beetles through HGT from a fungal source [54, 55, 81] and that this HGT event most likely took place in the last common ancestor of beetles from the two superfamilies Chrysomeloidea and Curculionoidea [48]. Putative cellobiosidases from the GH48 family are commonly found in bacteria, but have only been described in beetles of the Phytophaga and in only three fungi [79, 82, 83]. The GH48s found in species of the Chrysomeloidea and Curculionoidea possess a common ancestor and, similar to the GH45s, were most likely acquired from a bacterial donor through HGT in the last common ancestor of the Phytophaga [48, 54]. The function of these GH48 proteins in beetles remains unclear; they share high similarities with bacterial cellobiosidases, but one study suggests that these enzymes evolved to degrade chitin rather than cellulose and are important in diapause termination in a leaf beetle [84].

A total of 18 genes encoding pectolytic enzymes of the GH28 family could be annotated from the *A. glabripennis* genome, with 12 of them being actively transcribed in the larval midgut [47]. A functional characterization of the products of these genes showed that these GH28s evolved to act on polygalacturonic acid either as exo-acting or endo-acting enzymes. GH28-encoding genes are commonly distributed in species of the Chrysomeloidea and Curculionoidea, including at least one other longhorned beetle [47, 59, 79]. Our recent analysis of the evolutionary history of genes encoding members of the GH28 family in phytophagous beetles revealed that the GH28 genes found in Chrysomelidae and the ones we identified in longhorned beetles originated from two independent HGT events [59]. A first HGT event occurred about 200 Mya in a common ancestor of beetles of the Phytophaga from an ascomycete fungus of the Pezizomycotina. According to our analysis of the *A. glabripennis* genome and of the *Apriona japonica* larval gut transcriptome, this GH28 gene was subsequently lost in cerambycids in at least the subfamily Lamiinae, but expanded and diversified in Chrysomelidae and Curculionidae. After the initial loss, a GH28 gene in Lamiinae was re-acquired through another HGT event, this time from another fungal donor member of a clade containing both species of Pezizomycotina and Saccharomycotina [59]. It is important to note that due to the paucity of genomic and transcriptomic data for the family Cerambycidae (restricted only to species of the subfamily Lamiinae), we do not know at this stage whether the loss of the initial GH28 gene and the re-acquisition of a different gene from another donor holds true for all Cerambycidae or only for the Lamiinae.

**III. Detoxification and Polyphagy**

**III.1 Cytochrome P450s**

**Contributors**

**David R. Nelson** (University of Tennessee, Department of Microbiology, Immunology and Biochemistry)

**Stephanie Haddad (**University of Memphis, Department of Biological Sciences)

**Summary**

*Anoplophora glabripennis* has the four-clan pattern seen in all insects, with CYP2, CYP3, CYP4, and mitochondrial clans (Fig. S10). In addition, *A. glabripennis* has one outlier sequence, CYP413B1, that has no conserved cys at the heme signature region. This family is also seen in the Colorado potato beetle (*Leptinotarsa decemlineata*). There is now a small but growing collection of insect sequences that is lacking the highly conserved heme iron ligand. *Tribolium castaneum* has CYP348A1, which clusters with CYP413B1 as an outgroup of the CYP3 clan. The CYP408 family is a third example seen in leafhoppers and locusts. These unusual sequences probably do not represent a novel clan, but just highly diverged CYP3 clan members. The lack of the cys and in some cases the I-helix oxygen binding thr probably means these enzymes do not use molecular oxygen, and may lack a heme prosthetic group [85].

Another pattern that is apparent from the tree (Fig. S10) is the concentration of ortholog pairs (marked by dots) in the CYP2 clan and the mito clan. *A. glabripennis* has 106 CYP genes and 19 predicted pseudogenes, while *Tribolium* has 137 genes and 10 pseudogenes. In the CYP2 clan all sequences on the tree are in seven ortholog pairs with *Tribolium* sequences except one *Tribolium* sequence (CYP304E1). Similarly, the mito clan has seven ortholog pairs, with the CYP12 family being the only exception. CYP12H1 in *Tribolium* pairs with four CYP12T sequences in *A. glabripennis*. This is evidence of divergence and expansion of the ancestral CYP12H-like gene in *A. glabripennis*. Only two ortholog pairs are seen in the CYP3 clan (CYP6BS and CYP9Y subfamilies) and only three are seen in the CYP4 clan (two CYP4G pairs and CYP352A). Other sequences are found in species limited blocks with as many as 21 sequences. The common ancestor of *Tribolium* and *A. glabripennis* had many fewer sequences in the CYP3 and CYP4 clans with independent expansion taking place after speciation. The ortholog pairs are presumably carrying out specific functions conserved over many millions of years of evolution. These genes include the CYP halloween genes involved in 20-hydroxyecdysone synthesis and metabolism [86]. The CYP4G genes are critical for making the impervious hydrocarbon coating on the exoskeleton that prevents dehydration, which is essential for colonization of land by the ancestors of insects [87]. The complete list of curated Cytochrome P450’s in the *A. glabripennis* genome is given in Additional file 2: Table S12; these are also alternatively presented and summarized in Additional file 2: Tables S13-15.

**Table S12. Curated Cytochrome P450s in the Aglab genome**

Due to its size, this table has been moved to the supplementary excel file entitled “AdditionalFile 2_04Aug2016”.

**Table S13. Insect P450’s in CYP name order.**

Due to its size, this table has been moved to the supplementary excel file entitled “AdditionalFile 2_04Aug2016”.

**Table S14. Insect P450’s sorted by clan.**

Due to its size, this table has been moved to the supplementary excel file entitled “AdditionalFile 2_04Aug2016”.

**Table S15. Insect P450’s by clan.**

Due to its size, this table has been moved to the supplementary excel file entitled “AdditionalFile 2_04Aug2016”.

**Figure S10. Phylogenetic relationships among the *A. glabripennis* and *T. castaneum* CYP450s.** 103 *A. glabripennis* and 128 *T. castaneum* CYP450 sequences were aligned with CLUSTAL Omega (http://www.ebi.ac.uk/Tools/msa/clustalo/) and the resulting tree was drawn in Figtree v1.3.1 and labeled in Adobe Illustrator CC. *T. castaneum* sequences are in red and *A. glabripennis* sequences are in blue. 20 Orthologous pairs are marked with black brackets. The four clans CYP2, CYP3, CYP4 and mito are shown as well as two outlier sequences that are unusual in that they do not have the conserved cys at the heme signature sequence. They are part of a small group of sequences including the *Locusta migratoria* CYP408 family that have lost the defining cysteine of the P450 family. Ortholog pairs are prominent in the CYP2 and mito clans (including the halloween genes), while blocks of red and blue signal gene expansions in the CYP3 and CYP4 clans. See Suppl. Materials: Tribolium.longhorned.beetle.P450s.FASTA.named.docx**.**


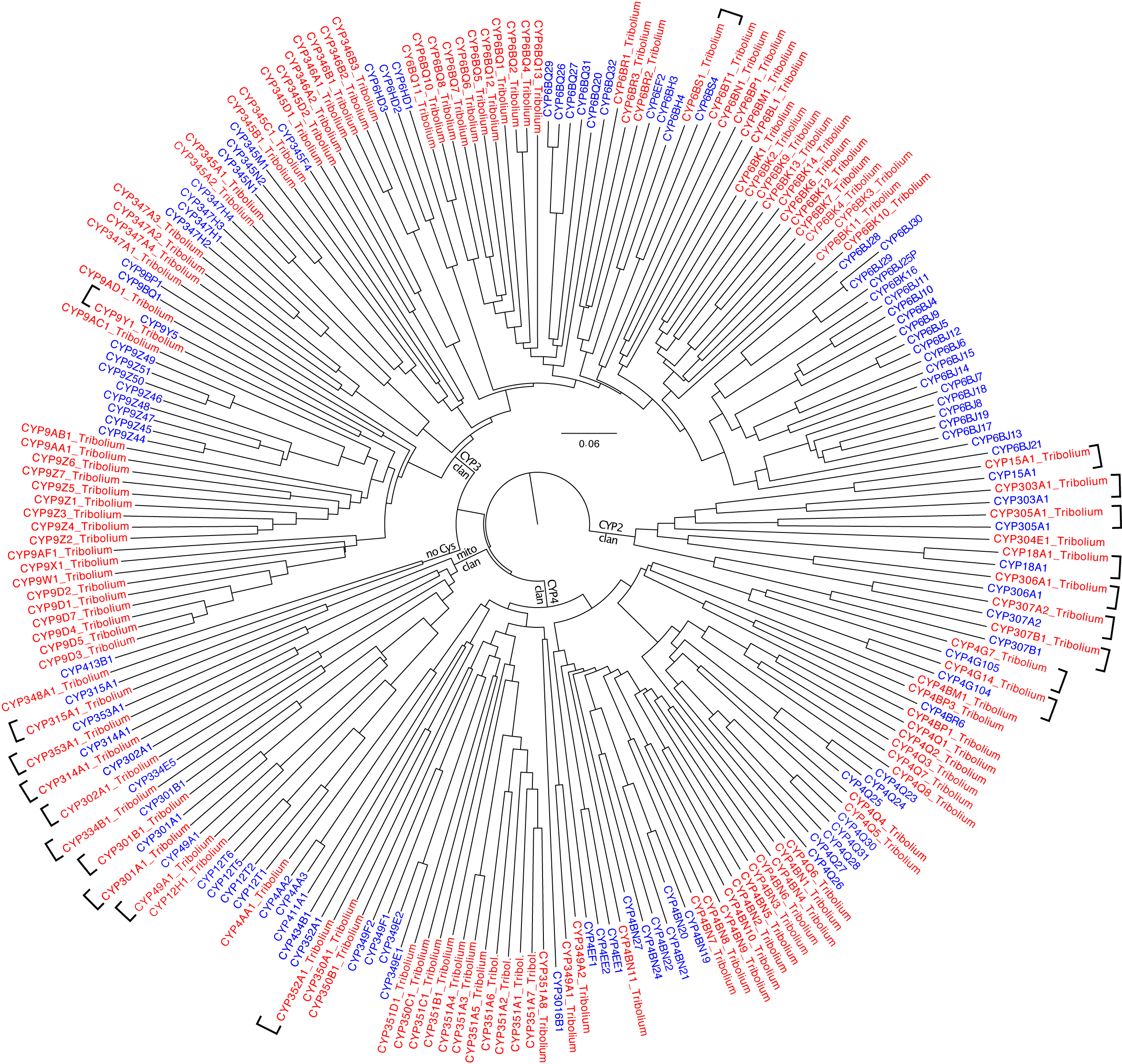


**III.2 UDP-glycosyltransferases**

**Contributor**

**Seung-Joon Ahn** (Max Planck Institute for Chemical Ecology, Department of Entomology)

**Summary**

UDP-glycosyltransferases (UGTs) catalyze the conjugation of a variety of small lipophilic molecules with uridine diphosphate (UDP) sugars, thereby increasing their solubility in water. Glycosylation by UGTs plays important roles in the detoxification and elimination of xenobiotics and in the regulation of endobiotics [88]. Insects have a large gene family encoding UGT [89], as it is common in viruses, bacteria, fungi, plants and animals [90], suggesting a vital role in living organisms. We identified 65 putative UGT genes including seven pseudogenes in the *Anoplophora glabripennis* genome by manual annotation (Additional file 2: Table S16). This number is the second largest among insects for which genomes have been sequenced. A gregarious locust, *Locusta migratoria*, contains the most (68 UGTs) according to a recent report [91]. Such a large number represents a substantial expansion of this gene repertoire in *A. glabripennis*. The deduced amino acid sequences showed similarity to insect UGTs and contained the UGT signature motif in the conserved C-terminal region supporting the idea that the genes are members of the UGT superfamily (Fig. S11).

**Discussion**

The maximum-likelihood tree constructed with the amino acid sequences revealed diversity and conservation of the *A. glabripennis* UGT gene family (Fig. S12B). The largest UGT family in *A. glabripennis*, UGT352, consisted of 21 UGTs and occupied a unique position in the tree, compared to other families which clustered together with *T. castaneum* UGTs. Members of the UGT352 family were located in three different genomic scaffolds, where most of them were arranged in a tandem manner (Fig. S12A), suggesting that several gene duplication events have occurred in these clusters. The *A. glabripennis*-specific UGT352 family may have obtained unique functions enabling *A. glabripennis* to adapt to a relatively wide range of host plants. Another lineage-specific gene expansion was found within UGT321 family, where seven *A. glabripennis* UGTs are clustered separately from four T. castaneum UGTs in the same family, thereby making its own subfamily (UGT321E). In contrast, a highly conserved member of UGT50 across diverse insects was also identified in *A. glabripennis* and placed in a separate position of the phylogenetic tree.

The gene structure supports the phylogenetic analysis of the *A. glabripennis* UGTs: each gene belonging to UGT352, UGT321, and UGT328 consists of 4 exons with a long first exon (ca. 810 aa) followed by three short exons, whereas each member of UGT323, UGT324, and UGT325 is composed of 4 exons with a short first exon (ca. 200 aa) and a long second exon (ca. 800 aa) followed by two short exons. UGT312 and UGT353 (AglaUGT_63 and _64) consistently contain genes with 5 exons.

The 65 *A. glabripennis* UGT genes (Additional file 2: Table S16) were mapped onto 16 different genomic scaffolds showing an uneven distribution of UGTs in the genome (Fig. S13). About 92% of UGTs are arranged in a tandem manner, with 50 UGTs concentrated in 7 different clusters containing more than 4 genes in each cluster. For example, the largest cluster is found on scaffold 72, where 14 genes of the UGT352 family are positioned all in the same orientation, suggesting that several gene duplication events have occurred in this cluster as shown together with the phylogenetic tree (Fig. S12). The second largest clusters are found on scaffold 44 and scaffold 133 with 8 UGTs in each. And the third largest clusters are placed in scaffold 76 and scaffold 772 with 6 UGTs in each. Such a clustered gene distribution indicates that most of the *A. glabripennis* UGTs have been abundant by tandem gene duplication, resulting in increased substrate range of host plant secondary metabolites probably by altering the N-terminal substrate binding domain of the enzyme.

**Table S16.** **List of UDP-glycosyltransferase (UGT) genes identified in the *Anoplophora glabripennis* genome.**

Due to its size, this table has been moved to the supplementary excel file entitled “AdditionalFile 2_04Aug2016”.

**Figure S11. The UGT signature motif of *Anoplophora glabripennis.*** Consensus sequences of the signature motifs were displayed by using WebLogo (<http://weblogo.berkeley.edu/>) **A.** Consensus insect sequence, **B.** *Anoplophora glabripennis* sequence.

**Figure S12 (Fig. 5, main paper).** **A maximum-likelihood phylogenetic tree of *Anoplophora glabripennis* UGTs.** **A.** indication of tandemly duplicated UGTs. **B.** Phylogenetic tree of UGTs: Deduced amino acid sequences of both *A. glabripennis* UGTs (color) and *T. castaneum* UGTs (black) were aligned using ClustalW and analyzed using the maximum-likelihood method based on the JTT matrix-based model with 1000 bootstrap replicates. Nodal support values lower than 70 are not shown.


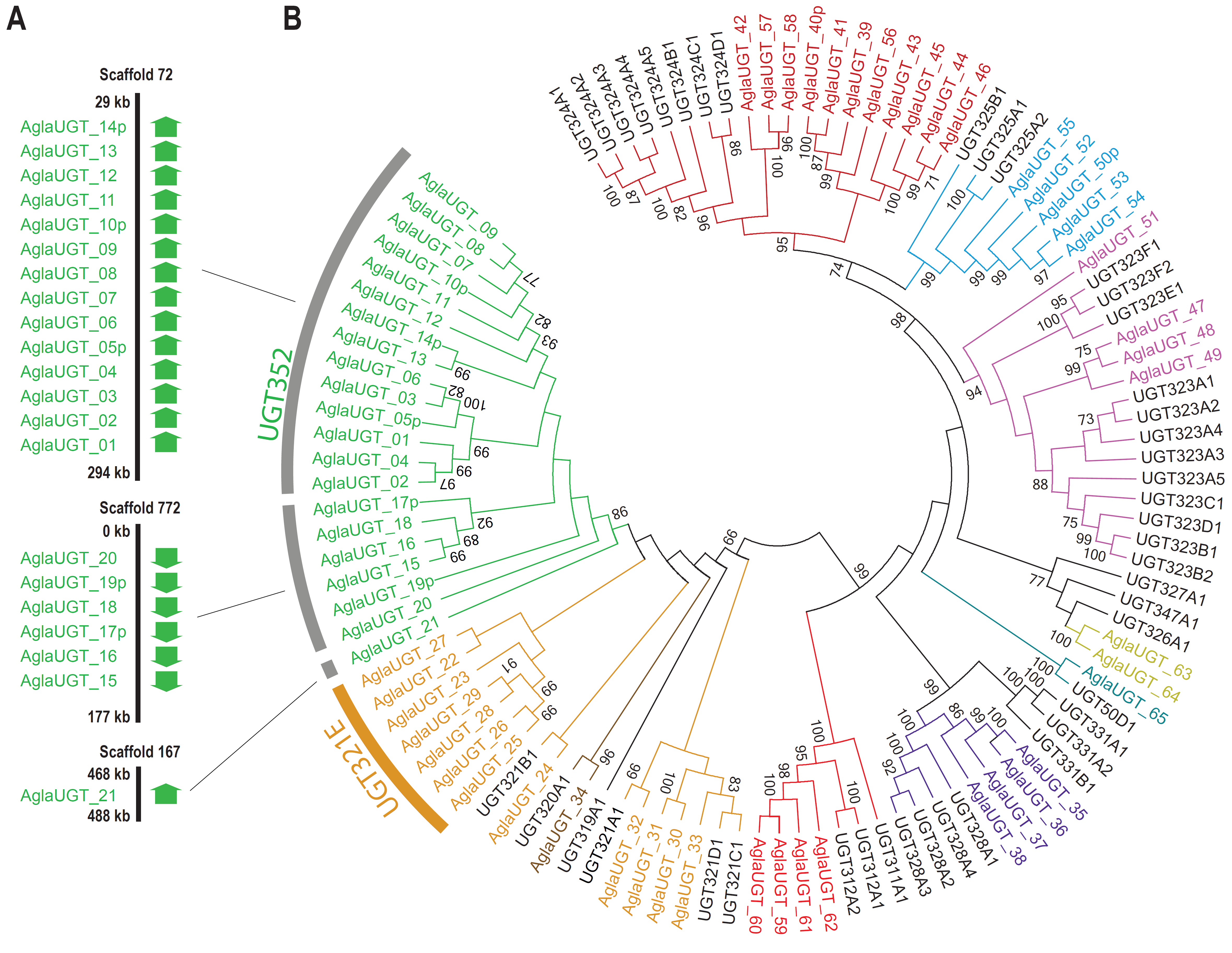


**Figure S13.** **Distribution of UGT genes on the *Anoplophora glabripennis* genomic scaffolds.** Only scaffolds harboring UGT genes are depicted, with UGT genes mapped at their relative physical location. Arrows indicate gene orientation.


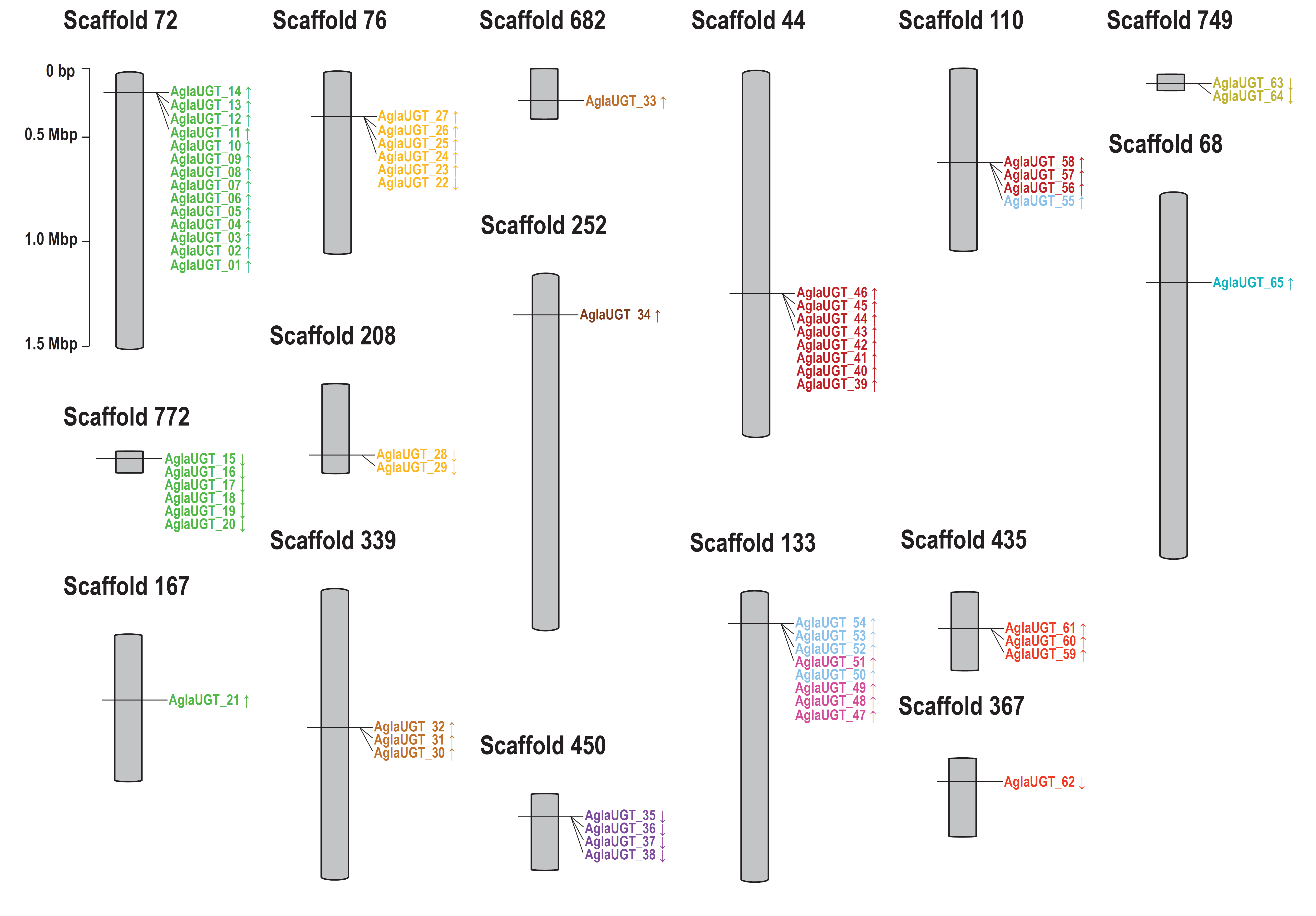


**III.3. Carboxylesterases**

**Contributor**

**Erin Scully** (USDA-ARS, Stored Product Insect and Engineering Research Unit, Manhattan, KS 66502)

**Summary**

Amino acid sequences from genes containing carboxylesterase (COesterase) PFAM domains in the *A. glabripennis* genome were aligned with genes annotated as COesterases in the mountain pine beetle and red flour beetle genomes in NCBI using muscle. OrthoMCL and BLASTP searches were also used to facilitate the identification of additional COesterases in the genomes of all three beetles. A maximum likelihood-based phylogenetic analysis was performed using Garli. 500 bootstrap pseudoreplicates were performed and the WAG + I + G substitution matrix was selected as the most optimal evolutionary model by ProtTest. Major functional clades of genes identified previously in [92] are colored. D: Integument Esterases; E: β- and pheromone esterases; F: Juvenile Hormone Esterases; H: Glutatactins; I: Function Unknown; K: Gliotacins; L: Neuroligins; M: Neurotactins (Fig. S14). The genes that are not color coded may be involved in metabolism of xenobiotics or may serve roles in digestion. Two large clades of COesterases present in the *A. glabripennis* genome are designated *A. glabripennis* Clade 1 and 2. Red dots indicate genes that were more highly expressed in the guts of *A. glabripennis* larvae reared on sugar maple compared to those feeding on artificial diet (see section III.1 Diet and digestive physiology). Black dots indicate genes that were more highly expressed in artificial diet fed larvae.

**Results and Discussion**

Carboxylesterases (COesterases) are members of large, divergent, and rapidly evolving multigene families that are often invovled phase I metabolism of xenbiotics via hydrolytic cleavage of carboxylic esters, the products of which are subsequently conjugated by other enzymes and ultimately excreted [93]. These genes are functionally diverse and can also play important roles in pheromone, plant volatile, cholesterol, and fatty acid metabolism [94, 95], digestion of ester linkages in plant cell walls [96], and processing neurotransmitters and other hormones [97]. In addition, several non-catalytic carboxylesterases were identified that can be involved in cell-cell interactions [98]. While their role in the detoxification of insectides has been extensively studied [99-101], the role of COesterases in the digestion of plant biomass [102] and the detoxification of plant defensive chemicals [24, 103] is only beginning to be understood. 107 gene models containing carboxylesterase domains were identified in the *A. glabripennis* genome, a number that is larger than the number of COesterases detected in the genome of any sequenced insect to date. Gene amplification followed by and diversificaton, subfunctionalization, and neofunctionalization is common in this gene family [104]. Supporting this theory, several of these genes occur in large clusters in various scaffolds throughout the genome, while others occur singly. For example, large clusters of carboxylesterases can be found on Scaffolds 315 (15 genes), 1 (8 genes), 39 (6 genes), and 146 (5 genes); however, 25% of the carboxylesterase genes occured as singletons (28 genes).

As described previously in *Leptinotarsa decemlineata*, large species specific clades were identified [92] in each of the three beetle genomes by ML analysis, which were hypothesized to metabolize xenobiotics. In the *A. glabripennis* genome, two large clades of COesterases specific to *A. glabripennis* were discovered. The first clade contained 17 COesterases, including all eight COesterases from scaffold 1 (AGLA000180 through AGLA000187), all six COesterases from scaffold 39 (AGLA000620 through AGLA000625), a cluster of three genes from scaffold 46 (AGLA001395, AGLA001397, and AGLA001398), and three singleton COesterases, one from Scaffold 229 (AGLA010530), a second from Scaffold1587 (AGLA019652), and a third from Scaffold 4721 (AGLA021994). While orthologous genes to AGLA00187 were detected in the genomes of both Tribolium (TCAS|XP_968291) and Dendroctonus (ENN80005), lineage specific expansions were also observed in these two beetle genomes, although not to the same extent as was observed in *A. glabripennis*. Specifically, the Dendroctonous genome harbored only a single paralog to ENN80005 while the Tribolium genome harbored three paralogs to XP_968291. A second clade contained 13 COesterases, including the majority of the COesterases from Scaffold 315 (AGLA013176, AGLA013178, AGLA013181, AGLA013183, AGLA013186, AGLA013187, AGLA013188, AGLA013189, and AGLA013190) and a cluster of five COesterases from Scaffold 146 (AGLA010575 through AGLA010579). These genes lacked orthologs in *Tribolium* and *Denodroctonus*. Other carboxylesterases could be readily assigned to orthologous groups and likely serve functions as juvenile hormone esterases, integument esterases, acetylcholinesterases, neurolignins, gliotacins, glutatacins, or neurotacins.

Carboxylesterase genes were among the most highly induced genes in larvae feeding in wood with 24 genes being more highly induced in these larvae. The most highly upregulated carboxylesterases were those found as tandem repeats, including five of the eight genes localized to Scaffold 1 (AGLA000181, AGLA000182, AGLA000184, AGLA000186, and AGLA000187), all five gene models located on scaffold 39 (AGLA000621-AGLA000625), four of the 15 genes located on scaffold 315 (AGLA013176, AGLA013184, AGLA013186, AGLA013190), and two of the five COesterases from Scaffold 146 (AGLA010575 and AGLA010579). In fact, AGLA000623 was one of the most highly upregulated genes in insects feeding in sugar maple with over an 8-logfold induction compared to wild type. An overwhelming majority of the COesterases that were induced when feeding in wood were the COesterases assigned to *A. glabripennis*-specific clades (including 14 of the 18 genes assigned to *A. glabripennis* clade 1). Although insect carboxylesterases can also have roles in pheromone metabolism, developmental regulation, and pesticide resistance, the induction of these carboxylesterases in insects feeding in living trees suggest these genes may be host plant inducible and serve detoxification purposes. Alternateively, hemicellulose polysaccharides are directly esterified to the lignin polymer. Subsets of these carboxylesterases could disrupt ester linkages, exposing reducing and non-reducing sugars to hydrolytic enzymes and directly enhancing the digestibility of cell wall carbohydrates as has been proposed in termites [96]. In contrast, only five singleton COesterase genes were more highly expressed in sugar maple fed larvae (AGLA000658, AGLA001496, AGLA002953, AGLA004258, and AGLA021994). Furthermore, many of the singleton carboxylesterases were expressed at similar levels in both the diet- and sugar maple reared insects. These genes often had detectable homologs in *Tribolium* and *Dendroctonus*, potentially signifying conserved roles in development, fatty acid metabolism, or other cellular and metabolic processes.

**Figure S14. Phylogenetic analysis of COesterases detected in the *Anoplophora glabripennis*, *Dendroctonus ponderosae*, and *Tribolium castaneum* genomes.** A maximum likelihood-based phylogenetic analysis was performed using Garli. 500 bootstrap pseudoreplicates were performed and the WAG + I + G substitution matrix was selected as the most optimal evolutionary model by ProtTest. Major functional clades of genes identified in [92] are colored. D: Integument Esterases; E: β- and pheromone esterases; F: Juvenile Hormone Esterases; H: Glutatactins; I: Function Unknown; K: Gliotacins; L: Neuroligins; M: Neurotactins. The genes that are not color coded may be involved in metabolism of xenobiotics or may serve roles in digestion. Two large clades of COesterases present in the *A. glabripennis* genome are designated *A. glabripennis* Clade 1 and 2. Red dots indicate genes that were more highly expressed in the guts of *A. glabripennis* larvae reared on sugar maple compared to those feeding on artificial diet (see section III.1 Diet and digestive physiology). Black dots indicate genes that were more highly expressed in artificial diet fed larvae.

**III.4 Myrosinase and cyanogenic β-glycosidase-like sequences**

**Contributors**

**Leslie A. Kuhn** (Michigan State University, Departments of Biochemistry and Molecular Biology, Computer Science and Engineering, and Fisheries and Wildlife)

**Ann M. Ray** (Xavier University, Department of Biology)

**Erin Scully** (USDA-ARS, Stored Product Insect and Engineering Research Unit, Manhattan, KS 66502)

**Introduction**

Here we analyze whether sequences putatively identified as members of the beta-glycosyl hydrolase GH1 family (referred to as beta-glycosidases for brevity) are likely to encode for myrosinase (thioglucoside hydrolase) or cyanogenic beta-glycosidase (CBG) enzymes, two subfamilies of GH1. These enzymes are involved in detoxification of host compounds and chemical communication in plants and insects. We became interested in insect myrosinases, their roles in chemical signaling, and the possibility of controlling their activity with inhibitors when we discovered a UniProt *Anoplophora glabripennis* sequence annotated by sequence homology as myrosinase-1 according to [58]. A myrosinase system very similar to that in plants is known to exist in insects such as cabbage aphids (*Brevicoryne brassicae* (L.)) and turnip aphids (*Lipaphis (Hyadaphis) erysimi* (Kaltenbach)*,* Hemiptera: Aphididae), which use the isothiocyanate products of myrosinase (ITCs) as components of an alarm pheromone in combination with *E*-β-farnesene [105, 106]. Furthermore, in striped flea beetles, (*Phyllotreta striolata* (F.), Coleoptera: Chyromelidae) the ITC products of the beetle-expressed myrosinase enhance the response of beetles to male-produced aggregation pheromones [50, 107-109]. Experiments are in progress to test the hypothesis that *A. glabripennis* adults and larvae express myrosinase, when fed on native food sources that require detoxification by myrosinase, and that field and laboratory-reared beetles differ in their expression of myrosinase. In addition to Brassicaceae, glucosinolates are produced in 15 other plant families, including Moringaceae and Pittosporaceae (moringa trees and pittosporum trees, shrubs, and lianas) which are widespread in Asia and also grown in the U.S. [252]. Additional studies are in progress to address the interesting question of whether myrosinase products act as chemical attractants for adults of *A. glabripennis*, possibly in combination with known male-produced compounds [110, 111].

Cyanogenic beta-glycosidases (CBGs) are another GH1 subfamily involved in food detoxification by insects. CBGs are known to detoxify the “cyanide bomb” that plants use to deter herbivory, with cyanide-containing compounds such as amygdalin (also known as laetrile) being synthesized in trees of the genus *Prunus* [112]. Insect herbivores may metabolize cyanogenic glucosides or sequester them, also for use in defense against predators [113]. A few species of Arthropoda (within the classes Diplopoda, Chilopoda, and Insecta) synthesize cyanogenic glucosides *de novo*, and some of these same species are able to sequester cyanogenic glucosides from their host plants, e.g., Insecta: Lepidoptera: Zygaenidae (forester and burnet moths) [113]. Given that *Prunus* is one of the host genera for *A. glabripennis* [114], sequences resembling myrosinase may actually function as CBGs in *A. glabripennis*, since the two enzymes are closely related [106]. In fact, some CBG sequences are more distantly related to each other than they are to myrosinase sequences or other GH1s [115]. Yet another member of the GH1 family, lactase-phlorizin hydrolase (also known as lactase or LPH; see http://www.cazy.org/GH1.html), is found in the midguts of Lepidoptera such as the fall armyworm (*Spodoptera frugiperda* (Smith), Noctuidae) and hydrolyzes galactolipids and hemicelluloses by cleaving them at two different active sites [116].

GH1s have a classical (α/β)_8_ TIM barrel fold in which the two key active site glutamic acid residues are ~200 residues apart, located at the C-termini of β-strands 4 (acid/base) and 7 (nucleophile) [117]. The *Sinapis alba* myrosinase, however, contains a glutamine residue in place of the classic glutamate at the end of strand 4, because instead it uses an ascorbate cofactor as the catalytic base [118]. Burmeister et al. [119] suggested that myrosinase and cyanogenic beta-glucosidase act identically on substrates.

**Methods and Results**

Twenty-three potential *A. glabripennis* myrosinase amino acid sequences were identified by BLAST search [37] for the highest-scoring alignments (typical identity of ~44%) between *A. glabripennis* GH1s and *Tribolium castaneum* sequences annotated as myrosinases in the SwissProt/UniProt database [120]. These sequences are labelled as follows in the *A. glabripennis* genome: *A. glabripennis* genomic sequences analyzed: AGLA004459-PA, AGLA004460-PA, AGLA004461-PA, AGLA009626-PA, AGLA009627-PA, AGLA009631-PA, AGLA009633-PA, AGLA009636-PA, AGLA009638-PA, AGLA009642-PA, AGLA009643-PA, AGLA009644-PA, AGLA014364-PA, AGLA014878-PA, AGLA016146-PA, AGLA016151-PA, AGLA016153-PA, AGLA016544-PA, AGLA016545-PA, AGLA017752-PA, AGLA018044-PA, AGLA018048-PA, and AGLA018242-PA. The *A. glabripennis* sequence lengths vary from 48 residues (likely a protein sequence fragment) to 1391 residues (probably forming a multi-domain protein). Amino acid sequence motifs have been defined for beta-glycosyl hydrolases by Henrissat and colleagues [121-125], including a set of five aligned sequence blocks available in the PRINTS database (http://www.bioinf.man.ac.uk/cgi-bin/dbbrowser/sprint/searchprintss.cgi?prints_accn=GLHYDRLASE1). The ProSite database contains a simpler set of two sequence motifs, the first also defined by Henrissat and colleagues (http://prosite.expasy.org/cgi-bin/prosite/nicedoc.pl?PS00653) as the consensus pattern:

**F-x-[FYWM]-[GSTA]-x-[GSTA]-x-[GSTA](2)-[FYNH]-[NQ]-x-E-x- [GSTA]**

All known GH1 sequences in SwissProt at the time the ProSite motif was defined in 1995 satisfied the above pattern, and there were no false positives. The second ProSite motif for GH1s, labeled PS00572, is:

**[LIVMFSTC]-[LIVFYS]-[LIV]-[LIVMST]-E-N-G-[LIVMFAR]-[CSAGN]**

According to ProSite, SwissProt sequence matches to this motif included 105 true positives, 95 false negatives, and 59 false positives. This motif is thus less discriminatory for GH1s. A third, nonredundant GH1 motif has been defined (Fig. 3 in [126]), corresponding to the **VKYWLTINQLYSVPTR** region in *S. alba* myrosinase in which the central glutamine (Q) residue corresponds to the conserved glutamate acid/base residue found in other GH1s.

To analyze relationships between the 23 *A. glabripennis* myrosinase-like GH1 sequences and known myrosinases and CBGs, multiple sequence analysis was performed with MUSCLE [31], as implemented in the software suite Mega6 (Molecular Evolutionary Genetics Analysis; http://www.megasoftware.net). The set of 23 myrosinase-like GH1 sequences was seeded with an additional set of seven GH1 sequences of known function (Fig. S15): *B. brassicae* myrosinase 1 (sp|Q95X01), *P. striolata* myrosinase (tr|A0A059UAD7), *S. alba* myrosinase (Protein Data Bank entry 2wxd, chain M), *P. striolata* GH1 (gi|634006832), *Lepisosteus oculatus* (Winchell) lactase-like protein (lactase-phlorizin hydrolase, a CBG homolog; gi|573881201), *Trifolium repens* CBG (Protein Data Bank entry 1cbg, chain A), and a beta-glycosidase from *S. sulfataricus* (Protein Data Bank entry 1uwt, chain A). From this multiple sequence alignment, which correctly aligned the conserved GH1 motifs for the subset of 17 *A. glabripennis* sequences with complete coverage of the catalytic domain (AGLA004459, 4460, 4461, 9627, 9636, 9638, 9643, 9644, 14364, 14878, 16146, 16153, 16544, 16545, 17752, 18044, and 18242) and the 7 additional previously characterized GH1 sequences, a pairwise distance matrix between sequences was calculated in Mega6 by using the [127] model of amino acid substitution likelihood, with uniform rates across sites and pairwise deletion for handling any sequence gaps. Based on the pairwise distance matrix, agglomerative cluster analysis of these 24 sequences was performed in SciPy [128] by using the complete linkage clustering function (scipy.cluster.hierarchy.complete). Complete linkage clustering has several practical and intuitive advantages. At any given sequence similarity cutoff (using the similarity values defined in the pairwise distance matrix described above), all sequences grouped in a cluster are guaranteed to have at least that pairwise degree of similarity. Furthermore, the defined clusters are guaranteed to be the densest such grouping of sequences for a given degree of similarity. The results are deterministic and do not depend on the order in which sequences are input. Upon clustering of the 17 complete *A. glabripennis* GH1-like sequences and seven GH1 sequences of known function (results not shown), the known myrosinases split across different sequence clusters; in particular, the *S. alba* myrosinase that uses an ascorbate cofactor appears in a separate cluster from the others (as was also found by the [115] analysis of complete myrosinase sequences).

Because analyses like the above treat all parts of the amino acid sequence as equally important, clustering aimed at functional annotation may be led astray by the low degree of conservation of solvent-exposed surface residues outside the active site, due to few steric, folding, or functional constraints on their evolution. Instead, focusing on active site and functional motif sequence conservation within the context of overall protein fold conservation is a better indicator of the conservation of substrate binding and reaction mechanism. Thus, to analyze the likelihood of myrosinase function in one or more of the *A. glabripennis* GH1-like sequences, we developed a sequence fingerprint consisting of only the residues contributing to the characteristic GH1 and myrosinase motifs described above, plus additional residues that contribute to the active site based on the available *S. alba* myrosinase crystal structure (Protein Data Bank entry 2wxd). These residues are not necessarily contiguous in sequence, since active sites typically involve discontiguous residues forming a substrate interaction surface. The motifs and active site residues only are presented in N-terminal to C-terminal order in the fingerprint (Fig. S15), as extracted from the MUSCLE alignment of the complete protein sequences. This fingerprint shows several interesting features:

- AGLA009631, 9633, and 9636 have an unusual residue (Asp) in place of the highly conserved Glu at the acid/base catalyst position in the [126] motif centered on the catalytic acid/base. Unconserved but chemically similar residues are known to occur in this position in some active GH1s: Gln in the *S. alba* myrosinase that uses ascorbate as a cofactor and Asn in the *L. oculatus* CBG-like lactase. Furthermore, Asp may provide a catalytic acid/base appropriate for interacting with a larger substrate than is found in active sites with Glu, as these side chains differ by just one methylene group in length.
- AGLA009636 and 9643 are unlikely to be active GH1s based on having an Ala or Gln substitution, respectively, for the conserved Glu in ProSite motif 2 (catalytic nucleophile position).

The pairwise distance matrix for the above aligned active site sequence motifs was calculated in Mega6 (using parameters described above), followed by complete linkage cluster analysis in order to group subfamilies of these sequences according to active site and GH1 motif similarity (Fig. S16). This analysis of motifs, unlike the full sequence analysis, groups the known myrosinases (lower blue lines in Fig. S16) and suggests that one of the *A. glabripennis* sequences in particular, AGLA018242-PA, is closely similar to a known insect myrosinase (UniProt Q95X01 from *B. brassicae*).

A known CBG and CBG-related GH1, lactase-phlorizin hydrolase, also fall in a single cluster when GH1 motifs and active site residues are used to assess similarity (green lines in Fig. S16). Further analysis is needed to evaluate the red cluster of *A. glabripennis* sequences, because they are intriguingly intermediate in similarity to known myrosinases and the known cyanogenic beta-glycosidase. Husebye et al., [106] noted that *B. brassicae* myrosinase shows close sequence and structural similarity to both *T. repens* CBG (41% sequence identity) and *S. alba* myrosinase (34% identity).

The 3-dimensional compatibility of *A. glabripennis* sequences to the myrosinase atomic structure can be assessed by homology modeling. For instance a 3-dimensional structural model (Fig. S17) was built for one of the GH1 myrosinase candidates (AGLA009643-PA, annotated by [58] as V5GKP2 in the SwissProt/UniProt database) based on its alignment with *B. brassicae* myrosinase, by using SwissModel homology modeling software [129] (http://swissmodel.expasy.org). The resulting model was judged to be of reasonable accuracy, based on the SwissModel structural evaluation of bond stereochemistry and intramolecular contacts indicating similar quality to well-resolved crystal structures. This model for the *A. glabripennis* protein structure (Fig. S17) also provides an unambiguous definition of the active site residues and their relative orientation, which is valuable for assessing the positioning of catalytic groups and the likelihood of interacting with glycosidic versus other substrates. An alternative model for this V5GKP2 sequence based on using the *T. repens* CBG structure (Protein Data Bank entry 1CBG) was of much lower stereochemical quality, consistent with the active-site and motif cluster analysis (above) indicating that the AGLA009643 GH1 motifs and active site are more closely related to myrosinases.

**Discussion**

Given that *A. glabripennis* is highly polyphagous (not limited to one food source) and not known to feed on glucosinolate-myrosinase system containing plants, it is unclear why the beetles would express one or more myrosinase-like enzymes. Myrosinase is necessary to defuse the so-called defensive “mustard oil bomb” of glucosinolates and their products (isothiocyanates) produced by plants in the order Brassicales in order to deter herbivory [130]. Fifteen non-Brassicaceous plant families are also known to produce glucosinolates, and at least two are woody plants native to the *A. glabripennis* range: Moringaceae (trees) and Pittosporaceae (trees, shrubs, and lianas). [252-256]. Oligophagous or monophagous insects that feed on these plants often employ a system for detoxification by sequestration of glucosinolates, which are then broken down and released by an insect-expressed myrosinase system such as those in flea beetles and cabbage aphids [50, 106]. In their native range, the larval host plants of *A. glabripennis* are trees of the genus *Acer, Populus, Salix* and *Ulmus*, whereas in North America *A. glabripennis* larvae can develop in hosts in the native tree genera and also in *Aesculus, Albizia, Betula, Cercidiphyllum, Fraxinus, Platanus, Prunus*, and *Sorbus* [114]. These genera are not known to contain the myrosinase system. However, glucosinolates could be taken up from another glucosinolate containing food source.

Myrosinase could also play a role in chemical signaling in *A. glabripennis* adults, as is the case for *P. striolata*. *A. glabripennis* may sequester glucosinolates or respond to the products of myrosinase hydrolysis (ITCs), and these compounds may synergize the attraction of adult beetles to the male-produced compounds, 4-(*n*-heptyloxy)butan-1-ol, 4-(*n*-heptyloxy)butanal, and (3*E*,6*E*)-α-farnesene [110, 111]. This finding would be analogous to a system already known to exist in cabbage and turnip aphids, which combine ITCs with (*E*)-β-

farnesene as an alarm pheromone [105, 106]. Experiments are in progress to evaluate the response of *A. glabripennis* adults to ITCs alone, and in combination with male-produced attractants (A.M.R., unpub. data).

Another intriguing possibility is that one or more *A. glabripennis* sequences is active as a CBG, as suggested by the presence of a cluster of *A. glabripennis* sequences intermediate between known CBGs and myrosinases. Toxic cyanogenic glycosides are used by plants as a defense system analogous to the myrosinase system. Cyanogenic glycoside substrates are packaged in an adjacent compartment to CBGs in plants. Herbivory breaches the compartments, initiating the breakdown of cyanogenic glycosides and release of hydrogen cyanide gas [113]. This system is present in a number of plants in the Rosaceae, including trees of the genus *Prunus*, a known host of *A. glabripennis* [114]. The cyanogenic glycosides prunasin and amygalin are found in tissues of *Prunus* [131], often in levels that are toxic to herbivores, including humans and domestic animals [132]. Aside from amygdalin and prunasin, linamarin and lotaustralin are cyanogenic glycosides that are widespread in plants and often occur together, particularly in Fabaceae [133]. Both cyanogenic glycosides and their release of hydrogen cyanide gas can affect the behavior of herbivorous insects and other animals. Specialist insect herbivores have developed CBG systems mirroring those in plants to metabolize cyanogenic glucosides or sequester them for use in predator defense, just as insects developed myrosinase systems to combat predation and aid in signaling to conspecifics [113].

**Acknowledgments**

We sincerely thank Sebastian Raschka (Michigan State University) for his contributions to the Mega6 and SciPy cluster analysis of sequences, and also Kathryn Morris, Vanessa Lopez, Scott Gula, and Brenna Walters (Xavier University) for their help with the preliminary laboratory bioassays.

**Author contributions**

L.A.K. developed the chemical signaling hypothesis for myrosinases and cyanogenic glycosidases in *A. glabripennis*, performed the sequence and structural bioinformatics analysis, and drafted this section on myrosinase and cyanogenic β-glycosidase-like sequences; A.M.R. designed and performed experiments and contributed to writing and editing the manuscript; E.S. provided myrosinase-like genomic sequence data for analysis, suggested roles for these enzymes in digestive physiology, and edited the manuscript.

**Fig. S15. GH1 and myrosinase motifs for 23 *A. glabripennis* proteins and seven known GH1s from other organisms**, as described in the Methods, colored by Mega6 according to amino acid chemistry: yellow, hydrophobic; light green, Tyr; dark green, polar; blue-green, His; pink, Gly; brown, Cys; red, Asp or Glu; dark blue, Lys or Arg. *A. glabripennis* sequences with substantial gaps (indicated by dashes on white background in the fingerprint) were omitted from the subsequent distance matrix calculation and clustering due to being incomplete sequences (unable to form the GH1 fold), though the corresponding full-length sequence, if expressed, could be a GH1. In the annotations beneath the fingerprint, “AS” indicates active site glucose or aglycone binding residues in myrosinase or CBG, as defined in Fig. 3 of [106]. Additional active-site residues were identified by using PyMOL software (v. 1.5.0.5, Schrödinger, LLC, NY, NY) based on occurring within contact distance (5 Ångstroms) of the E18 inhibitor in Protein Data Bank crystal structure 2wxd (*S. alba* myrosinase).


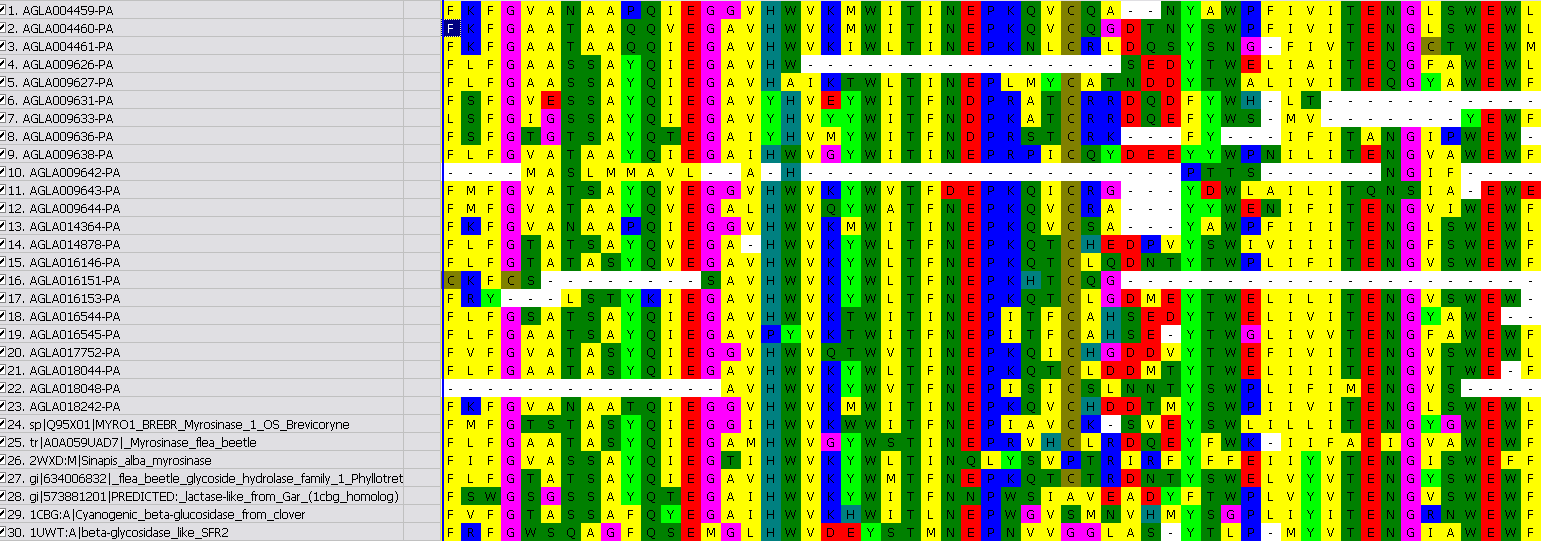


ProSite GH1 motif 1 region AS Cicek motif for GH1 including AS (discontig- ProSite GH1 motif 2; AS

conserved E, the acid/base uous residues) E is a catalytic

catalyst (Wang, 1990); preceding nucleophile

Asn H-bonds to 2-OH in GH1s ­­(Withers,1990)

**Fig. S16*.* Complete linkage clustering showing similarity in active site motif regions in *A. glabripennis* (AGLAB) myrosinase- and cyanogenic β-glycosidase-like sequences and characterized GH1s from other organisms**, based on pairwise Jones-Taylor-Thornton amino acid substitution distance followed by complete linkage clustering. Known myrosinases (MYR) appear in a cluster with candidate MYRs from *A. glabripennis* (blue) and known cyanogenic β-glycosidases (CBG) and close relatives also form a cluster (green).


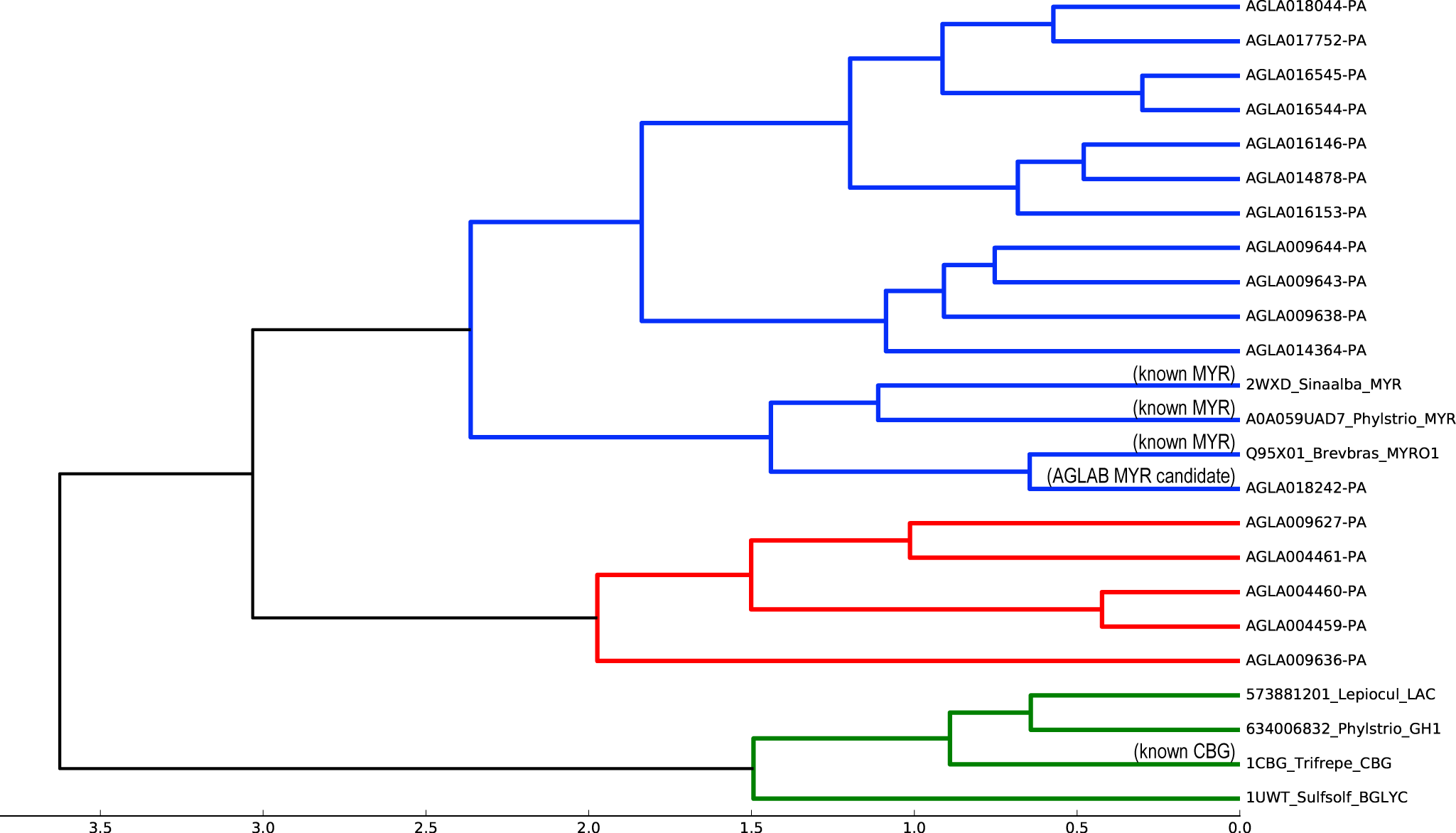


Jones-Taylor-Thornton Amino Acid Substitution Distance

**Fig. S17*.* Evaluation of a potential *A. glabripennis* myrosinase by 3-dimensional modeling** (carbon atoms and main-chain ribbons shown in yellow), using the X-ray structure of *B. brassicae* myrosinase (Protein Data Bank entry 1WCG; carbon atoms and main-chain ribbons in green) as a template for the *A. glabripennis* sequence AGLA009643-PA. The glucosinolate substrate-like E18 inhibitor from *S. alba* myrosinase is shown with carbon atoms in pink, for reference. Immediately beneath the alpha carbon of the phenyl group in E18, the GH1 conserved catalytic Glu acid/base appears in overlapping positions in the B. brassicae myrosinase and the *A. glabripennis* myrosinase-like protein. However, the Glu catalytic nucleophile conserved in GH1s including myrosinase is not conserved in this *A. glabripennis* sequence, suggesting it is not active as a myrosinase. The figure was rendered in PyMOL v. 1.5.0.5.


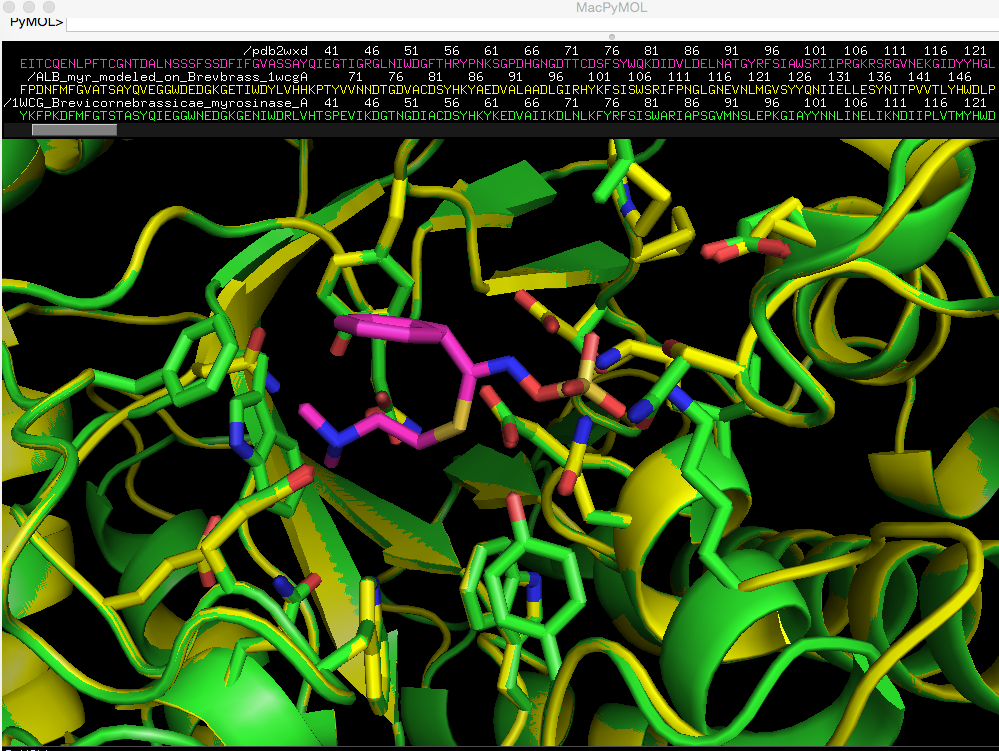


**III.5 Digestive Proteinases**

**Contributors**

**Erin Scully** (USDA-ARS, Stored Product Insect and Engineering Research Unit, Manhattan, KS)

**Summary:**

Digestive and metabolic plasticity is key to being able to survive in a broad range of healthy host plants with different nutritional compositions and varying repertoires of defensive compounds [134, 135]. Further, digestive enzyme inhibitors (e.g., proteinase inhibitors) are common compounds produced by plants as a primary defense against herbivores [136]. Expansions of families of digestive enzymes may allow *A. glabripennis* and other polyphagous insects to cope with these challenges [137], permitting them to successfully colonize and complete development in a broad range of host trees. Previously, proteinase activity levels were shown to fluctuate in the polyphagous cotton bollworm (*Helicoverpa armigera*) as it fed on different host plants with varying nutritional qualities and protein content [134] and mRNA levels of various proteinase genes were altered when it was exposed to various digestive proteinase inhibitors, such as the Kunitz type proteinase inhibtor produced by soybeans [138].

While several studies have shown that the ability to modulate proteinase gene expression is paramount to overcoming host plant defenses [139], the genomic repertoire of proteinases encoded by polyphagous insects have not been reportered previously. Gene models for over 280 genes predicted to encode various classes of proteinases were detected in the *A. glabripennis* genome, including 53 aspartyl proteases, 49 cysteine proteinases, and 186 trypsin proteinases. Approximately 100 of these proteinases possessed signal peptides (82 trypsin proteinases, 18 cysteine proteinases, and one aspartyl proteinase), signalling secretion and a possible role in digestion while 24 of the trypsin proteinases contained CLIP domains characteristic of proteinases with roles in innate immunity. In contrast, 26 aspartyl proteinases, 32 cysteine proteinases, 153 trypsin proteinases (14 with CLIP domains), were identified in the *T. castaneum* genome while 11 aspartyl peptidases, 35 cysteine proteinases, and 86 trypsin proteinases (4 with CLIP domains), were identified in the genome of the *D. ponderosae*.

Retreival of orthologous groups from orthoDB indicated lineage-specific expansions of several orthologous groups in *A. glabripennis* compared to the other two beetle genomes. The largest expansions were observed in orthogroups EOG8V724X and EOG8V19NQ, which both contained genes predicted to encode tyrpsin proteinases. The majority of these genes possessed signal peptides and all of the genes in these ortholog group lacked CLIP domains, suggesting that these genes may serve roles in digestion. In *A. glabripennis*, the occurrence of a tandem array of eight trypsin genes on scaffold Scaffold488 (orthogroup EOG8V724X) and seven trypsin genes on Scaffold 87 (orthogroup EOG8V19NQ) were associated with the expansions in these two orthogroups. In contrast, only two *D. ponderosae* gene models and no *T. castaneum* gene models were assigned to orthogroup EOG8V724X while four *T. castaneum* gene models and three *D. ponderosae* gene models were assigned to orthogroup EOG8V19NQ. In addition, only 36 of the 186 trypsin genes could be found as 1:1:1 orthologs in the genomes of these three beetles while 79 were paralogous copies scattered throughout the *A. glabripennis* genome. The remaining trypsin proteinases were unique to *A. glabripennis* and orthologous copies were not found in the other two beetle genomes. Additionally, of the the 24 CLIP domain serine proteinases harbored in the *A. glabripennis* genome, six could be found in 1:1:1 orthologs in the genomes of the three beetles, one was found as a 1:1 ortholog pair with *T. castaneum* and the rest were present as *A. glabripennis* specific paralogs. The same trends were noted for cysteine and apsartyl peptidases. Of the 49 cysteine proteinase genes detected in the *A. glabripennis* genome, 16 occurred as 1:1:1 orthologs in the three beetle genomes, 6 occurred as 1:1 orthologs in either *A. glabripennis* and *D. ponderosae* or *A. glabripennis* and *T. castaneum*, and the remainder were paralogous copies. Likewise, only four of the 53 aspartyl peptidase occurred as 1:1:1 orthologs while two were present as 1:1 orthologs in either *A. glabripennis* and *D. ponderosae* or *A. glabripennis* and *T. castaneum*, and the remainder were paralogous copies in the *A. glabripennis* genome. Similar findings were observed in the genomes of the other two beetles with the majority of the proteinase genes being unique to each beetle species, suggesting that the evolution of these proteinases in the three beetle genomes is largely independent and may be correlated with exposure to different digestive enzyme inhibitors and also with feeding on different diets. These genes families may be dynamic and may largely shape the digestive physiology of beetles and other insects.

Nitrogen, free amino acids, and protein are limited in woody tissue; however, several proteinases were induced in wood-feeding larvae and may play key roles in scavenging nitrogen from this environment either directly from plant cell wall proteins crosslinked in the lignocellulose matrix or from its midgut endosymbionts. 71 different proteinases were upregulated in insects feeding in wood, many of which were larval-specific and were not expressed in adults of *A. glabripennis*. These included 50 trypsin proteinases (only two of which possed CLIP domains associated with innate immunity in insects), 20 cysteine proteinases, and one aspartyl proteinase; 22 of the trypsin genes and 11 of the cysteine proteinase genes that were induced possesed signal peptides and could be involved in digestion. Further, clusters of trypsin proteinases on scaffolds 46 and 49, a series of cysteine proteinases on scaffolds 76, 224, and 1540, and a pair of aminopeptidases on scaffold 260 were induced in insects feeding in sugar maple. The majority of the proteinases induced as these larvae fed on a preferred host were those that occurred as paralogs or those that lacked orthologs in the the *T. castaneum* or *D. ponderosae* genomes. For example, only 7 of the trypsin genes that were induced as *A. glabripennis* fed in sugar maple occurred as 1:1:1 orthologs in the *T. castaneum* and *D. ponderosae* genomes while only 10 could be found as 1:1 orthologs between either *A. glabripennis* and *T. castaneum* or *A. glabripennis* and *D. ponderosae*. The remainder were present as *A. glabripennis*-specific paralogs. Likewise, a single cysteine proteinase that was upreguted as *A. glabripennis* larvae fed in sugar maple was found in a 1:1:1 orthologous relationship with *T. castaneum* and *D. ponderosae* genes. Three were found as 1:1 orthologs between either *T. castaneum* and *A. glabripennis* or *A. glabripennis* and *D. ponderosae* and 16 occurred as paralogous copies in the *A. glabripennis* gneome. Interestingly, very few reads mapped to the aspartic proteinases in insects feeding on either diet, suggesting that they were not expressed under our experimental conditions. Why such a diverse profile of digestive proteinases is so highly induced in insects feeing in sugar maple is unknown; however, phytophagous insects typically produce a diversity of proteinases, possibly as a mechanism to counteract the production of defensive proteinase inhibitors commonly produced by plants in response to herbivory [137, 140]. Although the expansion of digestive proteinases in *A. glabripennis* may contribute to the polyphagous lifestyle of this insect and the induction of these genes in wood-feeding larvae signify that they could be important for protein acquisition under nutrient limiting conditions, comparisons of the genomic repertoires of proteinases and proteinase inhibitors from the genomes of other polyphagous beetles as well as expresion profiles of these genes from *A. glabripennis* feeding in different host tree species will be informative for ascertaining what role these genes play in the determination of host range.

**IV. Comparative Genomics of Phytophagy and Detoxification**

**Contributors**

**Robert M. Waterhouse** (Department of Genetic Medicine and Development, University of Geneva Medical School, Geneva, Switzerland; Swiss Institute of Bioinformatics, Geneva, Switzerland; Computer Science and Artificial Intelligence Laboratory, Massachusetts Institute of Technology, Cambridge, MA, USA; The Broad Institute of MIT and Harvard, Cambridge, MA, USA)

**Jian Duan** (USDA-ARS Beneficial Insects Introduction Research, Newark, DE, USA)

**Scott Geib** (USDA-ARS, Pacific Basin Agricultural Research Center)

**Duane D. McKenna** (University of Memphis, Department of Biological Sciences)

**Armin Moczek** (Indiana University, Blomington, IN, USA)

**Erin Scully** (USDA-ARS, Stored Product Insect and Engineering Research Unit, Manhattan, KS)

**Methods**

Gene families and sub-families of interest associated with xylophagy and polyphagy or detoxification were identified by searching for matches to relevant InterPro domains in the complete gene sets of 15 insect species. These included 5 beetles: AGLAB *Anoplophora glabripennis*, DPOND *Dendroctonus ponderosae*, TCAST *Tribolium castaneum*, APLAN *Agrilus planipennis*, and OTAUR *Onthophagus taurus*; 5 basal insects: ZNEVA *Zootermopsis nevadensis*, PHUMA *Pediculus humanus*, APISU *Acyrthosiphon pisum*, AMELL *Apis mellifera*, and NVITR *Nasonia vitripennis*; and 2 lepidopterans: PXYLO *Plutella xylostella*, and DPLEX *Danaus plexippus*; and 3 dipterans: MDEST *Mayetiola destructor*, DMELA *Drosophila melanogaster*, and AGAMB *Anopheles gambiae*. Protein domains were annotated with InterProScan5 [141] using the following domain libraries: PfamA-27.0, PrositeProfiles-20.97, SMART-6.2, SuperFamily-1.75, and PRINTS-42.0. The families examined included glycoside hydrolases, peptidases, esterases, cytochrome P450s, and UDP-glucosyltransferases. The classifications based on InterPro domain counts were used only for those where the maximum gene count in a given species was greater than 5 (i.e. at least one species has a potential expansion of more than 5 genes). The orthology status of each of these identified genes was assessed using OrthoDB v8 [29] to determine if the gene was found as a single-copy ortholog, or with co-orthologs, or whether it showed homology to the domain but was not classified in any orthologous group. The results of the counts of each relevant domain type and the orthology status for the identified genes are given in Tables S17-26. Domains were selected for plotting from the complete list to avoid redundant domains (e.g. sub-families rather than families, and just one of N/C-terminal domains). For each gene family, bar charts were plotted with largest sub-family at the bottom and smallest at the top, showing the counts for each sub-family per species (Figs. S18-S22). The orthology status of genes in the sub-family bar charts (i.e. those plotted and where at least one species has >5 genes) show the totals in each species partitioned into single-copy and multi-copy orthologs, and homologs (Figs. S18-S22).

**Results and Discussion**

*See the below figures (and associated captions) and tables.*

**Figure S18 (Fig. 6, main paper). Orthology counts (a) and sub-family sizes (b) for gycoside hydrolases.** Among the examined species, *A. glabripennis* showed the most genes with matches to glycoside hydrolase (GH) domains, the majority of which were found as multi-copy orthologs. This elevated gene count was mainly due to GH Family 1 (IPR001360), members of which exhibit beta-glucosidase, beta-galactosidase, 6-phospho-beta-galactosidase, 6-phospho-beta-glucosidase, lactase-phlorizin hydrolase, beta-mannosidase, and myrosinase activities. Uniquely among the examined species, 6 *A. glabripennis* genes matched GH Family 5 (IPR001547), also known as cellulase family A, whose members exhibit endoglucanase, beta-mannanase, exo-1,3-glucanase, endo-1,6-glucanase, xylanase, and endoglycoceramidase activities. *A. glabripennis* also had 2 matches to the GH Family 45 (IPR000334, endoglucanase activity) also known as cellulase family K, which was also found in *Dendroctonus ponderosae* (9 copies). Members of GH Family 28 (IPR000743) are pectinases that exhibit polygalacturonase and rhamnogalacturonase activities, and had matches to 16 genes in *A. glabripennis* (18 were identified by manual annotation; 19 were reported in [79]), 16 in *D. ponderosae* and 7 in *A. planipennis* (50 were manually annotated).

**(a) orthology**

***
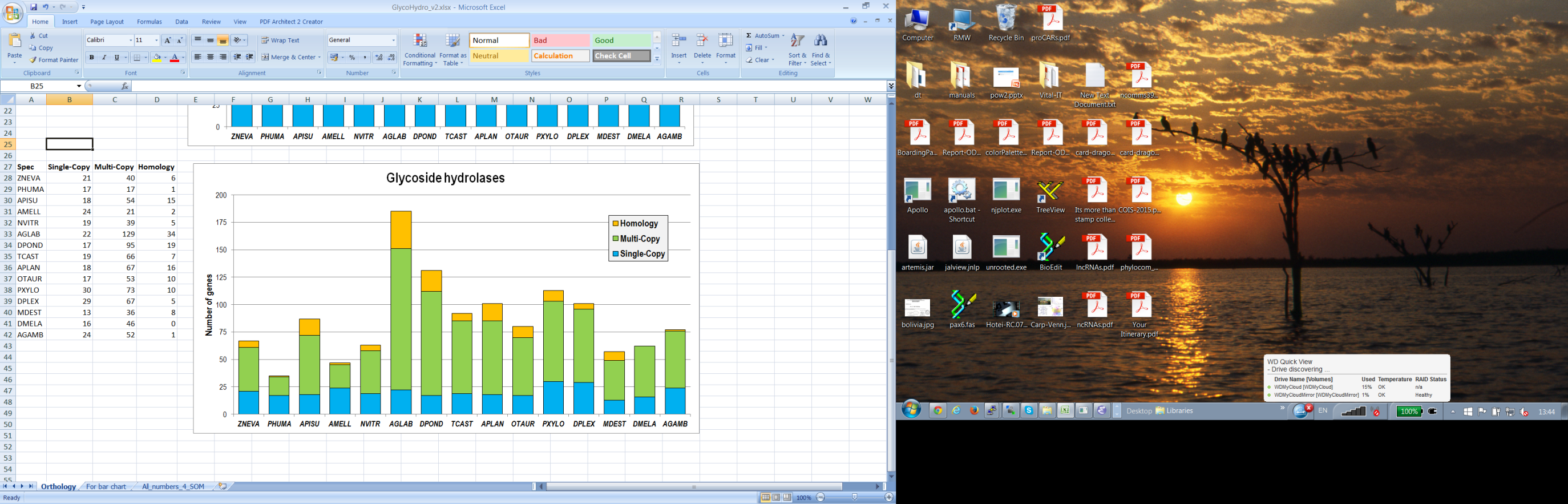
***

**(b) sub-family sizes**


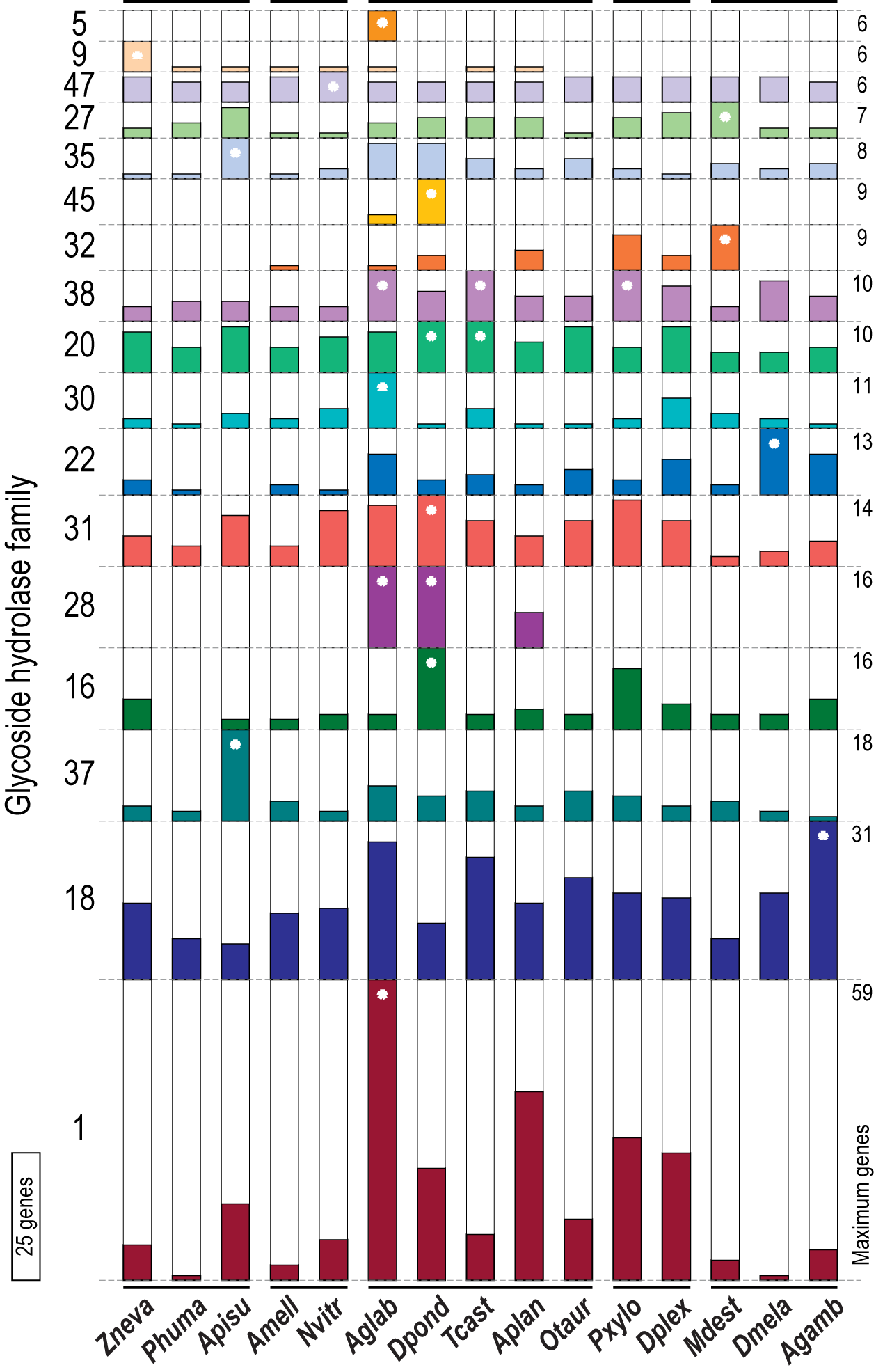


*Species with the maximum gene count are indicated with a white asterisk.***Figure S19. Orthology counts (a) and sub-family sizes (b) for peptidases.** The total numbers of genes matching peptidase C (cysteine), M (metallo), and S (serine) domains varies from about 230 for *Pediculus humanus* and *Apis mellifera* to more than 550 for *Plutella xylostella* and *Anopheles gambiae*. With 435 matches, *A. glabripennis* showed no overall expansion of these peptidase families as this was only slightly more than the average (392). The total number of serine proteinases containing CLIP domains (likely involved in immune function), identified by matching the InterPro profile IPR022700 (Proteinase, regulatory CLIP domain), varied from just 2 genes in *Pediculus humanus* to 25 in *Anopheles gambiae* and 24 in *A. glabripennis*, and the number of serine proteinases lacking these domains (likely involved in digestion) varied from 90 in Apis mellifera to 340 in Anopheles gambiae. Overall, there was not a greater number of digestive related serine proteinases in polyphages. Nevertheless, *A. glabripennis* did show relatively large, or occasionally the largest number of matches to several sub-families. It had the second highest number of matches to C1A (IPR000668) papain peptidases (35), which contain both endo- and exopeptidases and are found in protozoan digestive vacuoles and eukaryotic lysosomes. It also showed relatively high numbers of matches to C2 (IPR001300) calpain peptidases. Among the metallo-peptidases it had the highest numbers of matches to M10 (IPR001818) peptidases (15) known as matrixins, which are usually secreted and function in the degradation of matrix proteins in connective tissue. It also had slightly above average numbers of matches to M13 (IPR008753) neprilysin peptidases (17), which are usually secreted to degrade or convert polypeptides. The sub-family of S1 (IPR009003) serine endopeptidases, which include chymotrypsins and trypsins, is the largest of all of the peptidase families, and *A. glabripennis* (186) had just over half the maximum number of matches found in *Anopheles gambiae* (332). It showed the highest number of matches to S54 (IPR022764) peptidases (rhomboid-like proteins), which include membrane-bound serine endopeptidases, and an above average number of matches to S28 (IPR008758) peptidases, which are exopeptidases that hydrolyse prolyl bonds.

**(a) orthology**

***
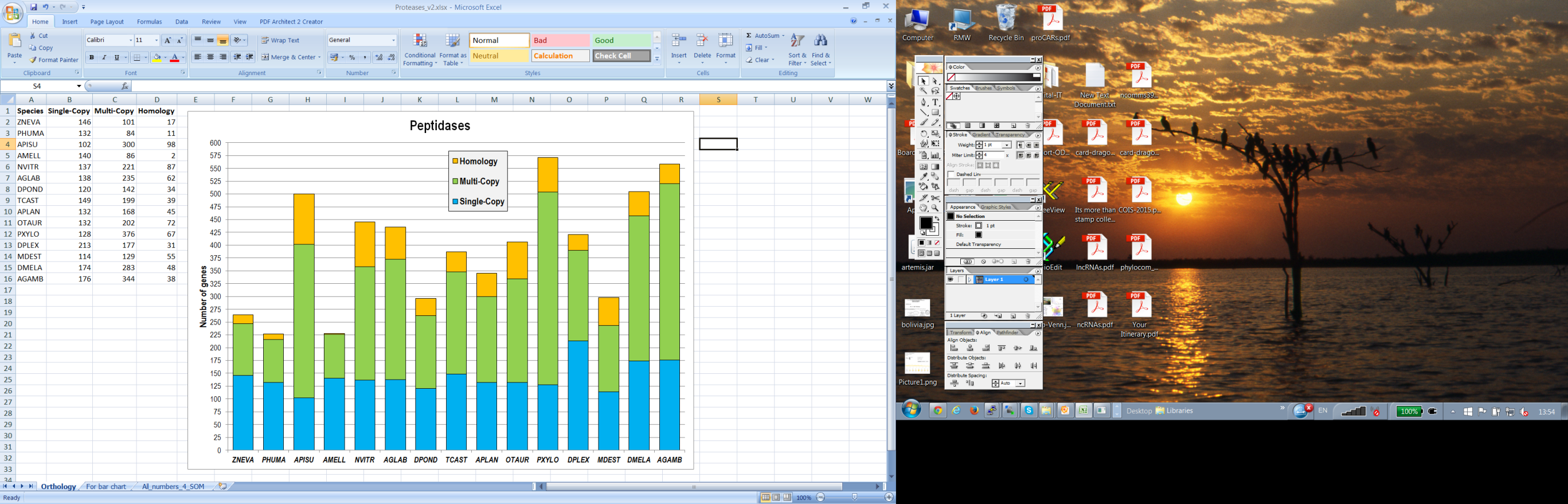
***

**(b) sub-family sizes**


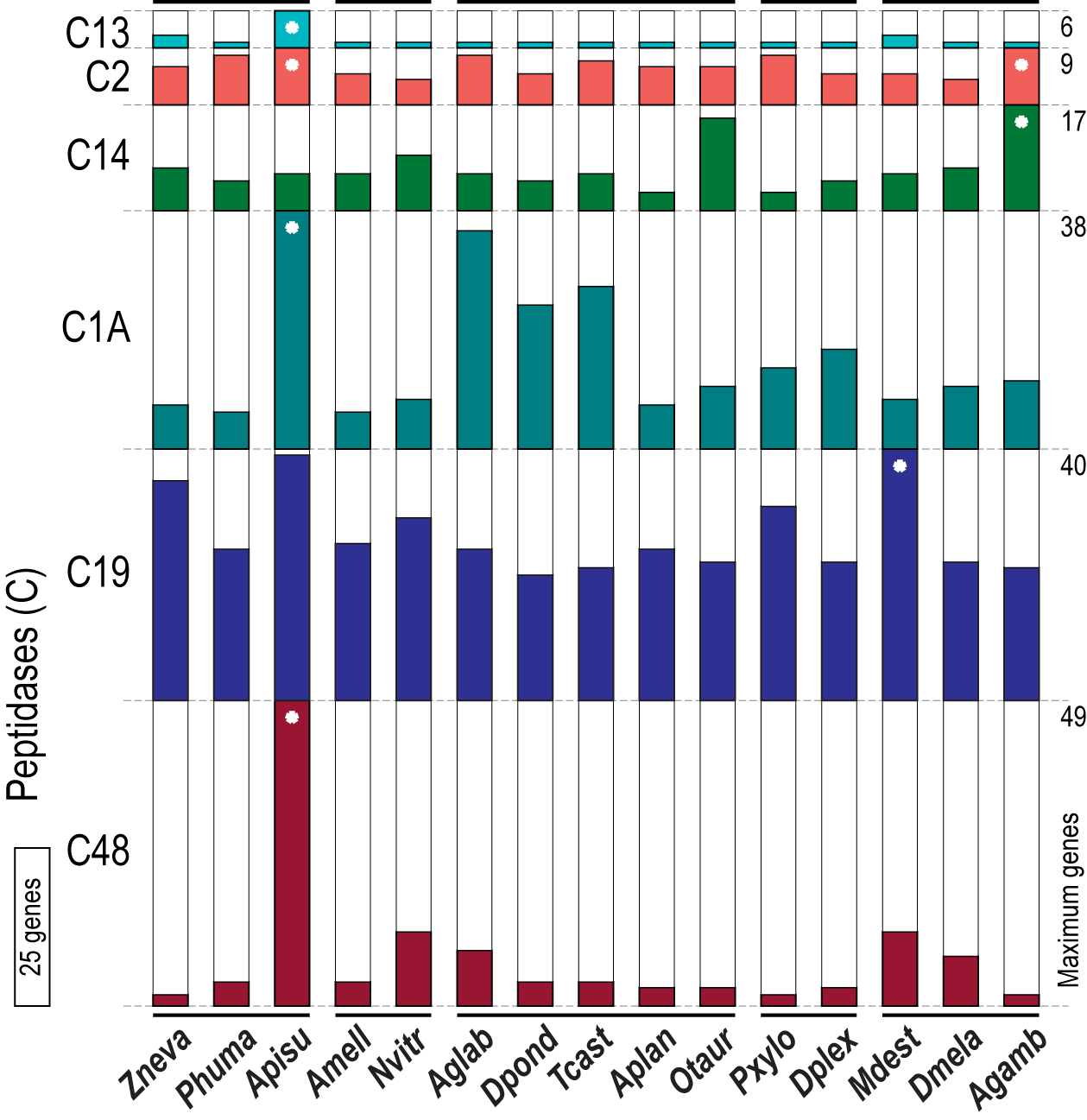


*Species with the maximum gene count are indicated with a white asterisk.*

**(b) sub-family sizes (continued)**


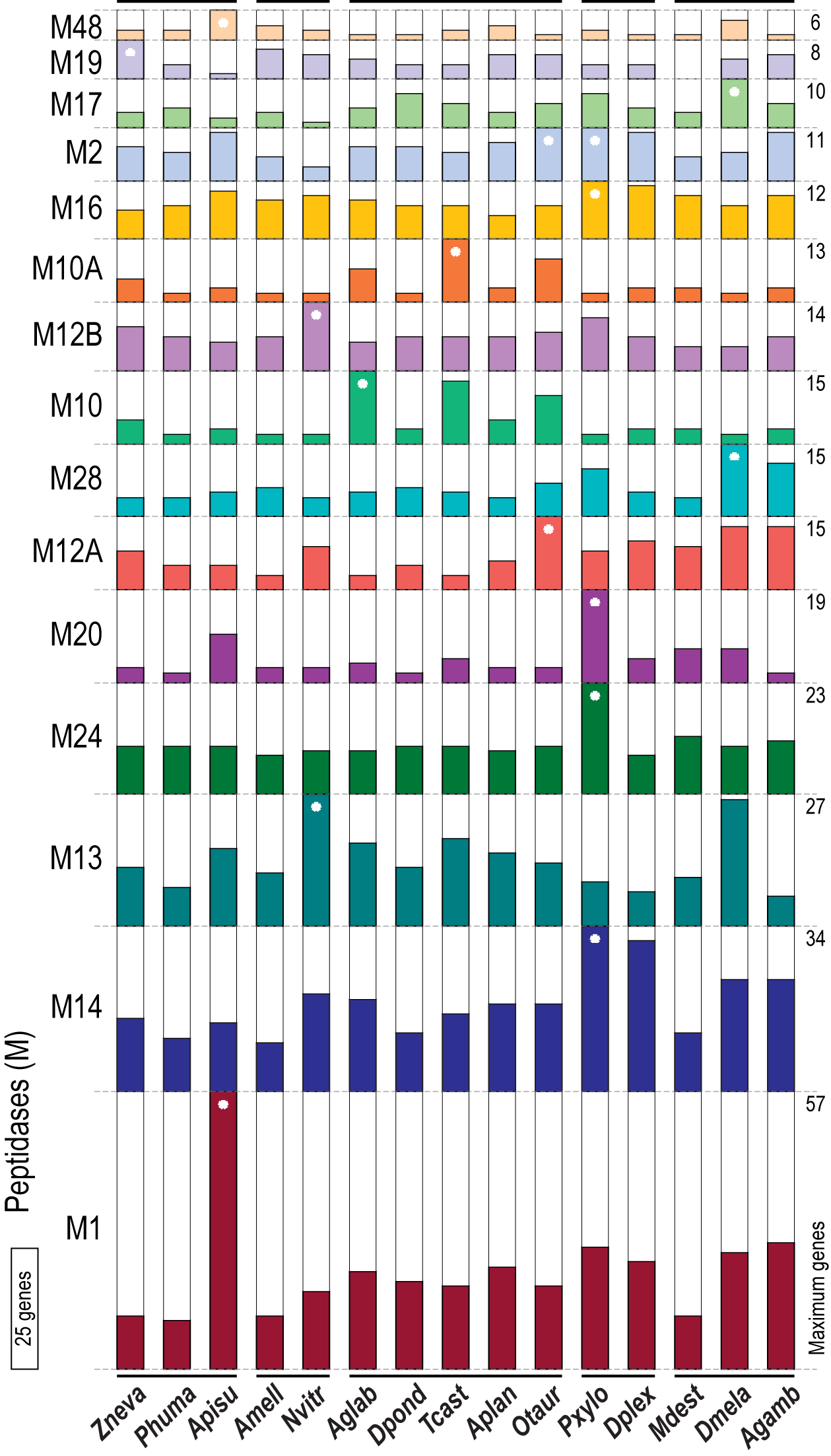


*Species with the maximum gene count are indicated with a white asterisk.*

**(b) sub-family sizes (continued)**


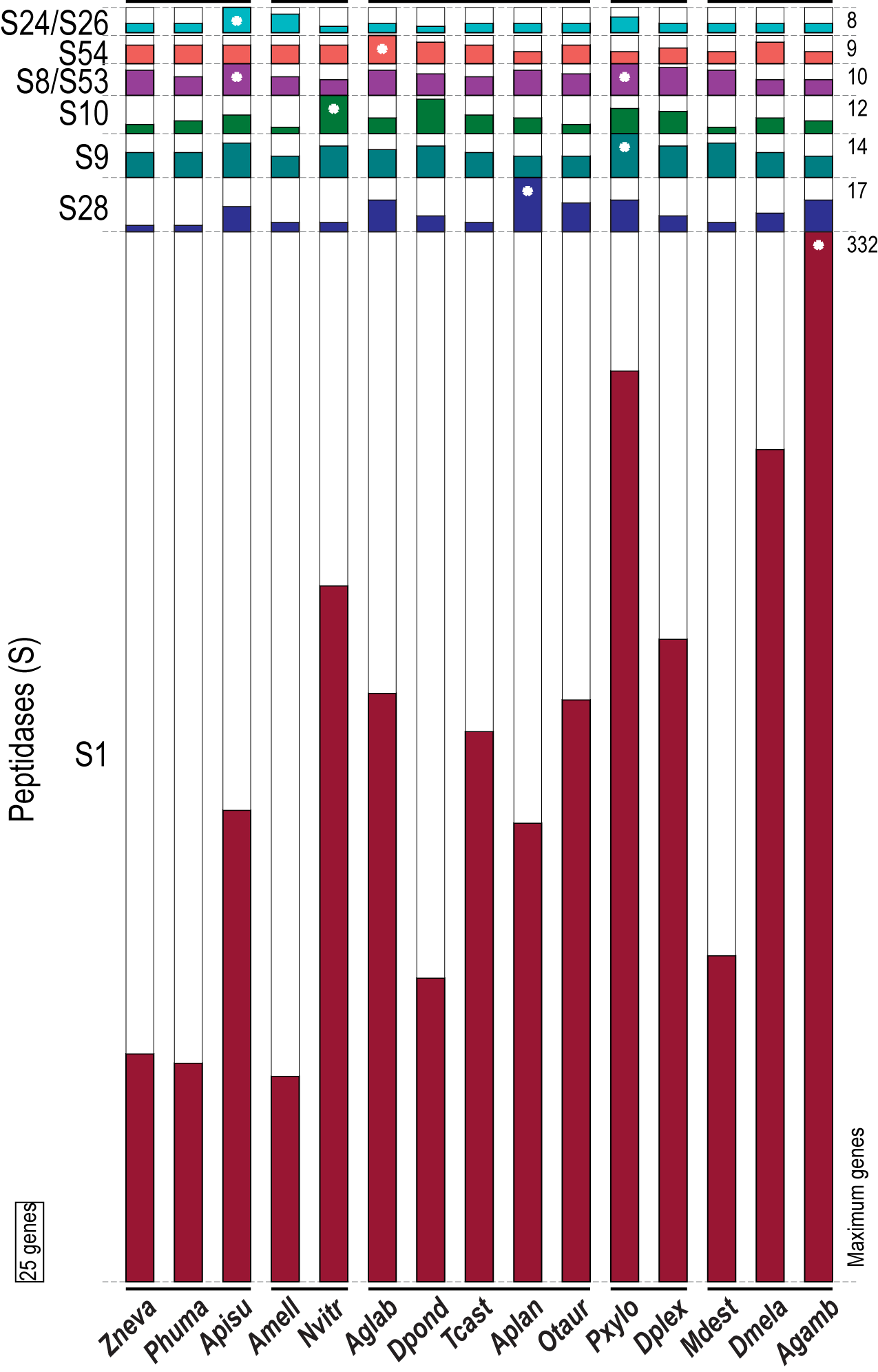


*Species with the maximum gene count are indicated with a white asterisk.*

**Figure S20. Orthology counts (a) and sub-family sizes (b) for esterases.** *A. glabripennis* showed the highest number of genes matching esterase domains among the examined species, the majority of which were found as multi-copy orthologs. This is due

mainly to a large expansion of type-B carboxylesterases (IPR002018), of which *A. glabripennis* had more than double (105) the average across the other 14 species (49). Carboxylesterases, most of which are serine hydrolases, act on carboxylic esters and are important for the metabolism of xenobiotics. Although a much smaller gene family, *A. glabripennis* also showed the highest number of genes (8) matching the thioesterase domain (IPR001031). Genes matching the pectinesterase domain (IPR000070), which is from cell-wall-associated enzymes that act in plant cell wall modification processes and subsequent breakdown, were found in *Dendroctonus ponderosae* (13) and *Plutella xylostella* (1).

**(a) orthology**

***
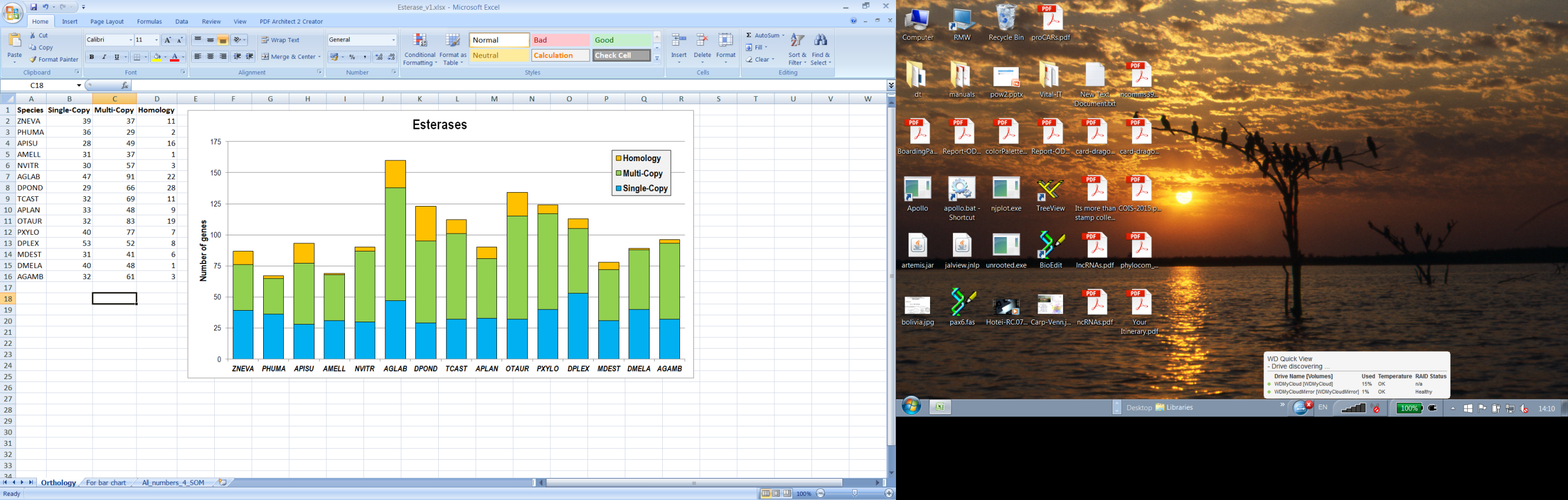
***

**(b) sub-family sizes**


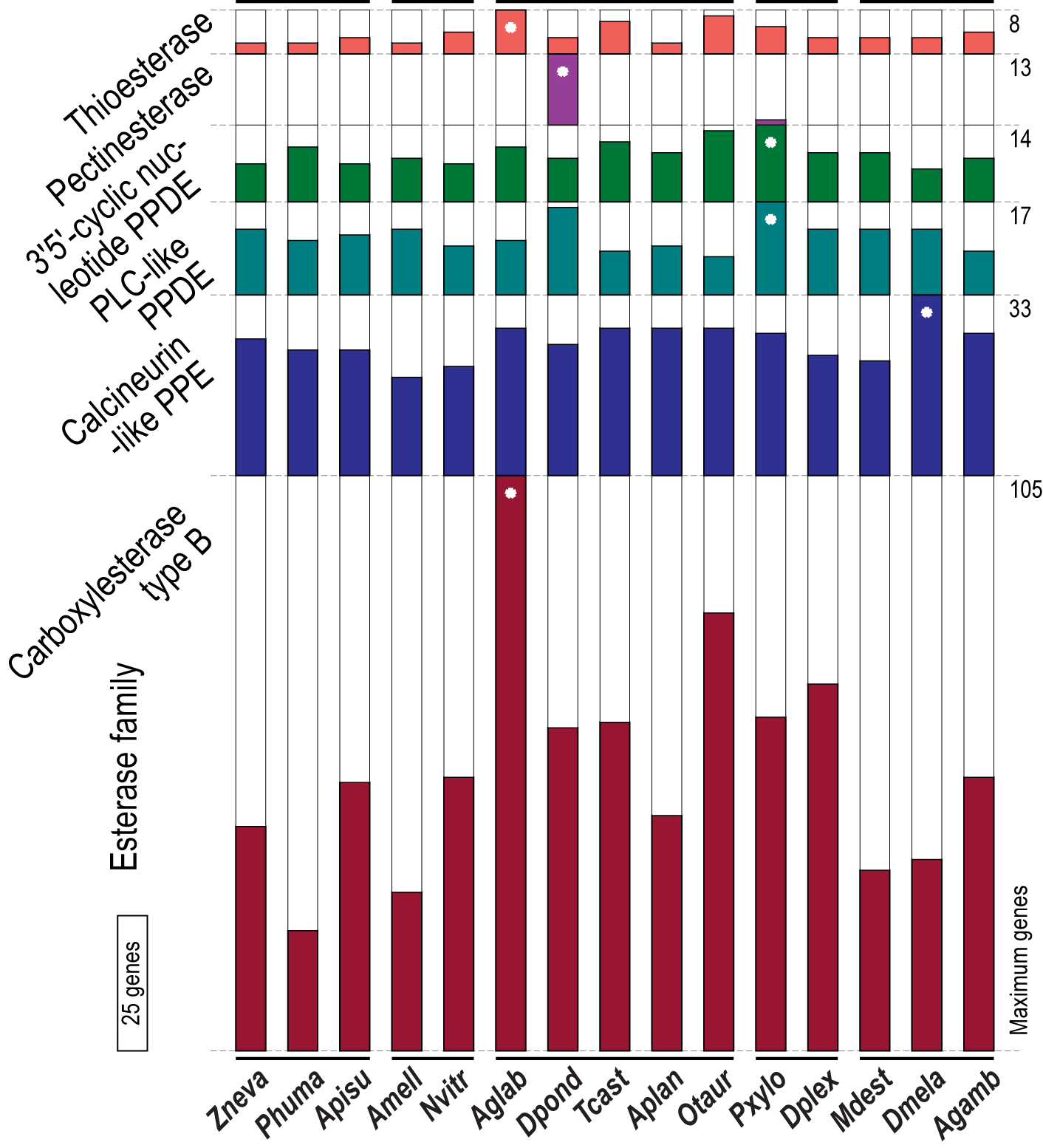


*Species with the maximum gene count are indicated with a white asterisk.*

**Figure S21. Orthology counts (a) and sub-family sizes (b) for cytochrome P450s.** Among the examined species, *A. glabripennis* showed the third highest number of genes with matches to Cytochrome P450 (CYP450) domains after *Tribolium castaneum* and *Onthophagus taurus*, the majority of which were found as multi-copy orthologs. However, examining the CYP450 sub-families showed that while Group I (IPR002401) matches were slightly above average, *A. glabripennis* showed the most matches to Group II (IPR002402) and Group IV (IPR002403) CYP450s. Strikingly, *A. glabripennis* had five times as many Group II matching genes (18) than the average across the other 14 species (3.6). These enzymes include insect CYP4 and CYP6 types where CYP6 ezymes are known to metabolize a wide range of toxic compounds.

**(a) orthology**

***
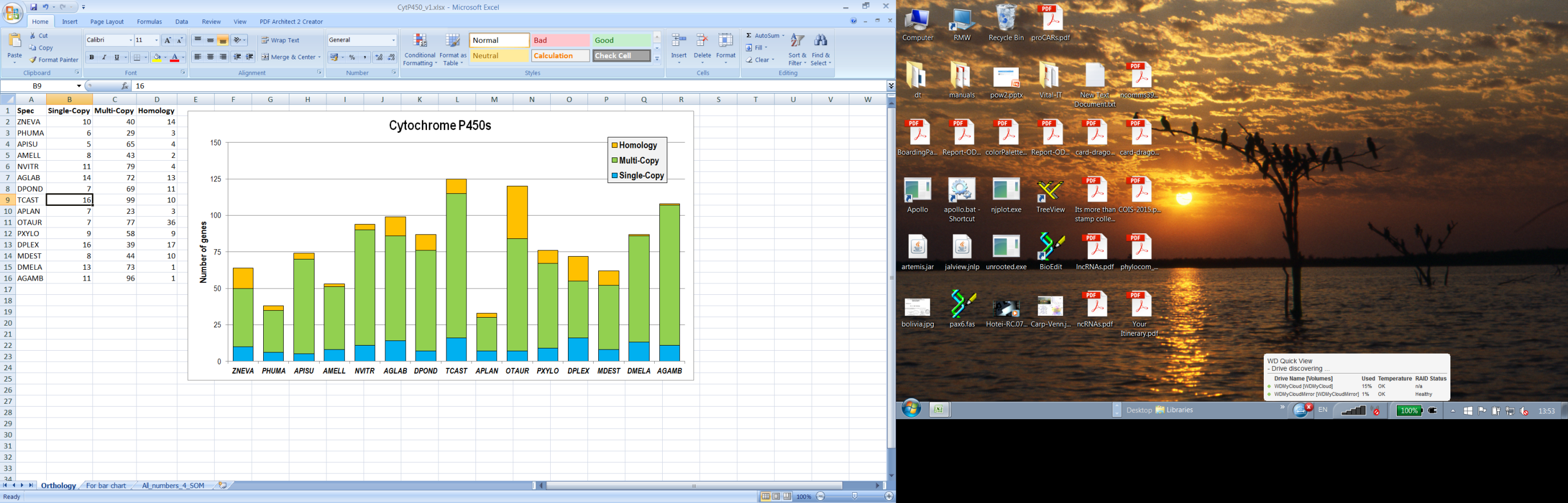
***

**(b) sub-family sizes**


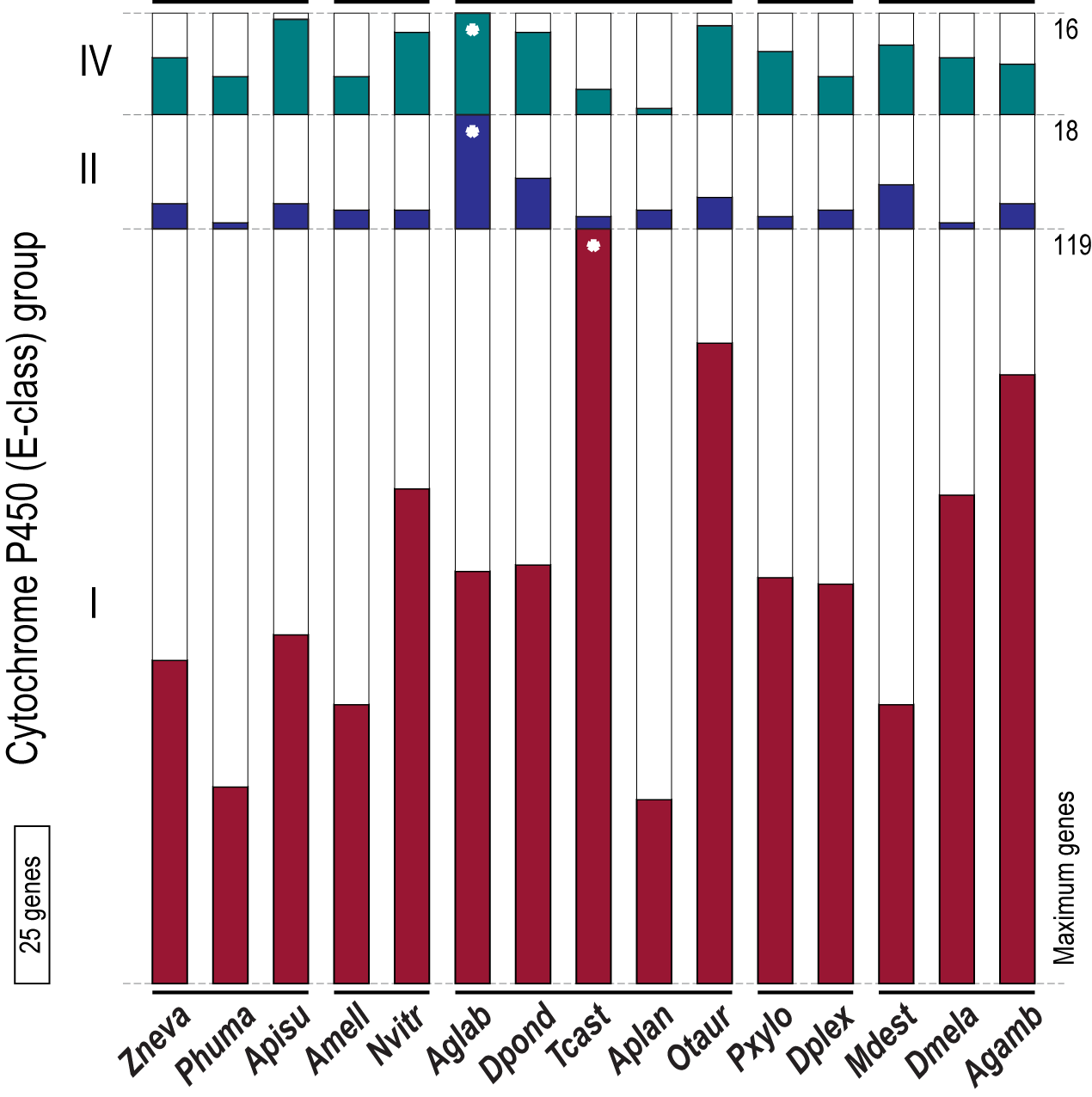


*Species with the maximum gene count are indicated with a white asterisk.*

**Figure S22. Orthology counts (a) and sub-family sizes (b) for UDP-glycosyltransferases.** *A. glabripennis* had the second highest number of matches (49) to the UDP-glucuronosyl/UDP-glucosyltransferase domain (IPR002213) after *Acyrthosiphon pisum* (72). This was about double the average number of matches from the other 13 species (24). These enzymes catalyze the addition of the glycosyl group from a UTP-sugar to a small hydrophobic molecule.

**(a) orthology**

***
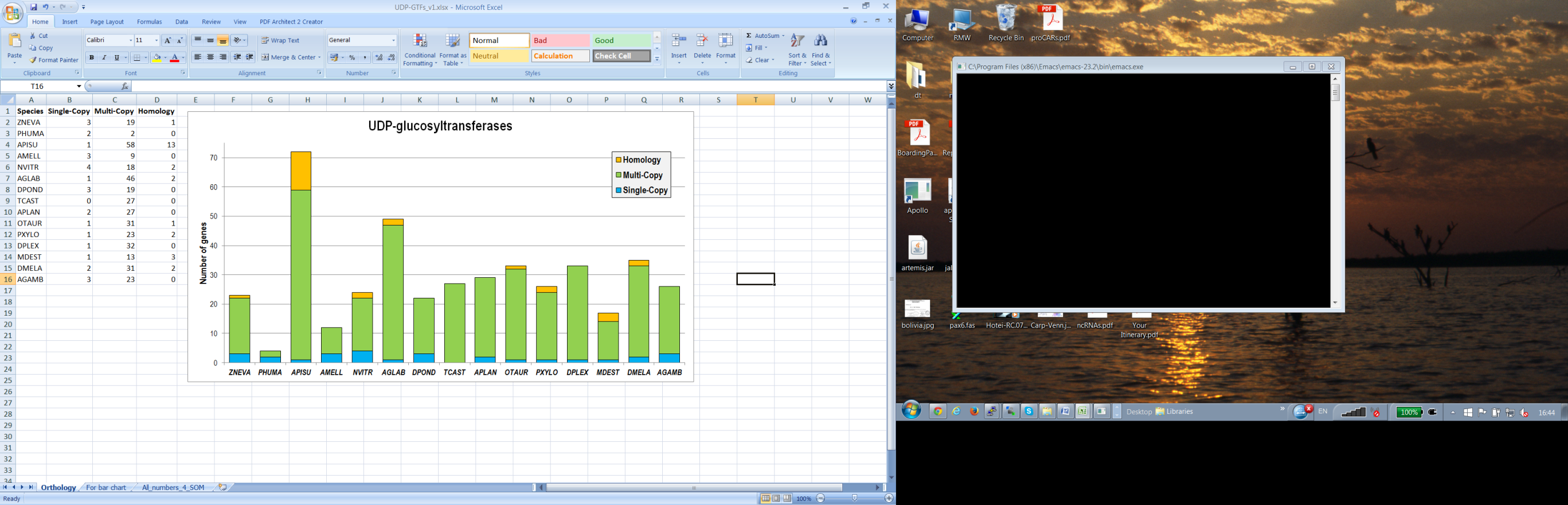
***

**(b) sub-family sizes**


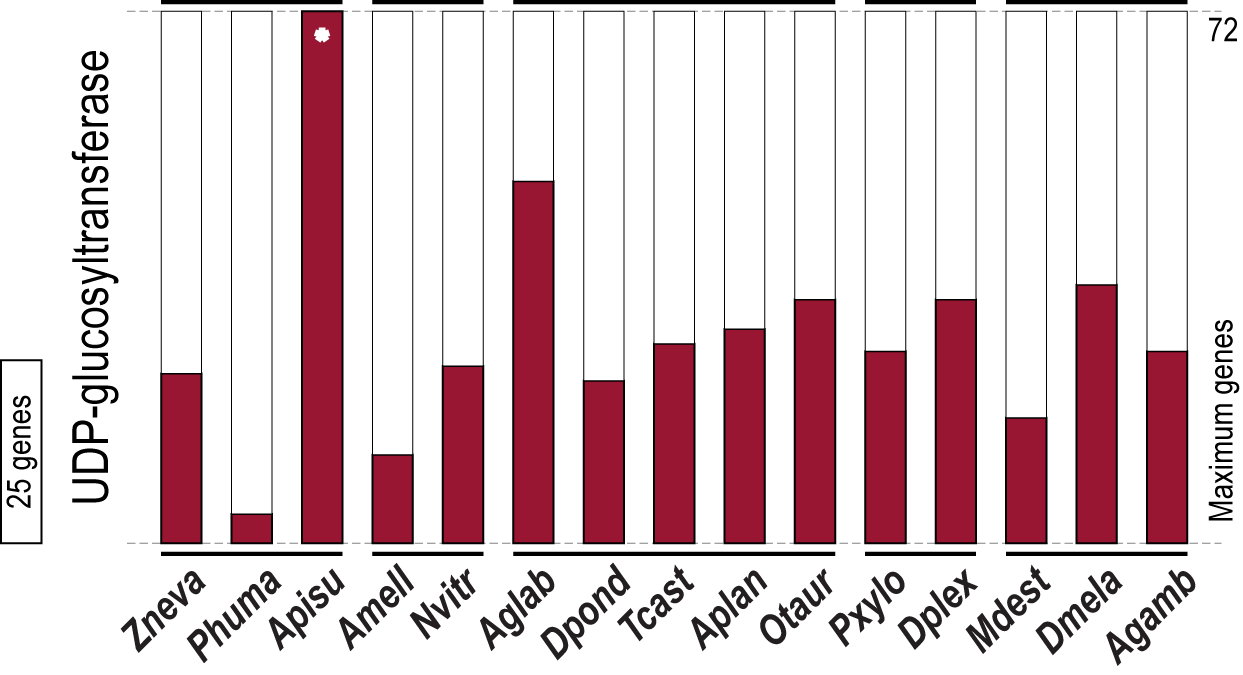


*Species with the maximum gene count are indicated with a white asterisk.*

**Table S17. Orthology counts for gycoside hydrolases.**

| **Species** | **Single-Copy** | **Multi-Copy** | **Homology** |
| --- | --- | --- | --- |
| ZNEVA | 21 | 40 | 6 |
| PHUMA | 17 | 17 | 1 |
| APISU | 18 | 54 | 15 |
| AMELL | 24 | 21 | 2 |
| NVITR | 19 | 39 | 5 |
| AGLAB | 22 | 129 | 34 |
| DPOND | 17 | 95 | 19 |
| TCAST | 19 | 66 | 7 |
| APLAN | 18 | 67 | 16 |
| OTAUR | 17 | 53 | 10 |
| PXYLO | 30 | 73 | 10 |
| DPLEX | 29 | 67 | 5 |
| MDEST | 13 | 36 | 8 |
| DMELA | 16 | 46 | 0 |
| AGAMB | 24 | 52 | 1 |

**Table S18. Orthology counts for peptidases**.

| **Species** | **Single-Copy** | **Multi-Copy** | **Homology** |
| --- | --- | --- | --- |
| ZNEVA | 146 | 101 | 17 |
| PHUMA | 132 | 84 | 11 |
| APISU | 102 | 300 | 98 |
| AMELL | 140 | 86 | 2 |
| NVITR | 137 | 221 | 87 |
| AGLAB | 138 | 235 | 62 |
| DPOND | 120 | 142 | 34 |
| TCAST | 149 | 199 | 39 |
| APLAN | 132 | 168 | 45 |
| OTAUR | 132 | 202 | 72 |
| PXYLO | 128 | 376 | 67 |
| DPLEX | 213 | 177 | 31 |
| MDEST | 114 | 129 | 55 |
| DMELA | 174 | 283 | 48 |
| AGAMB | 176 | 344 | 38 |

**Table S19. Orthology counts for esterases.**

| **Species** | **Single-Copy** | **Multi-Copy** | **Homology** |
| --- | --- | --- | --- |
| ZNEVA | 39 | 37 | 11 |
| PHUMA | 36 | 29 | 2 |
| APISU | 28 | 49 | 16 |
| AMELL | 31 | 37 | 1 |
| NVITR | 30 | 57 | 3 |
| AGLAB | 47 | 91 | 22 |
| DPOND | 29 | 66 | 28 |
| TCAST | 32 | 69 | 11 |
| APLAN | 33 | 48 | 9 |
| OTAUR | 32 | 83 | 19 |
| PXYLO | 40 | 77 | 7 |
| DPLEX | 53 | 52 | 8 |
| MDEST | 31 | 41 | 6 |
| DMELA | 40 | 48 | 1 |
| AGAMB | 32 | 61 | 3 |

**Table S20. Orthology counts for Cytochrome P450s.**

| **Species** | **Single-Copy** | **Multi-Copy** | **Homology** |
| --- | --- | --- | --- |
| ZNEVA | 10 | 40 | 14 |
| PHUMA | 6 | 29 | 3 |
| APISU | 5 | 65 | 4 |
| AMELL | 8 | 43 | 2 |
| NVITR | 11 | 79 | 4 |
| AGLAB | 14 | 72 | 13 |
| DPOND | 7 | 69 | 11 |
| TCAST | 16 | 99 | 10 |
| APLAN | 7 | 23 | 3 |
| OTAUR | 7 | 77 | 36 |
| PXYLO | 9 | 58 | 9 |
| DPLEX | 16 | 39 | 17 |
| MDEST | 8 | 44 | 10 |
| DMELA | 13 | 73 | 1 |
| AGAMB | 11 | 96 | 1 |

**Table S21. Orthology counts for UDP-glycosyltransferases.**

| **Species** | **Single-Copy** | **Multi-Copy** | **Homology** |
| --- | --- | --- | --- |
| ZNEVA | 3 | 19 | 1 |
| PHUMA | 2 | 2 | 0 |
| APISU | 1 | 58 | 13 |
| AMELL | 3 | 9 | 0 |
| NVITR | 4 | 18 | 2 |
| AGLAB | 1 | 46 | 2 |
| DPOND | 3 | 19 | 0 |
| TCAST | 0 | 27 | 0 |
| APLAN | 2 | 27 | 0 |
| OTAUR | 1 | 31 | 1 |
| PXYLO | 1 | 23 | 2 |
| DPLEX | 1 | 32 | 0 |
| MDEST | 1 | 13 | 3 |
| DMELA | 2 | 31 | 2 |
| AGAMB | 3 | 23 | 0 |

**Table S22. InterPro domain counts for glycoside hydrolases.**

| **IPR** | **IPR_name** | **ZNEVA** | **PHUMA** | **APISU** | **AMELL** | **NVITR** | **AGLAB** | **DPOND** | **TCAST** | **APLAN** | **OTAUR** | **PXYLO** | **DPLEX** | **MDEST** | **DMELA** | **AGAMB** | **Total** | **Max** |
| --- | --- | --- | --- | --- | --- | --- | --- | --- | --- | --- | --- | --- | --- | --- | --- | --- | --- | --- |
| IPR017853 | Glycoside hydrolase superfamily | 65 | 40 | 110 | 51 | 73 | 153 | 86 | 92 | 101 | 69 | 97 | 95 | 57 | 54 | 82 | 1225 | 153 |
| IPR001360 | Glycoside hydrolase, family 1 | 7 | 1 | 15 | 3 | 8 | 59 | 22 | 9 | 37 | 12 | 28 | 25 | 4 | 1 | 6 | 237 | 59 |
| IPR001223 | Glycoside hydrolase family 18, catalytic domain | 15 | 8 | 7 | 13 | 14 | 27 | 11 | 24 | 15 | 20 | 17 | 16 | 8 | 17 | 31 | 243 | 31 |
| IPR011330 | Glycoside hydrolase/deacetylase, beta/alpha-barrel | 8 | 8 | 14 | 8 | 8 | 18 | 14 | 19 | 10 | 15 | 23 | 13 | 8 | 15 | 11 | 192 | 23 |
| IPR001661 | Glycoside hydrolase, family 37 | 3 | 2 | 18 | 4 | 2 | 7 | 5 | 6 | 3 | 6 | 5 | 3 | 4 | 2 | 1 | 71 | 18 |
| IPR000757 | Glycoside hydrolase, family 16 | 6 | 0 | 2 | 2 | 3 | 3 | 16 | 3 | 4 | 3 | 12 | 5 | 3 | 3 | 6 | 71 | 16 |
| IPR000743 | Glycoside hydrolase, family 28 | 0 | 0 | 0 | 0 | 0 | 16 | 16 | 0 | 7 | 0 | 0 | 0 | 0 | 0 | 0 | 39 | 16 |
| IPR000322 | Glycoside hydrolase family 31 | 6 | 4 | 10 | 4 | 11 | 12 | 14 | 9 | 6 | 9 | 13 | 9 | 2 | 3 | 5 | 117 | 14 |
| IPR001916 | Glycoside hydrolase, family 22 | 3 | 1 | 0 | 2 | 1 | 8 | 3 | 4 | 2 | 5 | 3 | 7 | 2 | 13 | 8 | 62 | 13 |
| IPR001139 | Glycoside hydrolase, family 30 | 2 | 1 | 3 | 2 | 4 | 11 | 1 | 4 | 1 | 1 | 2 | 6 | 3 | 2 | 1 | 44 | 11 |
| IPR015883 | Glycoside hydrolase family 20, catalytic domain | 8 | 5 | 9 | 5 | 7 | 8 | 10 | 10 | 6 | 9 | 5 | 9 | 4 | 4 | 5 | 104 | 10 |
| IPR015341 | Glycoside hydrolase, family 38, central domain | 3 | 4 | 4 | 3 | 3 | 8 | 7 | 10 | 5 | 4 | 7 | 4 | 3 | 8 | 5 | 78 | 10 |
| IPR028995 | Glycoside hydrolase, families 57/38, central domain | 3 | 4 | 4 | 3 | 3 | 8 | 7 | 10 | 5 | 4 | 7 | 4 | 2 | 8 | 5 | 77 | 10 |
| IPR000602 | Glycoside hydrolase family 38, N-terminal domain | 3 | 4 | 4 | 3 | 3 | 10 | 6 | 10 | 5 | 5 | 10 | 7 | 3 | 8 | 5 | 86 | 10 |
| IPR001362 | Glycoside hydrolase, family 32 | 0 | 0 | 0 | 1 | 0 | 1 | 3 | 0 | 4 | 0 | 7 | 3 | 9 | 0 | 0 | 28 | 9 |
| IPR000334 | Glycoside hydrolase, family 45 | 0 | 0 | 0 | 0 | 0 | 2 | 9 | 0 | 0 | 0 | 0 | 0 | 0 | 0 | 0 | 11 | 9 |
| IPR000974 | Glycoside hydrolase, family 22, lysozyme | 2 | 0 | 0 | 0 | 0 | 0 | 0 | 0 | 0 | 0 | 1 | 1 | 1 | 9 | 7 | 21 | 9 |
| IPR001944 | Glycoside hydrolase, family 35 | 1 | 1 | 8 | 1 | 2 | 7 | 7 | 4 | 2 | 4 | 2 | 1 | 3 | 2 | 3 | 48 | 8 |
| IPR025887 | Glycoside hydrolase family 31, N-terminal domain | 2 | 3 | 3 | 2 | 2 | 8 | 4 | 5 | 3 | 4 | 3 | 3 | 1 | 1 | 1 | 45 | 8 |
| IPR002241 | Glycoside hydrolase, family 27 | 2 | 3 | 6 | 1 | 1 | 3 | 4 | 4 | 4 | 1 | 4 | 5 | 7 | 2 | 2 | 49 | 7 |
| IPR001547 | Glycoside hydrolase, family 5 | 0 | 0 | 0 | 0 | 0 | 6 | 0 | 0 | 0 | 0 | 0 | 0 | 0 | 0 | 0 | 6 | 6 |
| IPR001701 | Glycoside hydrolase, family 9 | 6 | 1 | 1 | 1 | 1 | 1 | 0 | 1 | 1 | 0 | 0 | 0 | 0 | 0 | 0 | 13 | 6 |
| IPR000111 | Glycoside hydrolase family 27/36, conserved site | 2 | 3 | 6 | 1 | 1 | 3 | 1 | 4 | 2 | 1 | 3 | 3 | 5 | 2 | 2 | 39 | 6 |
| IPR001382 | Glycoside hydrolase, family 47 | 5 | 4 | 4 | 5 | 6 | 4 | 4 | 4 | 4 | 5 | 5 | 5 | 5 | 5 | 4 | 69 | 6 |

**Table S23. InterPro domain counts for peptidases.**

| **IPR** | **IPR_name** | **ZNEVA** | **PHUMA** | **APISU** | **AMELL** | **NVITR** | **AGLAB** | **DPOND** | **TCAST** | **APLAN** | **OTAUR** | **PXYLO** | **DPLEX** | **MDEST** | **DMELA** | **AGAMB** | **Total** | **Max** |
| --- | --- | --- | --- | --- | --- | --- | --- | --- | --- | --- | --- | --- | --- | --- | --- | --- | --- | --- |
| IPR009003 | Peptidase S1, PA clan | 72 | 69 | 149 | 65 | 220 | 186 | 96 | 174 | 145 | 184 | 288 | 203 | 103 | 263 | 332 | 2549 | 332 |
| IPR001254 | Serine proteases, trypsin domain | 70 | 67 | 133 | 62 | 217 | 179 | 93 | 167 | 140 | 180 | 272 | 185 | 97 | 257 | 327 | 2446 | 327 |
| IPR001314 | Peptidase S1A, chymotrypsin-type | 53 | 50 | 75 | 54 | 175 | 134 | 75 | 147 | 98 | 145 | 180 | 119 | 63 | 227 | 257 | 1852 | 257 |
| IPR021109 | Aspartic peptidase domain | 8 | 2 | 227 | 2 | 72 | 89 | 5 | 62 | 30 | 35 | 47 | 6 | 37 | 14 | 2 | 638 | 227 |
| IPR008737 | Peptidase aspartic, putative | 1 | 0 | 176 | 0 | 17 | 71 | 3 | 12 | 58 | 19 | 21 | 0 | 39 | 0 | 0 | 417 | 176 |
| IPR018061 | Peptidase A2A, retrovirus RVP subgroup | 1 | 0 | 87 | 0 | 13 | 22 | 1 | 18 | 11 | 14 | 10 | 2 | 19 | 0 | 0 | 198 | 87 |
| IPR014782 | Peptidase M1, membrane alanine aminopeptidase, N-terminal | 11 | 10 | 57 | 11 | 16 | 20 | 18 | 17 | 21 | 17 | 25 | 22 | 11 | 24 | 26 | 306 | 57 |
| IPR003653 | Ulp1 protease family, C-terminal catalytic domain | 2 | 4 | 49 | 4 | 12 | 9 | 4 | 4 | 3 | 3 | 2 | 3 | 12 | 8 | 2 | 121 | 49 |
| IPR001394 | Peptidase C19, ubiquitin carboxyl-terminal hydrolase | 35 | 24 | 39 | 25 | 29 | 24 | 20 | 21 | 24 | 22 | 31 | 22 | 40 | 22 | 21 | 399 | 40 |
| IPR000668 | Peptidase C1A, papain C-terminal | 7 | 6 | 38 | 6 | 8 | 35 | 23 | 26 | 7 | 10 | 13 | 16 | 8 | 10 | 11 | 224 | 38 |
| IPR000834 | Peptidase M14, carboxypeptidase A | 15 | 11 | 14 | 10 | 20 | 19 | 12 | 16 | 18 | 18 | 34 | 31 | 12 | 23 | 23 | 276 | 34 |
| IPR008753 | Peptidase M13, N-terminal domain | 12 | 8 | 16 | 11 | 27 | 17 | 12 | 18 | 15 | 13 | 9 | 7 | 10 | 26 | 6 | 207 | 27 |
| IPR018497 | Peptidase M13, C-terminal domain | 11 | 7 | 15 | 11 | 26 | 14 | 14 | 17 | 10 | 10 | 7 | 5 | 10 | 23 | 5 | 185 | 26 |
| IPR006026 | Peptidase, metallopeptidase | 12 | 7 | 7 | 5 | 10 | 10 | 5 | 16 | 8 | 24 | 7 | 10 | 11 | 15 | 16 | 163 | 24 |
| IPR000994 | Peptidase M24, structural domain | 10 | 10 | 10 | 8 | 9 | 9 | 10 | 10 | 9 | 10 | 23 | 8 | 12 | 10 | 11 | 159 | 23 |
| IPR002933 | Peptidase M20 | 3 | 2 | 10 | 3 | 3 | 4 | 2 | 5 | 3 | 3 | 19 | 5 | 7 | 7 | 2 | 78 | 19 |
| IPR011650 | Peptidase M20, dimerisation domain | 3 | 2 | 10 | 3 | 2 | 3 | 2 | 4 | 2 | 2 | 19 | 2 | 7 | 7 | 2 | 70 | 19 |
| IPR011600 | Peptidase C14, caspase domain | 7 | 5 | 6 | 6 | 9 | 6 | 5 | 6 | 3 | 15 | 3 | 5 | 6 | 7 | 17 | 106 | 17 |
| IPR015917 | Peptidase C14A, caspase precursor p45, core | 6 | 4 | 6 | 6 | 8 | 8 | 5 | 8 | 3 | 14 | 3 | 4 | 6 | 7 | 17 | 105 | 17 |
| IPR008758 | Peptidase S28 | 2 | 2 | 8 | 3 | 3 | 10 | 5 | 3 | 17 | 9 | 10 | 5 | 3 | 6 | 10 | 96 | 17 |
| IPR001506 | Peptidase M12A, astacin | 8 | 5 | 5 | 3 | 9 | 3 | 5 | 3 | 6 | 15 | 8 | 10 | 9 | 13 | 13 | 115 | 15 |
| IPR011249 | Metalloenzyme, LuxS/M16 peptidase-like | 6 | 7 | 10 | 8 | 9 | 10 | 7 | 7 | 8 | 7 | 15 | 14 | 10 | 7 | 9 | 134 | 15 |
| IPR007484 | Peptidase M28 | 4 | 4 | 5 | 6 | 4 | 5 | 6 | 5 | 4 | 7 | 10 | 5 | 4 | 15 | 11 | 95 | 15 |
| IPR000101 | Gamma-glutamyltranspeptidase | 4 | 3 | 15 | 3 | 6 | 5 | 3 | 4 | 3 | 6 | 9 | 4 | 13 | 4 | 4 | 86 | 15 |
| IPR001818 | Peptidase M10, metallopeptidase | 5 | 2 | 3 | 2 | 2 | 15 | 3 | 13 | 5 | 10 | 2 | 3 | 3 | 2 | 3 | 73 | 15 |
| IPR001375 | Peptidase S9, prolyl oligopeptidase, catalytic domain | 8 | 8 | 11 | 7 | 10 | 9 | 10 | 8 | 7 | 7 | 14 | 10 | 11 | 8 | 7 | 135 | 14 |
| IPR001590 | Peptidase M12B, ADAM/reprolysin | 9 | 7 | 6 | 7 | 14 | 6 | 7 | 7 | 7 | 8 | 11 | 7 | 5 | 5 | 7 | 113 | 14 |
| IPR001461 | Aspartic peptidase | 6 | 2 | 2 | 1 | 2 | 2 | 3 | 2 | 2 | 2 | 3 | 2 | 4 | 13 | 1 | 47 | 13 |
| IPR021190 | Peptidase M10A | 5 | 2 | 3 | 2 | 2 | 7 | 2 | 13 | 3 | 9 | 2 | 3 | 3 | 2 | 3 | 61 | 13 |
| IPR001563 | Peptidase S10, serine carboxypeptidase | 3 | 4 | 6 | 2 | 12 | 5 | 11 | 6 | 5 | 3 | 8 | 7 | 2 | 5 | 4 | 83 | 12 |
| IPR007863 | Peptidase M16, C-terminal | 6 | 7 | 10 | 8 | 9 | 8 | 7 | 7 | 5 | 7 | 12 | 11 | 9 | 7 | 9 | 122 | 12 |
| IPR002870 | Peptidase M12B, propeptide | 10 | 8 | 8 | 12 | 11 | 6 | 7 | 10 | 8 | 9 | 9 | 7 | 6 | 7 | 8 | 126 | 12 |
| IPR002469 | Dipeptidylpeptidase IV, N-terminal domain | 6 | 6 | 6 | 6 | 7 | 7 | 10 | 6 | 8 | 9 | 12 | 8 | 8 | 6 | 6 | 111 | 12 |
| IPR012599 | Peptidase C1A, propeptide | 1 | 0 | 11 | 0 | 0 | 8 | 4 | 6 | 1 | 1 | 1 | 1 | 1 | 1 | 1 | 37 | 11 |
| IPR011765 | Peptidase M16, N-terminal | 6 | 7 | 9 | 7 | 8 | 5 | 6 | 6 | 5 | 5 | 11 | 8 | 10 | 7 | 8 | 108 | 11 |
| IPR001548 | Peptidase M2, peptidyl-dipeptidase A | 7 | 6 | 10 | 5 | 3 | 7 | 7 | 6 | 8 | 11 | 11 | 10 | 5 | 6 | 10 | 112 | 11 |
| IPR012338 | Beta-lactamase/transpeptidase-like | 2 | 1 | 6 | 1 | 1 | 1 | 1 | 1 | 0 | 0 | 10 | 1 | 4 | 1 | 0 | 30 | 10 |
| IPR028980 | Creatinase/Aminopeptidase P, N-terminal | 4 | 6 | 4 | 4 | 4 | 3 | 4 | 5 | 4 | 5 | 10 | 3 | 5 | 5 | 5 | 71 | 10 |
| IPR011356 | Leucine aminopeptidase/peptidase B | 3 | 4 | 2 | 2 | 1 | 4 | 7 | 5 | 3 | 5 | 3 | 4 | 3 | 10 | 5 | 61 | 10 |
| IPR000819 | Peptidase M17, leucyl aminopeptidase, C-terminal | 3 | 4 | 2 | 3 | 1 | 4 | 7 | 5 | 3 | 5 | 7 | 4 | 3 | 10 | 5 | 66 | 10 |
| IPR000209 | Peptidase S8/S53 domain | 8 | 6 | 10 | 6 | 5 | 8 | 7 | 6 | 8 | 7 | 10 | 9 | 8 | 5 | 5 | 108 | 10 |
| IPR001300 | Peptidase C2, calpain, catalytic domain | 6 | 8 | 9 | 5 | 4 | 8 | 5 | 7 | 6 | 6 | 8 | 5 | 5 | 4 | 9 | 95 | 9 |
| IPR022764 | Peptidase S54, rhomboid domain | 6 | 6 | 6 | 6 | 6 | 9 | 7 | 6 | 4 | 6 | 4 | 5 | 4 | 7 | 4 | 86 | 9 |
| IPR022684 | Peptidase C2, calpain family | 6 | 7 | 9 | 5 | 4 | 7 | 4 | 6 | 5 | 5 | 9 | 4 | 5 | 4 | 8 | 88 | 9 |
| IPR008257 | Renal dipeptidase family | 8 | 3 | 1 | 6 | 5 | 4 | 3 | 3 | 5 | 5 | 3 | 3 | 0 | 4 | 5 | 58 | 8 |
| IPR015500 | Peptidase S8, subtilisin-related | 6 | 6 | 8 | 6 | 4 | 4 | 5 | 6 | 6 | 7 | 8 | 8 | 4 | 5 | 5 | 88 | 8 |
| IPR022683 | Peptidase C2, calpain, domain III | 4 | 6 | 8 | 3 | 3 | 5 | 2 | 5 | 4 | 4 | 7 | 2 | 4 | 3 | 6 | 66 | 8 |
| IPR015927 | Peptidase S24/S26A/S26B/S26C | 3 | 3 | 8 | 6 | 2 | 3 | 2 | 3 | 3 | 3 | 5 | 3 | 3 | 3 | 3 | 53 | 8 |
| IPR008283 | Peptidase M17, leucyl aminopeptidase, N-terminal | 1 | 3 | 1 | 2 | 0 | 3 | 2 | 3 | 2 | 2 | 2 | 2 | 1 | 8 | 3 | 35 | 8 |
| IPR022682 | Peptidase C2, calpain, large subunit, domain III | 4 | 6 | 8 | 3 | 3 | 5 | 3 | 5 | 4 | 4 | 8 | 2 | 4 | 3 | 7 | 69 | 8 |
| IPR000243 | Peptidase T1A, proteasome beta-subunit | 3 | 3 | 2 | 2 | 3 | 3 | 3 | 3 | 4 | 4 | 1 | 4 | 2 | 7 | 3 | 47 | 7 |
| IPR013273 | Peptidase M12B, ADAM-TS | 7 | 5 | 4 | 7 | 5 | 5 | 6 | 7 | 6 | 5 | 3 | 4 | 4 | 5 | 5 | 78 | 7 |
| IPR008969 | Carboxypeptidase-like, regulatory domain | 6 | 5 | 6 | 3 | 2 | 5 | 4 | 7 | 4 | 4 | 7 | 4 | 1 | 3 | 2 | 63 | 7 |
| IPR003111 | ATP-dependent protease La (LON), substrate-binding domain | 3 | 3 | 7 | 4 | 3 | 3 | 3 | 3 | 4 | 3 | 5 | 3 | 4 | 2 | 2 | 52 | 7 |
| IPR000246 | Peptidase T2, asparaginase 2 | 3 | 4 | 3 | 4 | 4 | 5 | 2 | 5 | 4 | 6 | 3 | 3 | 3 | 5 | 3 | 57 | 6 |
| IPR023302 | Peptidase S9A, N-terminal domain | 1 | 1 | 0 | 1 | 1 | 1 | 1 | 1 | 2 | 1 | 4 | 1 | 6 | 2 | 1 | 24 | 6 |
| IPR001915 | Peptidase M48 | 2 | 2 | 6 | 3 | 2 | 1 | 1 | 2 | 3 | 1 | 2 | 1 | 1 | 4 | 1 | 32 | 6 |
| IPR019759 | Peptidase S24/S26A/S26B | 3 | 3 | 6 | 6 | 2 | 3 | 2 | 3 | 3 | 3 | 5 | 3 | 3 | 3 | 3 | 51 | 6 |
| IPR007865 | Aminopeptidase P, N-terminal | 3 | 2 | 2 | 2 | 2 | 1 | 2 | 3 | 2 | 3 | 6 | 2 | 2 | 3 | 2 | 37 | 6 |
| IPR001096 | Peptidase C13, legumain | 2 | 1 | 6 | 1 | 1 | 1 | 1 | 1 | 1 | 1 | 1 | 1 | 2 | 1 | 1 | 22 | 6 |

**Table S24. InterPro domain counts for esterases.**

| **IPR** | **IPR_name** | **ZNEVA** | **PHUMA** | **APISU** | **AMELL** | **NVITR** | **AGLAB** | **DPOND** | **TCAST** | **APLAN** | **OTAUR** | **PXYLO** | **DPLEX** | **MDEST** | **DMELA** | **AGAMB** | **Total** | **Max** |
| --- | --- | --- | --- | --- | --- | --- | --- | --- | --- | --- | --- | --- | --- | --- | --- | --- | --- | --- |
| IPR002018 | Carboxylesterase, type B | 41 | 22 | 49 | 29 | 50 | 105 | 59 | 60 | 43 | 80 | 61 | 67 | 33 | 35 | 50 | 784 | 105 |
| IPR004843 | Calcineurin-like phosphoesterase domain, apaH type | 25 | 23 | 23 | 18 | 20 | 27 | 24 | 27 | 27 | 27 | 26 | 22 | 21 | 33 | 26 | 369 | 33 |
| IPR017946 | PLC-like phosphodiesterase, TIM beta/alpha-barrel domain | 12 | 10 | 11 | 12 | 9 | 10 | 16 | 8 | 9 | 7 | 17 | 12 | 12 | 12 | 8 | 165 | 17 |
| IPR002073 | 3'5'-cyclic nucleotide phosphodiesterase, catalytic domain | 7 | 10 | 7 | 8 | 7 | 10 | 8 | 11 | 9 | 13 | 14 | 9 | 9 | 6 | 8 | 136 | 14 |
| IPR023088 | 3'5'-cyclic nucleotide phosphodiesterase | 7 | 9 | 7 | 8 | 7 | 8 | 6 | 11 | 8 | 13 | 13 | 9 | 9 | 6 | 8 | 129 | 13 |
| IPR000070 | Pectinesterase, catalytic | 0 | 0 | 0 | 0 | 0 | 0 | 13 | 0 | 0 | 0 | 1 | 0 | 0 | 0 | 0 | 14 | 13 |
| IPR001031 | Thioesterase | 2 | 2 | 3 | 2 | 4 | 8 | 3 | 6 | 2 | 7 | 5 | 3 | 3 | 3 | 4 | 57 | 8 |
| IPR006683 | Thioesterase superfamily | 1 | 1 | 2 | 0 | 1 | 1 | 2 | 3 | 1 | 3 | 6 | 1 | 2 | 2 | 1 | 27 | 6 |

**Table S25. InterPro domain counts for cytochrome P450s.**

| **IPR** | **IPR_name** | **ZNEVA** | **PHUMA** | **APISU** | **AMELL** | **NVITR** | **AGLAB** | **DPOND** | **TCAST** | **APLAN** | **OTAUR** | **PXYLO** | **DPLEX** | **MDEST** | **DMELA** | **AGAMB** | **Total** | **Max** |
| --- | --- | --- | --- | --- | --- | --- | --- | --- | --- | --- | --- | --- | --- | --- | --- | --- | --- | --- |
| IPR001128 | Cytochrome P450 | 76 | 39 | 85 | 56 | 103 | 120 | 93 | 127 | 45 | 135 | 90 | 88 | 72 | 88 | 113 | 1330 | 135 |
| IPR002401 | Cytochrome P450, E-class, group I | 51 | 31 | 55 | 44 | 78 | 65 | 66 | 119 | 29 | 101 | 64 | 63 | 44 | 77 | 96 | 983 | 119 |
| IPR002402 | Cytochrome P450, E-class, group II | 4 | 1 | 4 | 3 | 3 | 18 | 8 | 2 | 3 | 5 | 2 | 3 | 7 | 1 | 4 | 68 | 18 |
| IPR002403 | Cytochrome P450, E-class, group IV | 9 | 6 | 15 | 6 | 13 | 16 | 13 | 4 | 1 | 14 | 10 | 6 | 11 | 9 | 8 | 141 | 16 |

**Table S26. InterPro domain counts for UDP glycosyltransferases.**

| **IPR** | **IPR_name** | **ZNEVA** | **PHUMA** | **APISU** | **AMELL** | **NVITR** | **AGLAB** | **DPOND** | **TCAST** | **APLAN** | **OTAUR** | **PXYLO** | **DPLEX** | **MDEST** | **DMELA** | **AGAMB** | **Total** | **Max** |
| --- | --- | --- | --- | --- | --- | --- | --- | --- | --- | --- | --- | --- | --- | --- | --- | --- | --- | --- |
| IPR002213 | UDP-glucuronosyl/UDP-glucosyltransferase | 23 | 4 | 72 | 12 | 24 | 49 | 22 | 27 | 29 | 33 | 26 | 33 | 17 | 35 | 26 | 432 | 72 |

**V. Sensory Biology: Olfaction, Gustation and Vision**

**V.1 Chemoperception**

**Contributors**

**Hugh M. Robertson** (University of Illinois at Urbana-Champaign, Department of Entomology)

**Robert F. Mitchell** (University of Arizona, Center for Insect Science; University of Wisconsin, Department of Biology, Oshkosh, WI, USA)

**Tiffany Bledsoe** (University of Arizona, Center for Insect Science,Tuscon, AZ, USA)

**Summary**

Chemoperception and vision are two major sensory modalities of insects. Chemoperception involves four major gene families. The odorant binding proteins (OBPs) are involved in transporting commonly hydrophobic chemicals from the exterior to the sensory neurons, where three families of receptors mediate specificity and sensitivity of responses: the odorant receptors (ORs), gustatory receptors (GRs), and ionotropic receptors (IRs) [142, 143]. We compared the members of these four families with those from *Tribolium castaneum* and, when relevant, *Drosophila* *melanogaster* (Table S27). ORs were also compared to receptors identified from the transcriptome of the cerambycid *Megacyllene caryae* [144]. Like *T. castaneum* [10, 17, 145], *A. glabripennis* has large OR and GR repertoires compared with *D. melanogaster*, and indeed most other insects except ants, but their OBP and IR repertoires are more comparable with that of *D. melanogaster* and similar to many other insects.

**Discussion**

Odorant binding proteins

Odorant binding proteins (OBPs) are small, soluble proteins that bind and transport odorants as they enter the insect sensillum [142, 143]. Several families and expansions of OBPs are recognized in insects, which are broadly categorized by the number of cysteine residues: “classic” OBPs that present a conserved 6-cysteine motif; “minus-C” OBPs that have lost 1-2 of these residues; and “plus-C” OBPs that contain 12 conserved cysteines [143]. Additional radiations of OBPs identified to date are lineage-specific, such as the minus-C OBPs of *Apis mellifera* [145] and the “atypical” OBPs of *Anopheles gambiae* [146].

We identified 52 OBP genes (Fig. S23). The majority of OBPs comprise a large expansion of the minus-C subfamily, and the remaining genes are placed singly or in small radiations that exhibit the classic 6-cysteine motif. Two orthologs were recovered for DmelOBP59a and DmelOBP73a, but only a single OBP (AglaOBP51) was identified as a member of the plus-C group, consistent with observations for *T. castaneum* [145] and transcriptomes of the bark beetles *Dendroctonus ponderosae* and *Ips typographus* [147]. This pattern suggests that the tendency toward minus-C OBPs originated at least with the infraorder Cucujiformia (~240 mya) [148], but genomes from additional coleopteran groups will be necessary to determine if this is characteristic of the entire order.

Odorant receptors

Odorant receptor proteins are membrane-bound heteromeric complexes that are the primary means by which insects detect volatile chemicals, dating at least to the evolution of insect flight [149]. Insect odorant receptors contain seven transmembrane domains, but are not homologous with vertebrate odorant receptors, and the exact mechanism of their function remains elusive [150-152]. Each receptor complex consists of two structures: an olfactory receptor co-receptor (Orco) and an odorant receptor (Or) that contains the specific odorant-binding site (see [142] for review). To date, neopteran insect genomes have contained a single copy of Orco that is highly conserved, and a variable number of Ors that evolve rapidly and are extraordinarily divergent across insect orders [10, 153-155].

The Ors of beetles are known almost exclusively from the genome of *T*. *castaneum* [10] and transcriptomes of *M. caryae* [144] and the scolytine bark beetles [147, 155]*.* These receptors appear to form seven major lineages, with members of groups 1-3 present in all beetles to date, 4-6 exclusive to *T*. *castaneum*, and group 7 identified only from *M*. *caryae* and the scolytines [10, 144, 147]. Only three odorant receptors have been functionally characterized within the Coleoptera, and all from *M*. *caryae*; these receptors are sensitive to components of the long-range pheromone produced by males of that species [144].

*A. glabripennis* has 131 OR genes in addition to the highly conserved OR co-receptor, Orco. These ORs are represented among all seven recognized subfamilies of coleopteran ORs except group 6 [10, 144, 147], and follow the pattern of frequent paralogous radiations that characterizes insect chemoreceptors (Fig. S24). Two new lineages of ORs were identified from *A. glabripennis* and placed as outgroups to OR groups 4, 5, and 6 in *T. castaneum* (Or106-115/126-132 and Or101-103). This organization suggests that groups 4-6 are expansions specific to *T. castaneum* or tenebrionid beetles and may be subgroups of a larger coleopteran lineage. The functions of beetle ORs remain mostly unknown, and to date receptors have only been characterized from *M. caryae* (McarOr3, 5, 20) [144]. Notably, AglaOr29 is sister to McarOr3, which is sensitive to 2-methylbutan-1-ol, a pheromone component of *M. caryae* [144]. 2-methylbutan-1-ol may therefore be important to the life history of *A. glabripennis*; however, function cannot easily be inferred even across orthologs, because a single amino acid may change receptor specificity [156].

Gustatory receptors

The genome encodes an extensive suite of 234 GRs, including the conserved three candidate carbon dioxide receptors (Gr1-3), ten candidate sugar receptors (Gr4-13), and three candidate fructose receptors related to DmGr43a (Gr14-16). The remaining 127 GR genes encode 218 receptors through alternative splicing of several loci, and are presumed to belong to the general category of candidate bitter taste receptors, although some likely are also involved in contact pheromone perception [157].

Ionotropic receptors

*A. glabripennis* has 72 IRs including simple orthologs of the well-conserved co-receptors IR8a and 25a, and of IR21a, 40a, 41a, 68a, 76b, 93a, and 100a (in contrast, *T. castaneum* has 3 relatives of IR41a and 11 of IR100a). The IR75 lineage consists of 8 genes compared with 6 in *T. castaneum* and 7 in *D. melanogaster*. These are all candidate olfactory receptors, while the candidate gustatory receptors, represented by the DmIr20a clade of 40 genes [158], consist of 55 genes, compared to 53 in *T. castaneum*, although these two beetles exhibit differential species-specific expansion of gene lineages within this large grouping.

**Table S27. Genes/proteins in the family of odorant binding proteins (OBPs) and three families of chemoreceptors. Pseudogenes are included in the total count, and indicated separately in parentheses.**

| **Species** | **OBPs** | **Odorant** | **Gustatory** | **Ionotropic** |
| --- | --- | --- | --- | --- |
| *A. glabripennis* | 52 (1) | 131/131 (11) | 143/234 (32) | 72/72 (9) |
| *T. castaneum* | 49 (1) | 341/341 (79) | 215/245 (26) | 80/80 (9) |
| *D. melanogaster* | 52 (0) | 60/62 (1) | 60/68 (0) | 64/64 (4) |

**Figure S23**. **Unrooted phylogram illustrating relationships among peptide sequences of odorant binding proteins (OBPs) from *Tribolium castaneum* (Tenebrionidae) and *Anoplophora glabripennis* (Cerambycidae), and representative OBPs from *Drosophila melanogaster* and *Apis mellifera*.** Key clades of OBPs are designated by thick lines surrounding the phylogeny, and thin black lines indicate apparent orthologs shared between beetles and *D*. *melanogaster*. Support values are presented for major branches.


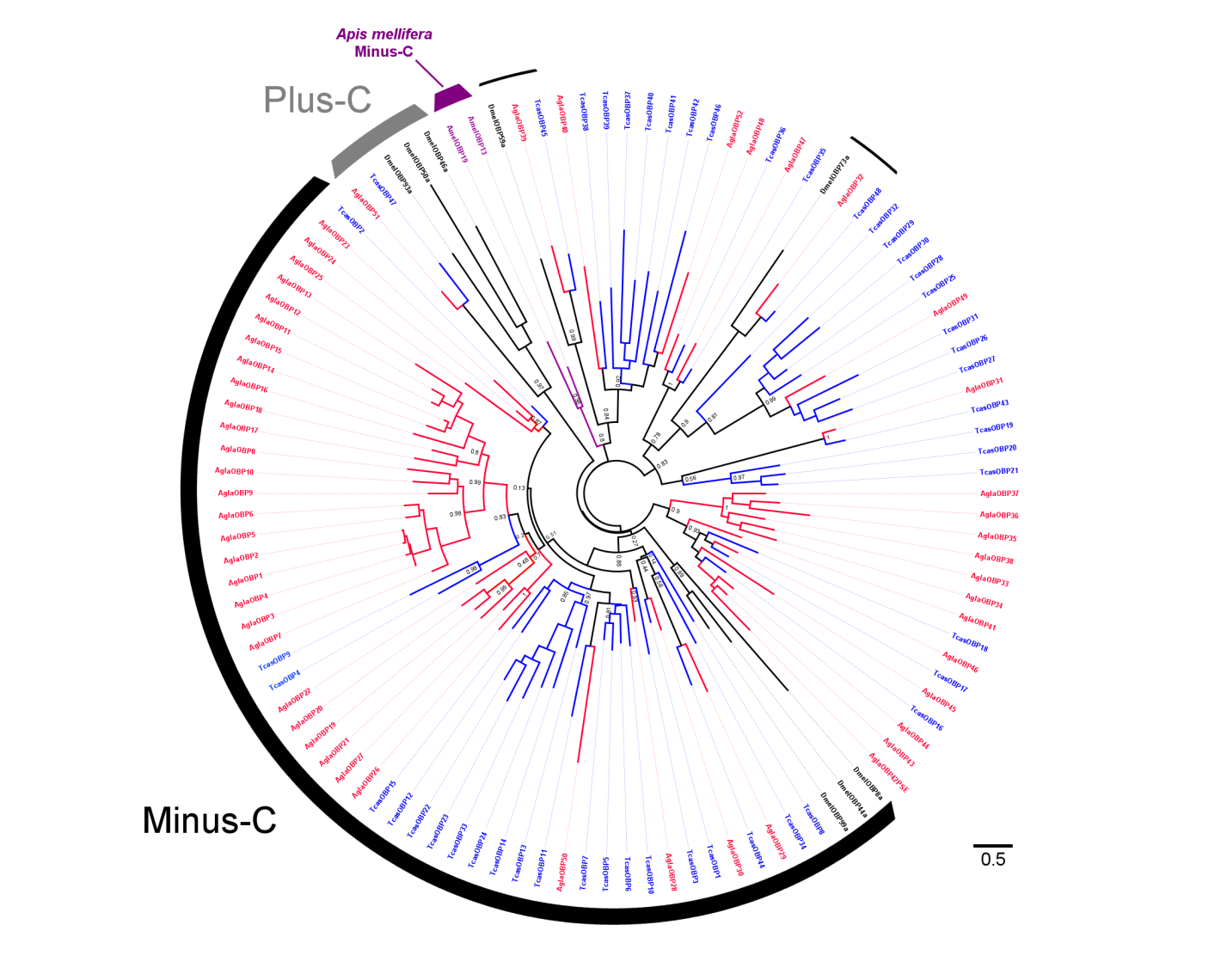


**Figure S24.** **Unrooted phylogram illustrating relationships among peptide sequences of odorant receptors (Ors) from *Tribolium castaneum* (Tenebrionidae: Tenebrioninae), *Megacyllene caryae* (Cerambycidae: Cerambycinae), and *Anoplophora glabripennis* (Cerambycidae: Lamiinae).** The seven recognized lineages of beetle receptors are indicated by thick colored lines surrounding the phylogeny. Ligands for deorphaned receptors are indicated where known. Support values are presented for major branches.


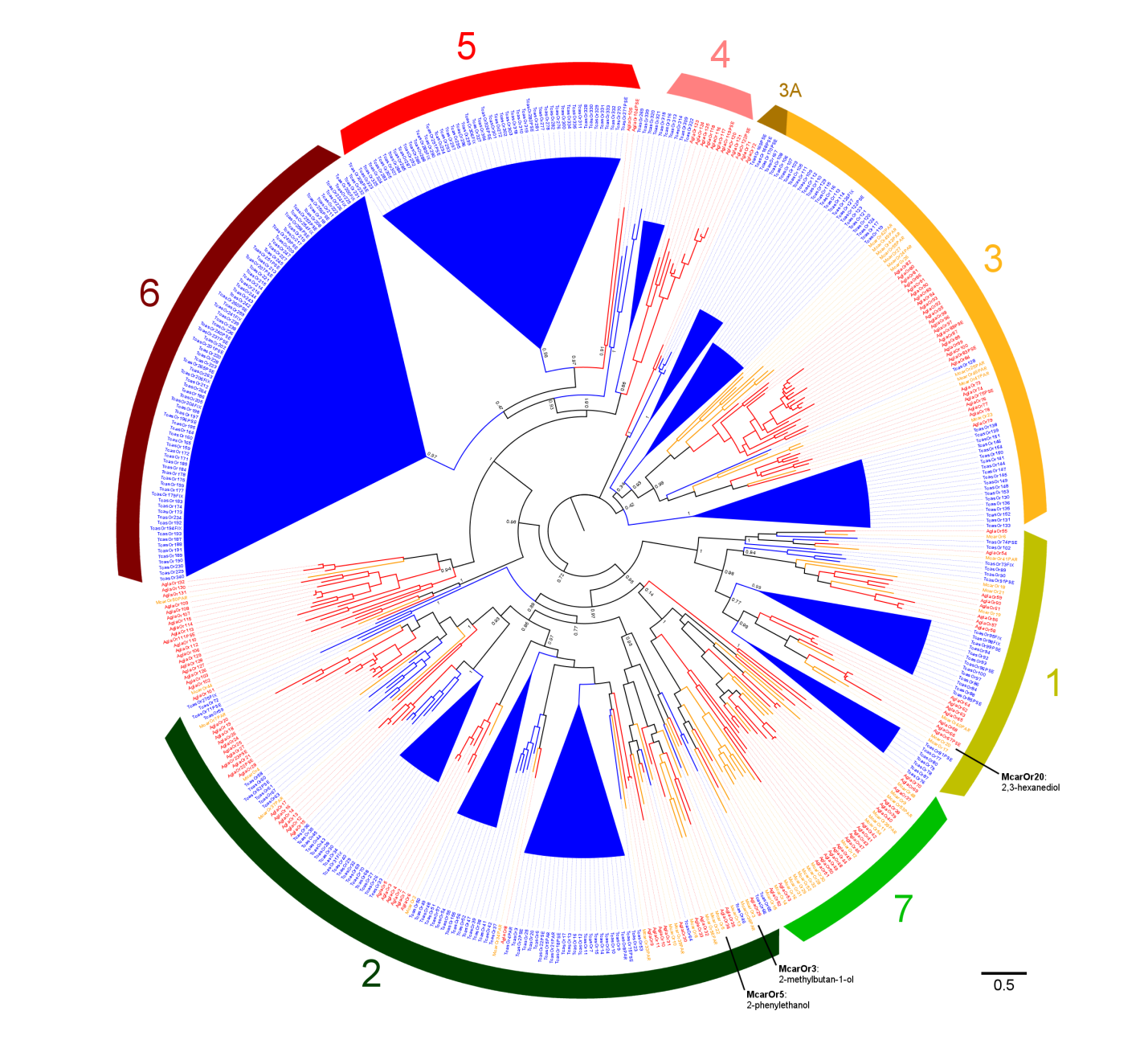


**V.2 Vision**

**Contributors**

**Marcus Friedrich** (Wayne State University, Department of Biological Sciences)

**Jeffery W. Jones** (Oakland University, Department of Biological Sciences)

**Summary**

Reciprocal BLAST searches of the *Anoplophora glabripennis* genome scaffolds recovered three members of the G-protein coupled transmembrane receptor opsin gene family. Two of them represent members of visual subfamilies, which are expressed in the retina of species, where expression and function has been investigated, such as the fruitfly *Drosophila melanogaster* and the red flour beetle *Tribolium castaneum*. Specifically, we found one member of the long wavelength-sensitive opsin subfamily and one member of the UV-sensitive opsin subfamily. This situation is identical with *T. castaneum*. In addition, we found a member of the Rh7 opsin subfamily, which is broadly conserved in insects including *D. melanogaster* although missing from *T. castaneum*. The function of Th7 opsins has not yet been characterized. *A. glabripennis* seems to further differ from *T. castaneum* by missing orthologs of the nonretinal subfamily of c-opsins, which is conserved in *T. castaneum* but likewise not characterized at the functional level.

**VI. Growth and Development**

**VI.1 Bone morphogenic protein signaling pathway**

**Contributor**

**Jeremy A. Lynch, Honghu Quan, Lorna Cohen, Deana Arsala, Dan Pers** (Department of Biological Sciences, University of Illinois at Chicago, Chicago, IL, USA)

**Summary**

The bone morphogenic protein signaling pathway (BMP) is used in a wide range of developmental processes throughout animals [159]. In general the assortment of BMP components in *Anoplophora glabripennis* is very similar to that of *Tribolium* *castaneum* [160]. One exception is a tandem duplication of the inhibitory SMAD *daughters against dpp*, whereas a single homolog is found in *T. castaneum* [160], some partially sequenced loci, and at least one missing gene (the inhibitor gremlin). Like *T. castaneum* and *Drosophila melanogaster* [160], and unlike Hymenoptera [161], *A. glabripennis* lacks an *anti-dorsalizing morphogenic proteing* (ADMP) ortholog, confirming that this gene was lost after the divergence of the lineage leading to beetles and flies from the Hymenoptera. Also interesting is the lack of the inhibitor gremlin, which was a surprising discovery in *T. castaneum* [162], but seems to be absent from *A. glabripennis*. To summarize the list of BMP signigalling pathway genes identified is: Ligands:

decapentaplegic (*Drosophila*): 1; glass bottom boat (*Drosophila*): 2 (same as in most insect species); myostatin: 1 provisionally; activin: partial, missing sequence; maverick: 1; BMP10: 1

ADMP: 0 (also missing from *T. castaneum* and *D. melanogaster*). **Receptors:** *Thickveins*: 1, potentially a duplication but not complete; saxophone: 1; *punt*: 1; *wishful thinking*:1; *baboon*: Potentially 2, but both are partial, with gaps in the assembly. **Smads (transcription factors downstream):** *Mothers against dpp* (*Mad*): 3 (similar to *T. castaneum*); *Smad on X*: 0, but hard to differentiate from *Mads*; *Medea*: 1; *daughters against dpp*: 2 (apparent tandem duplication).

**Modulators:** *short gastrulation*: 1; *tolloid/tolkin*: 1; *twisted gastrulation/crossveinless*: 1; *BAMBI*: 1; *Dan/neuroblastoma*: 1; *gremlin*:0; *dawdle*: 1

**VI.2 Germline genes**

**Contributor**

**Jeremy A. Lynch, Honghu Quan, Lorna Cohen, Deana Arsala, Dan Pers** (Department of Biological Sciences, University of Illinois at Chicago, Chicago, IL, USA)

**Summary**

All of the classically described germline specifying genes known from *Drosophila melanogaster* [163] are present in the genome of *Anoplophora glabripennis*. This includes an oskar ortholog, which is not found in *Tribolium castaneum* [162, 164], but is present in other chrysomelid genomes (*Leptinotarsa*, *Callosobruchus maculatus* (J. Lynch personal observation). This indicates that *A. glabripennis* employs a maternal provisioning mode of germline determination more similar to that of *D. melanogaster* than to that of *T. castaneum*. Other genes required for assembly and regulation of the germline were present in single copies (e.g., bruno, vasa, tudor, maelstrom, boule, and hrp48). The translational regulator nanos was also found, and like *T. castaneum* nanos [165] is highly divergent from other insect *nanos* sequences.

**VI.3 Homeodomain transcription factors (Hox, Iro-C)**

**Contributors**

**Kristen A. Panfilio and Iris M. Vargas Jentzsch** **(**University of Cologne, Institute for Developmental Biology, Cologne, Germany)

**Summary and Methods**

Here, we describe two distinct genomic clusters of homeodomain transcription factors that arose by ancient tandem duplications within the animals. Given the high degree of expected conservation, the identification of linked orthologous genes simultaneously serves as a quality assessment for genome assembly and as case study examples for evaluating synteny across insect species. In the two instances evaluated here, we could find linked, complete gene models for all expected orthologs. Of note are a particular instance of potential lineage specific duplications [166] the extent to which gene loci and gene cluster sizes are proportionately larger in the six-fold larger genome of *Anoplophora glabripennis* (estimated 981 Mb genome size, *cf.*, 710 Mb assembly; see genome size section above) compared to that of *Tribolium castaneum* (160 Mb) [162], and the use of *A. glabripennis* as a phylogenetically useful taxon for determining the history of tandem duplications across the insects and vertebrates.

We annotated the Hox genes by performing tblastn searches on the *A. glabripennis* scaffolds with the corresponding *T. castaneum* Hox gene protein sequences available in NCBI (the current official gene set, OGS, models). To confirm orthology, we then blasted our *A. glabripennis* models back into NCBI. Homology, intron/exon boundary assessments, and protein sequence completeness were identified by manual inspection and correction of protein alignments generated with ClustalW2 (http://www.ebi.ac.uk/Tools/msa/clustalw2/).

Splice site information for *T. castaneum* was obtained for the OGS gene models as accessed from Assemblies 3.0 and 4.0 in the genome browser of the Stanke group, University of Greifswald (http://bioinf.637 uni-greifswald.de/gb2/gbrowse/tcas4/). Possible gene loci duplications were identified by performing tblastn searches on the scaffolds using the protein sequences of completed annotation models as queries, and then re-blasting the resulting hit sequences into NCBI for Arthropoda hits.

**Results and Discussion**

Hox genes are a classic example of conservation across the Bilateria, both for the genomic organization and the developmental function of these transcription factors [166]. We were able to find and annotate complete gene models for all ten Hox cluster genes (Table S28). The genes for this cluster were found in the expected gene order, but split between two different scaffolds (Fig. S25A, B), presumably due to incomplete assembly of the draft genome. Assuming direct concatenation of these two scaffolds, the Hox cluster would span a region of 3.5 Mb. In contrast, the single Hox cluster in *T. castaneum* is much more compact at only 0.7 Mb [162]. Although the gene loci and the intergenic regions are larger in *A. glabripennis* (up to 8-fold and 5-fold, respectively), protein sizes are only 10% larger and intron splice sites are largely conserved between *T. castaneum* and *A. glabripennis*. Interestingly, we observed a change in transcriptional orientation within the *A. glabripennis* Hox cluster, as the class 3 Hox gene locus, encoding *zerknüllt* (*zen*), is inverted with respect to all other genes (Fig. S25A). This kind of microinversion is not uncommon in dipteran species for the Hox genes that lost their homeotic function in winged insects, namely *zen* and *ftz* [167].

Also as would be expected for highly conserved genes, we only found evidence for single orthologs, with *zen* again representing an exception. In the insects *zen* has diverged from a canonical Hox gene role in embryonic tissue specification along the anterior-posterior body axis, acquiring rather diverse roles in the extraembryonic tissue of the egg [168]. Associated with this evolutionary lability in function is a tendency for lineage specific duplications of this particular gene locus, which are known for a centipede, *Drosophila*, and indeed *T. castaneum* [169]. We did not find evidence for the tandem *zen* duplication of *T. castaneum* [170], but we did find potential gene models for up to six putative class 3 Hox genes, with three of these occurring in tandem within the Hox cluster. As these are purely bioinformatic models, it remains to be seen whether these loci represent genuine protein coding genes. If so, this would be a distinct instance of class 3 Hox gene duplications within the Coleoptera, further supporting the classification of this locus as an evolutionary ‘hotspot’.

Synteny is also conserved within the small Iroquois Complex (Iro-C), a second family of homeodomain transcription factors that arose from ancient tandem duplications. Whereas *Drosophila* has three family members [171-173], we find that both *A. glabripennis* and *T. castaneum* possess just two (Table S28): *mirror* (*mirr*), conserved across the Insecta, and *iroquois* (*iro*), which is the single gene ortholog corresponding to the tandem paralogs *araucan* (*ara*) and *caupolican* (*caup*) in *Drosophila melanogaster*. As in *T. castaneum*, the two Iro-C genes in *A. glabripennis* occur in tandem with the same transcriptional orientation along the scaffold, with *iro* upstream of *mirr* (Fig. S25B), although the *mirr* gene locus and intergenic region are each 2-3 times larger in *A. glabripennis* than in *T. castaneum*. Both Iro-C genes are also conserved compared to *T. castaneum* at the level of gene structure (4 conserved splice sites out of 4-5) and protein sequence (≥84% identity for ≥94% sequence coverage).

It is intriguing that in vertebrates the orthologous Irx genes occur in clusters of three genes each, as in *D. melanogaster*, and there is evidence that the genes within these clusters act in concert in regulating multiple developmental processes in both lineages [174-176]. However, phylogenetic comparisons between the *D. melanogaster* and vertebrate proteins already suggested that all three fly genes reflect tandem duplications after the divergence from the last common ancestor with vertebrates, while only a single Iroquois Complex gene is known for *Caenorhabditus elegans* [176]. Thus, in light of our analyses in beetles, the duplication to produce *iro* and *mirr* may have occurred as recently as the divergence of arthropods from other ecdysozoan lineages, while the second duplication giving rise to *ara* and *caup* is restricted within the Holometabola and may be specific to the Diptera.

**Figure S25.** **(A)** **Schematic representation of the Hox cluster. (B) Schematic representation of the Iroquois Complex.** Both are shown to scale for gene loci and transcriptional orientation within the context of total scaffold lengths. For clarity, only the canonical *zen* gene is shown.


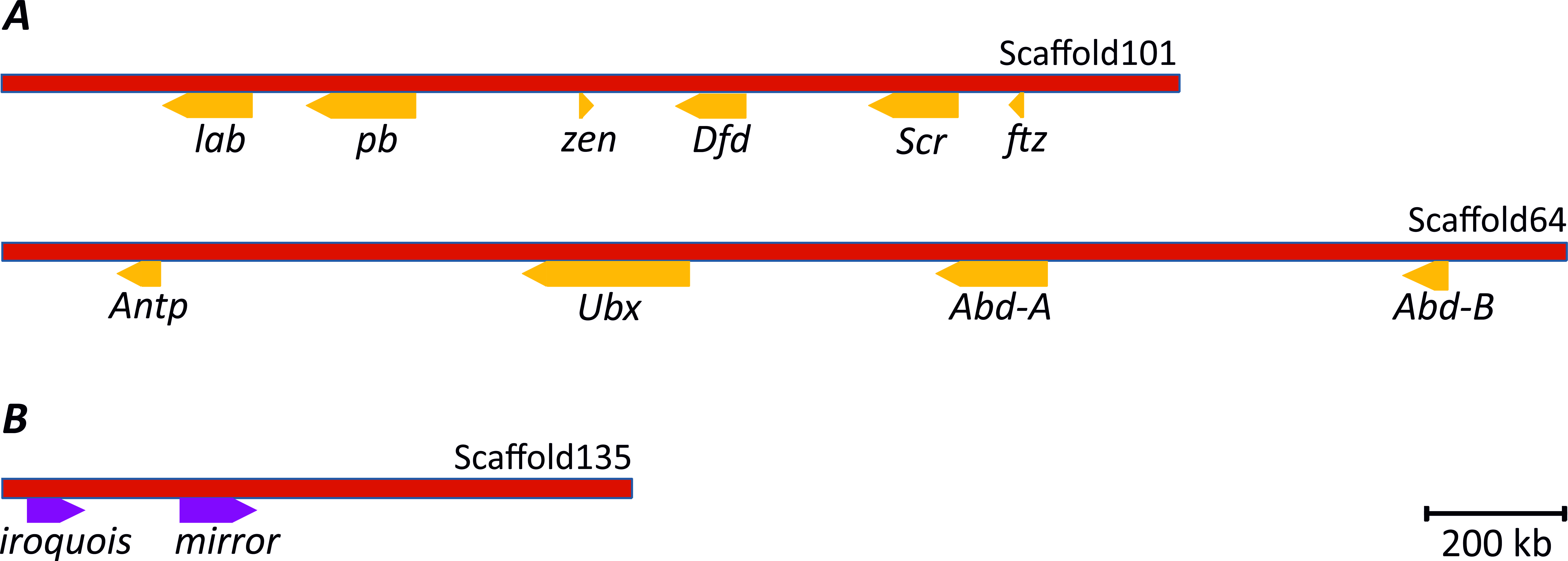


**Table S28.** **Positional information for the annotated genes from the Hox cluster and Iroquois-Complex.**

| **Gene** | **Scaffold: start..end** | **Locus length** | **Number of CDS exons** |
| --- | --- | --- | --- |
| *labial* | Scaffold101:1292696..1400624 | 107,929 | 2 |
| *proboscipedia* | Scaffold101:1056000..1203951 | 147,952 | 4 |
| *zerknüllt* | Scaffold101: 844,307..845,821 | 1,515 | 3 |
| *Deformed* | Scaffold101:613726..677278 | 63,553 | 2 |
| *Sex combs reduced* | Scaffold101:297481..400882 | 103,402 | 2 |
| *fushi tarazu* | Scaffold101:228190..230388 | 2,199 | 2 |
| *Antennapedia* | Scaffold64:2021094..2048266 | 27,173 | 2 |
| *Ultrabithorax* | Scaffold64:1222387..1434800 | 212,414 | 2 |
| *Abdominal-A* | Scaffold64:721315..851181 | 129,867 | 3 |
| *Abdominal-B* | Scaffold64:128442..142038 | 13,597 | 2 |
| *iroquois* | Scaffold135:803041..875793 | 72,753 | 6 |
| *mirror* | Scaffold135:526196..634537 | 108,340 | 5 |

**VI.4 Embryo and imaginal disc patterning genes**

**Contributor**

**Jeremy A. Lynch, Honghu Quan, Lorna Cohen, Deana Arsala, Dan Pers** (Department of Biological Sciences, University of Illinois at Chicago, Chicago, IL, USA)

**Summary**

**Representative embryo and imaginal disc patterning genes.**

We examined genes involved in large scale patterning of imaginal discs or embryos (gap genes) of *D. melanogaster.* All genes searched for were found, and were present in single copies. The exception was the gap-gene *knirps* which has a related paralog named eagle in *T. castaneum* and *Drosophila*. In *Anoplophora glabripennis* only a single gene of the *knirps* orthology group was found. The list of genes found is: *engrailed* (disc and segmentation); *omb* (disc gene); *spalt* (disc gene); *vestigial* (disc gene); *scalloped* (disc gene); *distalless* (disc gene); *ventral veins; lacking* (disc gene); *brinker* (disc gene); *wingless* (disc and segmentation); *Notch* (disc); *hunchback* (gap gene); *Krueppel* (gap gene); *giant* (gap gene); *knirps/eagle* (gap gene); *tailless* (gap gene); *orthodenticle 1* (gap genes); *orthodenticle 2* (gap gene); *empty spiracles* (gap gene); *buttonhead* (gap gene); *milles pattes* (poly cistronic mRNA, gap gene in *Tribolium*).

**VI.5 Dorso-ventral fate specifying factors**

**Contributor**

**Jeremy A. Lynch, Honghu Quan, Lorna Cohen, Deana Arsala, Dan Pers** (Department of Biological Sciences, University of Illinois at Chicago, Chicago, IL, USA)

**Summary**

Dorso-ventral (DV) patterning in insects divides and patterns the early embryo into distinct domains. Broadly these are the mesoderm, mesectoderm, neurogenic ectoderm, and extraembryonic membranes [177]. The classical mesoderm markers twist and snail were both found in single copy in *Anoplophora glabripennis,* similar to *Tribolium castaneum* [162], but different from *Drosophila* *melanogaster* where the paralogs *escargot* and *worniu* are also found [178]. The marker of the mesectoderm (giving rise to much of the central nervous system), *single-minded* [179], is present in *A. glabripennis*. Similarly, the neurogenic genes *ventral neuroblasts defective, intermediate neuroblasts defective*, and *muscle segment homeobox* [180] were all found, as is the marker of the dorsal ectoderm and extraembryonic membranes *pannier* [181]*.* While Toll and BMP signaling pathways provide the main maternal inputs into DV patterning in insects [161, 182, 183], other signaling pathways act downstream to elaborate and refine maternal information [184]. The FGF pathway acts to control mesoderm migration, and the crucial components FGF receptor, stumps, and fgf8 ligand are all present in *A. glabripennis*. Similarly, EGF signaling is important for ectodermal patterning, and the crucial components *spitz* (the lone EGF ligand, as in *T. castaneum* [185], *rhomboid*, and the *EGF receptor* were found in *A. glabripennis*. In summary the final list of DV patterning genes identified was: *twist*; *snail*; *single minded*; *brinker; ventral neuroblasts defective*; *intermediate neuroblasts defective*; *muscle segment homeobox*; *zerknuellt^[[1]](#footnote-1)^*; *pannier*-; *netrin*; *breathless* (fgfr); *stumps*; *fgf8* (*pyramus* and *thisbe*); *spitz*; *rhomboid*; and the EGF receptor.

**VI.6 Nuclear receptors**

**Contributor**

**Subba R. Palli** (University of Kentucky, Department of Entomology)

**Summary**

Nuclear receptors (NRs) are a group of proteins classified by the presence of two functional domains: a highly conserved DNA-binding domain (DBD) consisting of two C4 type zinc fingers that targets the receptor for specific response elements in the genome and a less conserved ligand–binding domain (LBD) containing a ligand binding pocket that can accommodate small molecules, dimerization domain and activation domain [186-188] (Fig. S26).

The members of the NR family in humans include receptors for steroid hormones, thyroid hormones, vitamin D, and retinoic acid. About half of human NRs function through known natural ligands and the other half, so-called orphan receptors, have no known natural ligands. NRs play key roles in embryonic and post-embryonic development and reproduction, functioning as key control points in diverse signaling and metabolic pathways such as metabolism, sex determination, circadian rhythm, aging, and xenobiotic detoxification. Because NRs are naturally switched on and off by small molecule ligands with properties very similar to therapeutic chemical compounds, NRs are attractive targets for the prevention and treatment of diverse diseases such as cancer, coronary heart disease, and diabetes [189]. In fact, several known anti-cancer drugs, such as tamoxifen or flutamide, target NRs, and many more are expected to reach commercial markets in the near future [190, 191]. Several insecticides based on NRs, such as the ecdysone receptor, are currently used to control insect pests [192]. Other NRs also could be exploited to identify small molecules that can be used for management of pests.

The number of NRs identified in sequenced genomes varies considerably. Two hundred and eighty four nuclear receptors have been identified in the nematode *Caenorhabditis elegans* compared to 18 identified in the genome of the fruitfly *Drosophila melanogaster* [193]. In addition to 18 canonical NRs, the *D. melanogaster* genome also contains three additional NRs (the Knirps family) that contain well-conserved DBD but lack the LBD [194]. Nineteen canonical NRs have been identified in the genome of *Tribolium castaneum* [195]. The same 19 NRs are also present in the genome of *A. glabripennis* (Fig. S27; Tables S29, S30). In addition, two Knirps family NRs have been identified in both *A. glabripennis* and *T. castaneum* (Table S29).

**Figure S26: A diagram showing different domains present in the members of nuclear receptor superfamily.**


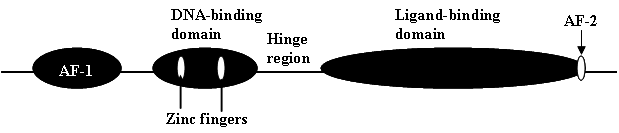


**Figure S27**. **Phylogenetic tree of 19 *A. glabripennis* nuclear receptors paired with their *Tribolium* *castaneum* homologues**.


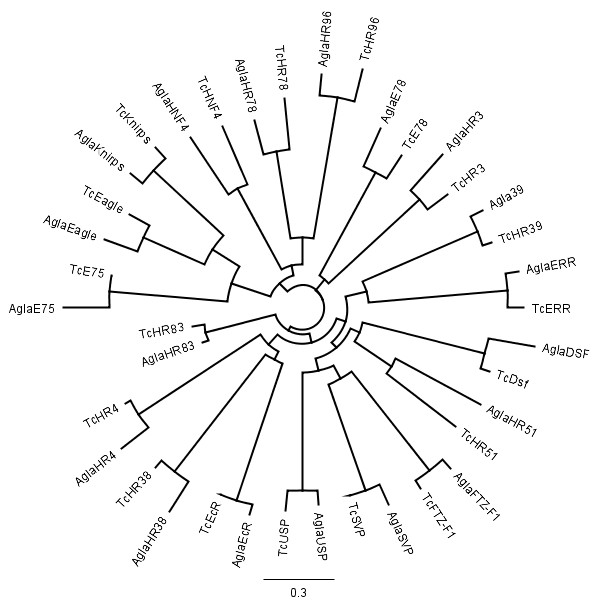


**Table S29. Nuclear receptors identified in *A. glabripennis***

| Name | Scaffold | Location | Length (kb) |
| --- | --- | --- | --- |
| HR78 | 16 | 753553..763202 | 9.65 |
| Sevenup | 206 | 572808..601607 | 28.8 |
| HR3 | 6 | 2474487..2521986 | 47.5 |
| USP | 263 | 713602..781985 | 68.39 |
| HR4 | 539 | 430816..500415 | 69.6 |
| FTZ-F1 | 877 | 76977..131776 | 54.8 |
| HR38 | 166 | 264400..265749 | 1.35 |
| ERR | 1146 | 134896..153695 | 18.8 |
| E78 | 404 | 484621..534620 | 50 |
| PNR | 33 | 1460493..1476442 | 15.95 |
| Knirps | 366 | 236110..288109 | 52 |
| Eagle | 366 | 751217..828916 | 77.7 |
| DSF | 76 | 869658..878637 | 8.98 |
| E75 | 83 | 1379169..1384768 | 5.6 |
| EcR | 3 | 2668745..2683444 | 14.7 |
| HNF4 | 1824 | 25864..28934 | 3.07 |
| HR51 | 282 | 521597..588396 | 66.8 |
| HR83 | 42 | 337100..349199 | 12.1 |
| HR96 | 9 | 1470954..1485103 | 14.15 |
| Tailess | 1356 | 67901..74950 | 7.05 |
| HR39 | 626 | 295900..319749 | 23.85 |

**Table S30. *A. glabripennis* nuclear receptors.**

Due to its size, this table has been moved to the supplementary excel file entitled “AdditionalFile 2_04Aug2016”.

**VI.7 Toll pathway**

**Contributor**

**Jeremy A. Lynch, Honghu Quan, Lorna Cohen, Deana Arsala, Dan Pers** (Department of Biological Sciences, University of Illinois at Chicago, Chicago, IL, USA)

**Summary**

The Toll signaling pathway plays a crucial role in both embryo dorsoventral (DV) patterning and immunity in insects [161, 182, 196]. All of the core components of Toll signaling were found in *Anoplophora glabripennis*, with the exception of the eggshell protease *nudel*. *Anoplophora glabripennis* has only one ortholog of the *Drosophila* Toll receptor, while *Nasonia vitripennis* and *Tribolium castaneum* each have four [161, 182]. There are two Dorsal (NFkB transcription factor) genes (same as *T. castaneum*), and two cactus (IkB inhibitory protein) genes, whereas *T. castaneum* only has a single cactus ortholog [182]. Interestingly, one *cactus* paralog is directly neighboring one of the Dorsal paralogs, which may have functional relevance, since transcriptional self-regulation has been shown to be important to the *T. castaneum* system [182, 196]. Single genes encoding orthologs of the adaptor proteins tube and *myd88* were found, and two paralogs of the kinase *pelle* were identified in *A. glabripennis*, compared to one of each in *T. castaneum* [182]. Five apparent paralogs of the Toll ligand spaetzle (*spz*) were found in *A. glabripennis*, compared to one in *Drosophila* and three in *T. castaneum* (JAL, personal observation). Four of these paralogs were clustered tandemly on scaffold 156.

A protease cascade is crucial for processing *spz* to an active form in *Drosophila*, and a similar cascade is also likely required in *A. glabripennis* and other insects. There are many genes showing high similarity to these trypsin like serine proteases. Based on a reciprocal BLAST, two paralogs of *easter* (*ea*), four paralogs of *snake* (*snk*), and two paralogs of *gastrulation defective* (*gd*) are present in *A. glabripennis*. A common theme among these is that many of the paralogs are embedded in genomic regions encoding numerous serine proteases of varying relatedness to either *ea*, *snk,* or *gd* *nudel* is a highly conserved component of the protease cascade [197], with a conserved function in DV patterning in *T. castaneum* (JAL, unpublished data)*.* This makes its absence in *A. glabripennis* extremely surprising, and requires further confirmation. Upstream of the proteases, the sulfotransferase Pipe modifies the eggshell to provide polarity to *spz* activation [198]. Unlike any other insects so far examined, there are two distinct *pipe* genes in *A. glabripennis*, the significance of which is unclear. To summarize, the final list of Toll pathway components identified is: a single *toll* receptor (tc nv have 4); 2 *Dorsal* genes (same as *Tribolium*); 1 *relish* gene; 2 *cactus* genes (1 in *Tribolium*) interestingly one is tandemly located next to a *Dorsal* paralog B on scaffold 32, 5 *spz* genes (4 clustered at Scaffold156:992601..1057000); compared to 1 locus in *Drosophila*, and 2 loci in *Tribolium* (potential tandem arrangement) best hit on scaffold 39; 1 *tube* gene; 2 *pelle* genes; 1 *myd88* gene; 2 *easter* genes (based on reciprocal blast hits) both embedded in cluster of serine proteases; 4 *snake* genes (based on reciprocal blast hits) 3 embedded in cluster of serine proteases; 2 *gastrulation defective* genes each in clusters of proteases; and 2 *pipe* genes.

**VI.8 RNAi pathway**

**Contributor**

**Scott M. Geib** (USDA-ARS, Pacific Basin Agricultural Research Center, Hilo, HA, USA)

**Summary**

To find *Anoplophora glabripennis* genes potentially involved in the insect RNAi pathway, 17 genes from *Tribolium castaneum* previously annotated as components of the RNAi pathway were used as reference [199, 200]. This species has the most complete and well-annotated set of RNAi pathway genes in the order Coleoptera. To obtain full sequences and corresponding peptides, the 17 genes were searched in the official gene set in BeetleBase (http://www.Beetlebase.org) [17]. A reciprocal blast alignment was performed between the *T. castaneum* RNAi peptides and all *A. glabripennis* peptide sequences. Reciprocal top hits were verified for domain annotations using Prosite. Thirteen sequences were found to be reciprocal top hits between both species (Table S31). The results suggest the presence of both a siRNA and a miRNA pathway in *A. glabripennis*, similar to the pathway present in *T. castaneum*.

**Background**

The RNAi pathway is found in most eukaryotes. Its role is to regulate transcription by means of destructing target mRNA molecules or preventing its translation with the use of small double stranded stretches of RNA (dsRNA). RNAi has an important role in development and in defending cells against viruses and transposons and also in defense against pathogens.

Two important applications of this pathway are functional genomics and pest control. RNAi can be exploited in the laboratory for gene knockdown and consequentially for functional screening of genes either in small or large scale [201]. Libraries of silencing vectors or dsRNA-expressing bacteria designed to target specific genes can be constructed to systematically silence genes and assess loss-of-function genotypes [202-205].

In most animals and plants use of RNAi implicate injection, and transformation approaches. The method was successfully used in several insect-plant systems [206-208]. Among coleopterans, RNAi has been used in the laboratory to silence genes in *T. castaneum* [199], and in the western corn rootworm (*Diabrotica virgifera virgifera*) [209]. Alternatively, host plants expressing dsRNA designed against specific genes in insects have been developed [210]. However, in nematodes and in several insect species it has been shown that soaking in or feeding on dsRNA can be enough to trigger the RNAi pathway [201, 211]. The direct applied implication of this finding is the use of dsRNA for control of insect pests in the field. It has been shown that feeding on dsRNA does trigger RNAi in the western corn rootworm [212] but more studies are necessary to assess the potential of RNAi by feeding in other coleopterans. Understanding the chore machinery behind the RNAi pathway in *A. glabripennis* can unveil the potential of this species for functional studies based on gene silencing and development of control strategies.

**Table S31. *A. glabripennis* genes potentially involved in the insect RNAi pathway.**

| **AGLA identifier** | **Protein product** | **Annotation** |
| --- | --- | --- |
| **AGLA015685-RA** | Endoribonuclease Dicer | Dicer-1 |
| **AGLA013917-RA** | Dicer-2 | Dicer-2 |
| **AGLA019222-RA** | Drosha, ribonuclease type III | Drosha |
| **AGLA000778-RA** | Pasha, partner of drosha-like protein | Pasha |
| **AGLA003298-RA** | Loquacious | Loquacious |
| **AGLA000321-RA** | C3PO | C3PO_component 3 promoter of RISC/A complex of Translin and Trax |
| **AGLA017309-RA** | Protein argonaute-1 | Argonaute-1 |
| **AGLA012768-RA** | Protein argonaute-2b | Argonaute-2b |
| **AGLA010388-RA** | Protein argonaute-3 | Argonaute-3 |
| **AGLA005113-RA** | Sid-likeA | SilA |
| **AGLA015910-RA** | Sid-likeC | SilC |
| **AGLA017225-RA** | Protein Rsd3 | Rsd3 |
| **AGLA013618-RA** | R2D2 | R2D2 |

**VI.9 Cuticular proteins**

**Contributors**

**Andrew J. Rosendale and Joshua B. Benoit** (University of Cincinnati, Department of Biological Sciences, Cincinnati, OH, USA)

**Kristen A. Panfilio (**University of Cologne, Institute for Developmental Biology, Cologne, Germany)

**Summary**

Using sequence motifs established by Willis JH [213] that are characteristic of several families of cuticle proteins, 168 genes coding for putative cuticle proteins were identified from predicted genes for *A. glabripennis* (Table S32). These genes were analyzed with CutProtFam-Pred, a cuticular protein family prediction tool described in [214]; using this approach 153 genes were assigned to one of 6 families (CPR, CPAP1, CPAP3, CPFC, CPF, and TWDL) (Fig. S28). The remaining 17 proteins lacked a defining conserved domain, but possessed characteristics commonly associated with cuticular proteins, including the repeated low-complexity sequences (AAPV/GGY). Many of the genes (~58%) were arranged in clusters of 3 to 25 genes that were type specific (Table S33), which is common for cuticular proteins in other species of insects. Clustering of these genes could allow for the coordinated regulation of cuticle proteins and thereby facilitate the development of insecticide resistance.

Similar to other insects, the CPR family, with both the RR-1 (soft cuticle) and RR-2 (hard cuticle) types, constituted the largest group of cuticle protein genes in the *Anoplophora glabripennis* genome. The 125-member CPR gene family is larger than in *Tribolium castaneum* (89), but smaller than in several mosquito species [146-216] [213]. The number of genes in the protein families CPAP1, CPAP3, CPF, and TWDL were similar to the number in other insects; however, there was a slight expansion in the number of genes in the CPFC family (Fig. S29). Whereas genes in the protein family CLPLCA and CPLCG are present in multiple insect orders, including Coleoptera [214], none of the predicted genes for *A. glabripennis* contained conserved sequences for CPLCA or CPLCG (Additional file 2: Table S34).

For cuticle production, a complete model for a single *chitin synthase* gene was determined (*chs*: Scaffold107:975,779..1,037,075). Expression data support two distinct isoforms based on alternate usage of exons 13 and 14. This is a conserved instance of alternate exon usage that has also been documented in the *T. castaneum, D. melanogaster*, and *Anopheles gambiae* *chitin synthase-1* orthologues [215].

**Author contributions**

A. J. R. performed the sequence and bioinformatics analysis. A. J. R. and J. E. B. contributed to writing and editing. K.A.P contributed to manual annotation and to writing and editing.

**Table S32. Number of genes identified as putative cuticle proteins (per gene family) in the *Anoplophora glabripennis* genome**

| CPR | |  |  |  |  |  |  |  |
| --- | --- | --- | --- | --- | --- | --- | --- | --- |
| RR-1 | RR-2 | CPAP1 | CPAP3 | CPCFC | CPF | TWDL | Unclassified | Total |
| 59 | 66 | 7 | 7 | 4 | 3 | 7 | 15 | 168 |

**Table S33. Clusters of genes encoding cuticle proteins in the *Anoplophora glabripennis* genome.**

|  | **Scaffold #** | **# Genes** | **Family** | **Length (Kbp)** | **Density (Kbp/gene)** |
| --- | --- | --- | --- | --- | --- |
| 1 | 58 | 25 | CRP RR-2 | 683.6 | 27.3 |
| 2 | 233 | 12 | CRP RR-2 | 110.5 | 9.2 |
| 3 | 47 | 12 | CRP RR-1 | 175.1 | 14.6 |
| 4 | 12 | 9 | CRP RR-2 | 115.6 | 12.8 |
| 5 | 152 | 6 | CRP RR-1 | 111.9 | 18.6 |
| 6 | 557 | 6 | CRP RR-1 | 94.3 | 15.7 |
| 7 | 119 | 5 | CRP RR-2 | 54.5 | 10.9 |
| 8 | 430 | 5 | TWDL | 46.9 | 9.4 |
| 9 | 828 | 4 | CPCFC | 84.9 | 21.2 |
| 10 | 376 | 4 | CPAP3 | 93.2 | 23.3 |
| 11 | 37 | 4 | CRP RR-1 | 62.2 | 15.5 |
| 12 | 1053 | 3 | CRP RR-1 | 60.3 | 20.1 |
| 13 | 1590 | 3 | CRP RR-1 | 46.9 | 15.6 |

**Table S34. Cuticle protein encoding genes and their orthologs**

Due to its size, this table has been moved to the supplementary excel file entitled “AdditionalFile 2_04Aug2016”.

**Figure S28. Phylogenetic tree demonstrating relationships between cuticle proteins from *Anoplophora glabripennis*.** The tree was constructed using the neighbor-joining method in MEGA6. Measures of statistical support (percent of 2,000 bootstrap replicates) above 25% are indicated above nodes.


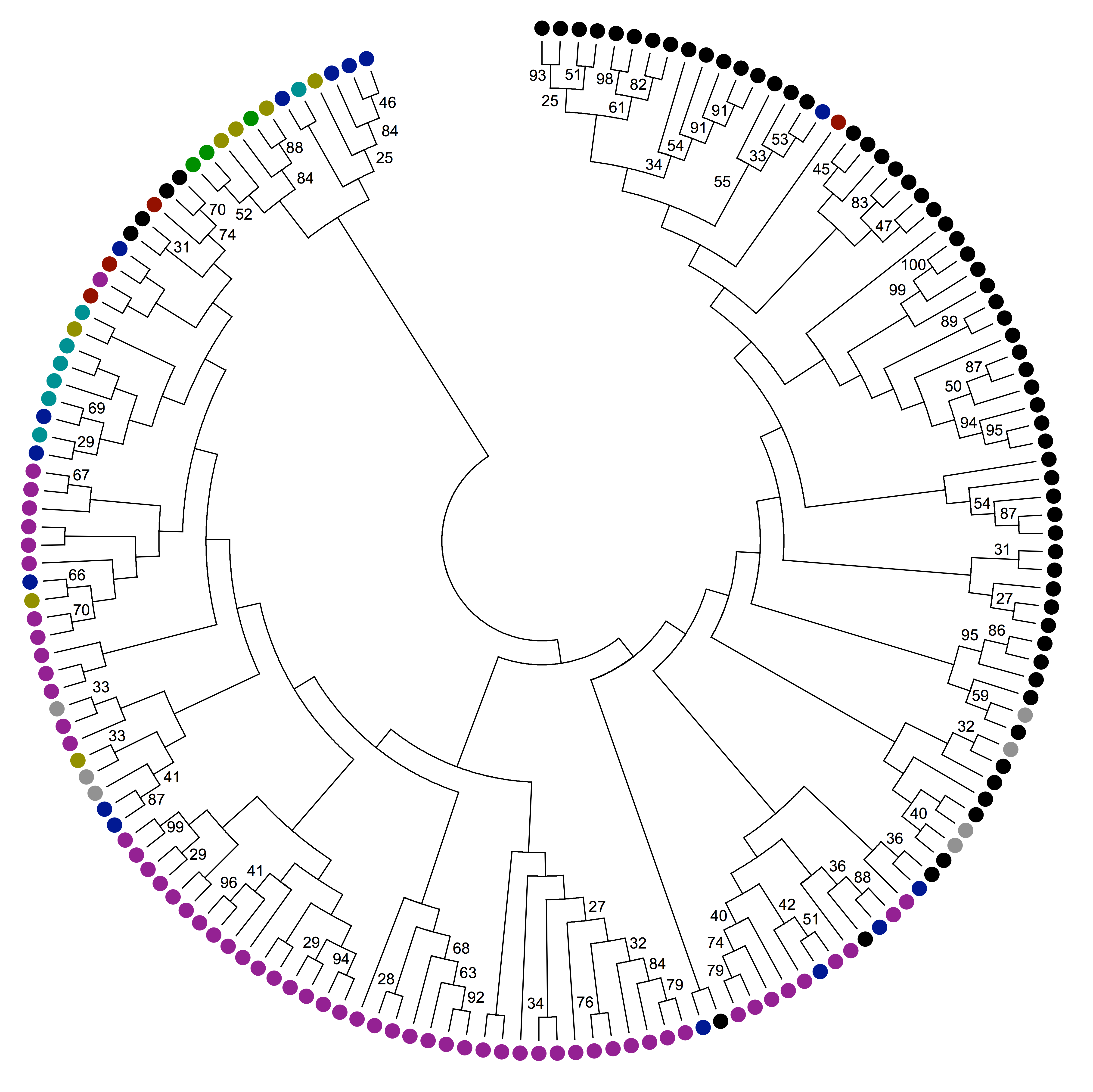


RR-2

RR-1

CPF

CPAP1

Unclassified

TWDL

CPCFC

CPAP3

**Figure S29.** **Phylogenetic tree demonstrating relationships of CPFC genes from *Anoplophora glabripennis* (AGLAB), *Acyrthosiphon pisum* (APISU), *Rhodnius prolixus* (RPROL), *Tribolium castaneum* (TCAST), *Drosophila melanogaster* (DMELO), and *Pediculus humanus* (PHUMA).** The tree was constructed using the neighbor-joining method with Poisson correction in MEGA6. The number of substitutions per amino acid site is represented in the scale bar. Measures of statistical support (percent of 10,000 bootstrap replicates) are indicated above nodes.

AGLAB

APISU

RPROL

TCAST

DMELO

PHUMA

**VI.10 DNA methylation**

**Contributors**

**Karl M. Glastad and Michael A. D. Goodisman** (Georgia Institute of Technology, School of Biology)

**Summary**

Our analyses suggest that *Anoplophora glabripennis* does not possess substantial levels of DNA methylation. Patterns of CpG depletion in *A. glabripennis* were not consistent with those expected for an insect possessing genome methylation and, instead, were very similar to those seen in *Tribolium castaneum*, which does not display appreciable levels of DNA methylation. While *A. glabripennis* apparently possesses an ortholog to the ‘maintenance’ methyltransferase, DNMT1, this ortholog is also present with similar identity in the genome of *T. castaneum*. Furthermore, both beetles’ DNMT1 orthologs showed less identity with outgroup (mammalian) DNMT1 orthologs than for DNMT1 orthologs found in insects with known functional DNA methylation systems. Consequently, conservation of this gene may not be related to the methylation of genomic DNA.

DNA methylation predominantly targets CpG dinucleotides (5’ – 3’ cytosine followed by guanine) in animal genomes. Depletion of normalized CpG content (CpG observed/expected [o/e]) represents an evolutionary signature of DNA methylation in animal genomes because methylated cytosines undergo spontaneous deamination to thymine with high frequency [216].

CpG o/e was calculated for genomic features as P_CpG_ / (P_C_ * P_G_), where P_CpG_, P_C_, and P_G_ are the frequencies of CpG dinucleotides, cytosines, and guanines, respectively [217]. In addition, the corresponding metric, GpC o/e, which controls for GC content, was also calculated using similar methods for genomic features in all taxa, as a control metric. Features with CpG o/e or GpC o/e values of 0 or greater than 6 were excluded from analyses. Short features with a length < 100bp were also not included in analyses.

Sliding-window analyses of CpG o/e across gene positions were conducted using a custom perl script. Window size was set as 200 bp, with a mean CpG o/e value calculated in 20-base increments. Windows were dropped if CpG o/e content was zero for a given window or if greater than 50% of the window was composed of masked (uninformative) bases

In order to test for the existence of DNMT genes in the genome of *A. glabripennis*, we blasted 10 known DNMT3 and DNMT1 orthologs from eight insects (*Apis mellifera, Solenopsis invicta, Harpegnathos saltator, Linepithema humile, Atta cephalotes, Camponotus floridanus, Nasonia vitrepennis,* and *Acyrthosiphon pisum*), as well as two vertebrates (*Homo sapiens, and Danio rerio*), against the gene sets of *T. castaneum*, *Drosophila melanogaster*, *A. glabripennis*, and *A. mellifera* [37]. We took the best hit to each of the 10 orthologs as the putative focal species’ DNMT ortholog. We then averaged the blast statistics among the 10 orthologs with any significant hit.

**Discussion**

We analyzed CpG dinucleotide depletion in the genome of *A. glabripennis* to investigate putative patterns of DNA methylation in its genome. CpG depletion represents an evolutionary signature of DNA methylation resulting from the mutability of methylated cytosines [216, 218-220]. If a genomic feature is methylated, then the observed frequency of CpG dinucleotides will be lower than that expected based on frequency of C and G nucleotides (i.e., the observed to expected CpG content, CpG o/e, will be less than 1.0). However, if a genomic feature is unmethylated then the observed frequency of CpG dinucleotides will equal that expected (i.e., CpG o/e will equal 1.0) [220].

The effects of CpG depletion in the genome are illustrated in Fig. S30D, H, I for *A. mellifera*, which is an insect with substantial, well-documented DNA methylation [221]. *Apis mellifera* displays a notably non-normal distribution of CpG o/e values for genic features, resulting from CpG depletion among a subset of genes that are methylated (low-CpG o/e mode of Fig. S30H; Note that values of CpG o/e > 1.0 in *A. mellifera* are believed to result from gene conversion [222]. This can be contrasted with *D. melanogaster*, an insect known to lack DNA methylation [223], whose genic CpG o/e values fall within a near-normal distribution around the mean of ~1.0 (Fig. S30C, G). Similarly, *T. castaneum* does not display substantial levels of genome methylation [224] and the corresponding distributions of CpG o/e are also approximately normal with means of ~1.0 (Fig. S30B, F).

The genome of *A. glabripennis* shows a somewhat non-normal distribution of genic CpG o/e values (Fig. S30E, specifically a ‘widening’ of the distribution at the lower CpG end). However, there is very little differentiation in the distribution of CpG o/e values among genomic elements, as would be expected for an insect genome lacking DNA methylation [225]. Moreover, the overall patterns of CpG depletion show strong similarity to values observed for *T. castaneum* (*cf*. Fig. S30A, E, F with Fig. S30B, F, J).

Furthermore, when a sliding window is used to investigate CpG o/e values across gene bodies (Fig. S30I-L), insects that possess substantial DNA methylation, such as *A.* *mellifera*, display a notable depletion of CpG’s along the gene frames (particularly in 5’ regions) of genes falling *below* the mean genic CpG o/e value (Fig. S30L). This differs considerably from the CpG o/e pattern for genes whose CpG o/e values fall *above* the mean of all genes (Fig. S30L). However, among species lacking DNA methylation, both low- and high- CpG o/e genes show similar (but offset) patterns of CpG depletion due to a lack of preferential dinucleotide depletion among the low-CpG o/e class of genes. In addition, the magnitude of difference between low- and high- CpG o/e values at each position is much lower in these species (*cf.* Fig. S30J, K with Fig. S30L). Notably the patterns of CpG depletion across *A. glabripennis* genes (Fig. S30I) are very similar to those found in other insects that lack DNA methylation (Fig. S30J, K). Thus, analysis of CpG o/e values provides no evidence that *A. glabripennis* possesses extensive levels of DNA methylation.

Finally, we queried the gene set of *A. glabripennis* for the DNA methyltransferase enzymes, DNMT1 and DNMT3, which are putatively involved in functional DNA methylation systems [225]. As expected, we found that *D. melanogaster* lacks both DNMT1 and DNMT3 (E-value > 3e-8). Both *T. castaneum* and *A. glabripennis* also apparently lack DNMT3 (E-value > 3e-8) (we searched in both the unannotated portions of the genome assembly, and in the unassembled raw reads). Surprisingly, though, both beetles showed evidence of possessing the DNMT1 gene (median E-value = 0). However, both beetles’ DNMT1 orthologs showed considerably lower percent identity with *H. sapiens* DNMT1 than found for *A. mellifera* (median % identity = 37.1 vs 61.6 for *A. glabripennis* and *A. mellifera*, respectively). Thus it appears that *A. glabripennis* does not possess a DNMT3 gene, but does possess some type of DNMT1 gene. However, the function of this DNMT1 gene in *A. glabripennis*, as well as *T. castaneum*, remains unclear.

**Figure S30**. **CpG o/e analysis of *Anoplophora glabripennis* shows little evidence of DNA methylation.**

**(A-D)** The mean CpG o/e value for each genomic feature (exon, intron, gene = region between start and stop codon, and genome = 1kb windows of all non-genic regions). Bolded and dimmed DNMT names indicate the presence or absence of the given DNMT, respectively.

**(E-H)** The comparison of CpG o/e density plots for each genomic feature.

**(I-L)** Spatial plots of mean CpG o/e values generated from sliding window analysis of genes and proximal regions. Vertical dashed lines indicate start codon and stop codon.

**
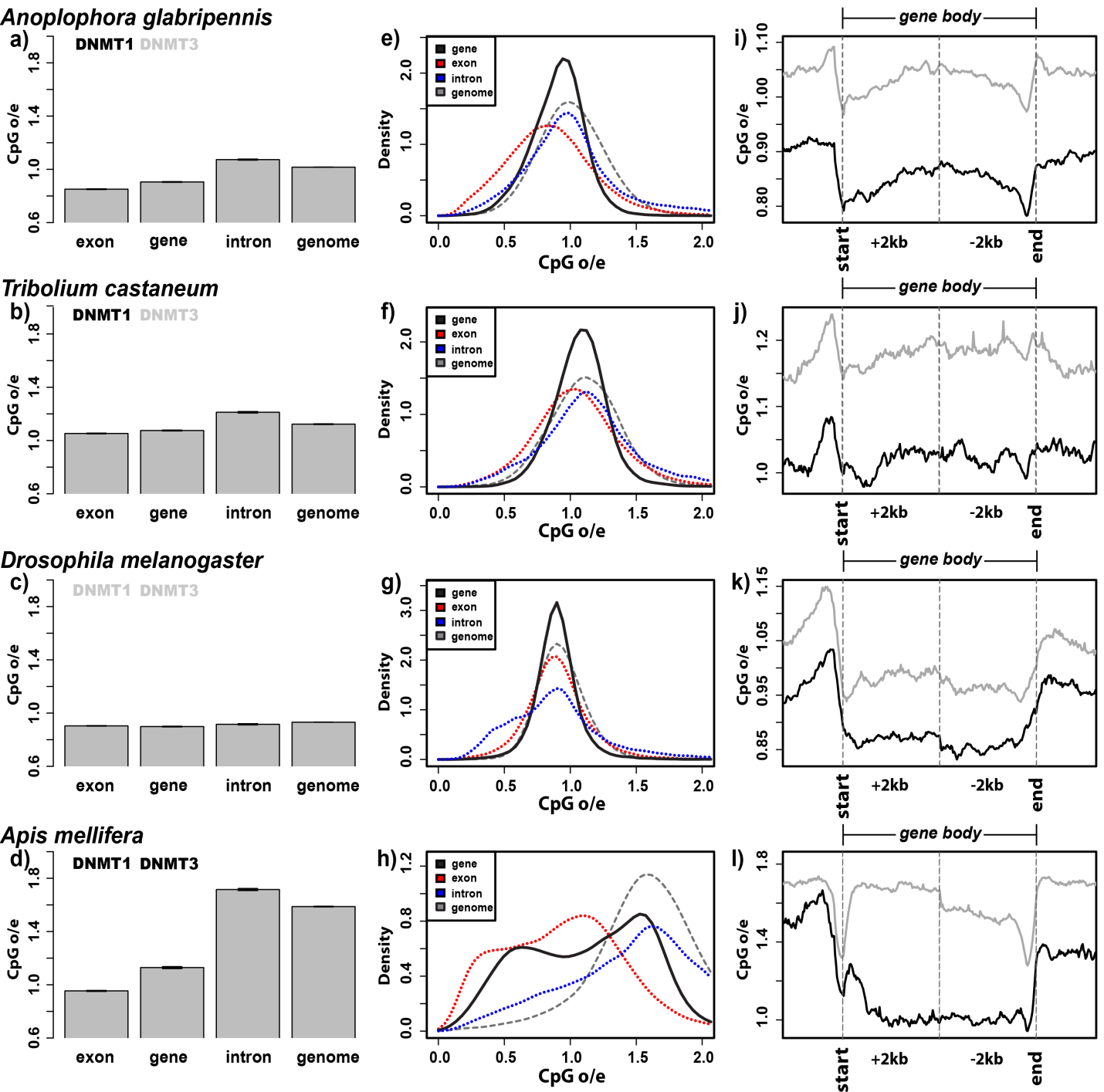
**

**VII. Diapause and Stress Tolerance**

**Contributors**

**George Yocum, Anna K. Bennett, Joseph P. Rinehart, Richard L. Roehrdanz** (USDA-ARS, Insect Genetics and Biochemistry Research)

**Julia H. Bowsher, Alex C. Torson (**North Dakota State University, Department of Biological Sciences**)**

**Summary**To survive reoccurring environmental stress such as cold or dry seasons, many insects employ the neuroendocrine controlled state of diapause [226], which is characterized by developmental arrest, decreased metabolism [227], and an increase in resistance to stresses including increased cold-hardiness [228, 229], all of which are under environmental control.

Several key components of the genetic mechanisms underlying diapause in other species were also found in the *Anoplophora glabripennis* genome. One such component is the insulin signaling pathway, which is essential for the regulation of diapause [230], and also plays a role in the regulation of development [231], growth [232], and reproduction [233]. The multi-gene insulin-like peptide (ILP) family is pivotal to these signaling cascades [234]. Analysis of the *A. glabripennis* genome indicates that this species has genes encoding seven insulin-like peptides ranging in size from 101-131 amino acids, which can be grouped according to their similarity to different ILPs from *Tribolium castaneum*. Four of the peptides form a cluster that are similar to the same ILP from *T. castaneum* (EFA02918). A second group consists of two peptides which are identical to each other, but have different flanking regions, and are similar to another *T. castaneum* ILP (EFA02796). The final peptide is the most divergent, although it does contain similarity to a third *T. castaneum* ILP (XP 008201457). Since the processing, storage, and release of ILPs remain uncharacterized in most insect species [234], further work will be required to develop a comprehensive view of the role that ILPs play in regulating diapause in *A. glabripennis*. Other components of the insulin-signaling pathway that were annotated in *A. glabripennis* included two genes with significant similarity to the insulin receptor and one gene for FOXO. The full characterization of the insulin regulatory pathway in *A. glabripennis* will greatly contribute to the understanding of regulatory processes in this species.

A second critical component of diapause in many species is the expression of heat shock proteins (hsps), a well characterized category of proteins initially identified for their role in stress responses [235, 236]. One physiological role of the hsps during diapause is enabling insects to survive low-temperature stress [162, 237, 238]. Analysis of the *A. glabripennis* genome indicates the presence of genes belonging to several hsp families including 13 members of the small hsp family, 25 genes containing the DnaJ domain (possible hsp40 family), 1 member of the hsp60 family, 14 members of the hsp70 family, and 1 member of the hsp90 family. As with the ILP genes, hsps are differentially expressed during diapause [237, 238]. The *A. glabripennis* genome was also screened for the presence of genes with sequence similarity to the cold-tolerance genes, Frost [239, 240] and antifreeze proteins [241, 242] but no genes were identified. Developing a comprehensive understanding of *A. glabripennis* diapause and environmental stress-regulated genes will provide insights into the mechanisms that enable this beetle to respond to environmental challenges and therefore determine its potential geographic range.

**VIII. Life Cycle**

**Figure S31 (Fig. 1, main paper).** (**A)** *A. glabripennis* life cycle (adapted from M. Bohne, used with permission; image of adult female courtesy of Barbara Strnadova, used with permission), **(B)** wood dissected to expose feeding *A. glabripennis* larva (image courtesy of Kelli Hoover, used with permission), **(C, D)** adults (images courtesy of Damon Crook, used with permission). Early stage larvae are specialized wood-borers, feeding in galleries under the bark of host trees (in the subcortical tissue and phloem). Larger, later stage larvae tunnel deep into the heartwood (mature xylem) of their hosts, where they continue feeding and complete development [243]. Adults are comparatively short-lived external feeders, consuming small amounts of tissue from host leaves and twigs. *A. glabripennis* is broadly polyphagous on woody angiosperms. It is native to eastern Asia, but has recently become established in several countries in North America, Europe, and beyond, via solid wood packing material. *A. glabripennis* is a high profile invasive pest species capable of inflicting severe damage on its hosts, which include many important orchard, ornamental and forest tree species. It is therefore a globally significant ecological and economic pest, whose economic impact in the U.S. alone, if uncontrolled, has been conservatively estimated at $889^[[2]](#footnote-2)^ billion [244]. *A. glabripennis* is capable of attacking both healthy and susceptible trees [245], and is broadly polyphagous, feeding on at least 100 species of woody angiosperms worldwide [114, 243, 246].

**
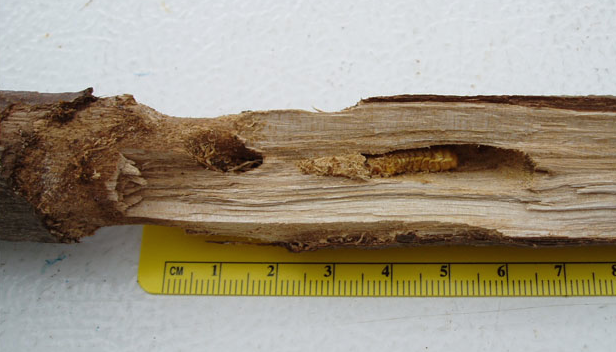
**

A

B

B.


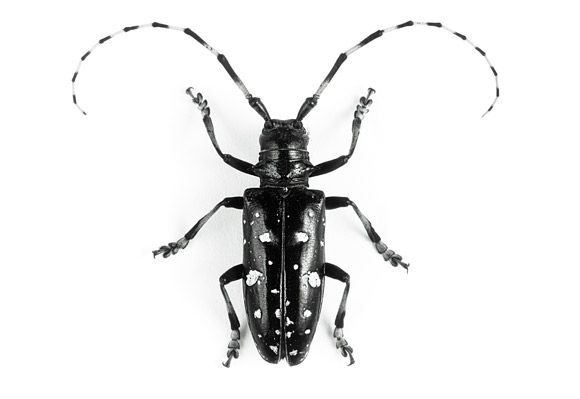

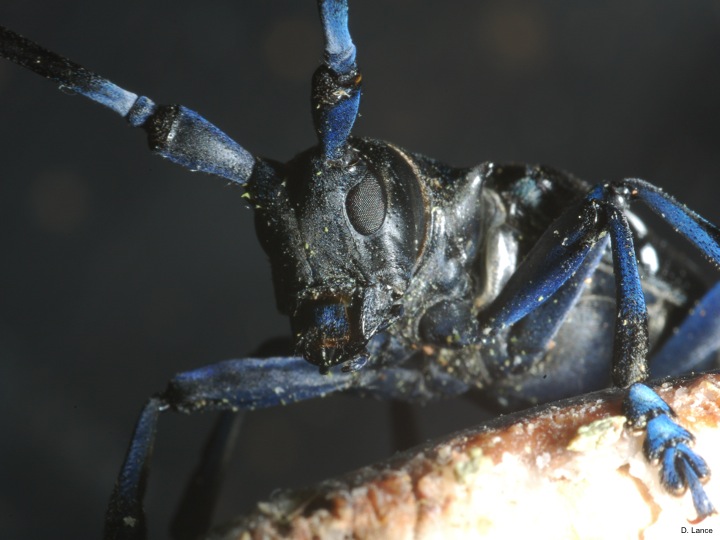
**
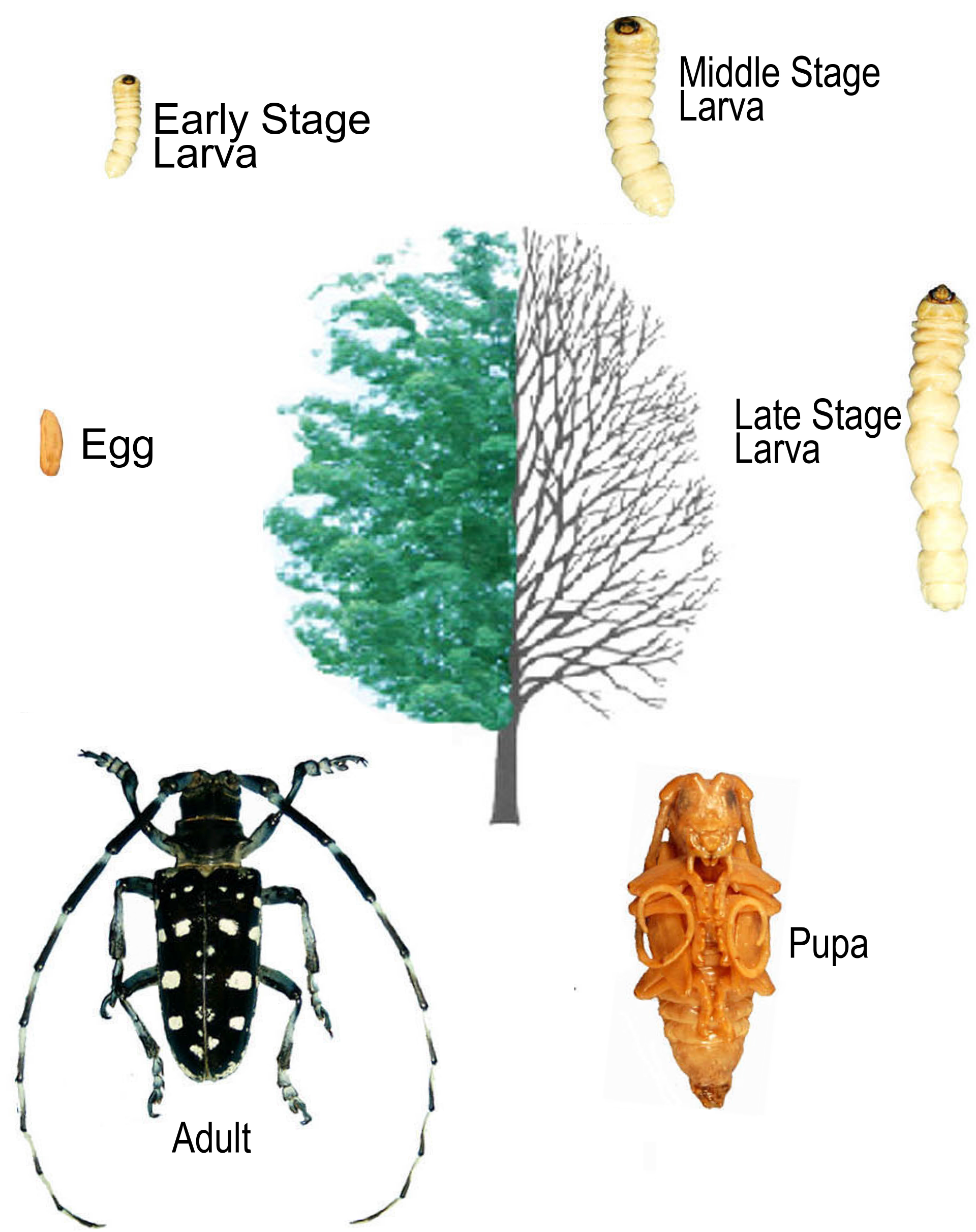
**

C

**
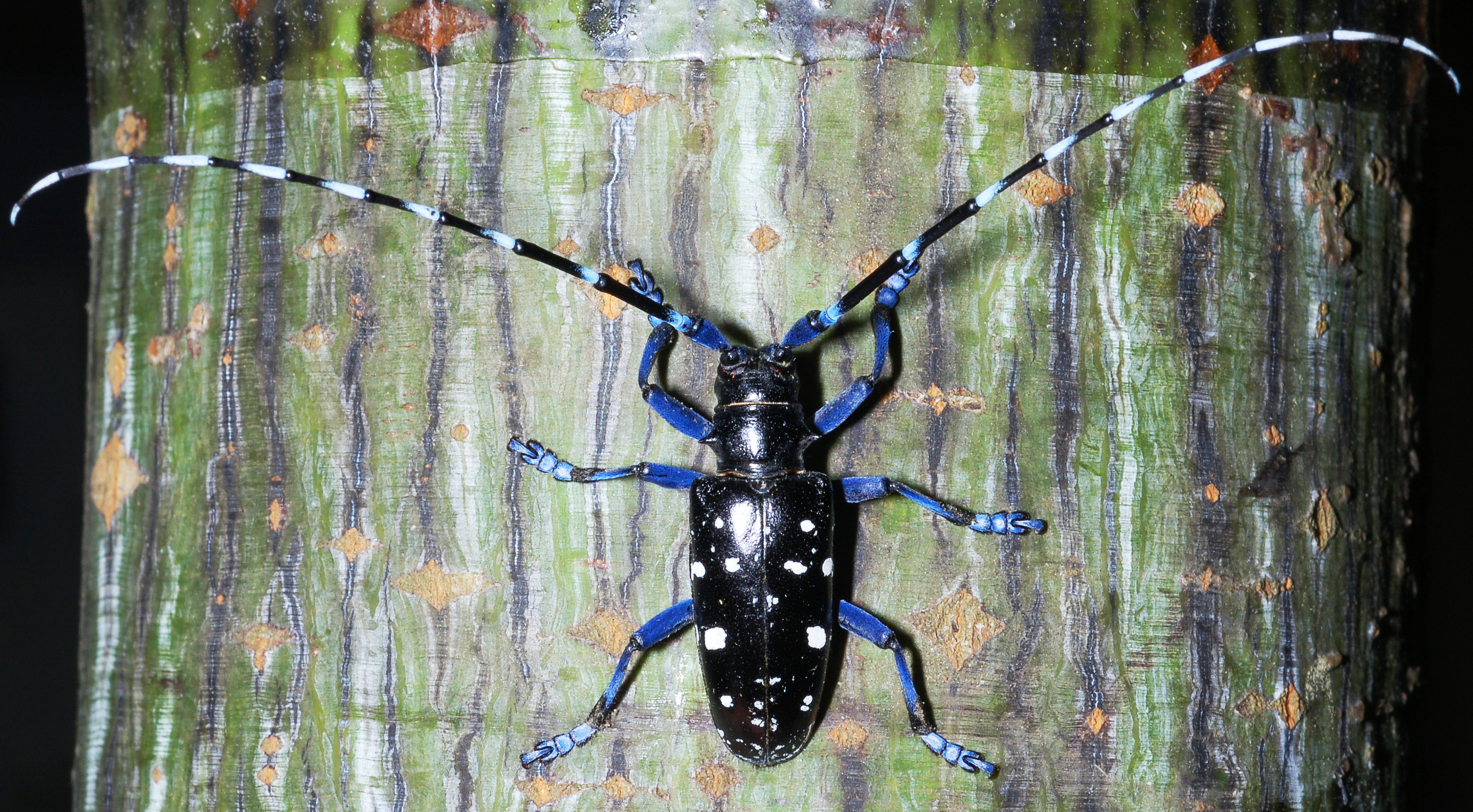
**

D

**IX. Supplemental References**

1. Hare EE, Johnston JS. Genome size determination using flow cytometry of propidium iodide-stained nuclei. Methods Mol Biol. 2011;772**:**3–12.

2. Gnerre S, Maccallum I, Przybylski D, Ribeiro FJ, Burton JN, Walker BJ, Sharpe T, Hall G, Shea TP, Sykes S, et al. High-quality draft assemblies of mammalian genomes from massively parallel sequence data. Proc Natl Acad Sci U S A. 2011;108**:**1513–8.

3. Holt C, Yandell M. MAKER2: an annotation pipeline and genome-database management tool for second-generation genome projects. BMC Bioinf. 2011;12**:**491.

4. Stanke M, Diekhans M, Baertsch R, Haussler D. Using native and syntenically mapped cDNA alignments to improve de novo gene finding. Bioinformatics. 2008;24**:**637–44.

5. Korf I. Gene finding in novel genomes. BMC Bioinf. 2004;14**:**59.

6. Lee E, Helt GA, Reese JT, Munoz-Torres MC, Childers CP, Buels RM, Stein L, Holmes IH, Elsik CG, Lewis SE. Web Apollo: a web-based genomic annotation editing platform. Genome Biol. 2013;14**:**R93.

7. Slater GS, Birney E. Automated generation of heuristics for biological sequence comparison. BMC Bioinf. 2005;6**:**31.

8. Poelchau M, Childers C, Moore G, Tsavatapalli V, Evans J, Lee CY, Lin H, Lin JW, Hackett K. The i5k Workspace@NAL--enabling genomic data access, visualization and curation of arthropod genomes. Nucleic Acids Res. 2015;43**:**D714–9.

9. Skinner ME, Uzilov AV, Stein LD, Mungall CJ, Holmes IH. JBrowse: a next-generation genome browser. Genome Res. 2009;19**:**1630–8.

10. Engsontia P, Sanderson AP, Cobb M, Walden KKO, Robertson HM, Brown S. The red flour beetle's large nose: An expanded odorant receptor gene family in *Tribolium* *castaneum*. Insect Biochem Mol Biol. 2008;38**:**387–97.

11. Hill CA, Fox AN, Pitts RJ, Kent LB, Tan PL, Chrystal MA, Cravchik A, Collins FH, Robertson HM, Zwiebel LJ. G protein-coupled receptors in *Anopheles gambiae*. Science. 2002;298**:**176–8.

12. Kent LB, Walden KKO, Robertson HM. The Gr family of candidate gustatory and olfactory receptors in the yellow-fever mosquito *Aedes aegypti*. Chem Senses. 2008;33**:**79–93.

13. Robertson HM, Gadau J, Wanner KW. The insect chemoreceptor superfamily of the parasitoid jewel wasp *Nasonia vitripennis*. Insect Mol Biol. 2010;19 Suppl 1**:**121–36.

14. Cantarel BL, Korf I, Robb SM, Parra G, Ross E, Moore B, Holt C, Sanchez Alvarado A, Yandell M. MAKER: an easy-to-use annotation pipeline designed for emerging model organism genomes. Genome Res. 2008;18**:**188–96.

15. Yandell M, Ence D. A beginner's guide to eukaryotic genome annotation. Nat Rev Genet. 2012;13**:**329–42.

16. Keeling CI, Yuen MM, Liao NY, Docking TR, Chan SK, Taylor GA, Palmquist DL, Jackman SD, Nguyen A, Li M, et al. Draft genome of the mountain pine beetle, *Dendroctonus ponderosae* Hopkins, a major forest pest. Genome Biol. 2013;14**:**R27.

17. Richards S, Gibbs RA, Weinstock GM, Brown SJ, Denell R, Beeman RW, Gibbs R, Beeman RW, Brown SJ, Bucher G, et al. The genome of the model beetle and pest *Tribolium castaneum*. Nature. 2008;452**:**949–55.

18. Terrapon N, Li C, Robertson HM, Ji L, Meng X, Booth W, Chen Z, Childers CP, Glastad KM, Gokhale K, et al. Molecular traces of alternative social organization in a termite genome. Nat Commun. 2014;5**:**3636.

19. Kirkness EF, Haas BJ, Sun W, Braig HR, Perotti MA, Clark JM, Lee SH, Robertson HM, Kennedy RC, Elhaik E, et al. Genome sequences of the human body louse and its primary endosymbiont provide insights into the permanent parasitic lifestyle. Proc Natl Acad Sci U S A. 2010;107**:**12168–73.

20. International Aphid Genomics Consortium. Genome sequence of the pea aphid *Acyrthosiphon pisum*. PLoS Biol. 2010;8**:**e1000313.

21. Elsik CG, Worley KC, Bennett AK, Beye M, Camara F, Childers CP, de Graaf DC, Debyser G, Deng J, Devreese B, et al. Finding the missing honey bee genes: Lessons learned from a genome upgrade. BMC Genomics. 2014;15**:**86.

22. Weinstock GM, Robinson GE, Gibbs RA, Worley KC, Evans JD, Maleszka R, Robertson HM, Weaver DB, Beye M, Bork P, et al. Insights into social insects from the genome of the honeybee *Apis mellifera*. Nature. 2006;443**:**931–49.

23. Werren JH, Richards S, Desjardins CA, Niehuis O, Gadau J, Colbourne JK, Nasonia Genome Working G, Werren JH, Richards S, Desjardins CA, et al. Functional and evolutionary insights from the genomes of three parasitoid *Nasonia* species. Science. 2010;327**:**343–8.

24. You M, Yue Z, He W, Yang X, Yang G, Xie M, Zhan D, Baxter SW, Vasseur L, Gurr GM, et al. A heterozygous moth genome provides insights into herbivory and detoxification. Nat Genet. 2013;45**:**220–5.

25. Zhan S, Merlin C, Boore JL, Reppert SM. The monarch butterfly genome yields insights into long-distance migration. Cell. 2011;147**:**1171–85.

26. Zhao C, Escalante LN, Chen H, Benatti TR, Qu J, Chellapilla S, Waterhouse RM, Wheeler D, Andersson MN, Bao R, et al. A massive expansion of effector genes underlies gall-formation in the wheat pest *Mayetiola destructor*. Curr Biol. 2015;25**:**613–20.

27. Adams MD, Celniker SE, Holt RA, Evans CA, Gocayne JD, Amanatides PG, Scherer SE, Li PW, Hoskins RA, Galle RF, et al. The genome sequence of *Drosophila melanogaster*. Science. 2000;287**:**2185–95.

28. Holt RA, Subramanian GM, Halpern A, Sutton GG, Charlab R, Nusskern DR, Wincker P, Clark AG, Ribeiro JM, Wides R, et al. The genome sequence of the malaria mosquito *Anopheles gambiae*. Science. 2002;298**:**129–49.

29. Kriventseva EV, Tegenfeldt F, Petty TJ, Waterhouse RM, Simao FA, Pozdnyakov IA, Ioannidis P, Zdobnov EM. OrthoDB v8: update of the hierarchical catalog of orthologs and the underlying free software. Nucleic Acids Res. 2015;43**:**D250–6.

30. Stamatakis A. RAxML-VI-HPC: maximum likelihood-based phylogenetic analyses with thousands of taxa and mixed models. Bioinformatics. 2006;22**:**2688–90.

31. Edgar RC. MUSCLE: multiple sequence alignment with high accuracy and high throughput. Nucleic Acids Res. 2004;32**:**1792–7.

32. Capella-Gutierrez S, Silla-Martinez JM, Gabaldon T. trimAl: a tool for automated alignment trimming in large-scale phylogenetic analyses. Bioinformatics. 2009;25**:**1972–3.

33. Rice P, Longden I, Bleasby A. EMBOSS: the European Molecular Biology Open Software Suite. Trends Genet. 2000;16**:**276–7.

34. Stover BC, Muller KF. TreeGraph 2: combining and visualizing evidence from different phylogenetic analyses. BMC Bioinf. 2010;11**:**7.

35. Huson DH, Scornavacca C. Dendroscope 3: an interactive tool for rooted phylogenetic trees and networks. Syst Biol. 2012;61**:**1061–7.

36. Simão FA, Waterhouse RM, Ioannidis P, Kriventseva EV, Zdobnov EM. BUSCO: assessing genome assembly and annotation completeness with single-copy orthologs. Bioinformatics. 2015;31**:**3210–2.

37. Altschul SF, Gish W, Miller W, Myers EW, Lipman DJ. Basic Local Alignment Search Tool. J Mol Biol. 1990;215**:**403–10.

38. R_Core_Team: R: a language and environment for statistical computing. R Foundation for Statistical Computing, Vienna, Austria. URL <http://www.R-project.org/>. 2013.

39. Zheng CF, Sankoff D. On the PATHGROUPS approach to rapid small phylogeny. BMC Bioinf. 2011;12**:**54.

40. Wheeler D, Redding AJ, Werren JH. Characterization of an ancient lepidopteran lateral gene transfer. PLoS One. 2013;8**:**e59262.

41. Marvaldi AE, Duckett CN, Kjer KM, Gillespie JJ. Structural alignment of 18S and 28S rDNA sequences provides insights into phylogeny of Phytophaga (Coleoptera: Curculionoidea and Chrysomeloidea). Zool Scr. 2009;38**:**63–77.

42. Wei YD, Lee KS, Gui ZZ, Yoon HJ, Kim I, Zhang GZ, Guo XJ, Sohn HD, Jin BR. Molecular cloning, expression, and enzymatic activity of a novel endogenous cellulase from the mulberry longicorn beetle, *Apriona germari*. Comp Biochem Phys B. 2006;145**:**220–9.

43. Sugimura M, Watanabe H, Lo N, Saito H. Purification, characterization, cDNA cloning and nucleotide sequencing of a cellulase from the yellow-spotted longicorn beetle, *Psacothea hilaris*. Eur J Biochem. 2003;270**:**3455–60.

44. Lee SJ, Kim SR, Yoon HJ, Kim I, Lee KS, Je YH, Lee SM, Seo SJ, Sohn HD, Jin BR. cDNA cloning, expression, and enzymatic activity of a cellulase from the mulberry longicorn beetle, *Apriona germari*. Comp Biochem Phys B. 2004;139**:**107–16.

45. Chang CJ, Wu CP, Lu SC, Chao AL, Ho THD, Yu SM, Chao YC. A novel exo-cellulase from white spotted longhorn beetle (*Anoplophora malasiaca*). Insect Biochem Mol Biol. 2012;42**:**629–36.

46. Calderon-Cortes N, Watanabe H, Cano-Camacho H, Zavala-Paramo G, Quesada M. cDNA cloning, homology modelling and evolutionary insights into novel endogenous cellulases of the borer beetle *Oncideres albomarginata chamela* (Cerambycidae). Insect Mol Biol. 2010;19**:**323–36.

47. Pauchet Y, Kirsch R, Giraud S, Vogel H, Heckel DG. Identification and characterization of plant cell wall degrading enzymes from three glycoside hydrolase families in the cerambycid beetle *Apriona japonica*. Insect Biochem Mol Biol. 2014;49**:**1–13.

48. Scully ED, Hoover K, Carlson JE, Tien M, Geib SM. Midgut transcriptome profiling of *Anoplophora glabripennis*, a lignocellulose degrading cerambycid beetle. Bmc Genomics. 2013;14.

49. Cantarel BL, Coutinho PM, Rancurel C, Bernard T, Lombard V, Henrissat B. The Carbohydrate-Active EnZymes database (CAZy): an expert resource for glycogenomics. Nucleic Acids Res. 2009;37**:**D233–8.

50. Beran F, Pauchet Y, Kunert G, Reichelt M, Wielsch N, Vogel H, Reinecke A, Svatos A, Mewis I, Schmid D, et al. *Phyllotreta striolata* flea beetles use host plant defense compounds to create their own glucosinolate-myrosinase system. Proc Natl Acad Sci U S A. 2014;111**:**7349–54.

51. Pauchet Y, Kirsch R, Giraud S, Vogel H, Heckel DG. Identification and characterization of plant cell wall degrading enzymes from three glycoside hydrolase families in the cerambycid beetle *Apriona japonica*. Insect Biochem Mol Biol. 2014;49**:**1–13.

52. Geib SM, Tien M, Hoover K. Identification of proteins involved in lignocellulose degradation using in gel zymogram analysis combined with mass spectroscopy-based peptide analysis of gut proteins from larval Asian longhorned beetles, *Anoplophora glabripennis*. Insect Sci. 2010;17**:**253–64.

53. Davison A, Blaxter M. Ancient origin of glycosyl hydrolase family 9 cellulase genes. Mol Biol Evol. 2005;22**:**1273–84.

54. Eyun SI, Wang HC, Pauchet Y, Ffrench-Constant RH, Benson AK, Valencia-Jimenez A, Moriyama EN, Siegfried BD. Molecular evolution of glycoside hydrolase genes in the western corn rootworm (*Diabrotica virgifera virgifera*). PLoS One. 2014;9**:**e102818.

55. Palomares-Rius JE, Hirooka Y, Tsai IJ, Masuya H, Hino A, Kanzaki N, Jones JT, Kikuchi T. Distribution and evolution of glycoside hydrolase family 45 cellulases in nematodes and fungi. BMC Evol Biol. 2014;14**:**69.

56. Scully ED, Geib SM, Carlson JE, Tien M, McKenna D, Hoover K. Functional genomics and microbiome profiling of the Asian longhorned beetle (*Anoplophora glabripennis*) reveal insights into the digestive physiology and nutritional ecology of wood feeding beetles. BMC Genomics. 2014;15**:**1096.

57. Scully ED, Geib SM, Hoover K, Tien M, Tringe SG, Barry KW, Glavina del Rio T, Chovatia M, Herr JR, Carlson JE. Metagenomic profiling reveals lignocellulose degrading system in a microbial community associated with a wood-feeding beetle. PLoS One. 2013;8**:**e73827.

58. Scully ED, Hoover K, Carlson JE, Tien M, Geib SM. Midgut transcriptome profiling of *Anoplophora glabripennis*, a lignocellulose degrading cerambycid beetle. BMC Genomics. 2013;14**:**850.

59. Kirsch R, Gramzow L, Theissen G, Siegfried BD, Ffrench-Constant RH, Heckel DG, Pauchet Y. Horizontal gene transfer and functional diversification of plant cell wall degrading polygalacturonases: Key events in the evolution of herbivory in beetles. Insect Biochem Mol Biol. 2014;52**:**33–50.

60. Keena MA. Pourable artificial diet for rearing *Anoplophora glabripennis* (Coleoptera : Cerambycidae) and methods to optimize larval survival and synchronize development. Ann Entomol Soc Am. 2005;98**:**536–47.

61. Intra J, Pavesi G, Horner DS. Phylogenetic analyses suggest multiple changes of substrate specificity within the Glycosyl hydrolase 20 family. BMC Evol Biol. 2008;8.

62. Scully ED, Hoover K, Carlson J, Tien M, Geib SM. Proteomic analysis of *Fusarium solani* isolated from the Asian longhorned beetle, *Anoplophora glabripennis*. PLoS One. 2012;7**:**e32990.

63. Dereeper A, Guignon V, Blanc G, Audic S, Buffet S, Chevenet F, Dufayard JF, Guindon S, Lefort V, Lescot M, et al. Phylogeny.fr: robust phylogenetic analysis for the non-specialist. Nucleic Acids Res. 2008;36**:**W465–9.

64. Ronquist F, Huelsenbeck JP. MrBayes 3: Bayesian phylogenetic inference under mixed models. Bioinformatics. 2003;19**:**1572–4.

65. Tamura K, Peterson D, Peterson N, Stecher G, Nei M, Kumar S. MEGA5: Molecular evolutionary genetics analysis using maximum likelihood, evolutionary distance, and maximum parsimony methods. Mol Biol Evol. 2011;28**:**2731–9.

66. Voragen AGJ, Schols HA, Pilnik W. Determination of the degree of methylation and acetylation of pectins by h.p.l.c. Food Hydrocolloid. 1986;1**:**65–70.

67. Yoo SH, Fishman ML, Savary BJ, Hotchkiss AT. Monovalent salt-induced gelation of enzymatically deesterified pectin. J Agr Food Chem. 2003;51**:**7410–7.

68. Highley TL. Carbohydrolase Assays**.** In: Dashek WV (editor). *Methods in Plant Biochemistry and Molecular Biology.* Boca Raton, FL: CRC Press; 1997. 309–21.

69. Lee SJ, Lee KS, Kim SR, Gui ZZ, Kim YS, Yoon HJ, Kim I, Kang PD, Sohn HD, Jin BR. A novel cellulase gene from the mulberry longicorn beetle, *Apriona germari*: gene structure, expression, and enzymatic activity. Comp Biochem Phys B. 2005;140**:**551–60.

70. Willis JD, Oppert B, Oppert C, Klingeman WE, Jurat-Fuentes JL. Identification, cloning, and expression of a GHF9 cellulase from *Tribolium castaneum* (Coleoptera: Tenebrionidae). J Insect Physiol. 2011;57**:**300–6.

71. Brett CT, Waldron KW. The molecular components of the wall**.** In: Brett CT, Waldron KW (editors). *hysiology and Biochemistry of Plant Cell Walls.* Springer; 1996. ??

72. O'Neill MA, York WS. The composition and structure of plant primary cell walls**.** In: Rose JKC (editor). *The Plant Cell Wall.* Blackwell; 2003. 1–54.

73. Beguin P. Detection of cellulase activity in polyacrylamide gels using Congo red-stained agar replicas. Anal Biochem. 1983;131**:**333–6.

74. Danchin EGJ, Rosso MN, Vieira P, de Almeida-Engler J, Coutinho PM, Henrissat B, Abad P. Multiple lateral gene transfers and duplications have promoted plant parasitism ability in nematodes. Proc Natl Acad Sci U S A. 2010;107**:**17651–6.

75. Rybarczyk-Mydlowska K, Maboreke HR, van Megen H, van den Elsen S, Mooyman P, Smant G, Bakker J, Helder J. Rather than by direct acquisition via lateral gene transfer, GHF5 cellulases were passed on from early Pratylenchidae to root-knot and cyst nematodes. BMC Evol Biol. 2012;12**:**221.

76. Todaka N, Inoue T, Saita K, Ohkuma M, Nalepa CA, Lenz M, Kudo T, Moriya S. Phylogenetic analysis of cellulolytic enzyme genes from representative lineages of termites and a related cockroach. PLoS One. 2010;5**:**e8636.

77. Aspeborg H, Coutinho PM, Wang Y, Brumer H, Henrissat B. Evolution, substrate specificity and subfamily classification of glycoside hydrolase family 5 (GH5). BMC Evol Biol. 2012;12**:**186.

78. Ohtoko K, Ohkuma M, Moriya S, Inoue T, Usami R, Kudo T. Diverse genes of cellulase homologues of glycosyl hydrolase family 45 from the symbiotic protists in the hindgut of the termite *Reticulitermes speratus*. Extremophiles. 2000;4**:**343–9.

79. Pauchet Y, Wilkinson P, Chauhan R, Ffrench-Constant RH. Diversity of beetle genes encoding novel plant cell wall degrading enzymes. PLoS One. 2010;5**:**1–8.

80. Xu B, Hellman U, Ersson B, Janson JC. Purification, characterization and amino-acid sequence analysis of a thermostable, low molecular mass endo-beta-1,4-glucanase from blue mussel, *Mytilus edulis*. Eur J Biochem. 2000;267**:**4970–7.

81. Kikuchi T, Cotton JA, Dalzell JJ, Hasegawa K, Kanzaki N, McVeigh P, Takanashi T, Tsai IJ, Assefa SA, Cock PJA, et al. Genomic insights into the origin of parasitism in the emerging plant pathogen *Bursaphelenchus xylophilus*. PLoS Pathog. 2011;7**:**e1002219.

82. Steenbakkers PJ, Freelove A, Van Cranenbroek B, Sweegers BM, Harhangi HR, Vogels GD, Hazlewood GP, Gilbert HJ, Op den Camp HJ. The major component of the cellulosomes of anaerobic fungi from the genus *Piromyces* is a family 48 glycoside hydrolase. DNA Sequence. 2002;13**:**313–20.

83. Wang TY, Chen HL, Lu MY, Chen YC, Sung HM, Mao CT, Cho HY, Ke HM, Hwa TY, Ruan SK, et al. Functional characterization of cellulases identified from the cow rumen fungus *Neocallimastix patriciarum* W5 by transcriptomic and secretomic analyses. Biotechnol Biofuels. 2011;4**:**24.

84. Fujita K, Shimomura K, Yamamoto K, Yamashita T, Suzuki K. A chitinase structurally related to the glycoside hydrolase family 48 is indispensable for the hormonally induced diapause termination in a beetle. Biochem Bioph Res Co. 2006;345**:**502–7.

85. Sezutsu H, Le Goff G, Feyereisen R. Origins of P450 diversity. Philo Trans R Soc, B. 2013;368**:**20120428.

86. Rewitz KF, O'Connor MB, Gilbert LI. Molecular evolution of the insect Halloween family of cytochrome P450s: phylogeny, gene organization and functional conservation. Insect Biochem Mol Biol. 2007;37**:**741–53.

87. Qiu Y, Tittiger C, Wicker-Thomas C, Le Goff G, Young S, Wajnberg E, Fricaux T, Taquet N, Blomquist GJ, Feyereisen R. An insect-specific P450 oxidative decarbonylase for cuticular hydrocarbon biosynthesis. Proc Natl Acad Sci U S A. 2012;109**:**14858–63.

88. Bock KW. Vertebrate UDP-glucuronosyltransferases: functional and evolutionary aspects. Biochem Pharmacol. 2003;66**:**691–6.

89. Ahn SJ, Vogel H, Heckel DG. Comparative analysis of the UDP-glycosyltransferase multigene family in insects. Insect Biochem Mol Biol. 2012;42**:**133–47.

90. Mackenzie PI, Bock KW, Burchell B, Guillemette C, Ikushiro S, Iyanagi T, Miners JO, Owens IS, Nebert DW. Nomenclature update for the mammalian UDP glycosyltransferase (UGT) gene superfamily. Pharmacogenet Genom. 2005;15**:**677–85.

91. Wang XH, Fang XD, Yang PC, Jiang XT, Jiang F, Zhao DJ, Li BL, Cui F, Wei JN, Ma CA, et al. The locust genome provides insight into swarm formation and long-distance flight. Nat Commun. 2014;5**:**1–9.

92. Lü FG, Fu KY, Li Q, Guo WC, Ahmat T, Li GQ. Identification of carboxylesterase genes and their expression profiles in the Colorado potato beetle *Leptinotarsa decemlineata* treated with fipronil and cyhalothrin. Pestic Biochem Phys. 2015;122**:**86–95.

93. Wheelock CE, Shan G, Ottea J. Overview of carboxylesterases and their role in the metabolism of insecticides. J Pestic Sci. 2005;30**:**75–83.

94. Durand N, Carot-Sans G, Bozzolan F, Rosell G, Siaussat D, Debernard S, Chertemps T, Maibeche-Coisne M. Degradation of pheromone and plant volatile components by a same odorant-degrading enzyme in the cotton leafworm, Spodoptera littoralis. PLoS One. 2011;6**:**e29147.

95. Yu QY, Lu C, Li WL, Xiang ZH, Zhang Z. Annotation and expression of carboxylesterases in the silkworm, *Bombyx mori*. BMC Genomics. 2009;10**:**553.

96. Tartar A, Wheeler MM, Zhou X, Coy MR, Boucias DG, Scharf ME. Parallel metatranscriptome analyses of host and symbiont gene expression in the gut of the termite *Reticulitermes flavipes*. Biotechnol Biofuels. 2009;2**:**25.

97. Riddiford LM, Hiruma K, Zhou XF, Nelson CA. Insights into the molecular basis of the hormonal control of molting and metamorphosis from *Manduca sexta* and *Drosophila melanogaster*. Insect Biochem Mol Biol. 2003;33**:**1327–38.

98. Oakeshott J, Claudianos C, Campbell P, Newcomb R, Russell R. Biochemical genetics and genomics of insect esterases**.** In: Gilbert L, Iatrou K, Gill S (editors). *Comprehensive Molecular Insect Science.* London: Elsevier; 2005. 309–61.

99. Devonshire AL, Moores GD. A carboxylesterase with broad substrate specificity causes organophosphorus, carbamate and pyrethroid resistance in peach-potato aphids (*Myzus persicae*). Pestic Biochem Phys. 1982;18**:**235–46.

100. Newcomb RD, Campbell PM, Ollis DL, Cheah E, Russell RJ, Oakeshott JG. A single amino acid substitution converts a carboxylesterase to an organophosphorus hydrolase and confers insecticide resistance on a blowfly. Proc Natl Acad Sci U S A. 1997;94**:**7464–8.

101. Soderlund DM. Molecular Mechanisms of Insecticide Resistance**.** In: Sjut V (editor). *Molecular Mechanisms of Resistance to Agrochemicals.* Berlin Heidelberg: Springer Berlin Heidelberg; 1997. 21–56.

102. Scharf ME, Boucias DG. Potential of termite-based biomass pre-treatment strategies for use in bioethanol production. Insect Sci. 2010;17**:**166–74.

103. Despres L, David JP, Gallet C. The evolutionary ecology of insect resistance to plant chemicals. Trends Ecol Evol. 2007;22**:**298–307.

104. Small GJ, Hemingway J. Molecular characterization of the amplified carboxylesterase gene associated with organophosphorus insecticide resistance in the brown planthopper, *Nilaparvata lugens*. Insect Mol Biol. 2000;9**:**647–53.

105. Dawson GW, Griffiths DC, Pickett JA, Wadhams LJ, Woodcock CM. Plant-derived synergists of alarm pheromone from turnip aphid, *Lipaphis* (*Hyadaphis*) *erysimi* (Homoptera, Aphididae). J Chem Ecol. 1987;13**:**1663–71.

106. Husebye H, Arzt S, Burmeister WP, Hartel FV, Brandt A, Rossiter JT, Bones AM. Crystal structure at 1.1 Angstroms resolution of an insect myrosinase from *Brevicoryne brassicae* shows its close relationship to beta-glucosidases. Insect Biochem Mol Biol. 2005;35**:**1311–20.

107. Beran F, Mewis I, Srinivasan R, Svoboda J, Vial C, Mosimann H, Boland W, Buttner C, Ulrichs C, Hansson BS, Reinecke A. Male *Phyllotreta striolata* (F.) Produce an aggregation pheromone: Identification of male-specific compounds and interaction with host plant volatiles. J Chem Ecol. 2011;37**:**85–97.

108. Tóth M, Csonka E, Bartelt RJ, Cosse AA, Zilkowski BW. Similarities in pheromonal communication of flea beetles *Phyllotreta cruciferae* Goeze and *Ph*. *vittula* Redtenbacher (Coleoptera, Chrysomelidae). J Appl Entomol. 2012;136**:**688–97.

109. Tóth M, Csonka E, Bartelt RJ, Cosse AA, Zilkowski BW, Muto SE, Mori K. Pheromonal activity of compounds identified from male *Phyllotreta cruciferae*: field tests of racemic mixtures, pure enantiomers, and combinations with allyl isothiocyanate. J Chem Ecol. 2005;31**:**2705–20.

110. Crook DJ, Lance DR, Mastro VC. Identification of a potential third component of the male-produced pheromone of *Anoplophora glabripennis* and its effect on behavior. J Chem Ecol. 2014;40**:**1241–50.

111. Zhang AJ, Oliver JE, Aldrich JR, Wang BD, Mastro VC. Stimulatory beetle volatiles for the Asian longhorned beetle, *Anoplophora glabripennis* (Motschulsky). Z Naturforsch C. 2002;57**:**553–8.

112. Gleadow RM, Moller BL. Cyanogenic glycosides: synthesis, physiology, and phenotypic plasticity. Annu Rev Plant Biol. 2014;65**:**155–85.

113. Zagrobelny M, Bak S, Rasmussen AV, Jorgensen B, Naumann CM, Lindberg Moller B. Cyanogenic glucosides and plant-insect interactions. Phytochemistry. 2004;65**:**293–306.

114. Haack RA, Herard F, Sun JH, Turgeon JJ. Managing invasive populations of Asian longhorned beetle and citrus longhorned beetle: a worldwide perspective. Annu Rev Entomol. 2010;55**:**521–46.

115. Rask L, Andreasson E, Ekbom B, Eriksson S, Pontoppidan B, Meijer J. Myrosinase: gene family evolution and herbivore defense in Brassicaceae. Plant Mol Biol. 2000;42**:**93–113.

116. Marana SR, Terra WR, Ferreira C. Purification and properties of a beta-glycosidase purified from midgut cells of *Spodoptera frugiperda* (Lepidoptera) larvae. Insect Biochem Mol Biol. 2000;30**:**1139–46.

117. Henrissat B, Callebaut I, Fabrega S, Lehn P, Mornon JP, Davies G. Conserved catalytic machinery and the prediction of a common fold for several families of glycosyl hydrolases. Proc Natl Acad Sci U S A. 1995;92**:**7090–4.

118. Burmeister WP, Cottaz S, Rollin P, Vasella A, Henrissat B. High resolution x-ray crystallography shows that ascorbate is a cofactor for myrosinase and substitutes for the function of the catalytic base. J Biol Chem. 2000;275**:**39385–93.

119. Burmeister WP, Cottaz S, Driguez H, Iori R, Palmieri S, Henrissat B. The crystal structures of *Sinapis alba* myrosinase and a covalent glycosyl-enzyme intermediate provide insights into the substrate recognition and active-site machinery of an S-glycosidase. Structure. 1997;5**:**663–75.

120. Magrane M, UniProt Consortium. UniProt Knowledgebase: a hub of integrated protein data. Database. 2011;2011**:**bar009.

121. El Hassouni M, Henrissat B, Chippaux M, Barras F. Nucleotide sequences of the arb genes, which control beta-glucoside utilization in *Erwinia chrysanthemi*: comparison with the *Escherichia coli* bgl operon and evidence for a new beta-glycohydrolase family including enzymes from eubacteria, archeabacteria, and humans. J Bacteriol. 1992;174**:**765–77.

122. Gonzalez-Candelas L, Ramon D, Polaina J. Sequences and homology analysis of two genes encoding beta-glucosidases from *Bacillus polymyxa*. Gene. 1990;95**:**31–8.

123. Henrissat B. A classification of glycosyl hydrolases based on amino acid sequence similarities. Biochem J. 1991;280 ( Pt 2)**:**309–16.

124. Henrissat B, Bairoch A. New families in the classification of glycosyl hydrolases based on amino acid sequence similarities. Biochem J. 1993;293 ( Pt 3)**:**781–8.

125. Withers SG, Warren RAJ, Street IP, Rupitz K, Kempton JB, Aebersold R. Unequivocal demonstration of the involvement of a glutamate residue as a nucleophile in the mechanism of a retaining glycosidase. J Am Chem Soc. 1990;112**:**5887–9.

126. Cicek M. Mechanism of Substrate Specificity and Catalysis in Retaining B-glucosidases from Maize and Sorghum**.** Department of Biology, Virginia Tech (URN etd-093099-112234) 1999.

127. Jones DT, Taylor WR, Thornton JM. The rapid generation of mutation data matrices from protein sequences. CABIOS Comput Appl Biosci. 1992;8**:**275–82.

128. SciPy: Open source scientific tools for Python. <http://www.scipy.org/> [Online; accessed 2015-01-01] [<http://www>. scipy. org ]

129. Schwede T, Kopp J, Guex N, Peitsch MC. SWISS-MODEL: An automated protein homology-modeling server. Nucleic Acids Res. 2003;31**:**3381–5.

130. Hopkins RJ, van Dam NM, van Loon JJ. Role of glucosinolates in insect-plant relationships and multitrophic interactions. Annu Rev Entomol. 2009;54**:**57–83.

131. Hegnauer R. Chemotaxonomie der Pflanzen (Birkhaeuser Verlag, Basel and Stuttgart). vol. 3, Dicotyledoneae: Acanthaceae-Cyrillaceae. R. Hegnauer. *.* Birkhäuser, Basel, Switzerland; 1964.

132. USDA Forest Products Laboratory FS. Extractives in Eastern Hardwoods - A review*.* Madison, Wisconsin 1979.

133. Bernays EA, Chapman RF. Host-Plant Selection by Phytophagous Insects, *.* Chapman & Hall, New York, NY.; 1994.

134. Sarate PJ, Tamhane VA, Kotkar HM, Ratnakaran N, Susan N, Gupta VS, Giri AP. Developmental and digestive flexibilities in the midgut of a polyphagous pest, the cotton bollworm, *Helicoverpa armigera*. J Insect Sci. 2012;12**:**42.

135. Cates RG. Feeding patterns of monophagous, oligophagous, and polyphagous insect herbivores - the effect of resource abundance and plant chemistry. Oecologia. 1980;46**:**22–31.

136. Ryan CA. Protease inhibitors in plants - genes for improving defenses against insects and pathogens. Annu Rev Phytopathol. 1990;28**:**425–49.

137. Jongsma MA, Bakker PL, Peters J, Bosch D, Stiekema WJ. Adaptation of *Spodoptera exigua* larvae to plant proteinase-inhibitors by induction of gut proteinase activity insensitive to inhibition. Proc Natl Acad Sci U S A. 1995;92**:**8041–5.

138. Song HK, Suh SW. Kunitz-type soybean trypsin inhibitor revisited: refined structure of its complex with porcine trypsin reveals an insight into the interaction between a homologous inhibitor from *Erythrina caffra* and tissue-type plasminogen activator. J Mol Biol. 1998;275**:**347–63.

139. Broadway RM. Are insects resistant to plant proteinase-inhibitors. J Insect Physiol. 1995;41**:**107–16.

140. Patankar AG, Giri AP, Harsulkar AM, Sainani MN, Deshpande VV, Ranjekar PK, Gupta VS. Complexity in specificities and expression of *Helicoverpa armigera* gut proteinases explains polyphagous nature of the insect pest. Insect Biochem Mol Biol. 2001;31**:**453–64.

141. Jones P, Binns D, Chang HY, Fraser M, Li W, McAnulla C, McWilliam H, Maslen J, Mitchell A, Nuka G, et al. InterProScan 5: genome-scale protein function classification. Bioinformatics. 2014;30**:**1236–40.

142. Leal WS. Odorant reception in insects: Roles of receptors, binding proteins, and degrading enzymes. Annu Rev Entomol. 2013;58**:**373–91.

143. Sánchez-Gracia A, Vieira FG, Rozas J. Molecular evolution of the major chemosensory gene families in insects. Heredity. 2009;103**:**208–16.

144. Mitchell RF, Hughes DT, Luetje CW, Millar JG, Soriano-Agaton F, Hanks LM, Robertson HM. Sequencing and characterizing odorant receptors of the cerambycid beetle *Megacyllene caryae*. Insect Biochem Mol Biol. 2012;42**:**499–505.

145. Fêret S, Maleszka R. Function and evolution of a gene family encoding odorant binding-like proteins in a social insect, the honey bee (*Apis mellifera*). Genome Res. 2006;16**:**1404–13.

146. Xu PX, Zwiebel LJ, Smith DP. Identification of a distinct family of genes encoding atypical odorant-binding proteins in the malaria vector mosquito, *Anopheles gambiae*. Insect Mol Biol. 2003;12**:**549–60.

147. Andersson MN, Grosse-Wilde E, Keeling CI, Bengtsson JM, Yuen MMS, Li M, Hillbur Y, Bohlmann J, Hansson BS, Schlyter F. Antennal transcriptome analysis of the chemosensory gene families in the tree killing bark beetles, *Ips typographus* and *Dendroctonus ponderosae* (Coleoptera: Curculionidae: Scolytinae). BMC Genomics. 2013;14**:**198.

148. Hunt T, Bergsten J, Levkanicova Z, Papadopoulou A, John OS, Wild R, Hammond PM, Ahrens D, Balke M, Caterino MS, et al. A comprehensive phylogeny of beetles reveals the evolutionary origins of a superradiation. Science. 2007;318**:**1913–6.

149. Missbach C, Dweck HKM, Vogel H, Vilcinskas A, Stensmyr MC, Hansson BS, Grosse-Wilde E. Evolution of insect olfactory receptors. Elife. 2014;3.

150. Nolte A, Funk NW, Mukunda L, Gawalek P, Werckenthin A, Hansson BS, Wicher D, Stengl M. In situ tip-recordings found no evidence for an Orco-based ionotropic mechanism of pheromone-transduction in *Manduca sexta*. PLoS One. 2013;8.

151. Sato K, Pellegrino M, Nakagawa T, Nakagawa T, Vosshall LB, Touhara K. Insect olfactory receptors are heteromeric ligand-gated ion channels. Nature. 2008;452**:**1002–9.

152. Wicher D, Schafer R, Bauernfeind R, Stensmyr MC, Heller R, Heinemann SH, Hansson BS. *Drosophila* odorant receptors are both ligand-gated and cyclic-nucleotide-activated cation channels. Nature. 2008;452**:**1007–10.

153. Kirkness EF, Haas BJ, Sun WL, Braig HR, Perotti MA, Clark JM, Lee SH, Robertson HM, Kennedy RC, Elhaik E, et al. Genome sequences of the human body louse and its primary endosymbiont provide insights into the permanent parasitic lifestyle. Proc Natl Acad Sci U S A. 2010;107**:**12168–73.

154. Robertson HM, Warr CG, Carlson JR. Molecular evolution of the insect chemoreceptor gene superfamily in *Drosophila melanogaster*. Proc Natl Acad Sci U S A. 2003;100**:**14537–42.

155. Zhou X, Slone JD, Rokas A, Berger SL, Liebig J, Ray A, Reinberg D, Zwiebel LJ. Phylogenetic and transcriptomic analysis of chemosensory receptors in a pair of divergent ant species reveals sex-specific signatures of odor coding. PLoS Genet. 2012;8**:**e1002930.

156. Leary GP, Allen JE, Bunger PL, Luginbill JB, Linn CE, Jr., Macallister IE, Kavanaugh MP, Wanner KW. Single mutation to a sex pheromone receptor provides adaptive specificity between closely related moth species. Proc Natl Acad Sci U S A. 2012;109**:**14081–6.

157. Zhang A, Oliver JE, Chauhan K, Zhao B, Xia L, Xu Z. Evidence for contact sex recognition pheromone of the Asian longhorned beetle, *Anoplophora glabripennis* (Coleoptera: Cerambycidae). Naturwissenschaften. 2003;90**:**410–3.

158. Koh TW, He Z, Gorur-Shandilya S, Menuz K, Larter NK, Stewart S, Carlson JR. The *Drosophila* IR20a clade of ionotropic receptors are candidate taste and pheromone receptors. Neuron. 2014;83**:**850–65.

159. Miyazono K, Kamiya Y, Morikawa M. Bone morphogenetic protein receptors and signal transduction. J Biochem. 2010;147**:**35–51.

160. Van der Zee M, da Fonseca RN, Roth S. TGFbeta signaling in *Tribolium*: vertebrate-like components in a beetle. Dev Genes Evol. 2008;218**:**203–13.

161. Özüak O, Buchta T, Roth S, Lynch JA. Dorsoventral polarity of the *Nasonia* embryo primarily relies on a BMP gradient formed without input from Toll. Curr Biol. 2014;24**:**2393–8.

162. Richards S, Gibbs RA, Weinstock GM, Brown SJ, Denell R, Beeman RW, Gibbs R, Bucher G, Friedrich M, Grimmelikhuijzen CJP, et al. The genome of the model beetle and pest Tribolium castaneum. Nature. 2008;452**:**949–55.

163. Rongo C, Lehmann R. Regulated synthesis, transport and assembly of the *Drosophila* germ plasm. Trends Genet. 1996;12**:**102–9.

164. Lynch JA, Ozuak O, Khila A, Abouheif E, Desplan C, Roth S. The phylogenetic origin of oskar coincided with the origin of maternally provisioned germ plasm and pole cells at the base of the Holometabola. PLoS Genet. 2011;7**:**e1002029.

165. Schmitt-Engel C, Cerny AC, Schoppmeier M. A dual role for nanos and pumilio in anterior and posterior blastodermal patterning of the short-germ beetle *Tribolium castaneum*. Dev Biol. 2012;364**:**224–35.

166. Krumlauf R. Evolution of the vertebrate Hox homeobox genes. Bioessays. 1992;14**:**245–52.

167. Negre B, Ruiz A. HOM-C evolution in *Drosophila*: is there a need for Hox gene clustering? Trends Genet. 2007;23**:**55–9.

168. Panfilio KA, Liu PZ, Akam M, Kaufman TC. *Oncopeltus fasciatus* zen is essential for serosal tissue function in katatrepsis. Dev Biol. 2006;292**:**226–43.

169. Panfilio KA, Akam M. A comparison of Hox3 and Zen protein coding sequences in taxa that span the Hox3/zen divergence. Dev Genes Evol. 2007;217**:**323–9.

170. Brown SJ, Fellers JP, Shippy TD, Richardson EA, Maxwell M, Stuart JJ, Denell RE. Sequence of the *Tribolium castaneum* Homeotic complex: The region corresponding to the *Drosophila melanogaster* Antennapedia complex. Genetics. 2002;160**:**1067–74.

171. Gómez-Skarmeta JL, Diez del Corral R, delaCalle-Mustienes E, Ferré-Marcó D, Modolell J. Araucan and caupolican, two members of the novel Iroquois complex, encode homeoproteins that control proneural and vein-forming genes. Cell. 1996;85**:**95–105.

172. Kehl BT, Cho KO, Choi KW. Mirror, a *Drosophila* homeobox gene in the iroquois complex, is required for sensory organ and alula formation. Development. 1998;125**:**1217–27.

173. McNeill H, Yang CH, Brodsky M, Ungos J, Simon MA. Mirror encodes a novel PBX-class homeoprotein that functions in the definition of the dorsal-ventral border in the *Drosophila* eye. Gene Dev. 1997;11**:**1073–82.

174. Cavodeassi F, Modolell J, Gomez-Skarmeta JL. The Iroquois family of genes: from body building to neural patterning. Development. 2001;128**:**2847–55.

175. Maeso I, Irimia M, Tena JJ, Gonzalez-Perez E, Tran D, Ravi V, Venkatesh B, Campuzano S, Gomez-Skarmeta JL, Garcia-Fernandez J. An ancient genomic regulatory block conserved across bilaterians and its dismantling in tetrapods by retrogene replacement. Genome Res. 2012;22**:**642–55.

176. Peters T, Dildrop R, Ausmeier K, Ruther U. Organization of mouse Iroquois homeobox genes in two clusters suggests a conserved regulation and function in vertebrate development. Genome Res. 2000;10**:**1453–62.

177. Lynch JA, Roth S. The evolution of dorsal-ventral patterning mechanisms in insects. Gene Dev. 2011;25**:**107–18.

178. Nieto MA. The snail superfamily of zinc-finger transcription factors. Nat Rev Mol Cell Bio. 2002;3**:**155–66.

179. Nambu JR, Lewis JO, Wharton KA, Crews ST. The *Drosophila* single-minded gene encodes a helix-loop-helix protein that acts as a master regulator of cns midline development. Cell. 1991;67**:**1157–67.

180. Von Ohlen T, Doe CQ. Convergence of dorsal, Dpp, and Egfr signaling pathways subdivides the *Drosophila* neuroectoderm into three dorsal-ventral columns. Dev Biol. 2000;224**:**362–72.

181. Calleja M, Herranz H, Estella C, Casal J, Lawrence P, Simpson P, Morata G. Generation of medial and lateral dorsal body domains by the pannier gene of *Drosophila*. Development. 2000;127**:**3971–80.

182. Nunes da Fonseca R, von Levetzow C, Kalscheuer P, Basal A, van der Zee M, Roth S. Self-regulatory circuits in dorsoventral axis formation of the short-germ beetle *Tribolium castaneum*. Dev Cell. 2008;14**:**605–15.

183. Van der Zee M, Stockhammer O, Von Levetzow C, Da Fonseca RN, Roth S. Sog/Chordin is required for ventral-to-dorsal Dpp/BMP transport and head formation in a short germ insect. Proc Natl Acad Sci U S A. 2006;103**:**16307–12.

184. Stathopoulos A, Van Drenth M, Erives A, Markstein M, Levine M. Whole-genome analysis of dorsal-ventral patterning in the *Drosophila* embryo. Cell. 2002;111**:**687–701.

185. Lynch JA, Peel AD, Drechsler A, Averof M, Roth S. EGF signaling and the origin of axial polarity among the insects. Curr Biol. 2010;20**:**1042–47.

186. Evans RM. The nuclear receptor superfamily: A Rosetta Stone for physiology. Mol Endocrinol. 2005;19**:**1429–38.

187. Mangelsdorf DJ, Thummel C, Beato M, Herrlich P, Schutz G, Umesono K, Blumberg B, Kastner P, Mark M, Chambon P, Evans RM. The nuclear receptor superfamily - the 2nd decade. Cell. 1995;83**:**835–39.

188. Auwerx J, Baulieu E, Beato M, Becker-Andre M, Burbach PH, Camerino G, Chambon P, Cooney A, Dejean A, Dreyer C, et al. A unified nomenclature system for the nuclear receptor superfamily. Cell. 1999;97**:**161–3.

189. Gronemeyer H, Gustafsson JA, Laudet V. Principles for modulation of the nuclear receptor superfamily. Nat Rev Drug Discov. 2004;3**:**950–64.

190. Moore JT, Collins JL, Pearce KH. The nuclear receptor superfamily and drug discovery. Chemmedchem. 2006;1**:**504–23.

191. Ribeiro RCJ, Kushner PJ, Baxter JD. The nuclear hormone-receptor gene superfamily. Annu Rev Med. 1995;46**:**443–53.

192. Palli SR, Hormann RE, Schlattner U, Lezzi M. Ecdysteroid receptors and their applications in agriculture and medicine. Vitam Horm. 2005;73**:**59–100.

193. Enmark E, Gustafsson JA. Nematode genome sequence dramatically extends the nuclear receptor superfamily. Trends Pharmacol Sci. 2000;21**:**85–7.

194. King-Jones K, Thummel CS. Nuclear receptors - a perspective from *Drosophila*. Nat Rev Genet. 2005;6**:**311–23.

195. Tan A, Palli SR. Identification and characterization of nuclear receptors from the red flour beetle, *Tribolium castaneum*. Insect Biochem Mol Biol. 2008;38**:**430–9.

196. Jacobs CGC, Spaink HP, van der Zee M. The extraembryonic serosa is a frontier epithelium providing the insect egg with a full-range innate immune response. Elife. 2014;3.

197. LeMosy EK, Kemler D, Hashimoto C. Role of Nudel protease activation in triggering dorsoventral polarization of the *Drosophila* embryo. Development. 1998;125**:**4045–53.

198. Sen J, Goltz JS, Stevens L, Stein D. Spatially restricted expression of pipe in the *Drosophila* egg chamber defines embryonic dorsal-ventral polarity. Cell. 1998;95**:**471–81.

199. Tomoyasu Y, Denell RE. Larval RNAi in *Tribolium* (Coleoptera) for analyzing adult development. Dev Genes Evol. 2004;214**:**575–8.

200. Miller SC, Miyata K, Brown SJ, Tomoyasu Y. Dissecting systemic RNA interference in the red flour beetle *Tribolium castaneum*: Parameters affecting the efficiency of RNAi. PLoS One. 2012;7**:**e47431.

201. Bellés X. Beyond *Drosophila*: RNAi in vivo and functional genomics in insects. Annu Rev Entomol. 2010;55**:**111–28.

202. Horn T, Sandmann T, Boutros M. Design and evaluation of genome-wide libraries for RNA interference screens. Genome Biol. 2010;11**:**R61.

203. Kamath RS, Ahringer J. Genome-wide RNAi screening in *Caenorhabditis elegans*. Methods. 2003;30**:**313–21.

204. Rual JF, Ceron J, Koreth J, Hao T, Nicot AS, Hirozane-Kishikawa T, Vandenhaute J, Orkin SH, Hill DE, van den Heuvel S, Vidal M. Toward improving *Caenorhabditis elegans* phenome mapping with an ORFeome-based RNAi library. Genome Res. 2004;14**:**2162–8.

205. Young M, Beeman RW, Arakane Y. RNAi-based functional genomics in *Tribolium castaneum* and possible application for controlling insect pests. Entomol Res. 2012;42**:**1–10.

206. Li XX, Zhang MY, Zhang HY. RNA interference of four genes in adult *Bactrocera dorsalis* by feeding their dsRNAs. PLoS One. 2011;6.

207. Tian HG, Peng H, Yao Q, Chen HX, Xie Q, Tang B, Zhang WQ. Developmental control of a lepidopteran pest *Spodoptera exigua* by ingestion of bacteria expressing dsRNA of a non-midgut gene. PLoS One. 2009;4**:**e6225.

208. Walshe DP, Lehane SM, Lehane MJ, Haines LR. Prolonged gene knockdown in the tsetse fly *Glossina* by feeding double stranded RNA. Insect Mol Biol. 2009;18**:**11–19.

209. Alves AP, Lorenzen MD, Beeman RW, Foster JE, Siegfried BD. RNA interference as a method for target-site screening in the western corn rootworm, *Diabrotica virgifera virgifera*. J Insect Sci. 2010;10.

210. Mao YB, Cai WJ, Wang JW, Hong GJ, Tao XY, Wang LJ, Huang YP, Chen XY. Silencing a cotton bollworm P450 monooxygenase gene by plant-mediated RNAi impairs larval tolerance of gossypol. Nat Biotechnol. 2007;25**:**1307–13.

211. Timmons L, Court DL, Fire A. Ingestion of bacterially expressed dsRNAs can produce specific and potent genetic interference in *Caenorhabditis elegans*. Gene. 2001;263**:**103–12.

212. Miyata K, Ramaseshadri P, Zhang YJ, Segers G, Bolognesi R, Tomoyasu Y. Establishing an in vivo assay system to identify components involved in environmental RNA interference in the western corn rootworm. PLoS One. 2014;9.

213. Willis JH. Structural cuticular proteins from arthropods: annotation, nomenclature, and sequence characteristics in the genomics era. Insect Biochem Mol Biol. 2010;40**:**189–204.

214. Ioannidou ZS, Theodoropoulou MC, Papandreou NC, Willis JH, Hamodrakas SJ. CutProtFam-Pred: detection and classification of putative structural cuticular proteins from sequence alone, based on profile hidden Markov models. Insect Biochem Mol Biol. 2014;52**:**51–9.

215. Arakane Y, Hogenkamp DG, Zhu YC, Kramer KJ, Specht CA, Beeman RW, Kanost MR, Muthukrishnan S. Characterization of two chitin synthase genes of the red flour beetle, *Tribolium castaneum*, and alternate exon usage in one of the genes during development. Insect Biochem Mol Biol. 2004;34**:**291–304.

216. Bird AP. DNA methylation and the frequency of cpg in animal DNA. Nucleic Acids Res. 1980;8**:**1499–504.

217. Elango N, Hunt BG, Goodisman MAD, Yi SV. DNA methylation is widespread and associated with differential gene expression in castes of the honeybee, *Apis mellifera*. Proc Natl Acad Sci U S A. 2009;106**:**11206–11.

218. Glastad KM, Hunt BG, Yi SV, Goodisman MAD. DNA methylation in insects: on the brink of the epigenomic era. Insect Mol Biol. 2011;20**:**553–65.

219. Simola DF, Wissler L, Donahue G, Waterhouse RM, Helmkampf M, Roux J, Nygaard S, Glastad KM, Hagen DE, Viljakainen L, et al. Social insect genomes exhibit dramatic evolution in gene composition and regulation while preserving regulatory features linked to sociality. Genome Res. 2013;23**:**1235–47.

220. Yi SV, Goodisman MAD. Computational approaches for understanding the evolution of DNA methylation in animals. Epigenetics. 2009;4**:**551–6.

221. Wang Y, Jorda M, Jones PL, Maleszka R, Ling X, Robertson HM, Mizzen CA, Peinado MA, Robinson GE. Functional CpG methylation system in a social insect. Science. 2006;314**:**645–7.

222. Kent CF, Minaei S, Harpur BA, Zayed A. Recombination is associated with the evolution of genome structure and worker behavior in honey bees. Proc Natl Acad Sci U S A. 2012;109**:**18012–7.

223. Rae PMM, Steele RE. Absence of cytosine methylation at C-C-G-G and G-C-G-C sites in the rDNA coding regions and intervening sequences of *Drosophila* and the rDNA of other higher insects. Nucleic Acids Res. 1979;6**:**2987–95.

224. Zemach A, McDaniel IE, Silva P, Zilberman D. Genome-wide evolutionary analysis of eukaryotic DNA methylation. Science. 2010;328**:**916–9.

225. Hunt BG, Glastad KM, Yi SV, Goodisman MAD. The function of intragenic DNA methylation: insights from insect epigenomes. Integr Comp Biol. 2013;53**:**319–28.

226. Denlinger DL, Yocum GD, Rinehart JP. Hormonal control of diapause**.** In: Gilbert LI (editor). *Insect Endocrinology.* Elsevier; 2012. 430–63.

227. Tauber MJ, Tauber CA, Masaki S. *Seasonal Adaptations of Insects*. Oxford University Press; 1986.

228. Rinehart JP, Li A, Yocum GD, Robich RM, Hayward SA, Denlinger DL. Up-regulation of heat shock proteins is essential for cold survival during insect diapause. Proc Natl Acad Sci U S A. 2007;104**:**11130–7.

229. Lee RE, Denlinger DL. Ontogeny of cold-hardiness in the flesh fly. Cryobiology. 1985;22**:**632–2.

230. Sim C, Denlinger DL. Insulin signaling and the regulation of insect diapause. Front Physiol. 2013;4.

231. Riehle MA, Brown MR. Insulin stimulates ecdysteroid production through a conserved signaling cascade in the mosquito *Aedes aegypti*. Insect Biochem Mol Biol. 1999;29**:**855–60.

232. Nijhout HF. The control of body size in insects. Dev Biol. 2003;261**:**1–9.

233. Brown MR, Clark KD, Gulia M, Zhao Z, Garczynski SF, Crim JW, Sulderman RJ, Strand MR. An insulin-like peptide regulates egg maturation and metabolism in the mosquito *Aedes aegypti*. Proc Natl Acad Sci U S A. 2008;105**:**5716–21.

234. Antonova Y, Arik AJ, Moore W, Riehle MR, Brown MR. Insulin-like peptides: Structure, Signaling, and Function**.** In: Gilbert LI (editor). Insect Endocrinology. Elsevier; 2012. 63–92.

235. Feder ME, Hofmann GE. Heat-shock proteins, molecular chaperones, and the stress response: Evolutionary and ecological physiology. Annu Rev Physiol. 1999;61**:**243–82.

236. Lindquist S. The heat-shock response. Annu Rev Biochem. 1986;55**:**1151–91.

237. Denlinger DL. Regulation of diapause. Annu Rev Entomol. 2002;47**:**93–122.

238. MacRae TH. Gene expression, metabolic regulation and stress tolerance during diapause. Cellular and molecular life sciences: CMLS. 2010;67**:**2405–24.

239. Colinet H, Lee SF, Hoffmann A. Functional characterization of the Frost gene in *Drosophila melanogaster*: Importance for recovery from chill coma. PLoS One. 2010;5**:**e10925.

240. Goto SG. A novel gene that is up-regulated during recovery from cold shock in *Drosophila melanogaster*. Gene. 2001;270**:**259–64.

241. Liou YC, Thibault P, Walker VK, Davies PL, Graham LA. A complex family of highly heterogeneous and internally repetitive hyperactive antifreeze proteins from the beetle *Tenebrio molitor*. Biochemistry. 1999;38**:**11415–24.

242. Zachariassen KE, Husby JA. Antifreeze effect of thermal hysteresis agents protects highly supercooled insects. Nature. 1982;298**:**865–7.

243. Meng PS, Hoover K, Keena MA. Asian longhorned beetle (Coleoptera: Cerambycidae), an introduced pest of maple and other hardwood trees in North America and Europe. J Integr Pest Manag. 2015;6**:**1–13.

244. Nowak DJ, Pasek JE, Sequeira RA, Crane DE, Mastro VC. Potential effect of *Anoplophora glabripennis* (Coleoptera : Cerambycidae) on urban trees in the United States. J Econ Entomol. 2001;94**:**116–22.

245. Haack RA, Law KR, Mastro VC, Ossenbruggen HS, Raimo BJ. New York's battle with the Asian long-horned beetle. J Forest. 1997;95**:**11–5.

246. Hu JF, Angeli S, Schuetz S, Luo YQ, Hajek AE. Ecology and management of exotic and endemic Asian longhorned beetle *Anoplophora glabripennis*. Agr Forest Entomol. 2009;11**:**359–75.

247. Bradnam KR, Fass JN, Alexandrov A, Baranay P, Bechner M, Birol I, Boisvert S, Chapman JA, Chapuis G, Chikhi R*,* et al. Assemblathon 2: evaluating de novo methods of genome assembly in three vertebrate species. GigaScience. 2013;2:10.

248. Parra G, Bradnam K, Korf I. CEGMA: a pipeline to accurately annotate core genes in eukaryotic genomes. Bioinformatics. 2007;23:1061-1067.

249. Keller O, Kollmar M, Stanke M, Waack S. A novel hybrid gene prediction method employing protein multiple sequence alignments. Bioinformatics. 2011;27:757-763.

250. Simao FA, Waterhouse RM, Ioannidis P, Kriventseva EV, Zdobnov EM. BUSCO: assessing genome assembly and annotation completeness with single-copy orthologs. Bioinformatics. 2015;31:3210-3212.

251. Zheng CF, Sankoff D. On the PATHGROUPS approach to rapid small phylogeny. BMC Bioinf. 2011;12**:**54.

252. Fahey JW, Zalcmann AT, Talalay P. The chemical diversity and distribution of glucosinolates and isothiocyanates among plants. Phytochemistry 2001;56(1):5-51.

253. Daxenbichler R, Van Etten CH. Glucosinolates in *Limnanthes alba* Benth. seed. J Amer Oil Chemists' Soc. 1974;51: 449-450.

254. Rodman JE. A taxonomic analysis of glucosinolate-producing plants, Part 2: Cladistics. Syst Bot. 1991;16:619-629.

255. Kjñr A, Malver O, El-Menshaw B, Reisch J. Isothiocyanates in myrosinase-treated seed extracts of *Moringa peregina*. Phytochemistry. 1979;18:1485-1487.

256. Sorensen H. o-( -l-Rhamnopyranosyloxy)benzylamine and o-hydroxybenzylamine in *Reseda odorata*. Phytochemistry. 1970;9:865-870.

1. See VI.3 Homeodomain transcription factors (Hox, Iro-C) for further details on this gene. [↑](#footnote-ref-1)
2. Original report ($889 billion) adjusted for inflation (March 2016). [↑](#footnote-ref-2)
